# The impact of sex on the immune system explored at the single-cell level

**DOI:** 10.1016/j.ajhg.2026.04.003

**Published:** 2026-05-07

**Authors:** Seyhan Yazar, Jose Alquicira-Hernandez, Kristof Wing, Anne Senabouth, Stacey Andersen, Kirsten A. Fairfax, Alex W. Hewitt, Joseph E. Powell, Sara Ballouz

**Affiliations:** 1 Precision Immunology, Garvan Institute of Medical Research, Sydney, NSW, Australia; 2 School of Clinical Medicine, UNSW Medicine and Health, Sydney, NSW, Australia; 3 Translational Genomics, Garvan Institute of Medical Research, Sydney, NSW, Australia; 4 Tasmanian School of Medicine, University of Tasmania, Hobart, TAS, Australia; 5 Institute for Molecular Bioscience, University of Queensland, Brisbane, QLD, Australia; 6 Department of Ophthalmology, Royal Hobart Hospital, Hobart, TAS, Australia; 7 Centre for Eye Research Australia, University of Melbourne, East Melbourne, VIC, Australia; 8 UNSW Cellular Genomics Futures Institute, University of New South Wales, Sydney, NSW, Australia; 9 School of Computer Science and Engineering, University of New South Wales, Sydney, NSW, Australia

**Keywords:** sex differences, immune cells, sex-biased expression, expression quantitative trait loci, sex chromosomes

## Abstract

Sex has a key role in disease susceptibility (in particular, autoimmunity). Sex differences in the immune system originate from genes and their interactions with both intrinsic and extrinsic factors. However, the cellular-level factors influencing sexual dimorphism are not fully understood. We thus examined immune sex differences at single-cell resolution to dissect the genetic impacts. Female-biased sex-differentially expressed genes (sex-DEGs) in multiple immune cells were involved in tumor necrosis factor alpha (TNF-α) signaling, whereas male DEGs were enriched for ribosomal-related functions. While *cis*-expression trait quantitative loci (eQTLs) were less common on sex chromosomes, we identified over 1,000 sex-specific eQTLs and 51 sex-interacting eQTLs on autosomes. When we examined the effect of genetic control on sex-DEGs, we found genetic variants affecting the female-biased expression of *FCGR3A* in natural killer (NK) cells (rs2099684) and *ITGB2* in monocytes (rs760462), both of which are associated with systemic lupus erythematosus. Our work reveals biases masked in bulk analyses and highlights sexually dimorphic genes and pathways at baseline.

## Graphical abstract

**Figure:**
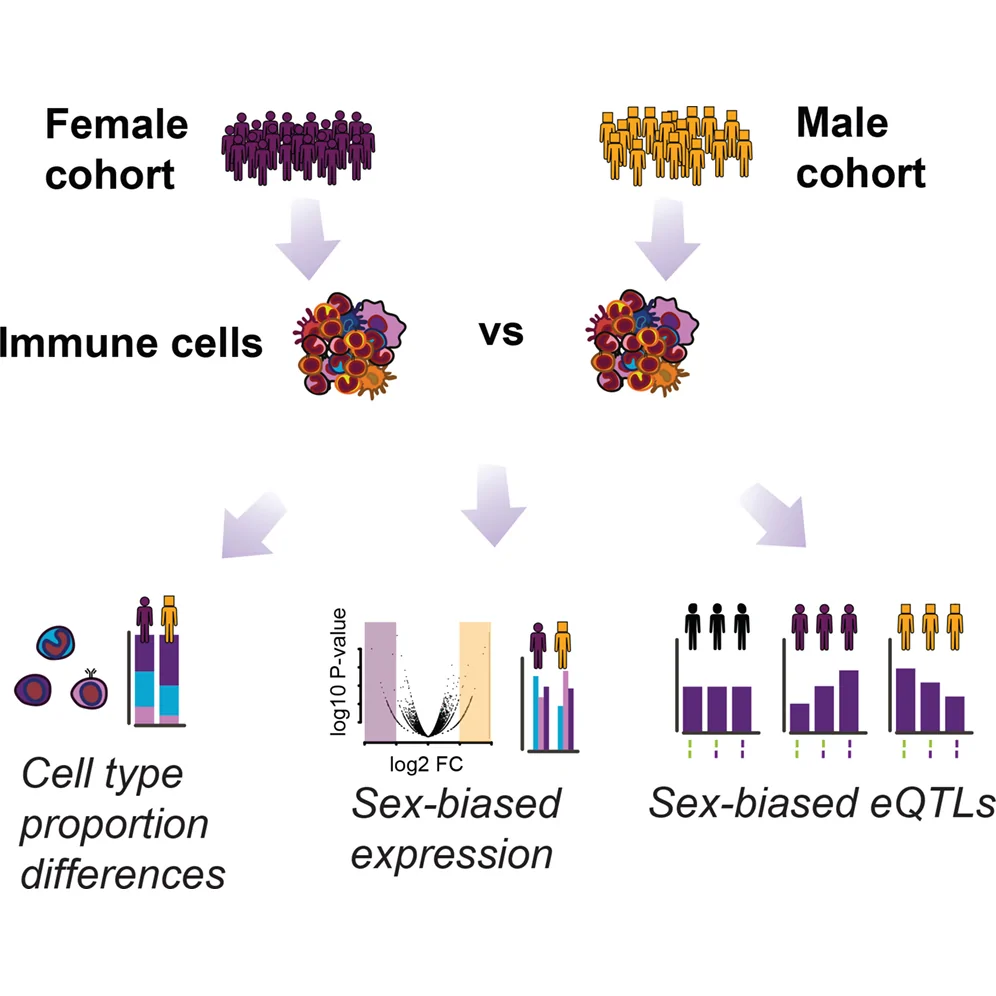

## Teaser

Yazar et al. characterized sex-based immune differences at single-cell resolution using sex-differential expression (differentially expressed genes [DEGs]) and sex-stratified expression quantitative loci (eQTLs). They identified sex-biased genes and pathways, including those previously linked to autoimmune diseases, suggesting that baseline sexual dimorphism in the immune system provides a molecular foundation for sex-specific disease susceptibility.

## Introduction

Sex differences in the immune system play a critical role in the susceptibility, progression and outcome of autoimmune diseases. These differences are evident in immune parameters such as antigen presentation strength and duration and intensity of immune responses, all of which vary between females and males. For instance, in females, vaccine responses are typically stronger and the rates of chronic viral infections and degree of viremia (e.g., in HIV [MIM: 609423]) are lower. However, the disadvantage of these robust immune responses is that they predispose females to higher rates of autoimmunity and inflammatory diseases. In contrast, males are more likely to develop non-reproductive cancers and become affected by bacterial and parasitic infections than females.^1^^,^^2^

At the cellular level, these clinical differences are quantifiable between males and females in both the innate and adaptive immune compartments. While males exhibit a greater number of circulating natural killer (NK) cells, females display a higher frequency of B cells.^3^ Additionally, females show heightened T cell cytotoxic and inflammatory responses, particularly following multiple stimulations.^4^ At the gene-expression level, most sexually dimorphic traits originate from and are influenced by the sex chromosomes. The X chromosome holds several critical immune-related genes, including interleukin receptors (*IL2RG* [MIM: 308380]), chemokines (*CXCR3* [MIM: 300574]), toll-like receptors (*TLR7* [MIM: 300365], *TLR8* [MIM: 300366]), and genes involved in T cell and B cell effector functions (*BTK* [MIM: 300300], *IKBKG* [MIM: 300248], *NKRF* [MIM: 300440]),^5^ as well as regulatory molecules such as *FOXP3* [MIM: 300292] and *CD40LG* [MIM: 300386] (CD154). Their differential expression is a potential driver of sex-specific immune phenotypic variation and disease.

While differences are observable at the phenotypic and cellular levels as described, they are not apparent at the genetic level. Despite the identification of over 2,000 single-nucleotide variants (SNVs) associated with autoimmune diseases, our understanding of sex-differentiated genetic architecture remains limited. Specifically, only a small fraction of these SNVs demonstrate sex-specific effects. For example, polymorphisms of *TLR7* located on the X chromosome are a well-characterized risk factor for the autoimmune condition systemic lupus erythematosus (SLE [MIM: 301080]), which has a 9:1 prevalence in women compared to men.^6^ We propose that the genetic regulation of sex-biased gene expression may provide additional evidence to clarify this unresolved research area.

Characterization of sexual dimorphism in the adaptive and innate immune systems has previously focused on investigating *a priori*-defined subsets of immune cells or on bulk analyses. While this hypothesis-driven research has demonstrated key phenotypic differences between male and female immune systems, including cell counts, population dynamics, and cytokine production, it can lead to biased and insensitive analyses. As sex differences in the immune system arise from cellular diversity, cell-population composition, and cellular activity,^1^ the application of single-cell analyses uniquely permits the unbiased characterization of sex differences in the peripheral immune system.

**Figure 1:**
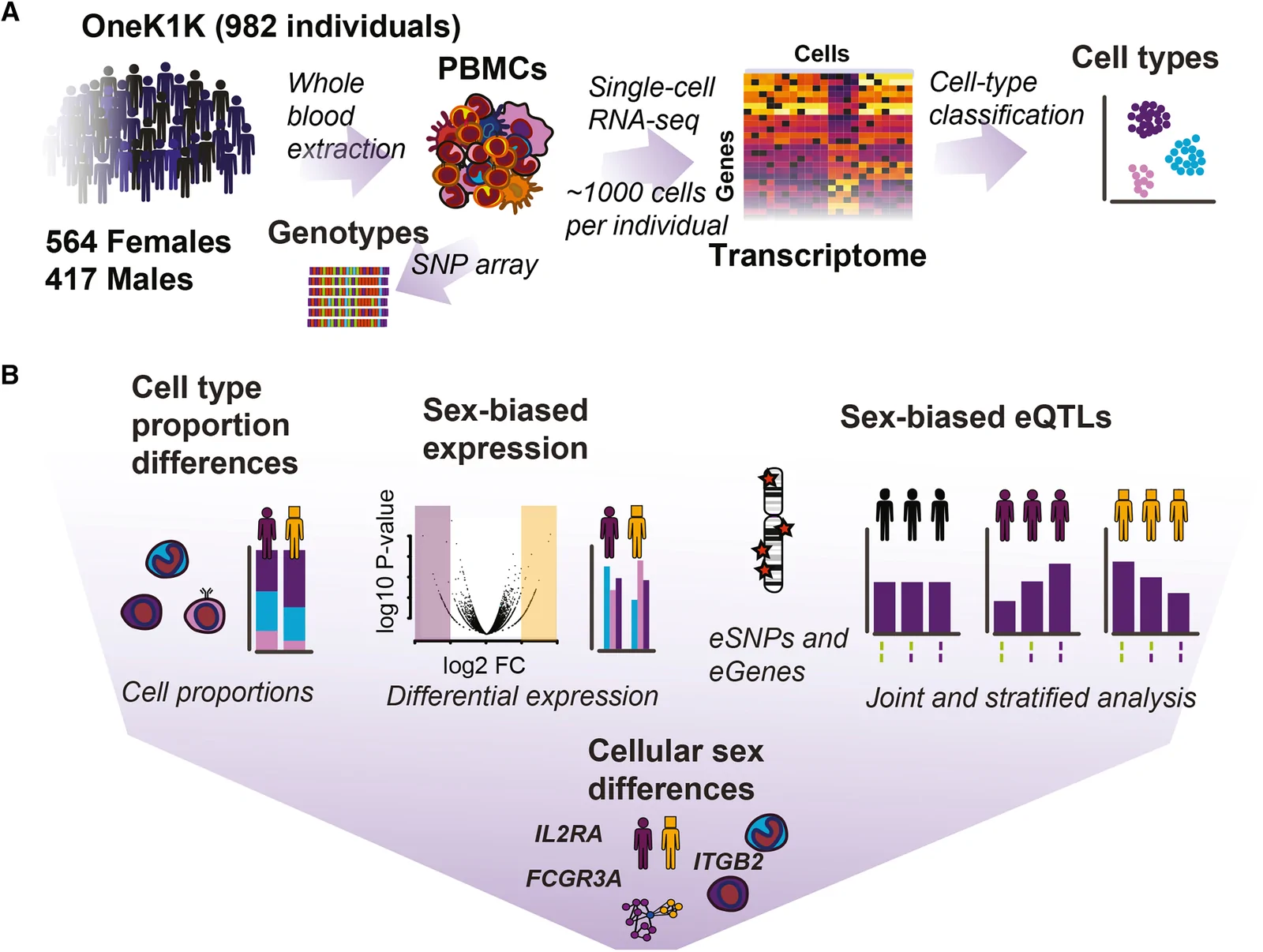
Overview of study (A) Cohort: OneK1K of 982 individuals, with 564 females and 417 males. After single-cell sequencing of ∼1,000 cells per individual, 1,267,758 PBMCs were classified into 30 cell types. (B) Study design. We assessed differences in cell-type proportions, sex-biased differential expression, and sex-biased eQTLs using a statistical association framework.

Here, we combine single-cell gene expression (single-cell RNA sequencing [sc-RNA-seq]) and genetic variation to assess sex differences at the cellular level in peripheral blood mononuclear cells (PBMCs) across a large cohort (Figure 1A). We evaluated cell-type proportions, sex-biased gene expression, and sex-specific and sex-interacting expression quantitative trait loci (eQTLs) along with co-expression networks (Figure 1B). By conditioning our analyses on sex and/or cell type, we disentangle their contributions to better understand the causes and consequences of sexual dimorphism in circulating immune cells.

## Methods

### OneK1K data and cell-type classification

Details of the OneK1K cohort and data generation have been described previously.^7^ Briefly, 1,104 individuals from the Tasmanian Ophthalmic Biobank were recruited, and the study adhered to the tenets of the Declaration of Helsinki and was approved through the Human Research Ethics Committee University of Tasmania (approval number H0012902) and St Vincent’s Hospital Sydney (2020/ETH01307). Written informed consent was sought from all the participants. All participants genotyped using Illumina Infinium Global Screening Array and had PBMCs sequenced using the 10× Genomics Chromium Single Cell 3′ v2 platform. Imputation was performed using the Michigan Imputation Server,^8^ with Minimac4^9^ and the Haplotype Reference Consortium (HRC) panel.^10^

While most participants identified themselves with a northern European ancestry in the study survey, the ancestral relationships were further investigated using genotype information. Individuals of non-European ancestry were excluded to maintain cohort homogeneity.^7^ Following quality control, 982 individuals remained (564 females and 418 males); sex was confirmed through a SNP-based analysis. Approximately 1,200 cells on average were sequenced per individual, totaling 1,267,758 cells across 75 batches (i.e., pools). Samples were multiplexed (10–14 samples per pool), with a target capture of 20,000 cells per pool.

Sequencing was done with the Illumina NovaSeq 6000. Reads were processed using the Cell Ranger Single Cell Software Suite (v 2.2.0; 10× Genomics)^11^ and demultiplexed into their respective pools. Mapping and alignment were performed against GRCh37/hg19 (release 84) reference using STAR^12^ within the Cell Ranger Suite. Batch correction and SCTransform normalization were performed through Seurat (v4.4.0).^13^ Cell types were classified via the Azimuth pipeline with the human PBMC reference for L1 and L2 annotations^14^ (Table S22). Dendritic cells subsets (ASDC, cDC1, cDC2, and pDC) were merged due to low cell counts, and other rare cell types were retained but excluded from specific analyses where higher cell numbers were required. Furthermore, genotype-based principal components (PCs) were extracted from previous work,^7^ with PCs 1–4 used to account for ancestry.

### Cell-type proportions

For each individual, cell-type proportions were calculated as a ratio of specific cell-type counts to the total cell count. To normalize these proportions, we applied both the *logit* and *arcsin* square-root transformation from the Speckle R package.^15^ While both methods were evaluated, we report the results from *arcsin* transformation. The *propeller* function was used to determine whether the average proportions were significantly different between the sexes using an F test on the transformed data.

Normality was assessed post transformation using the Shapiro-Wilk test. Further to this, we ran the Kruskal-Wallis test (non-parametric test) on the data (without transformation) as some of the cell types failed the normality tests. To account for potential confounders, we repeated the F test using a custom design matrix (∼0 + sex + age + PCs1–4) to adjust for age and ancestry. Furthermore, as cell proportions bounded between 0 and 1, we implemented a beta-regression model using the DCATS package.^16^ Specifically, we used the *dcats_GLM()* function with the adjusted design matrix to validate our findings.

### Age correlation analysis

We tested for significant correlation between cell proportions and age using Spearman’s rho for the correlation, and then measured significance with the adjusted *p* value (*cor.test* and *p.adjust* in R^17^). This was done per cell type, first jointly across the sexes, and then stratified by sex.

### Sex-differential expression analysis

We initially performed single-cell differential expression analysis for each cell type with the *FindMarkers* function in Seurat (v4.4.0) using the Wilcoxon test with default parameters and log_2_ fold change (log_2_FC) threshold set to 0. Additionally, to adjust for confounders, we used MAST^18^ within the *FindMarkers* function, incorporating age, donor ID, and PCs 1–4 as latent variables. To address correlated data structures and sample overlap across cell types, we performed a cell-type meta-analysis using multivariate adaptive shrinkage (MASH) via the R package *mashr*.^19^ For all tested genes, we provided *Z* scores derived from the log_2_FC; for genes not tested within a specific cell type, we set the log_2_FC to 0. We used canonical covariances to fit the model and accounted for measurement correlations using the expectation maximization (EM) method as detailed in the *mashr* vignette. Significant sex-differentially expressed genes (sex-DEGs) were filtered using the calculated local false sign rate (LFSR) <0.05 and the cell-type-specific |log_2_FC| >0.1. This log_2_FC threshold was determined based on an original analysis that calculated absolute fold changes of approximately 3 SD.

### Sex-chromosome gene-expression analysis

To validate the sex-specific molecular profiles of captured cells, we performed dimensionality reduction using expression data from X and Y chromosome genes. Uniform manifold approximation and projection (UMAP) was generated via the Seurat R package^13^ (v4.4.0) using sex-linked genes as the variable feature set to partition cells by chromosomal sex. We visualized these clustering results via their UMAP dimensions with the *DimPlot* function in Seurat and highlighted the expression of a subset of genes of interest using the *plot_density* function in Nebulosa.^20^

### Cell-type gene-marker identification

For each cell type, we determined cell-type marker genes using the *FindAllMarkers* function in Seurat with a |log_2_FC| greater than 0.25 and percent expressed 0.25. We then filtered on significance using a false discovery rate (FDR) of 0.05. We performed this per batch and took the genes that recurred in at least 80% of the 75 batches as markers. Finally, we repeated this procedure in a sex-stratified manner to identify cell-type markers that were conditioned on sex, allowing for the characterization of sex-specific patterns within each lineage.

### Classifier and class prediction

To test for the ability of the gene sets to label or classify cells, we used a classification score based on the ranked gene-expression levels and calculated an enrichment statistic (*analytic_auroc* in EGAD^21^). An AUROC score approaching 1 indicates that the gene set is highly specific and consistently expressed within the target cell population, serving as a robust marker for classification.

### Gene sets and functional-enrichment analysis

We curated gene sets from multiple biological domains. We downloaded the Gene Ontology (GO)^22^^,^^23^ and the generic GO slim subset. Additionally, we used MSigDB,^24^ with a focus on the HALLMARK,^25^ Kyoto Encyclopedia of Genes and Genomes (KEGG),^26^ REACTOME,^27^ and BIOCARTA^28^ gene sets and pathways. We downloaded regulatory TFs from MotifMap^29^ and ENCODE TF-target^30^ gene sets curated through Harmonizome.^29^ Furthermore, we generated curated X-linked datasets from sex-differential and X-inactivation analysis papers. We labeled these datasets as Jansen2014,^31^ Mele2015,^32^ Tukiainen2017,^33^ Schmiedel2018,^34^ Bongen2019,^35^ and Oliva2020.^36^ Escape genes were selected from Tukiainen et al.^33^ (Table S14); PAR genes (Table S15) and genes within the major histocompatibility complex (MHC) locus (Table S16) were extracted from GENCODE (v47^37^).

For gene set enrichment analysis, we used the hypergeometric test in R (*phyper*) and adjusted for multiple tests using *p.adjust*. For network assessment and analysis, we ran the neighbor-voting algorithm in EGAD in R,^21^ which uses the guilt-by-association (GBA) principle to assess network connectivity.

### Sex-specific eQTL and sex-interacting eQTL analysis

Here, we define multiple types of sex eQTLs based on their calculations. Sex-specific eQTLs are *cis*-eQTLs that show female or male effects, but not both, when sex stratified. Sex-interacting eQTLs are *cis*-eQTLs that show opposing effects in males and females or weaker effects in one sex versus the other when sex stratified. In some cases where we observe the eQTLs in the joint analysis but only in one sex, we have labeled these as ambiguous. Autosomal eQTLs are sex-specific and sex-interacting *cis*-eQTLs that are tested on the autosomal chromosomes. Sex-chromosome eQTLs are sex-specific *cis*-eQTLs that are tested on the sex chromosomes. The analyses are split into PAR (diploid) and non-PAR (haploid) tests. Finally, we define sex-biased eQTLS as *cis*-eQTLs that show sex-specific effects or are sex interacting from both the autosomes and sex chromosomes. We go through the calculations of each in the next sections.

#### Joint eQTLs

We performed *cis*-eQTL analysis per cell type across all the autosomes jointly (code available from https://github.com/powellgenomicslab/onek1k_phase1). Details of this analysis were described previously.^7^ In brief, average expression of each gene per person across all genes available for each cell type was calculated using the corrected counts with SCTransform.^38^ We then calculated the number of individuals with non-zero expression for each gene and filtered genes expressed in less than 10% of the cohort. All values are then log transformed (log *x* + 1). Within each cell type, *cis*-eQTLs were identified by Spearman’s rank correlation testing using residual expression levels adjusted for sex, age, first four genotype-based PCs, and two PEER factors from original analysis. We restricted our search to variants within 1Mb of the TSS of either end of a gene. The resulting SNP-gene pairs were filtered at the FDR threshold of 5% at the chromosomal level for each cell type, and the most significantly associated SNPs were labeled as *cis*-eQTLs.

Equation 1: joint *cis*-eQTLs

$$\hat{G_{X}}=\beta_{0}+\beta_{S}.sex+\beta_{A}\;.age+\beta_{PC1}\;.PC1\;\ldots +\beta_{PC4}\;.PC4+\beta_{PF1}\;.PF1+\beta_{PF2}\;.PF2$$

$$e_{X_{0}}=G_{X}-\hat{G_{X}}$$

$\hat{G_{X}}$ is a matrix consisting of the average expression of gene X per individual. $e_{X_{0}}$ is the matrix including residual expression of gene X after adjusting for sex, age, six genotyping PCs, and two PEER factors.

$$for\;each\;SNP\;and\;e_{X_{0}}\;pair,\varrho =1-\frac{6\sum d^{2}}{n\left(n^{2}-1\right)}$$

$$\begin{array}{ccccc} \left[\begin{array}{c} \begin{array}{ccc} {SNP}_{v} & \rho_{1} & q_{1}\\ {SNP}_{w} & \rho_{2} & q_{2}\\ {SNP}_{x} & \rho_{3} & q_{3} \end{array}\\ \begin{array}{ccc} {SNP}_{y} & \rho_{4} & q_{4} \end{array}\\ \begin{array}{ccc} {SNP}_{z} & \rho_{5} & q_{5} \end{array}\\ .\\ .\\ . \end{array}\right] & \begin{array}{c} q-value\\ \to \\ ranking \end{array} & \left[\begin{array}{c} \begin{array}{ccc} {SNP}_{x} & \rho_{3} & q_{3}\\ {SNP}_{y} & \rho_{4} & q_{4}\\ {SNP}_{w} & \rho_{2} & q_{2} \end{array}\\ \begin{array}{ccc} {SNP}_{z} & \rho_{5} & q_{5} \end{array}\\ \begin{array}{ccc} {SNP}_{v} & \rho_{1} & q_{1} \end{array}\\ .\\ .\\ . \end{array}\right] & \begin{array}{c} top\;SNP\\ \to \\ determined \end{array} & \begin{array}{c} \left[{SNP}_{x}={eSNP}_{1}\right] \end{array} \end{array}$$

*ρ* is the correlation between $e_{X_{0}}$ and a matrix of three genotypes coded as 0,1 and 2 where 2 represents the assessed allele for each of 5,433,038 SNPs and *q* is the associated *q* value. *d* is the difference between two rankings of residuals (*e*_*A*_), and *n* is the number of measurements.

#### Sex-specific eQTL discovery (stratified approach)

To identify sex-specific eQTLs, we performed a sex-stratified analysis as a discovery step then formally validated these using an interaction model.

##### Step 1: Discovery

In the OneK1K cohort, we have slightly more female participants than males. To ensure equal power to detect eQTLs in each sex, we downsampled the number of female participants in each cell type to match numbers of male participants.

Equation 2: sex-specific cis-eQTLs

$$\hat{G_{X}}=\beta_{0}+\beta_{A}\;.age+\beta_{PC1}\;.PC1\;\ldots +\beta_{PC4}\;.PC4+\beta_{PF1}\;.PF1+\beta_{PF2}\;.PF2$$

$$e_{X_{0}}=G_{X}-\hat{G_{X}}$$

$\hat{G_{X}}$ is a matrix consisting of the average expression of gene X per individual. $e_{X_{0}}$ is the matrix including residual expression of gene X after adjusting for age, six genotyping PCs, and two PEER factors.

$$for\;each\;SNP\;and\;e_{X_{0}}\;pair,\varrho =1-\frac{6\sum d^{2}}{n\left(n^{2}-1\right)}$$

$$\begin{array}{ccccc} \left[\begin{array}{c} \begin{array}{ccc} {SNP}_{v} & \rho_{1} & q_{1}\\ {SNP}_{w} & \rho_{2} & q_{2}\\ {SNP}_{x} & \rho_{3} & q_{3} \end{array}\\ \begin{array}{ccc} {SNP}_{y} & \rho_{4} & q_{4} \end{array}\\ \begin{array}{ccc} {SNP}_{z} & \rho_{5} & q_{5} \end{array}\\ .\\ .\\ . \end{array}\right] & \begin{array}{c} q-value\\ \to \\ ranking \end{array} & \left[\begin{array}{c} \begin{array}{ccc} {SNP}_{x} & \rho_{3} & q_{3}\\ {SNP}_{y} & \rho_{4} & q_{4}\\ {SNP}_{w} & \rho_{2} & q_{2} \end{array}\\ \begin{array}{ccc} {SNP}_{z} & \rho_{5} & q_{5} \end{array}\\ \begin{array}{ccc} {SNP}_{v} & \rho_{1} & q_{1} \end{array}\\ .\\ .\\ . \end{array}\right] & \begin{array}{c} top\;SNP\\ \to \\ determined \end{array} & \begin{array}{c} \left[{SNP}_{x}={eSNP}_{1}\right] \end{array} \end{array}$$

*ρ* is the correlation between $e_{X_{0}}$ and a matrix of three genotypes coded as 0,1 and 2, where 2 represents the assessed allele for each of 5,433,038 SNPs and *q* is the associated *q* value. *d* is the difference between two rankings of residuals (*e*_*A*_), and *n* is the number of measurements.

##### Step 2: Filtering

After applying FDR threshold of 5% at the chromosomal level for each cell type and identifying the most significant *cis*-eQTL per gene per cell type in each sex, we implemented two additional statistical assessments to filter away false positives.

1. Distributional consistency (*π*_0_): we estimated the proportion of null hypotheses (*π*_0_) for the female-only eQTLs in the male dataset (and vice versa).^39^ We removed associations where the opposite sex showed evidence of an underlying signal, ensuring we only retained associations that were truly different (threshold *π*_0_ > X).^39^
2. Effect size comparison (Z test): we used the two-sample Z test to compare the beta estimates of two populations (*β*_*female*_ vs. *β*_*male*_). For this analysis, we ran the stratified analysis using *MatrixEQTL*^40^ and generated beta estimates and standard errors for each SNP-gene pair in each cell type for females and males. Next, we calculated the z-statistics for *cis*-eQTLs identified in each sex analysis and retained only eQTLs with significant difference in magnitude (*p*_*Z*-*test*_ < 0.05). In our final step, we defined a sex-specific eQTL only if the *cis*-eQTL passed both levels of testing.

##### Step 3: Validation

We applied a SNP × sex interaction model to all candidate eQTLs that passed the filtering steps. We assessed the significance of interaction term, considering the candidates are validated if they reach an FDR threshold of <0.05.

Note that, for each of the three analyses (joint, female, and male specific), we controlled for multiple testing using the FDR and considered associations significant if their *q* values (FDR-adjusted *p* values) were ≤0.05.

#### Sex-interacting eQTLs

Next, to identify sex-differential effects among robust, “established” associations, using the joint *cis*-eQTLs results, we ran a sex-interaction analysis to identify eQTLs with varying effect sizes by sex. In this analysis, for each gene-SNP pair within each cell type, we fitted a linear regression model and tested for genotype-by-sex interaction while adjusting for previously mentioned additional factors:

Equation 3: sex-interacting cis-eQTLs

(Equation 3) $$\begin{array}{c} y=\beta_{0}+\beta_{S}.sex+\beta_{A}\;.age+\beta_{PC1}\;.PC1\;\ldots +\beta_{PC4}\;.PC4+\beta_{PF1}\;.PF1+\beta_{PF2}\;.PF2+\\ \beta_{G}.genotype+\beta_{GxS}.genotype\;.\;sex \end{array}$$

where *y* is the gene expression, *β*_0_ is the intercept, and *β* is the corresponding effect size. *β*_*GxS*_ is the effect size of genotype-by-sex interaction on gene expression. Since we have already applied a multiple-testing correction and accounted for the number of independent eQTLs tested per chromosome in our initial analysis, we applied Storey *q* value across genes to identify genes with at least one significant (FDR ≤ 0.25) sex-interacting eQTL.

We also repeated our original analysis in a sex-stratified manner, using age, first four genotype-based PCs, and two PEER factors for each sex.

$$\hat{G_{X}}=\beta_{0}+\beta_{A}\;.age+\beta_{PC1}\;.PC1\;\ldots +\beta_{PC4}\;.PC4+\beta_{PF1}\;.PF1+\beta_{PF2}\;.PF2\;e_{X_{0}}=G_{X}-\hat{G_{X}}$$

$$for\;each\;SNP\;and\;e_{X_{0}}\;pair,\varrho \;=1-\frac{6\sum d^{2}}{n\left(n^{2}-1\right)}$$

removethis(mergedwithabove)

$$\left[\begin{array}{c} \begin{array}{ccc} {SNP}_{v} & \rho_{1} & q_{1}\\ {SNP}_{w} & \rho_{2} & q_{2}\\ {SNP}_{x} & \rho_{3} & q_{3} \end{array}\\ \begin{array}{ccc} {SNP}_{y} & \rho_{4} & q_{4} \end{array}\\ \begin{array}{ccc} {SNP}_{z} & \rho_{5} & q_{5} \end{array}\\ .\\ .\\ . \end{array}\right]\begin{array}{c} q-value\to \\ ranking \end{array}\left[\begin{array}{c} \begin{array}{ccc} {SNP}_{x} & \rho_{3} & q_{3}\\ {SNP}_{y} & \rho_{4} & q_{4}\\ {SNP}_{w} & \rho_{2} & q_{2} \end{array}\\ \begin{array}{ccc} {SNP}_{z} & \rho_{5} & q_{5} \end{array}\\ \begin{array}{ccc} {SNP}_{v} & \rho_{1} & q_{1} \end{array}\\ .\\ .\\ . \end{array}\right]\begin{array}{c} top\;SNP\to \\ determined \end{array}\left[{SNP}_{x}={eSNP}_{1}\right]\rho$$

It is important to note that we applied Storey *q* value^39^ across genes to identify genes with at least one significant (FDR ≤ 0.25) sex-interacting eQTL. We made this choice given the reduced power of interacting testing and the exploratory nature of this analysis, which aims to identify broad patterns and prioritize candidate genes for future validation. Applying a stricter threshold (≤0.10 and ≤0.05) yielded a smaller number of associations, justifying a more relaxed threshold to have more meaningful biological signals.

### Imputation of the sex chromosomes

The sex chromosomes in humans share pseudoautosomal regions (PARs) and are assessed as diploid regions. PAR1 (X 60,001 to 2,699,520 and Y 10,001 to 2,649,520) and PAR2 (X 154,931,044 to 155,260,560 and Y 59,034,050 to 59,363,566) are located at the tips of the chromosomes and recombine. The remainder of each chromosome is labeled the non-PAR. In females, the non-PAR X is genotypically diploid, while in males the non-PAR Y is haploid. Thus, to analyze the sex chromosomes, we needed to impute them split by these regions. For the X chromosome, we extracted genotyped SNPs from the genotype PLINK file from the non-PAR (chrX or 23) and PAR (1 and 2) (chrXY or 25). Further filtering was performed on the PAR variants to match the HRC panel. Imputation was performed using the Michigan Imputation Server^8^ with Minimac4^9^ and the HRC panel^10^ separately for the non-PAR and PAR segments. For the Y chromosome, we extracted genotyped SNPs from the genotype PLINK file (chrY or 24). As the non-PAR Y does not undergo recombination like the non-PAR X does in females, we cannot impute genotypes on the Y. Instead, we can haplotype the Y based on their genotypes. To this end, we ran *yhalpo*^41^ to identify the broad haplogroup of the male individuals.

### Sex-chromosome eQTL analysis

We performed first sex stratified then joint eQTL analysis for PAR1, PAR2, and non-PAR regions separately. When PAR1 regions were tested, both genotypes on chromosome X and Y were modeled as 0 (homozygous for allele 1), 1 (heterozygous for allele 1), and 2 (homozygous for allele 2) and the analysis was performed as described for sex-specific eQTLs (Equation 3). Due to differences in gene locations on X and Y chromosomes, we tested an average of 4,419 SNP-gene pairs per cell type in females and 4,318 SNP-gene pairs per cell type in males. Joint sex-chromosome eQTLs were identified as depicted in Equation 1, where sex was included as a covariate when calculating the residuals before testing for Spearman’s rank correlation. To overcome the differences in available SNP-gene pairs between females and males, only pairs that were present in both sexes were included in this analysis. We conducted the eQTL analysis for PAR2 region in the same manner as PAR1. A similar approach was followed when analyses were performed for the non-PAR region with the difference being that, when testing for males, genotypes on chromosome X were modeled as 0 (homozygous for allele 1) and 1 (homozygous for allele 2) and analysis was completed using non-parametric Mann-Whitney U-test.

## Results

### Single-cell data reveal cell-type proportion differences

**Figure 2:**
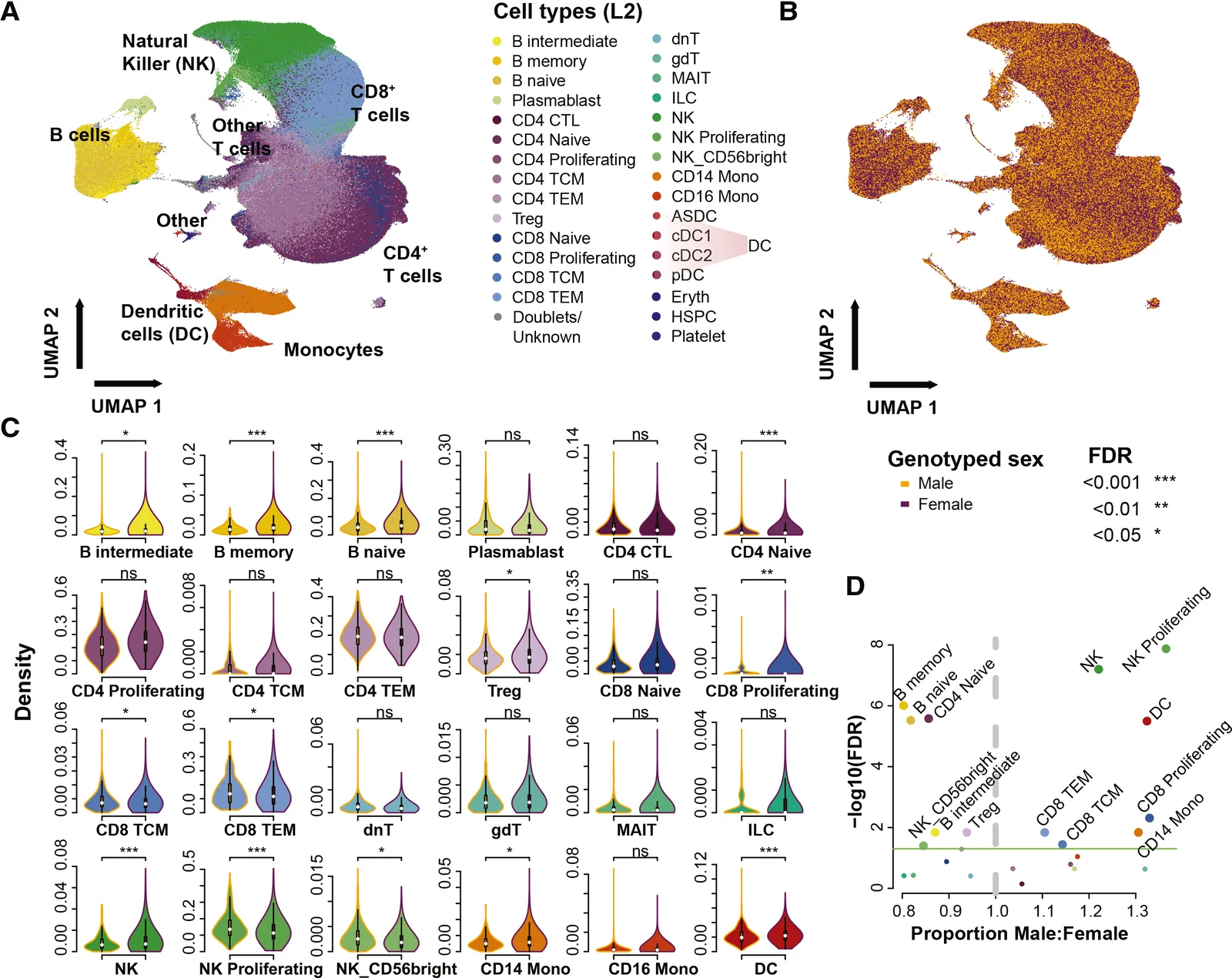
Distributions of cell-type proportions across sex (A) UMAP of all 1,267,758 cells, colored by cell type. Dendritic cell labels (ASDC, cDC1, cDC2, and pDC) were combined for all downstream work. (B) UMAP of cells colored by sex: males in gold, females in purple. (C) Density plots showing the distribution of proportions of each cell type. Females are shown as a density plot on the left, and males on the right. Significance of differences based on an FDR from an F test (proportions) are indicated by asterisks (^∗^FDR < 0.05, ^∗∗^FDR < 0.01, ^∗∗∗^FDR < 0.001). (D) Male to female ratios versus the FDR.

We classified ∼1.25 million cells from 982 individuals from the OneK1K study^7^ (565 females, 418 males) into 30 transcriptionally distinct cell types using the Azimuth classification tool^13^ (Figures 2A and 2B). Calculating proportions of each cell type per individual, we observed clear compositional differences between the sexes (Figures 2C and 2D; Table S1). In males, we found higher proportions of CD14^+^ monocytes, dendritic cells (DCs), NK cells, NK proliferating cells, CD8^+^ proliferating cells, T-effector memory (TEM) cells, and T-central memory (TCM) cells. In females, we found significantly higher proportions of B cells, CD4^+^ naive T cells, NK CD56^+^, and regulatory T cells (Tregs). Most of these differences have been reported in the literature,^3^^,^^42^^,^^43^^,^^44^^,^^45^ yet some were not previously reported, including Tregs and DCs.

In the innate immune system, CD14^+^ monocytes were present at higher proportions in males (3.71% in males versus 2.84% in females; FDR = 0.014). Higher proportions of monocytes have been reported in male infants^46^ and some ethnicities,^47^ but it is not a well-established phenomenon between the sexes.^48^^,^^49^ No proportional difference was present in CD16^+^ monocytes, consistent with others’ observations.^48^ Due to low overall counts, we combined the DC subtypes plasmacytoid (pDC), conventional cell 1 (cDC1), conventional cell 2 (cDC2), and *AXL*+ DC (aDC) into a broad dendritic cell category. We observed a strong relationship between sex and DC proportion, with higher percentages in males (0.64% in males vs. 0.48% in females; FDR < 0.001). This has not been previously observed in flow-cytometric studies of immune variation.

In the adaptive immune compartment, we found higher proportions of Tregs in our female samples (2.18% in males vs. 2.32% in females; FDR = 0.0145), whereas the opposite or no difference was typically reported.^44^^,^^50^ Tregs vary during the female menstrual cycle in response to estrogen^51^ and other sex steroids, so these results are potentially confounded with sex hormone levels. As we did not have associated hormone-related information, we used age as a proxy to test our hypothesis. As an in-depth analysis with age is beyond the scope of this work,^52^ we performed a correlation analysis (Figure S1; Table S2) and identified no significant correlation between Tregs and age.^53^ To adjust for confounders and consider statistical assumptions, we tested potential alternative models and ran additional diagnostic checks. These included the incorporation of age and ethnicity as covariates as well as the application of alternative modeling frameworks, specifically a non-parametric Kruskal-Wallis test and a beta-regression model (see methods). We observed no changes to the results (Table S3).

### Sex-differential expression by cell type reveals sex biases obscured in bulk analyses

**Figure 3:**
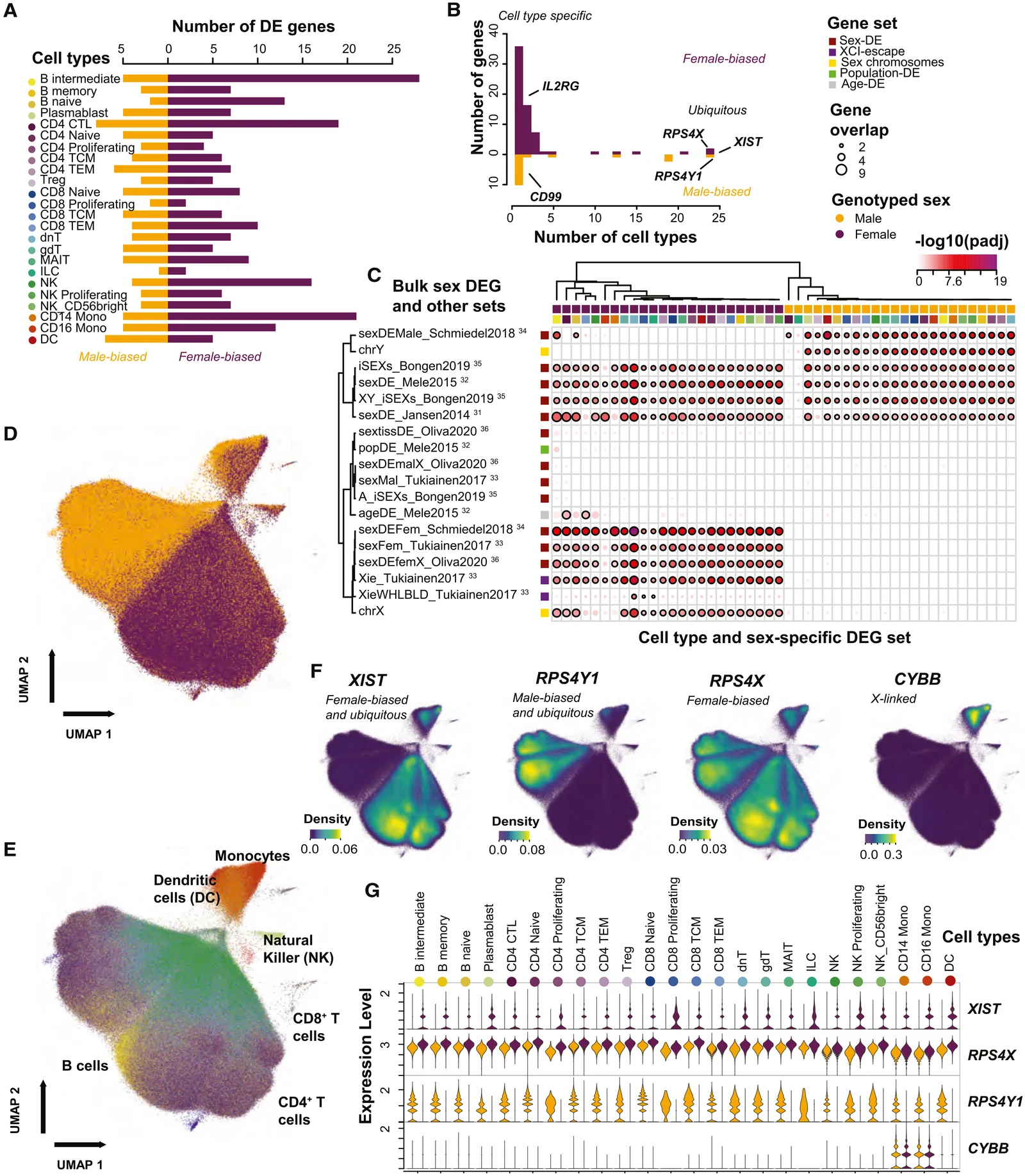
Sex-differential expression (A) Number of sex-DEGs using the Wilcoxon test per cell type (two sided). (B) Recurrence of these DEGs shows mostly unique genes, but sex-specific markers were recurrent across all cell types (ubiquitous). (C) Gene set enrichment analysis of DEGs showing enrichment of sex-specific gene sets. Additionally, age-related enrichment in DEGs of CD8 TEMs and CD4 CTLs and population-related genes in B intermediate cells. (D) Exploiting variation of the genes on the XY chromosomes to visualize sex through a UMAP. (E) Then colored by cell types, showing that some cell-type markers are on the sex chromosomes. (F and G) (F) UMAPs and (G) violin plots of genes of interest. Cell-type-specific markers (*XIST* and *RPS4Y1*) show clear sex differences. Expression of X-linked genes *CYBB*, with no sex bias, and *RPS4X*, which is differentially expressed.

To measure differences in response and activity across the transcriptome, we performed a differential expression analysis between the sexes to identify sex-biased gene expression for each cell type. We detected between three and 33 sex-DEGs per cell type prior to multiple test correction. Adjusting for confounding factors (age and ethnicity) retained the same DEGs (MAST^18^), with the exception that *NKG7* (MIM: 606008) was no longer significant in gdT. To model the effects across cell types, given sample dependence and potentially correlations, we applied MASH.^19^ Following this analysis, we detected 78 DEGs across 24 cell types: 16 genes with male-biased expression and 65 genes with female-biased expression (Figures 3A and 3B; Tables S4, S5, S6, and S7), and three showing both female and male-biased expression in different cell types (*CD79A* [MIM: 112205]), *TSC22D3* [MIM: 300506], and *JUN* [MIM: 165160]). These genes showed female-biased expression in B cells but were more highly expressed in male dendritic cells. Of the three, TSC22 Domain Family Member 3 (*TSC22D3*), also known as glucocorticoid (GC)-induced leucine zipper (*GILZ*), is a known sex-biased and X-linked gene, while the others were not previously reported. Important to note that, of the 78 genes, only 16 of these genes were on the sex chromosomes (11 on the X and five on the Y), implying additional sex-specific regulation of autosomal genes. For further sensitivity analysis, see the supplemental notes.

We tested for overlap between our results and known sex-DEGs from bulk RNA-seq studies and X-linked gene sets of interest^31^^,^^32^^,^^33^^,^^34^^,^^35^^,^^36^ (Figure 3C; see methods). Of the 78 genes, 42 have been identified as sex-DEGs in bulk studies of whole blood or other tissues. We also noted consistent sex-biased expression profiles. That is, if it is upregulated in females (female biased), this expression pattern is maintained at the cellular level (Table S5). The exceptions include *FLNA* (filamin A [MIM: 300017]) and *SAT1* (Spermidine [MIM: 313020]). These are male-biased sex-DEGs in bulk, but both appear female biased in our data. This inversion of biased expression could also be due to bulk analyses masking expression or could be linked to variation in X escape of these genes in different tissues and cell types. Of the remaining 36 genes, around 17 were detected in one cell type, suggesting that there are signals at single-cell resolution results obscured in bulk.

### Immune pathways are enriched in sex-biased genes

To quantify the overlap between sex-biased genes and their functions, we tested for gene set enrichment of pathways and gene groups (Figure S2). The female-biased sex-DEGs in B intermediate, B naive, CD14^+^ monocytes, CD8^+^ TEM, and NK cells were enriched for the tumor necrosis factor alpha (TNF-α) signaling pathway (adjusted *p*: B intermediate ∼2.04 × 10^−9^, B memory ∼6.74 × 10^−3^, CD14^+^ Mono ∼2.81 × 10^−2^, CD8^+^ TEM ∼2.94 × 10^−3^, and NK ∼3.41 × 10^−4^), which included genes regulated by nuclear factor kappa-light-chain-enhancer of activated B cells (NF-κB) in response to TNF-α expression. TNF-α is a cytokine used by the immune system for cell signaling, and dysregulation of NF-κB has been linked to inflammatory and autoimmune diseases.^54^ The genes driving this enrichment were similar for most cell types and included *JUN*, *DUSP1* (MIM: 600714), *DUSP2* (MIM: 603068), *IER2* (MIM: 620036), *ZFP36* (MIM: 190700), *CD69* (MIM: 107273), *CD83* (MIM: 604534), *SAT1*, *KLF6* (MIM: 602053), and *PPP1R15A* (MIM: 611048). Many of these genes encode proteins involved in the hypoxia pathway that was significantly enriched in B cells (*p*-adjusted: B intermediate ∼9.72 × 10^−5^, B naive ∼5.73 × 10^−3^). Furthermore, these genes also encoded proteins involved in cellular proliferation, specifically of T cells.^55^ Interestingly, the CD14^+^ monocytes had a distinct set of genes driving the TNF-α enrichment, including *G0S2* (MIM: 614447), *NFKBIA* (MIM: 164008), and *PLAUR* (MIM: 173391). The proteins encoded by these genes were also involved in the inflammatory response (CD14^+^ monocytes *p*-adjusted ∼8.88 × 10^−4^), along with *CD14* (MIM: 158120) and *EMP3* (MIM: 602335), both linked to monocyte differentiation/proliferation. In CD4^+^ CTLs, the interferon-gamma response (*p*-adjusted∼3.77 × 10^−9^) and allograft rejection (*p*-adjusted∼2.20 × 10^−2^) were enriched in female-biased sex-DEGs. Some genes were shared between these two pathways, including *CD2* (MIM: 186990), *GZMA* (MIM: 140050), *HLA-A* (MIM: 142800), *HLA-E* (MIM: 143010), *IL2RG* (MIM: 308380), *FLNA*, and *CCL5* (MIM: 187011). The sex specificity of all these genes is unclear as their proteins have broad functions; however, this could be due to the higher activity of these pathways in females. Monocytes are reported to have increased functional activity in females, summarized as primed interferon (IFN)/immune pathways and overexpression of immune genes at basal levels.^56^ Male-biased sex-DEGs were enriched for ribosomal-related functions and pathways. These include rRNA processing, translation, and metabolism of RNA pathways (Figure S6B).

One mechanism for sex differences in gene expression is believed to occur through gene regulation by sex hormones and their receptors in immune cells.^57^ To evaluate this hypothesis, we tested for enrichment of sex hormone receptor target gene sets (estrogen receptors *ESR1* [MIM: 133430], *ESR2* [MIM: 601663], and androgen receptor *AR* [MIM: 313700]) from MotifMap.^58^ We found no enrichment of the receptor target genes in the DEGs (Figure S2F). Instead, genes with female-biased expression in CD14^+^ monocytes were enriched for the *ESR1* TF targets gene set from ENCODE (*G0S2*, *ITGB2* [MIM: 600065], *MALAT1* [MIM: 607924], *MT2A* [MIM: 156360], *MYL6* [MIM: 609931], *NFKBIA*, *S100A10* [MIM: 114085], and *S100A11* [MIM: 603114], *p*-adjusted ∼5.42 × 10^−4^). Additionally, *LGALS2* (MIM: 150571) (sex-DE in CD14^+^ monocytes) was a target of *ESR2* (ER*β*). Monocyte cell counts decrease as estrogen levels increase through the Fas/FasL system and the ER*β* receptor (*ESR2*).^59^ Thus, our analysis highlights the role of estrogen in differing monocyte cell counts and their functional activity.

### Genes responsible for establishing immune cell-type identity are mostly distinct from those determining sex identity

Cell-type transcriptional identity is defined by the expression levels of marker genes, some of which are located on the sex chromosomes. These genes are not merely markers but also potentially functional elements. We examined the intersection between the sex-chromosome genes, sex-DEGs (including autosomal), and known cell-type marker genes to assess their impact on cellular and sex-linked identity. We first evaluated the variation of sex-chromosome genes within a dimension reduction framework (UMAP), which allowed us to visualize the influence of these genes on cell identity, agnostic of their sex-biased expression profiles. Using the variation of genes expressed on the X and Y chromosomes, we observed a clustering by sex (Figure 3D) and a strong clustering by the myeloid and lymphoid lineages, with monocytes and dendritic cells split from the remaining cell types (Figure 3E). This separation indicates an important role for a subset of sex-chromosome genes in monocyte and dendritic cell identity with between 10 and 16 marker genes located on the sex chromosomes (examples in Figures 3F and 3G). However, this overlap was not significantly enriched but was strong enough to drive the clustering. We do note, although the clustering is quite tight, that this may be due to artificial structures generated from the dimensionality-reduction approach.

To further explore this finding and focus on a potential set of sexually dimorphic and immune-related genes, we tested for overlap of the sex-biased genes (sex-DEGs) and cell-type marker genes. For this, we utilized the L2 cell-type classification marker genes from the Azimuth reference panel. Overall, 20 of the 215 cell-type marker genes (Table S23) were sex biased. When we looked at the cell-type-specific distribution of these 20 genes, we found 14 of them were both cell-type markers and sex-DEGs within the same cell type (Table S5). These included *CD79A* (MIM: 112205) in B intermediate and naive cells; *CXCR4* (MIM: 162643) in B naive cells; *CD14*, *G0S2*, and *S100A8* (MIM: 123885) in CD14^+^ monocytes; *FCGR3A* (MIM: 146740) in CD16^+^ monocytes; *FGFBP2* (MIM: 607713), *GZMA*, and *GZMH* (MIM: 116831) in CD4 CTLs; *GZMK* (MIM: 600784) in CD4 TEM, CD8 TEM, and MAIT cells; *KLRC1* (MIM: 161555) in gDT; *NKG7* in MAIT; and finally *FCER1G* (MIM: 147139) in NK cells. Of these, none were on the sex chromosomes.

The 49 autosomal sex-biased marker genes could play crucial roles in cell function and in sexual dimorphism of immune traits. For example, the autosomal gene *CCL5*, which is a marker for CD8^+^ T cells, shows cell-type-specific expression and is differentially expressed by sex, with higher expression in females in CD8^+^ TCM cells (log2FC = −0.22, FDR ≈ 2.02 × 10^−22^). Increased expression of *CCL5* is potentially linked to improved antiviral immunity through lymph node and splenic homing of viral-specific CD8 T cells.

### Sex-specific *cis*-eQTLs at the single-cell level are mostly cell-type specific

**Figure 4:**
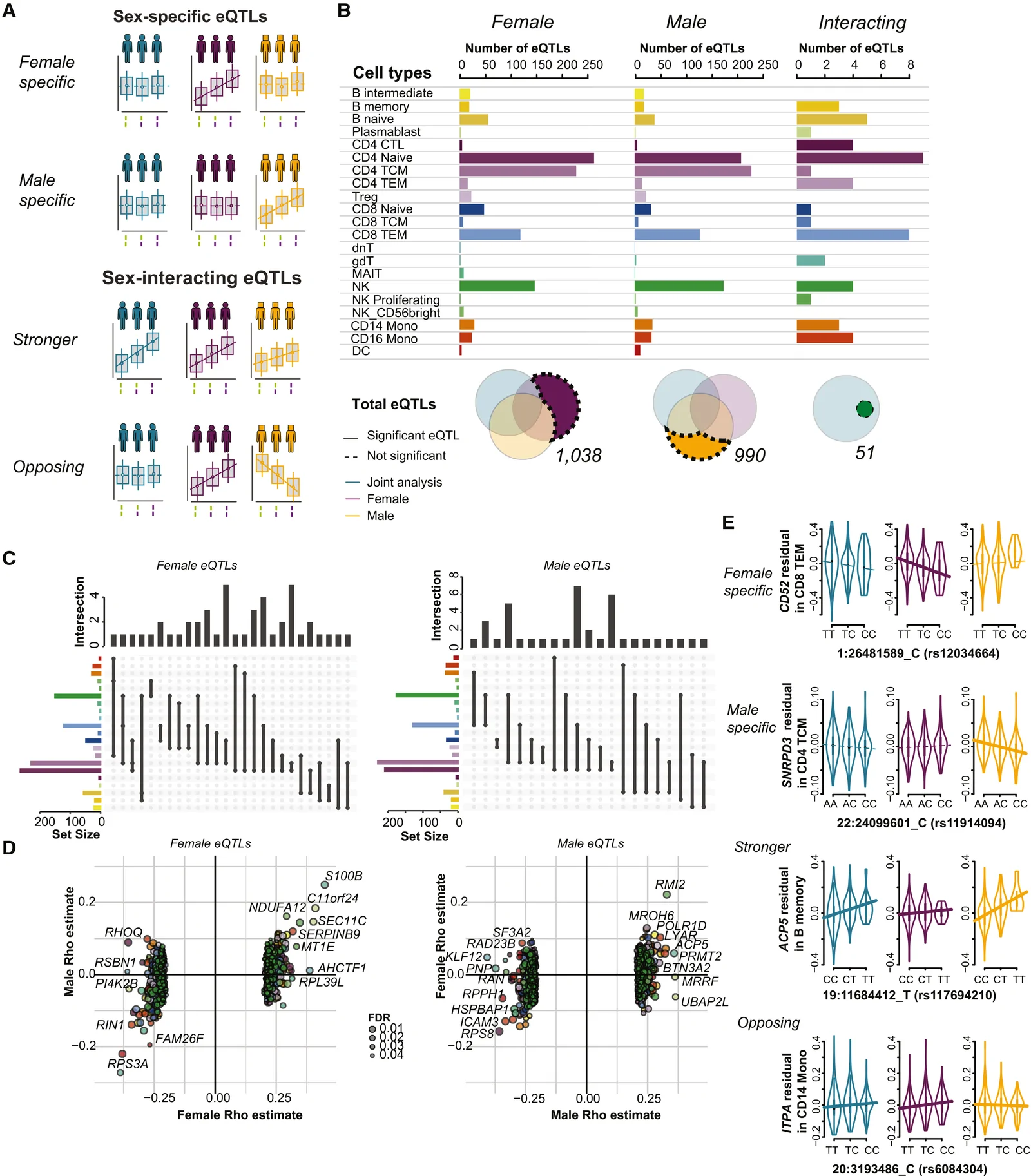
Sex-linked *cis*-eQTLs and sex-interacting eQTLs (A) Sex-biased eQTLs can either be sex specific or sex interacting. Sex-specific eQTLs can be female or male, without significant association in the joint analysis or in the opposite sex. Sex-interacting eQTLs are significant in all analyses but have different effect sizes. Joint analysis in the model is colored turquoise, with males in gold and females in purple. (B) Total number of *cis*-eQTLs per cell type across the joint, stratified, and interacting eQTLs. (C) Overlapping *cis*-eQTLs across cell types for female-specific (left) and male-specific (right) eQTLs. (D) Scatterplot of effect sizes (rho estimates) for female (left) and male (right) specific eQTLs plotted against estimate in the opposite sex. Colored by cell type, and size of point reflects sex-specific FDR. (E) Example eQTLs for all four sex-biased eQTLs from (A).

**Figure 5:**
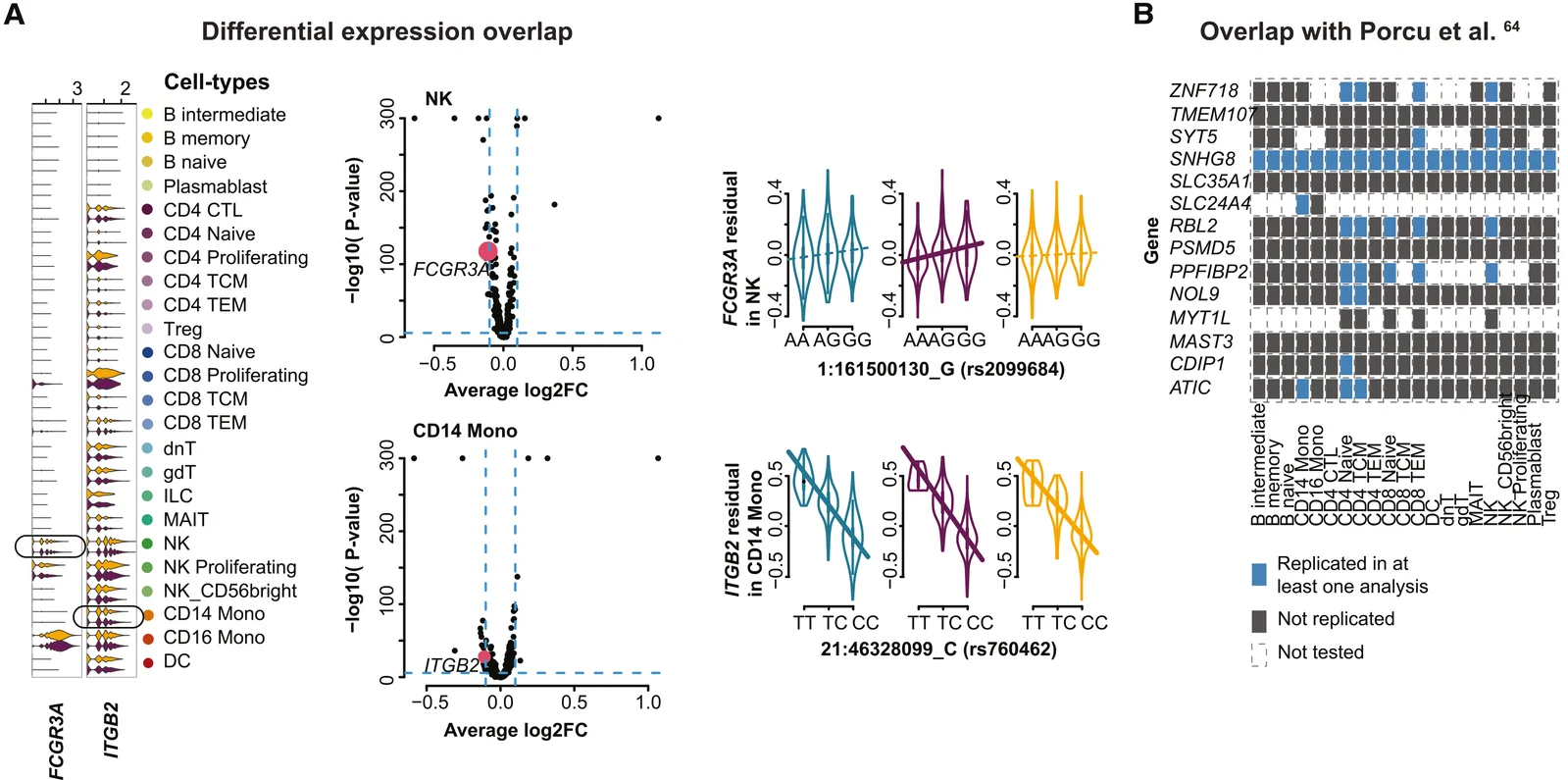
Overlap and replication of *cis*-eQTLs (A) Overlap of sex-biased eQTLs with a sex-biased expression showing eQTL plot, differential expression, and expression of genes *FCGR3A* (female specific) and *ITGB2* (interacting). (B) Replication of sex-interacting SNPs from Porcu et al.^64^ in our data per cell type. Blue indicates replication, while gray boxes describe no replication. Dashed were not tested.

Sexually dimorphic phenotypes may partly derive from genetic effects and their interactions with the environment. Several eQTLs have been identified to show sex-biased or sex-interacting effects using bulk RNA-seq^36^^,^^60^^,^^61^ and more recently using single-cell sequencing of lymphoblastoid cell lines^62^ and PBMCs from the Asian Immune Diversity Atlas.^63^ To understand the impact of sex on genetic control of gene expression at the single-cell level at the population scale, we tested for *cis*-eQTLs on the autosomes (Figure 4) and sex chromosomes (Figure 5). For autosomes, we first performed a joint analysis of both sexes to identify robust shared eQTLs. We found 14,432 autosomal eQTLs across 21 cell types in our joint analysis (Figure 4B; Table S10). Next, we conducted a sex-stratified discovery analysis in downsampled groups to identify sex-specific eQTLs. The candidate eQTLs from this analysis were then filtered using a two-sample Z test and *π*_0_ estimation and subsequently validated through a genotype-by-sex model (Figure 4A; Table S9). In our stratified analyses, we found fewer eQTLs overall, likely due to reduced power from stratification and stringent filtering, with 1,038 eQTLs in females and 990 in males (Figure 4B; Tables S11 and S12). Between cell types, many significant eQTLs were also unique (i.e., cell-type specific; Figure 4C) (global *q* <0.05); however, these discoveries are based on the number of available cells for that cell type.^7^ A total of 951 eGenes were observed in males, with 39 (4%) occurring in more than two cell types. In females, of the total of 989 eGenes, 41 were in two cell types, and four genes were in three (4.6% > 1). Additionally, 14 female-specific eGenes were in the MHC region, compared to 16 male-specific eGenes. Although the Spearman’s *p* estimates for sex-specific eQTLs were modest (|Rho| ≈ 0.3), these associations were significant, reflecting consistent but weaker genotype-expression correlations relative to joint eQTLs (Figure 4D). This reflects low but significant effects of SNPs on sex-specific eGenes. While the SNPs differed, 122 eGenes were common to both male and female associations. The majority of these eGenes (107) showed significance in distinct cell types, indicating that a small subset of genes may have a sex-specific and cell-type-specific regulatory mechanism. The 15 eGenes in the same cell types had variants that were not in linkage disequilibrium (LD), suggesting these eGenes were also under different genetic regulatory control.

We next tested for functional enrichment of the eGenes with sex-specific associations. We identified enrichment of transcription factor targets and immune-related functions using MSigDB.^25^ Genes with associations in either females or males are likely to have sex-specific functions, which are not captured by the enrichment. For example, the female-specific eQTL of *CD52* (MIM: 114280) rs12034664 in CD8 TEMs (Figure 4E) may be linked to downstream activity: the CD52 glycoprotein functions as an effector molecule in suppressing Tregs in type 1 diabetes (MIM: 222100).^65^ Interestingly, a second variant in DC (rs11577318) is a male-specific eQTL. These SNPs are not in LD (rs11577318 and rs12034664, R^2^ = 0.0087) and thus exhibit independent control of the same gene in different cell types. Another eGene of interest is *SNRPD3* (MIM: 601062) with a male-specific eQTL rs11914094 in CD4 TCMs (Figure 4E). *SNRPD3* encodes a subunit of the spliceosome in humans, but, in mice, this gene and associated complex have increased expression in male mice, believed to be linked to sex determination through alternative splicing.^66^

### Sex-specific allele effects drive gene-expression differences

The previous stratified analysis focused on *cis*-eQTLs that were absent in the other sex, yet there may be different effect sizes of the same variant, which we call sex interacting. To identify sex-interacting eSNPs, we took eQTLs from the joint set and tested for a genotype-by-sex interaction (see methods). This analysis highlights expression differences that occur when the allelic effect of genotype differs between males and females.^61^ We identified 51 sex-interacting eQTLs (Table S13). The majority (46 eSNPs) were in the same allelic direction in both sexes but with a noticeable difference in magnitude; specifically, 27 showed a stronger effect in males and 19 in females. While we used a discovery threshold of FDR ≤ 0.25, we found a core set of 12 and two associations remain significant at the more stringent FDR <0.10 and <0.05 levels, respectively (Table S13).

Our approach also detected effects present in one sex or in the opposite direction of the allelic effect (45 eGenes). These include *NLRP2* (MIM: 609364) (*p* = 3.18 × 10^−3^, FDR = 0.17), *ITPA* (MIM: 147520) (*p* = 5.98 × 10^−3^, FDR = 0.19), *IL2RA* (MIM: 147730) (*p* = 2.11 × 10^−9^, FDR = 0.079), and *NAGK* (MIM: 606828) (*p* = 4.52 × 10^−3^, FDR = 0.19). *NLRP2* (NACHT, LRR, and PYD domains-containing protein 2, rs12969457 B naive) is a sensing component of NLRP2 inflammasomes and contributes to the regulation of immune responses regulating activities of NK-κB and acting as a pro-inflammatory molecule through caspase-1 activation.^67^ Furthermore, NLRP2 also has reproductive functions, believed to be responsible for maintaining fertility in females^68^ and establishing maternal-fetal tolerance during pregnancy.^69^ *ITPA* (inosine triphosphate pyrophosphatase ITPase, rs6084304 CD14^+^ monocytes) variants have been linked to chronic hepatitis C response treatment efficacy.^70^ For *IL2RA* (interleukin-2 receptor subunit alpha, rs7261003, CD8 TEM), the interleukin-2 receptor is involved in regulating immune tolerance by controlling the activity of Tregs.^71^

### Sex-biased gene expression in SLE-associated genes is influenced by genetic regulation

To link gene expression that is biased between sexes to genetic regulation, we examined the overlap between sex-biased eQTLs and the sex-biased DEGs. We identified most of the overlap signals occurring in eQTLs with joint effects rather than those with sex-specific effects (19 genes). However, we found one gene with a female-specific eQTL and sex-biased expression: *FCGR3A* in NK cells. This gene forms part of the immunoglobulin gamma (IgG) receptor and mediates IgG effector function in NK cells. It has also been associated with sex-linked traits: immunodeficiency of NK cells,^72^ including susceptibility to recurrent viral infections^73^; severity of COVID-19^74^; and the autoimmune disease SLE.^75^ More specifically, the rs2099684 variant is associated with Takayasu arteritis (MIM: 207600) in the Han Chinese population, which has a 90% female bias.^76^ Other variants (rs396991) on *FCGR3A* are shown to affect the efficacy of antibody-dependent NK cell-mediated cytotoxicity in patients receiving rituximab treatment.^44^ Additionally, we find a *ITGB2* sex-interacting eQTL in CD14^+^ monocytes, which also shows female-biased expression in our data (Figure 5A). *ITGB2* (Integrin beta chain-2), along with the alpha subunit, encode integrin heterodimers involved in cell adhesion and cell-surface mediated signaling. It is linked to the inflammatory response in monocytes,^77^ along with roles in the autoimmune disease systemic sclerosis (scleroderma (MIM: 181750)).^78^ More recently the *ITGB2* signaling pathway was shown to be enriched both in SLE and primary Sjogren’s syndrome (MIM: 270150).^79^ However, the specific variant (rs760462) has no known clinical correlation. Nevertheless, these two genes are also located in two distinct *cis*-regulatory elements and are interesting examples of sex-specific genetic regulation.

To verify and replicate our sex-specific eQTLs on autosomes, we looked for overlap with other bulk studies available.^36^^,^^61^^,^^64^^,^^80^ In these studies, little replication was evident across them. Of the 26 genes identified overall, we successfully replicated 10 eGenes/eQTLs across various cell types using different analyses (Figure 5B). Once again, this analysis suggests that single-cell resolution can detect associations missed in bulk. Lastly, we conducted a gene co-expression network analysis (see supplemental notes and methods), considering cell type and sex specificity, to identify additional genes sharing common regulatory control or pathway involvement (Figures S7–S10; Table S22). However, the generated networks did not overlap with modules for either *FCGR3A* or *ITGB2*.

### *cis*-eQTLs are depleted on the sex chromosomes

**Figure 6:**
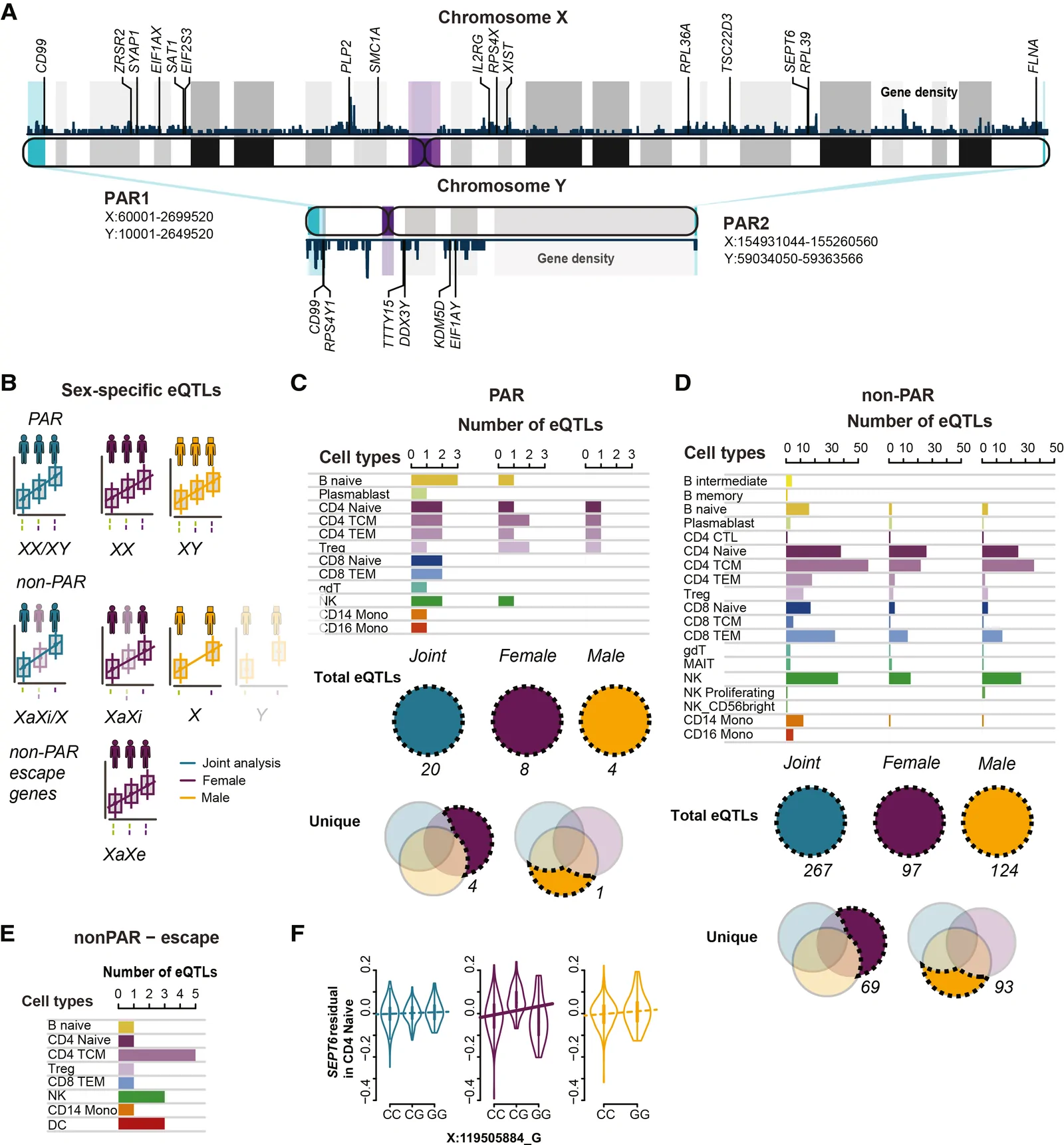
Sex-chromosome eQTL analysis (A) Ideograms of the X and Y chromosomes showing PAR and non-PAR regions (cyan) and centromere (purple). Gene-density (navy) histogram across the chromosomes, along with highlighted genes of interest on the X and Y. (B) Sex-specific eQTL models of the sex chromosomes split into the PAR, non-PAR, and X escapee genes. Each model highlights the joint analysis (turquoise) and the sex stratified (females in purple and males in gold). PAR analysis is similar to the autosomes, where genes escape X-inactivation. In contrast, heterozygous alleles in females in the non-PAR analysis require knowledge of the inactive/active allele (light purple) as males are only ever homozygous. (C–E) Total number of *cis*-eQTLs by cell type in the (C) PAR regions, (D) non-PAR regions, and (E) non-PAR escape genes. (F) Example gene *SEPT6* with a female-specific eQTL and female-biased DEG.

The effects of genetic variants on the sex chromosomes have not been thoroughly assessed in many eQTL studies, and this may explain some of the observed sex differences. To assess the sex chromosomes for eQTLs, we separated our analysis of the X into PAR and non-PAR and then further into X-escape and non-escape genes (Figure 6A; Table S17). The non-PAR regions of the X chromosome are haploid, as females inactivate one of their X chromosomes, and males only have one copy of the X. This region spans most of the X chromosome, approximately 152 Mbp. Around 953 genes are located in this region (GENCODE hg19/GRCh37), with 41 immune-related genes (∼4%) and approximately 66 genes escaping X-inactivation.^33^ The pseudoautosomal regions (PAR1 and PAR2) are short regions of homology between the X and Y chromosomes at the tips of both chromosomes. The PARs are thus diploid, as genes on the female inactive X escape inactivation. In total, 26 protein-coding and lncRNAs sit in these PARs.

For the PAR analysis, we genotyped approximately 1,300 SNPs on the PAR XY, with around 300 passing QC. As with the autosomes, we ran our analyses both jointly and stratified (Figure 6B; Table S18). In the joint analysis, we identified 14 eQTLs in PAR1 and six in PAR2, totaling of 20 eQTLs (nine eGenes). In the stratified analysis, we found eight eQTLs in females and five in males, all in PAR1. We identified no significant associations in PAR2, which may be due to a loss of power. All eGenes detected in the stratified analysis were detected in the joint analysis (Figure 6C) except *AKAP17A* (MIM: 312095), a protein kinase A anchoring protein, found in female Tregs (FDR ≈ 0.0107),^81^ and *IL3RA* (MIM: 308385) in females in NK cells (FDR ≈ 0.01). The latter encodes CD123 (interleukin 3 receptor alpha) and has been shown to influence COVID-19 responses between the sexes.^82^

**Figure 7:**
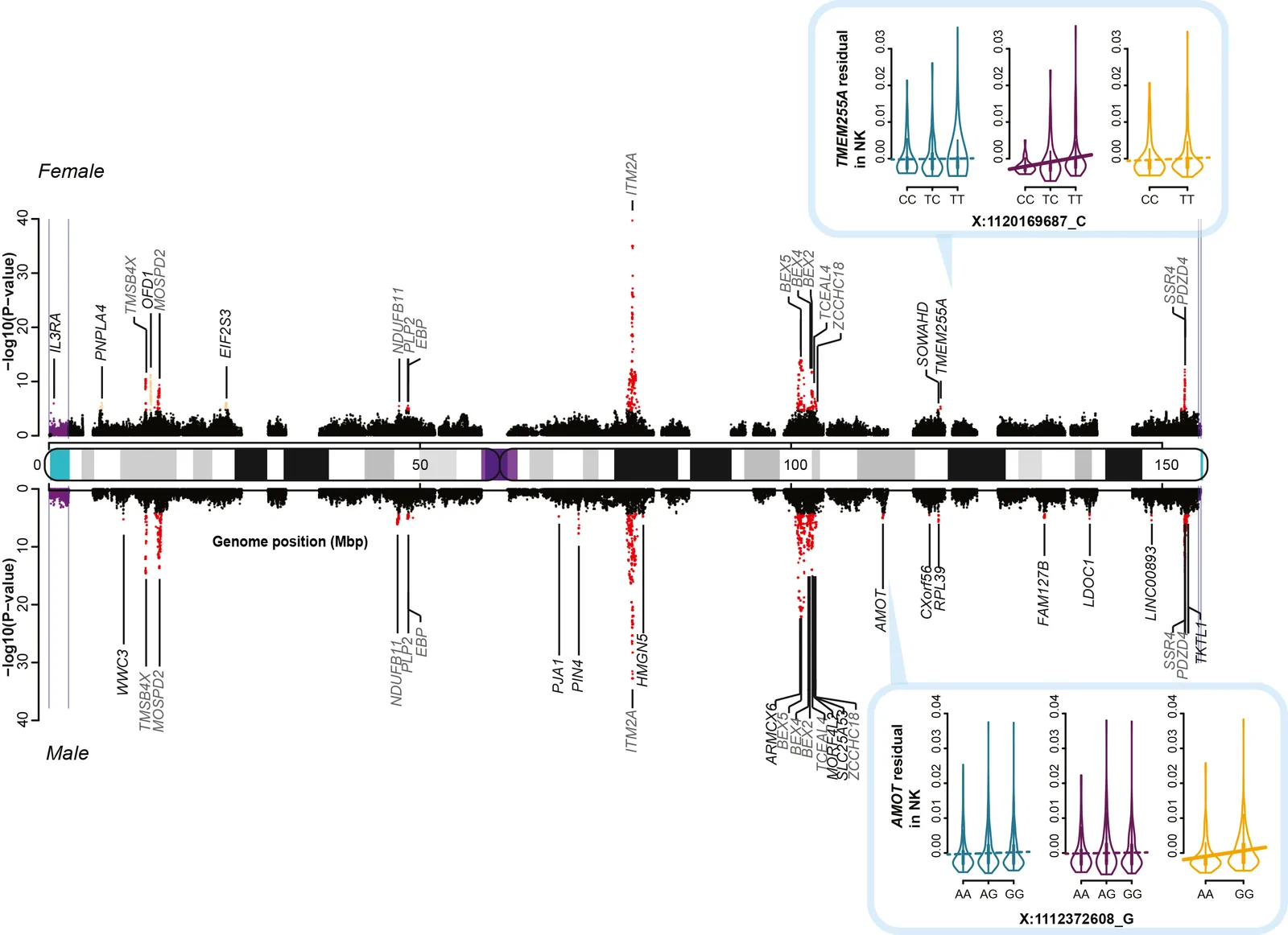
Example Manhattan plots of sex-chromosome eQTLs for NK cells Top female, bottom males, with female specific (*TMEM255A*) and male specific (*AMOT*) as examples. The *x* axis is the chromosome position, while the *y* axis shows the −log10(*p* value) at that position.

In the non-PAR joint analysis, we found fewer eQTLs on the X chromosome relative to autosomes (Figure 6D; Table S19). We tested genes that do not escape X-inactivation by removing known X-escaping genes from our list (66 genes^33^; Table S14). This was done to compare similar gene dosages across males and females. We identified 97 significant eQTLs in the female-stratified analysis. In males, we detected 124 eQTLs on the non-PAR X chromosome. The additional results in the male analysis were likely due to variation in XCI for heterozygous females. We jointly repeated the analysis and identified 267 eQTLs. Of the 97 eQTLs identified in females, 69 were unique (i.e., not in the joint and male analysis), and, of the 124 in males, 93 were unique. Finally, we examined genes that are known to escape X-inactivation in females. In this analysis, we found 16 eQTLs, with *XIST* and *RPS4X* (MIM: 312760) being the most recurrent across cell types (Figure 6E). Many sex-biased eQTLs that were in the same eGenes had differing lead SNPs. For example, in NK cells (Figure 7), 13 eGenes had all male-specific associations (e.g., *AMOT* [MIM: 300410]). At the same time, three were female-specific (e.g., *TMEM255A*) and three X-escape genes (*PNPLA4* [MIM: 300102], *OFD1* [MIM: 300170], and *EIF2S3* [MIM: 300161]) were not tested in males.

In addition to the X non-PAR, the Y chromosome has a non-PAR spanning 56 Mbp and containing 102 genes. In our data, we genotyped ∼7,000 SNPs, of which 3,243 remained after QC. As no recombination occurs on the Y, imputation here was difficult and unlikely to be informative. We attempted to use the haplogroups of the Y chromosomes^41^; however, all the males were classified as belonging to major haplogroups BT-M8947 and BT-M8949, which differ by one variant each and thus there is not enough variation to test for *cis*-eQTLs.

In cases where genes are differentially expressed by sex on the sex chromosomes, this may be due to genetic control. To test this, we again looked for an overlap between the eQTLs and sex-DEGs (Tables S20 and S21). Among the PAR X chromosome genes, we observed no overlap between eGenes and sex-DEGs. In the non-PAR X, *SEPT6* (MIM: 300683) was an eGene with both female-biased expression and an eQTL in CD4^+^ naive and CD4^+^ TCMs (Figure 6F). *SEPT6*, a septin GTPase, plays a role in T cell migration^83^ and may potentially escape XCI.^84^ Of the escape genes, *RPS4X*, *XIST*, and *EIF2S3* had both eQTLs and female-biased expression in a few of the cell types (B naive, CD4^+^ naive, CD4^+^ TCM, DC, and Treg). As in the autosomal analysis, no male-specific eQTLs overlapped male-biased expression. Together, these results indicate higher immune gene expression in females at baseline, reflected primarily in more female-biased expression but also alternate regulatory control, likely linked to X-inactivation and escape.

## Discussion

Our analysis of sex differences in PBMCs highlights the importance of studying the immune system in a sex-specific manner. Despite known differences in the immune systems of males and females being recorded, many researchers still study and assess their functions, pathways, gene expression, and genetic regulation agnostic of sex. This work examined these differences in a dataset of close to 1,000 individuals and found that small differences in cell-type proportions do exist between the sexes. These differences are likely to underpin functional effects, such as heightened responses to foreign stimuli (e.g., monocytes) and autoimmune reactivity (e.g., T and B cells).

In addition to differences in the cellular landscape between male and female individuals, we also identified sex-specific differences in gene expression. Expectedly, many of these genes were on the sex chromosomes. However, the differentially expressed functions of these genes remain open to investigation. Ribosomal genes that exhibit male-biased expression may be linked to differences in proliferation, cell activation, and cellular exhaustion, common mechanisms in cancer. In contrast, the number of genes that showed sex-biased expression in females was linked to immune pathways, highlighting downstream activity likely to be influenced in activated or stimulated immune systems.

Through a comprehensive evaluation of the effect of sex and the X chromosome on control of gene expression in a cell-type-specific manner, we identified numerous sex-specific eQTLs that were not previously observed in bulk whole-blood studies. The impact of genetic variation on gene expression on the X chromosome differed from that on autosomes, with fewer eQTLs overall and lower effect sizes. Moreover, the X-chromosome eQTLs were less likely to be shared between cell types. These findings align with previous studies and support the hypothesis that a more efficient purifying selection on the X is present compared to autosomes.^61^ When considering the functional enrichment of sex-specific DEGs and eQTLs, we found that male-specific DEGs and eGenes were related to non-reproductive-system cancers (e.g., lung). In contrast, female-specific ones were generally involved in immunological pathways.

However, our findings have their limitations. In addition to differences in sex, several other factors can influence gene expression. For example, immune responses change over a lifetime; as we age, B cells increase in females, while Tregs increase in males. Furthermore, there is considerable evidence that parity^85^^,^^86^ and hormonal changes with menopause impact gene-expression levels in mammary glands, which can change the risk of breast cancer. These changes may also have affected circulating immune cells and should therefore be considered in future studies. A further caveat to this analysis is that none of these cells were stimulated or activated by any infectious trigger, and the effect sizes we observe reflect baseline differences. In other conditions, these small differences may be exacerbated. Additionally, because we have collected our data at a single time point, any fluctuations in hormone levels or dynamic/periodic changes in gene expression, such as circadian rhythms, will be missed or averaged out. Future work to assess the influence of infections, stress, or other environmental factors may show additional or larger effect sizes.

There is a well observed disparity in disease prevalence between sexes with autoimmune diseases being more common in females.^1^ Overall, our results suggest that genes with sex differences are involved in immunologically important functions, showing higher overall activity in females. These results highlight that genes that are sexually dimorphic at baseline could potentially vary in their response in immune disease between the sexes in a cell-type-specific way.

## Data and code availability

The datasets and code generated during this study are available on Zenodo: https://doi.org/10.5281/zenodo.19210998 (https://zenodo.org/records/19210998) or on GitHub: https://github.com/ballouzlab/sex_diffs.

## Acknowledgments

This research was supported by a National Health and Medical Research Council Research Fellowship and MS Australia Postdoctoral Fellowship (S.Y.), Leader Fellowship (A.W.H., 2009079), Career Development Fellowship (J.E.P., 1107599), and Investigator Fellowship (J.E.P., 1175781). K.A.F. is supported by the Alex Gadomski Fellowship, funded by 10.13039/501100022890Maddie Riewoldt’s Vision. Additional grant support was provided by the 10.13039/501100000925National Health and Medical Research Council (1150144, 1143163, and 2020517), the 10.13039/501100000923Australian Research Council (180101405), and the 10.13039/100012503Royal Hobart Hospital Research Foundation. The content is solely the responsibility of the authors and does not necessarily represent the official views of the funding agents. The funders had no role in study design, data collection and analysis, decision to publish, or preparation of the manuscript.

## Declaration of interests

The authors declare no competing interests.

## Web resources

Gene Expression Omnibus (GSE196830), https://www.ncbi.nlm.nih.gov/geo/query/acc.cgi?acc=GSE196830
GitHub, https://github.com/powellgenomicslab/onek1k_phase1
Human Cell Atlas (HCA), https://cellxgene.cziscience.com/collections/dde06e0f-ab3b-46be-96a2-a8082383c4a1

## Supplemental information

Document S1. Figures S1–S12, Tables S1–S4, S9, S14, S15, S17, S20, and S21, supplemental note, and supplemental methods

Data S1. Tables S5–S8, S10–S13, S16, S18, S19, S22, and S23

Document S2. Article plus supplemental information
