# Supplementary material for: The impact of sex on the immune system explored at the single-cell level
Source: Am J Hum Genet. 2026 May 7;113(5):1006–23. doi: 10.1016/j.ajhg.2026.04.003 (PMC13277697; doi:10.1016/j.ajhg.2026.04.003)
Supplement: Document S1. Figures S1–S12, Tables S1–S4, S9, S14, S15, S17, S20, and S21, supplemental note, and supplemental methods [file mmc1.pdf]

The American Journal of Human Genetics, Volume *113*

Supplemental information

The impact of sex on the immune system explored at the single-cell level

Seyhan Yazar, Jose Alquicira-Hernandez, Kristof Wing, Anne Senabouth, Stacey Andersen, Kirsten A. Fairfax, Alex W. Hewitt, Joseph E. Powell, and Sara Ballouz

Supplemental Notes

Impact of age on sex differences in immune cell types

Cell type abundances change with age, and this occurs in a sex-specific manner [1; 2]. The sex-specific changes in cell proportions with age is linked to hormonal changes during puberty, menstruation and menopause[3] and loss of clonal diversity in stem cell populations[4]. When comparing proportions to recorded biological age **(Figure S1, Table S2)**, we see clear correlation patterns in NK cells (Rho=0.32, FDR~0.002)[5] and CD8+ Naïve T-cells (Rho=-0.67, FDR< 0.001)[6], both of which change with age irrespective of sex. However, we see no significant correlation with Tregs with age which had been reported[7], instead NK proliferating cells, Plasmablasts, CD8+ TEM and gamma-delta T cells (gdT) are correlated with age in females (Rho NK proliferating=0.17 FDR~ 0.001, Rho CD8+ TEM=0.23, FDR~3.7×10^{-7} and Rho gdT=-0.25, FDR~4.4×10^{-8}) but not in males.

Cell-type and sex-specific co-expression replicates known biological functions

In addition to genetic control, we also wished to check whether observed sex-biased expression was linked to co-variation (*i.e.,* co-expression or co-regulation). Genes that co-vary are believed to be co-functional, either under common regulatory control or are in common pathways. Like gene set enrichment analyses, this approach includes additional network information in the enrichment, defined as a gene-gene interaction. Thus, analysing co-expression gene-gene networks that are conditioned on properties of the underlying cell types, tissues or sex highlights modules or interactions that are specific to these conditions. To assess sex-specific networks derived from PBMCs, we built cell-type and sex-specific networks from the data. We performed differential co-expression to determine the sex and cell-type specific modules and gene sets that differ by sex and/or cell type (**Figure S7-10, Table S22**). To perform this robustly and remove confounding variables such as batch effects, we built separate networks per pool and aggregated these networks[8]. By conditioning on sex and cell-type, we aimed to remove co-variation confounded with these conditions and capture the intrinsic variation within each "state". We use the performance of the neighbour-voting algorithm in cross-validation (EGAD[9], **Methods**) to measure enrichment for known biological pathways and gene sets, with model performance measured by the averaged AUROC (area under the ROC curve) for each group across the *n*-folds (**Figure S7A**).

Using the Gene Ontology (GO [10; 11]) slim gene sets to measure broad biological functions first, we find similar average AUROC scores between the sexes of the same cell-type, with average scores varying based on cell-type (**Figure S7B,** AUROC~0.53-0.58). The joint aggregate networks show slightly higher performances than the sex-stratified networks, even with down-sampling, suggesting that some of the connections within these joint networks are lost when conditioning on sex – *i.e.*, sex has some influence on co-expression and contributes to the broad GO pathways and functions (AUROCs +0.02). We see that the individuals' network aggregates outperform the cell-type specific networks (AUROC~0.6), highlighting that cell-type composition (variation) drives a

fraction (+0.04) of the additional co-expression in these networks. Notably, these performances are still just above average, reflecting the sparsity in single-cell RNA-seq data, as many genes are not expressed or detected and could not be assessed. The co-expression analysis reaffirms, as in bulk data, that a large fraction of the co-expression we observe is driven by cellular composition [12]. Conditioning on cell type removes these connections in the network, impacting the AUROC performance. Sex, on the other hand, has much less of an effect, as removing sex-specific connections in the network does not impact the AUROC as much.

We then looked at the specific gene set module differences between the networks (**Figure S7C**). The highest performing gene set module in most of the cell-types was GO:0003735 (structural constituent of the ribosome, AUROC~0.79),yet had lower performances in NK proliferating and plasmablast cells (AUROC~0.61). On the other hand, NK proliferating and plasmablast cells co-express mitotic-related genes (GO GO:0000278, mitotic cell cycle AUROCs~0.75), while other cell types do not (AUROCs~0.5). To identify functional specificity further, we ran our analysis on additional gene sets from the Molecular Signature Database (MSigDB[13]), with a focus on the HALLMARK[14], KEGG[15], REACTOME[16] and BIOCARTA[17] gene sets and pathways. As before, we see cell-type specific networks scoring highly in particular pathways and gene sets related to their cell-type specific functions. Of note were monocyte-specific networks, where higher AUROCs in pathways linked to hypoxia, apoptosis and TNFα signaling were similar to the pathways found to be sex-differentially expressed in those cell-types (**Figure S10**). This suggests that these pathways and their genes show differential activity in monocytes [18; 19] dependent on sex.

In addition to pathways, co-expression may reflect co-regulation. Thus, we tested for TF-target enrichment within the networks, and whether we could observe differences in co-regulation between the sexes. We used the Gene Transcription Regulation Database (GTRD)[20]. TF-target genes dataset curated in MSigDB and observed highly correlated performances between the sexes once more (**Figure S7D**). The biggest differences were in the co-expression values of the target genes of *NR5A1* (steroidogenic factor 1 (SF-1)), a transcriptional activator involved in sex determination, and *CREBL2*[21], linked to adipose tissue differentiation. Whether these genes play roles in the immune system requires additional validation, but their roles in sex differences and sex-specific phenotypes (such as adipogenesis) are well established.

To further explore the performance differences, we looked at the topological network differences (i.e., gene-gene connections, **Figure S7E**) by comparing the pairwise co-expression ranked values of the cell-type specific aggregate networks. On average, ~16% of the edges change between the networks, with the most differences between the CD4+ T cell aggregates and the other cell types (~38%). Overall, we see that sex differences on average are low, as most cell-type specific networks have greater similarities to their same cell-type or similar cell-type within the hematopoietic hierarchy. To further measure the impact of sex and cell-type conditioning on the network topology, we then compared the change in overall connectivity per gene by assessing the node degree changes. As in the previous analysis, node degrees were similar between cell-type specific aggregates conditioned

on sex, with most differences appearing between the different cell types (**Figure S3F**). On average, less than 1% of genes (~99) have changes in their node degree standardized residuals of 3SDs across cell types (holding sex constant). In contrast, between the sexes of the same cell type, there are less than ~0.5% of genes that have significant node degree differences (~43 genes). We find these genes to be enriched for those on the sex chromosome, a few of which are paralogs (e.g., *UTY* and its X paralog *KDM6A/UTX*, *ZFY* and *ZFX, KDM5D* and *KDM5C*). X chromosome paralogs are likely more variable as they are known to escape X-inactivation. Overall, we observe little sex network differences from the autosomal genes, reaffirming our observation of few detectable changes in these networks as reflected by their similar AUROCs performance. This analysis highlights that sex differences have small effects on the broad biological pathways or functions in contrast to cell type, as highlighted by the more similar AUROCs in the former and specific gene set performances differences in the latter.

We identified core co-expressed gene-gene modules in the immune cell types along with gene expression differences. Our work highlighted differential co-expression changes between the sexes primarily a result of sex-chromosome genes, along with a few autosomal genes. The regulatory relationships that were impacted were not significant, but the differences do reflect changes to signalling pathways that are likely buffered in a sex-specific way. The Y chromosome is known to influence inflammatory pathways. As such, females have paralogs to several Y-specific genes that escape X-inactivation to compensate. Their co-expression partners and regulatory factors are likely shared, which we observe in our data. RNA-seq analysis may need to distinguish between the XY paralogs and hence expression estimates, and subsequently, co-expression relationships may be obscured or miscalculated.

Differential expression sensitivity analysis

To ensure our findings are not artifacts of approach or software implementation, we performed a sensitivity analysis. First, we applied Simes for global multiple test correction, which returned similar significant genes, except for cell types with low cell or individual counts which may have influenced the analysis's power (**Table S4**). To ensure that these were not only due to differences in power, we performed a downsampling analysis. We observed that most of these genes were robust to this process (**Table S7-8**). We further benchmarked our sex-DE results across Seurat v4.4.0 and Seurat v5.3.0. While Seurat v5 produced systematically larger absolute |log2FC| values due to updated pseudocount handling, the gene rank-order remained highly stable (**Figure S11**). Spearman correlation of |log2FC| values across all cell types was high (average rho = 0.88), confirming that the relative magnitude of sex-biased effects is preserved (**Figure S12**). Applying our significance thresholds (FDR < 0.05, |log2FC| > 0.1) to both versions yielded a 98% consensus overlap. Genes unique to v5 were typically marginal cases pushed over the fold-change threshold by version-specific scaling.

Supplemental Figures

Figure S1 Cell type proportions and age changes
Figure S2 Tests of normality of proportions for each cell type
Figure S3 Proportions compared across covariates
Figure S4 Downsampling differential expression analysis
Figure S5 Replication of unique DEGs in downsampling analysis
Figure S6 Sex-DEG enrichments
Figure S7 Co-expression by cell type and sex
Figure S8 Co-expression downsampling analysis
Figure S9 Differential co-expression comparisons
Figure S10 Functional enrichment results of aggregate networks using EGAD
Figure S11 DEG comparison between Seurat v4 and v5: log2FC
Figure S12 DEG comparison between Seurat v4 and v5: ranked log2FC

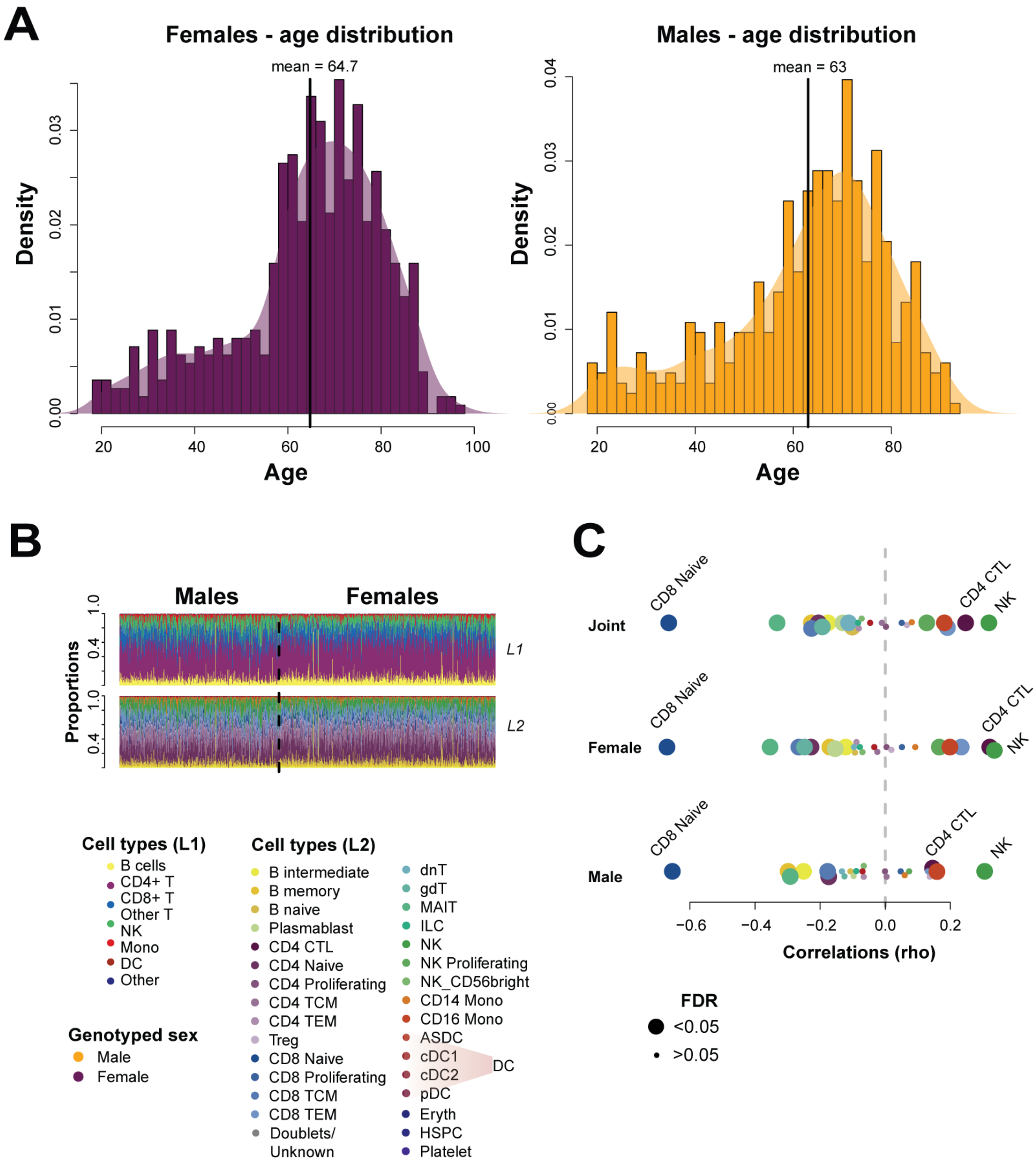

Figure S1 Cell-type proportions and age changes. (A) Distribution of ages for females and males in OneK1K. (B) Distributions of cell-type proportions for L1 (broad) classification and L2 classifications across all individuals. (C) Correlations of cell-type proportions to age for both sexes (joint), females only and males only.

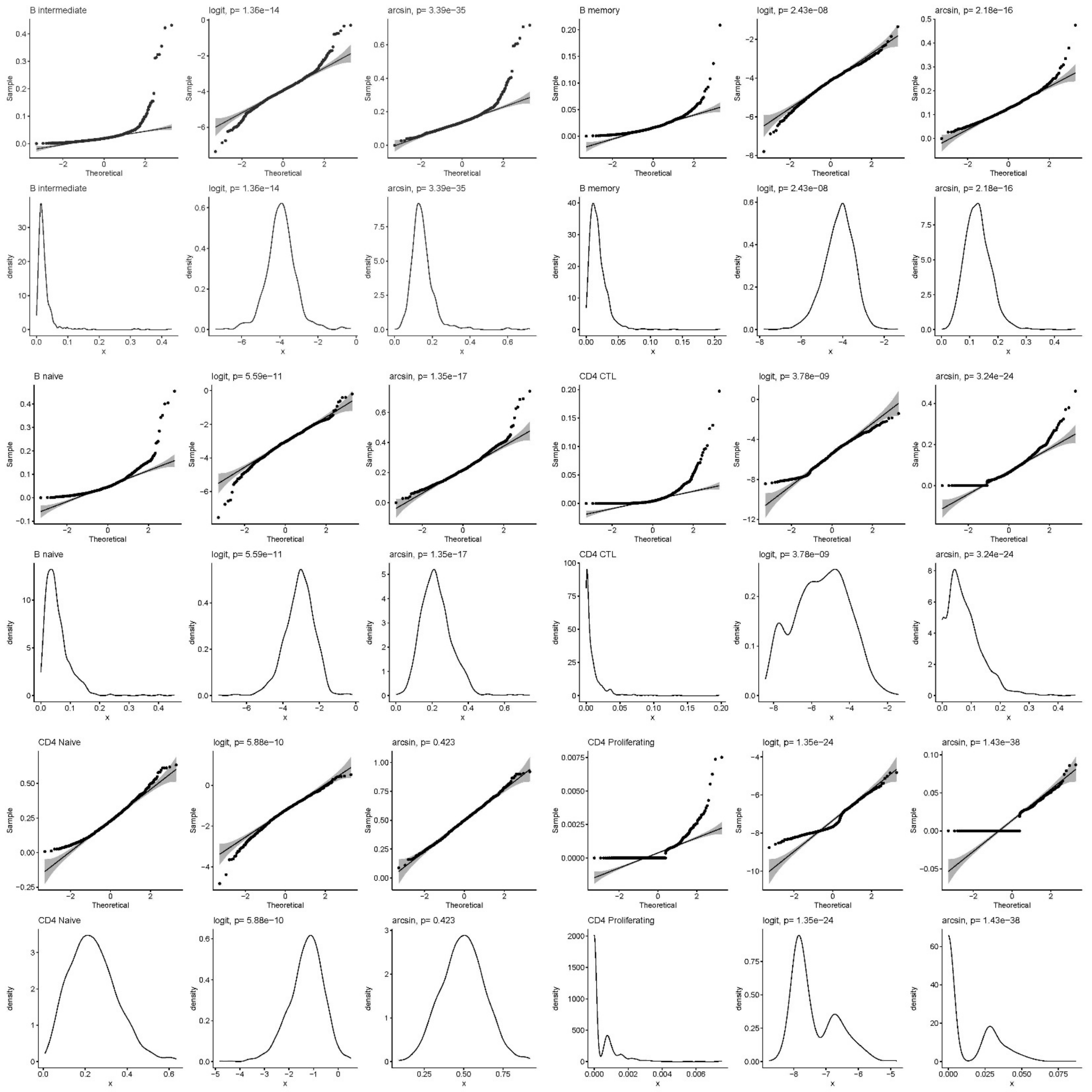

Figure S2 Tests of normality of proportions for each cell-type. Each QQ-plot shows the original proportions, the logit transformed and then the arcsin transformed data. Beneath each QQ-plot is the density distribution plot. Note, these are not split by sex. The remaining cell types are continued on the following pages.

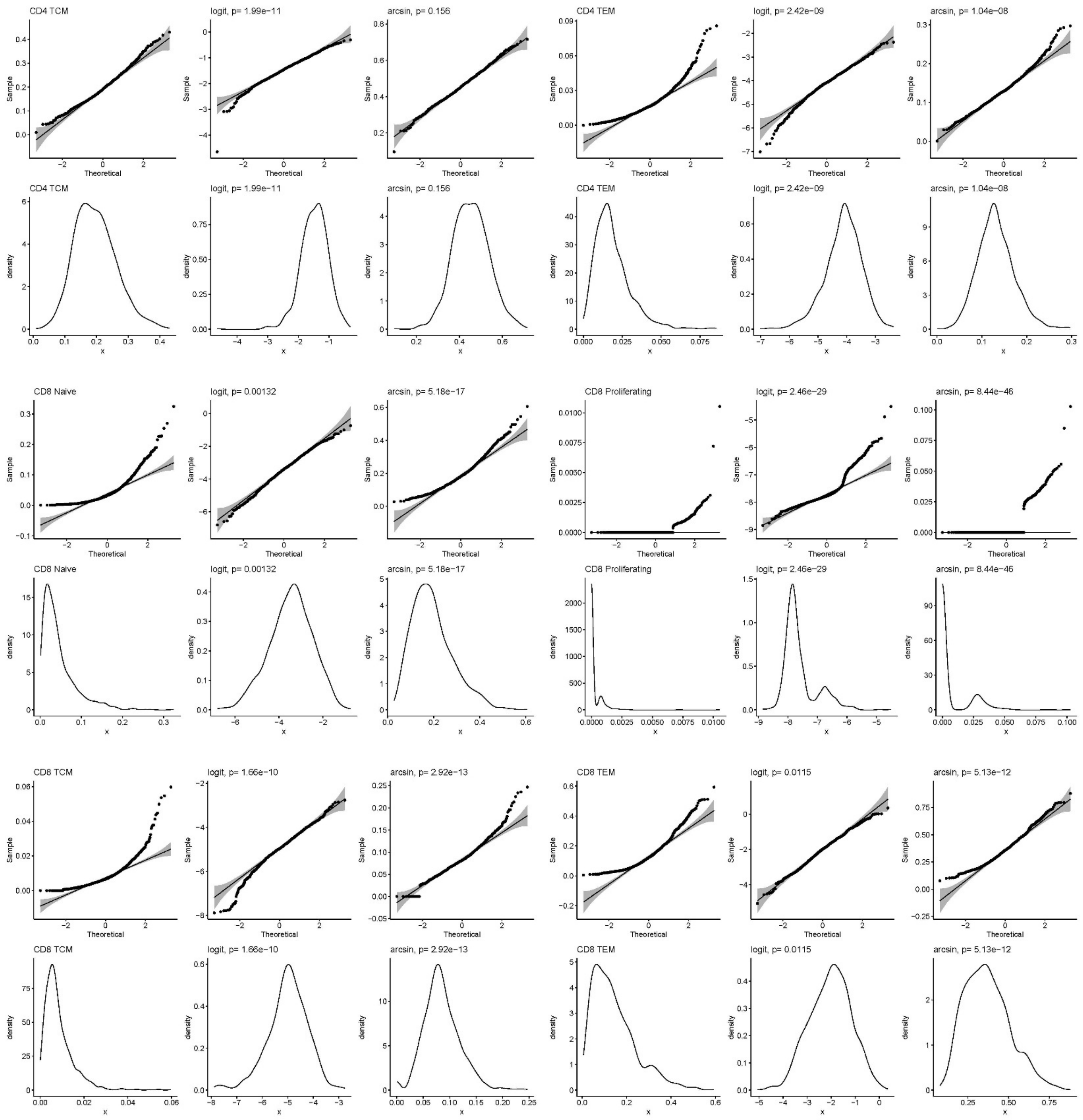

Figure S2 (continued) Tests of normality of proportions for each cell-type.

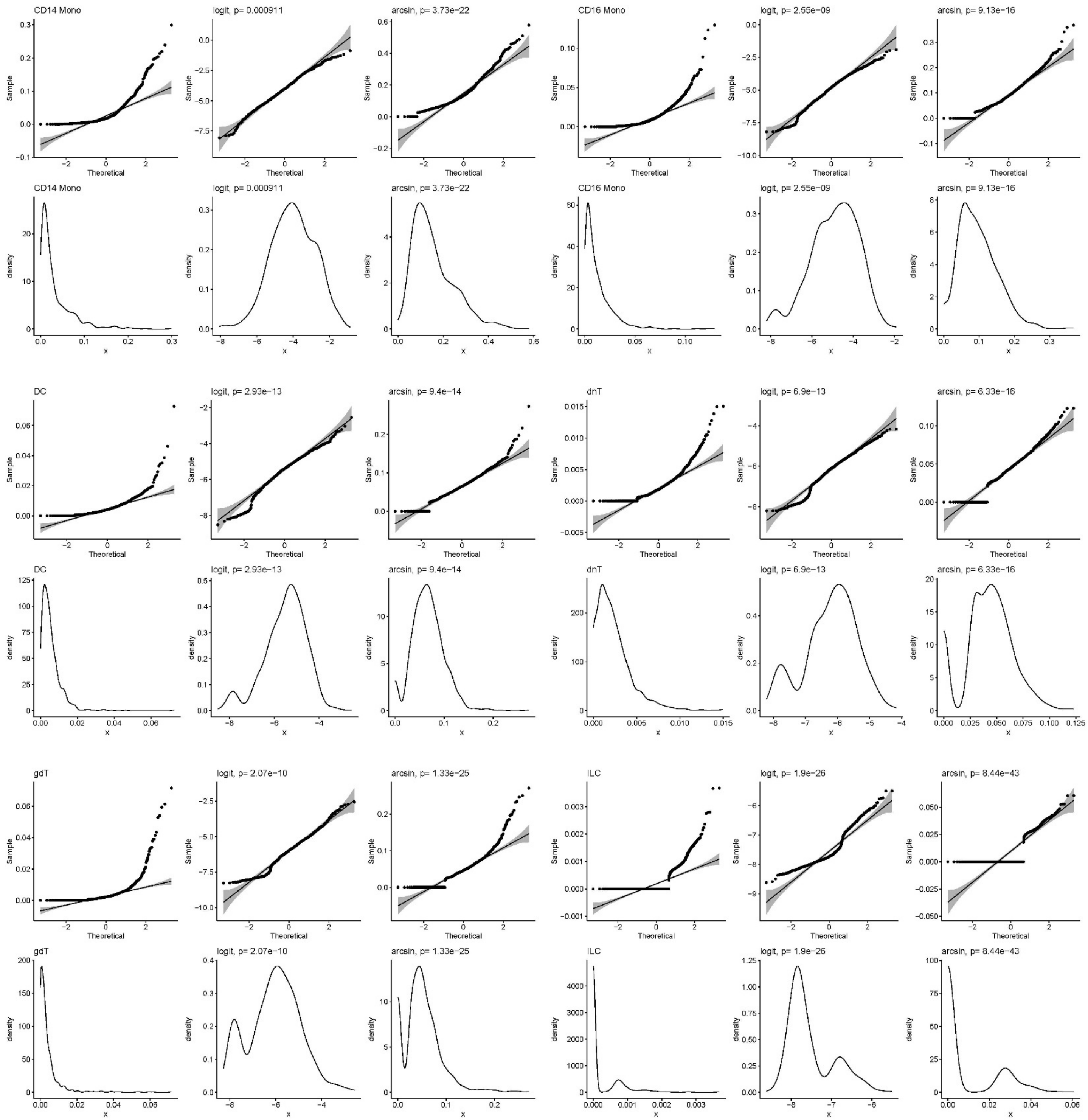

Figure S2 (continued) Tests of normality of proportions for each cell-type.

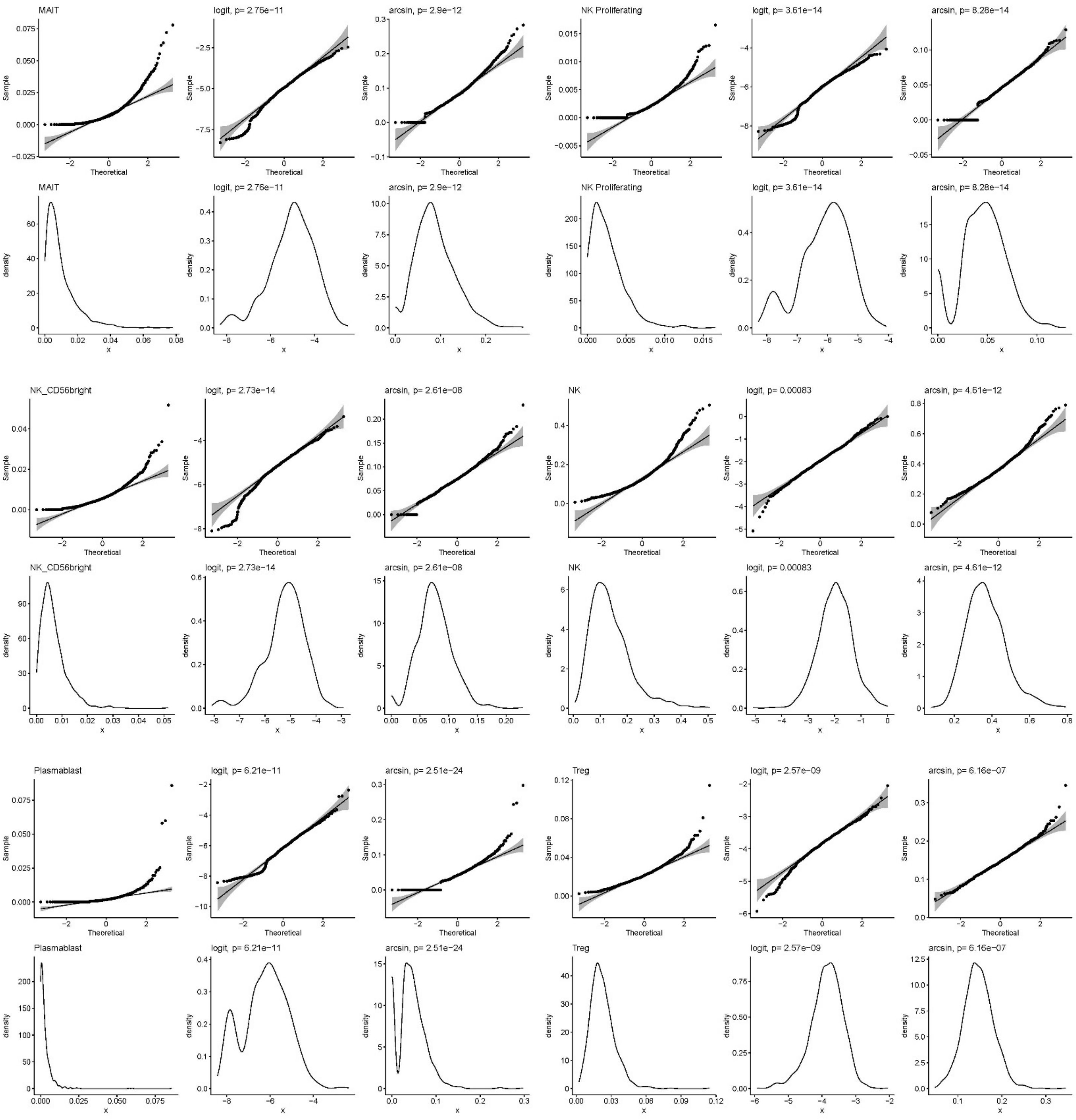

Figure S2 (continued) Tests of normality of proportions for each cell-type.

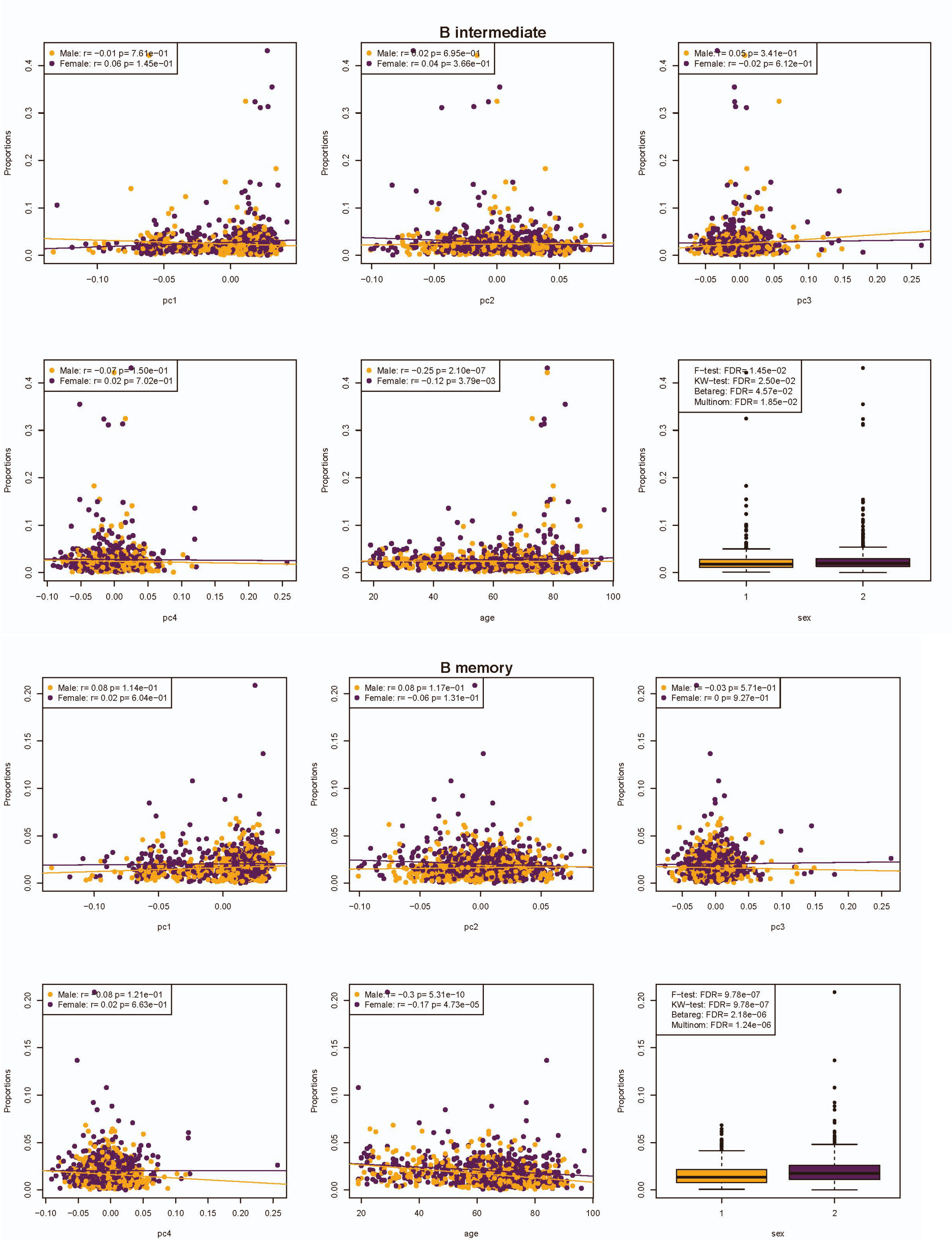

Figure S3 Proportions compared across covariates. Each scatter plot shows a variable (e.g., age, PC) versus the proportion, colored by sex. Purple are females, gold are males. A box plot shows the differences by sex. Each of the tests are labeled and marked in the plots.

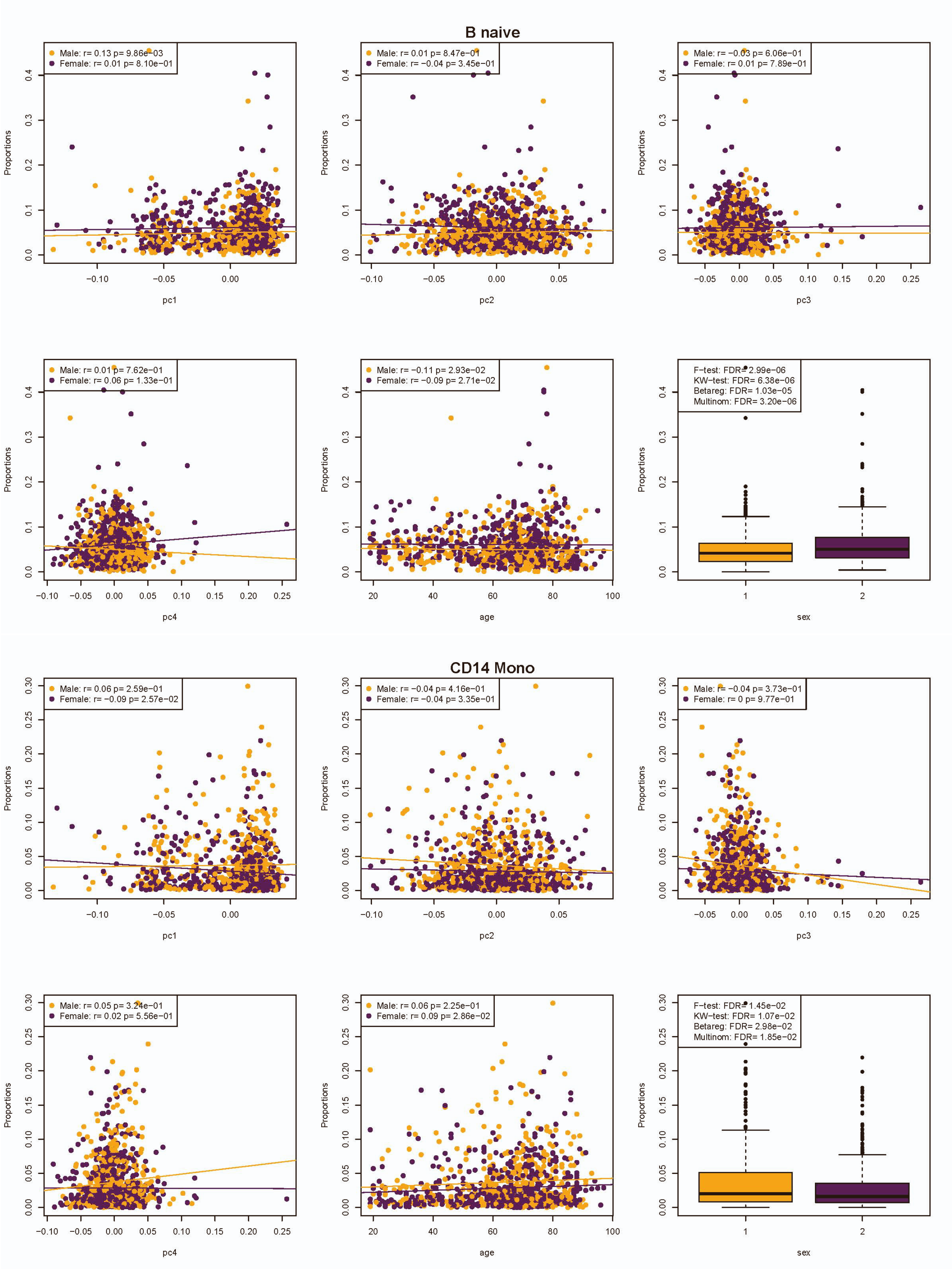

Figure S3 (continued) Proportions compared across covariates.

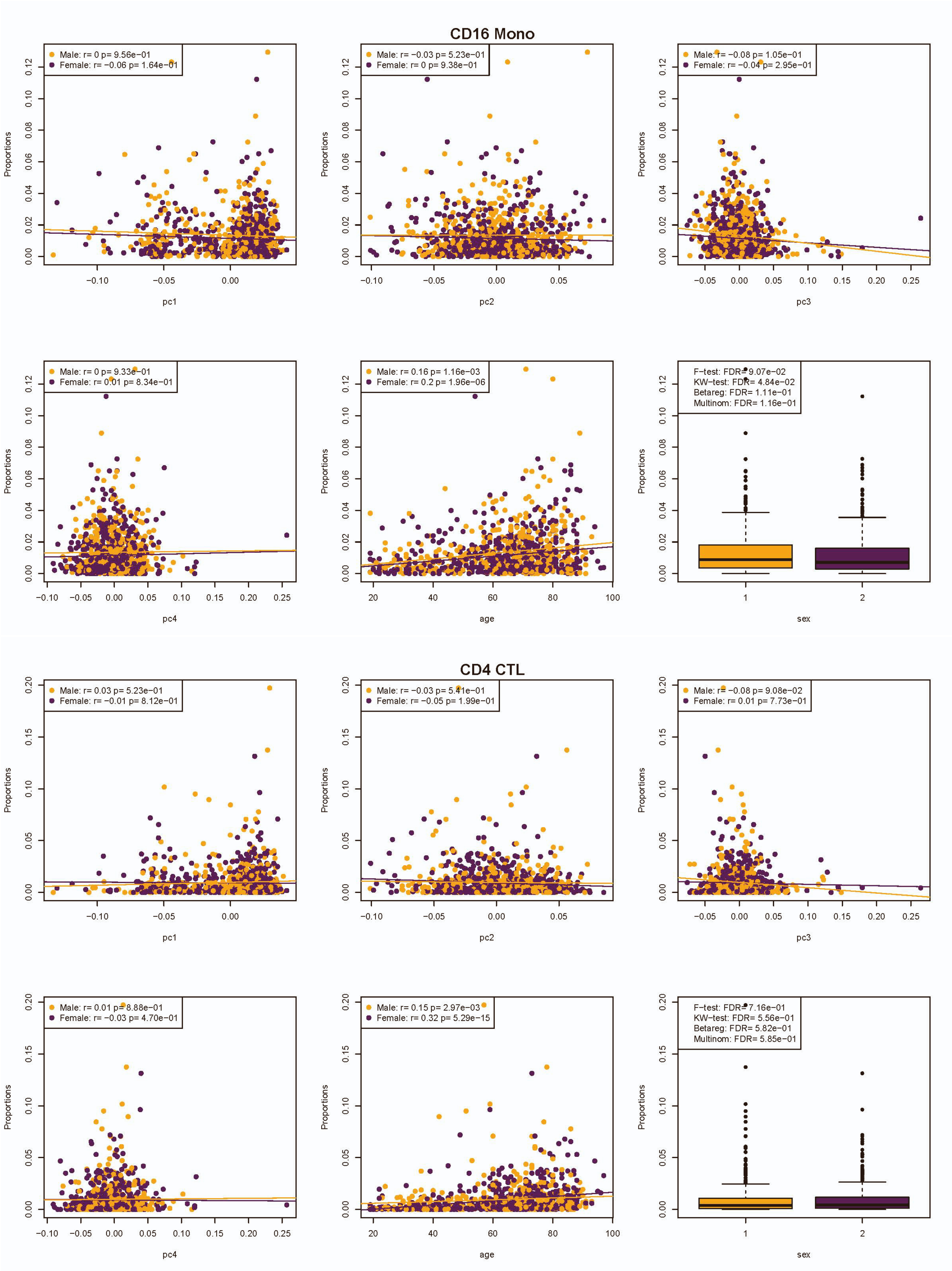

Figure S3 (continued) Proportions compared across covariates.

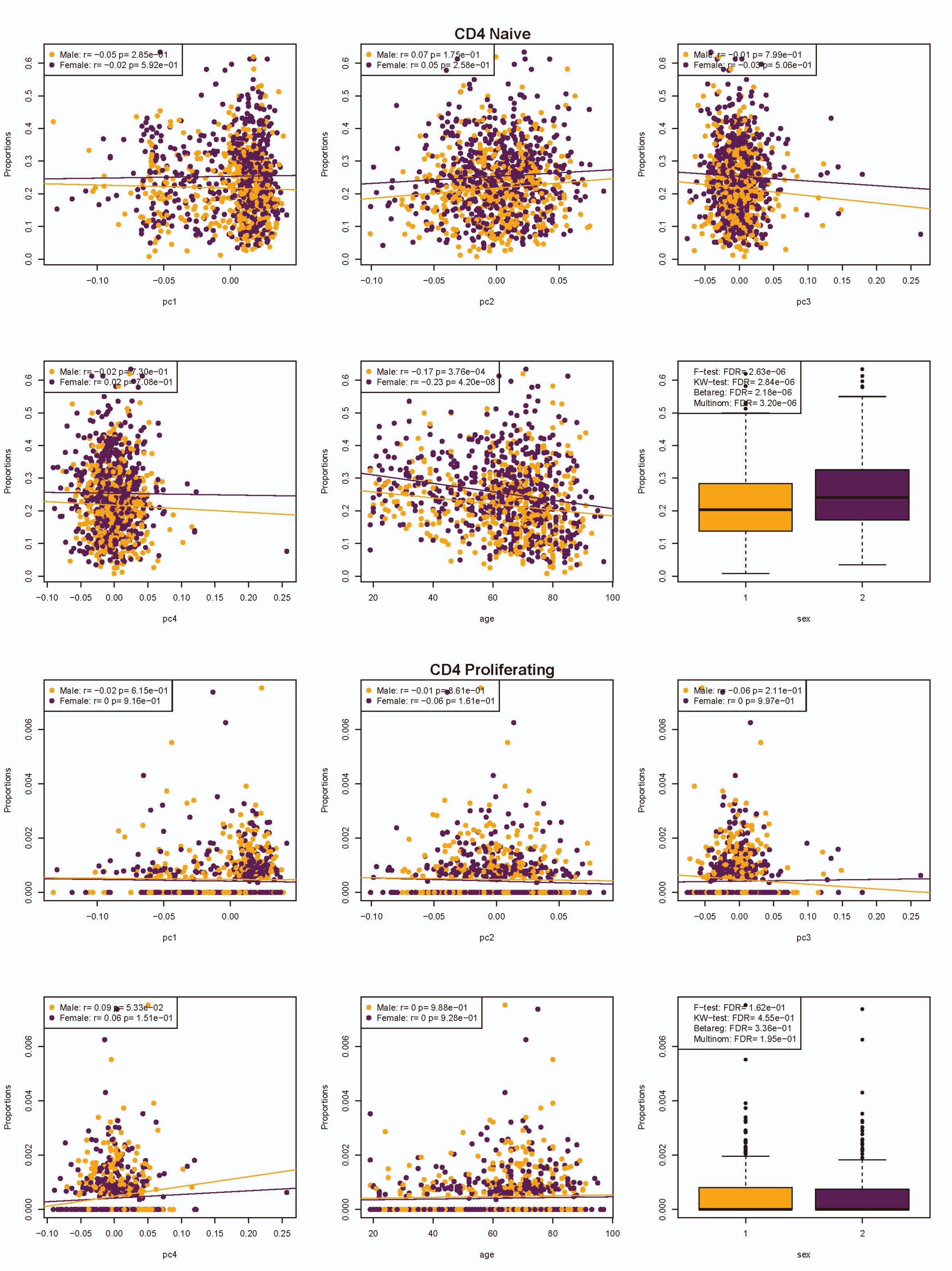

Figure S3 (continued) Proportions compared across covariates.

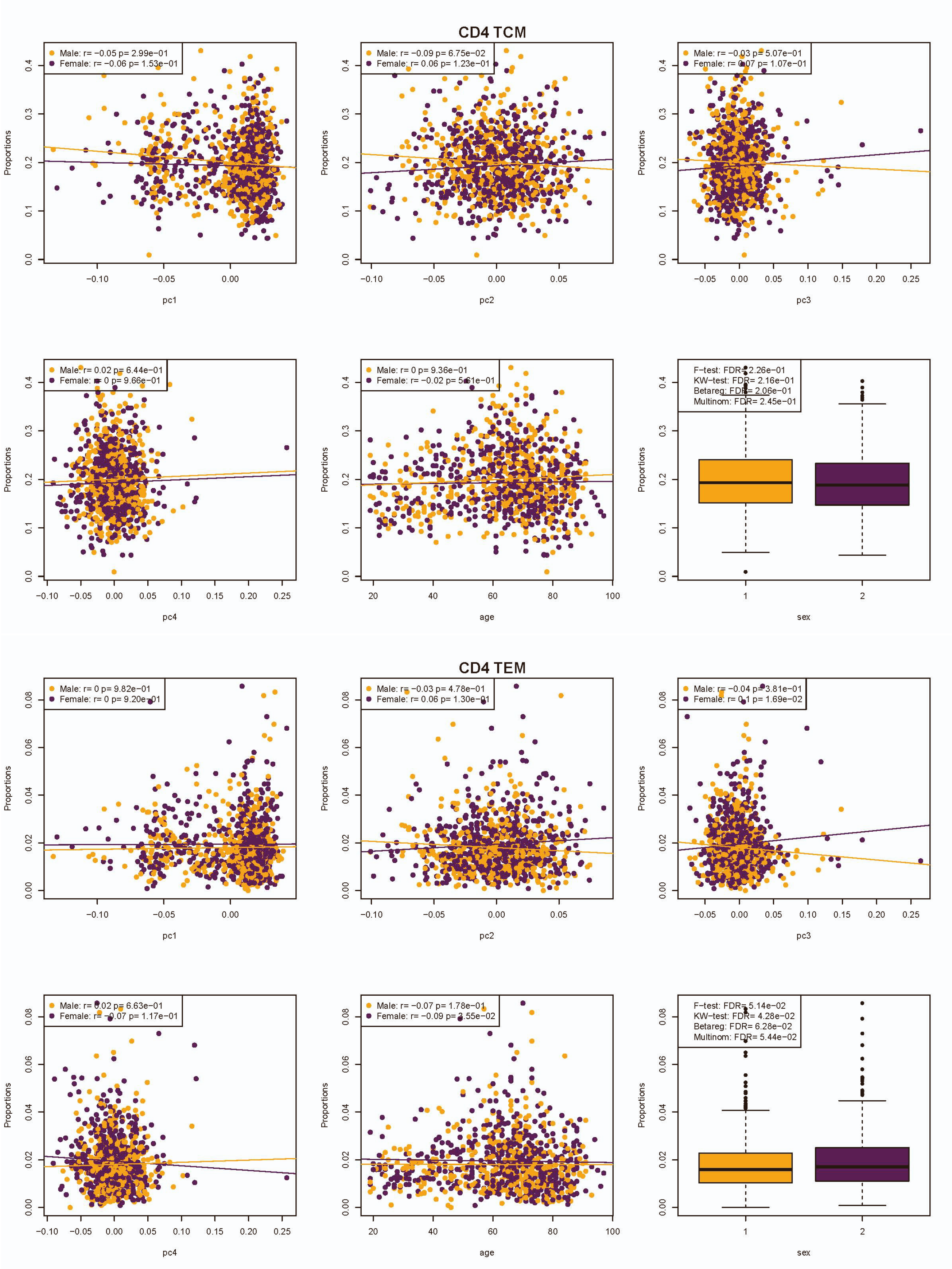

Figure S3 (continued) Proportions compared across covariates.

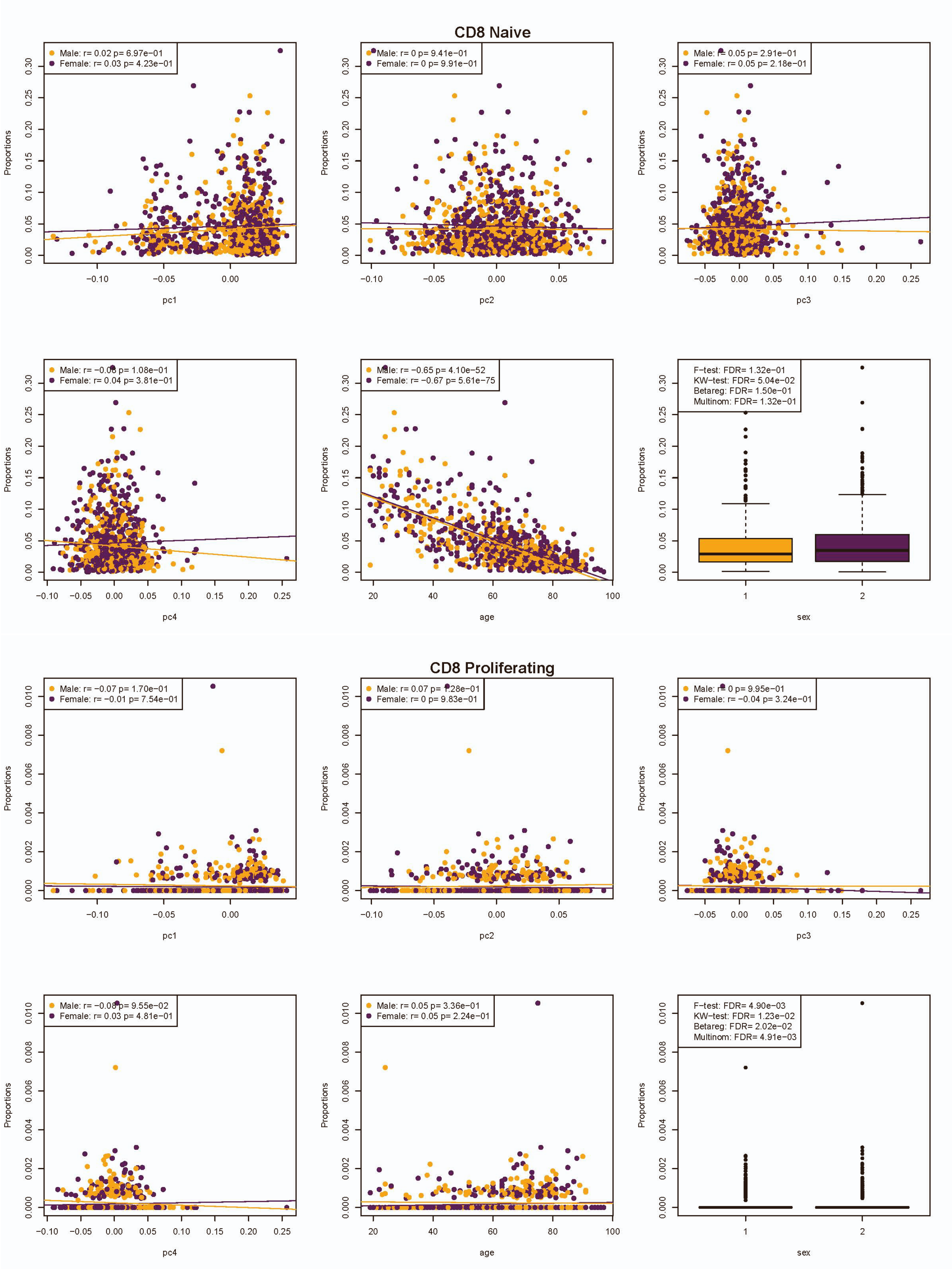

Figure S3 (continued) Proportions compared across covariates.

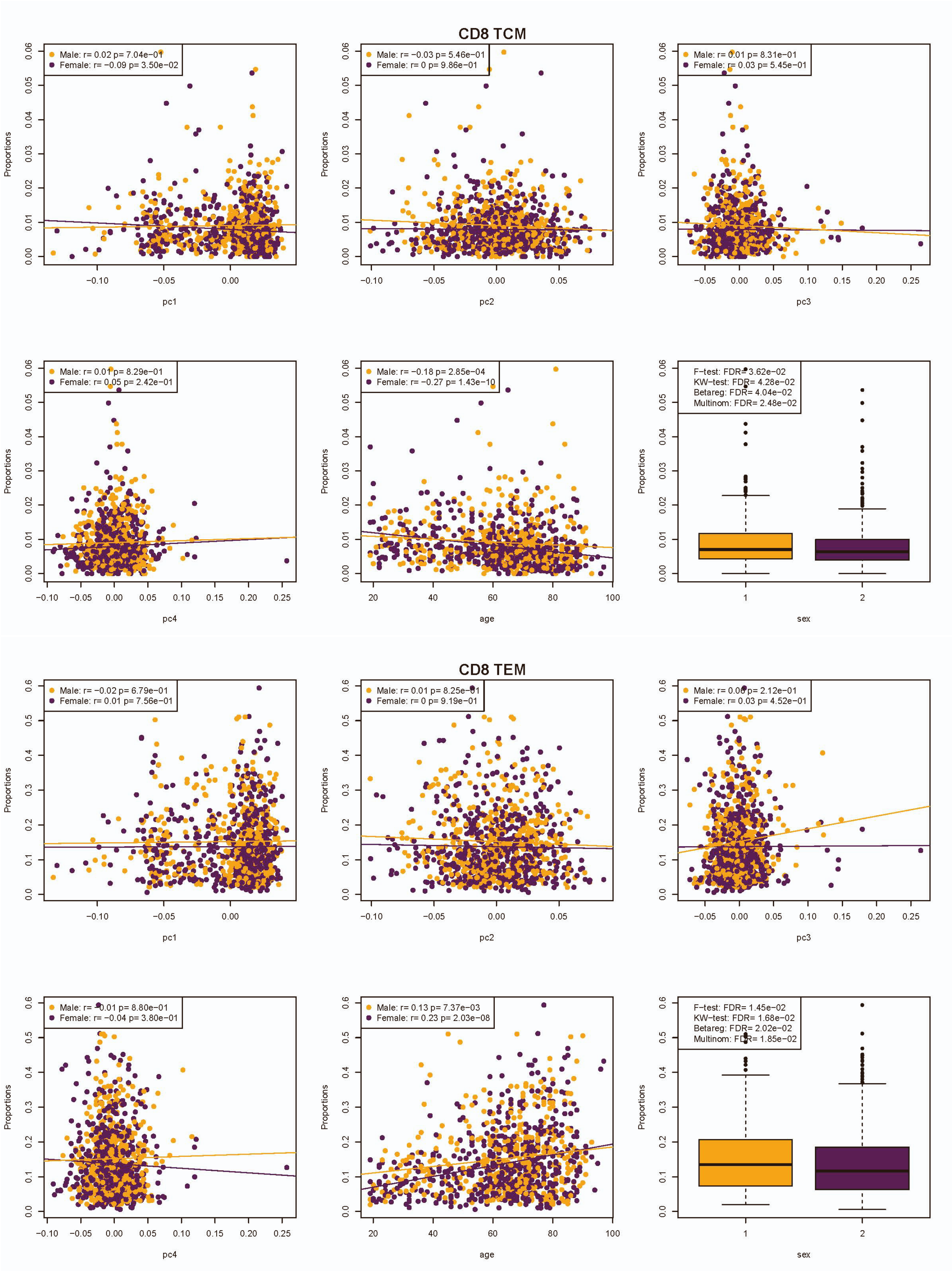

Figure S3 (continued) Proportions compared across covariates.

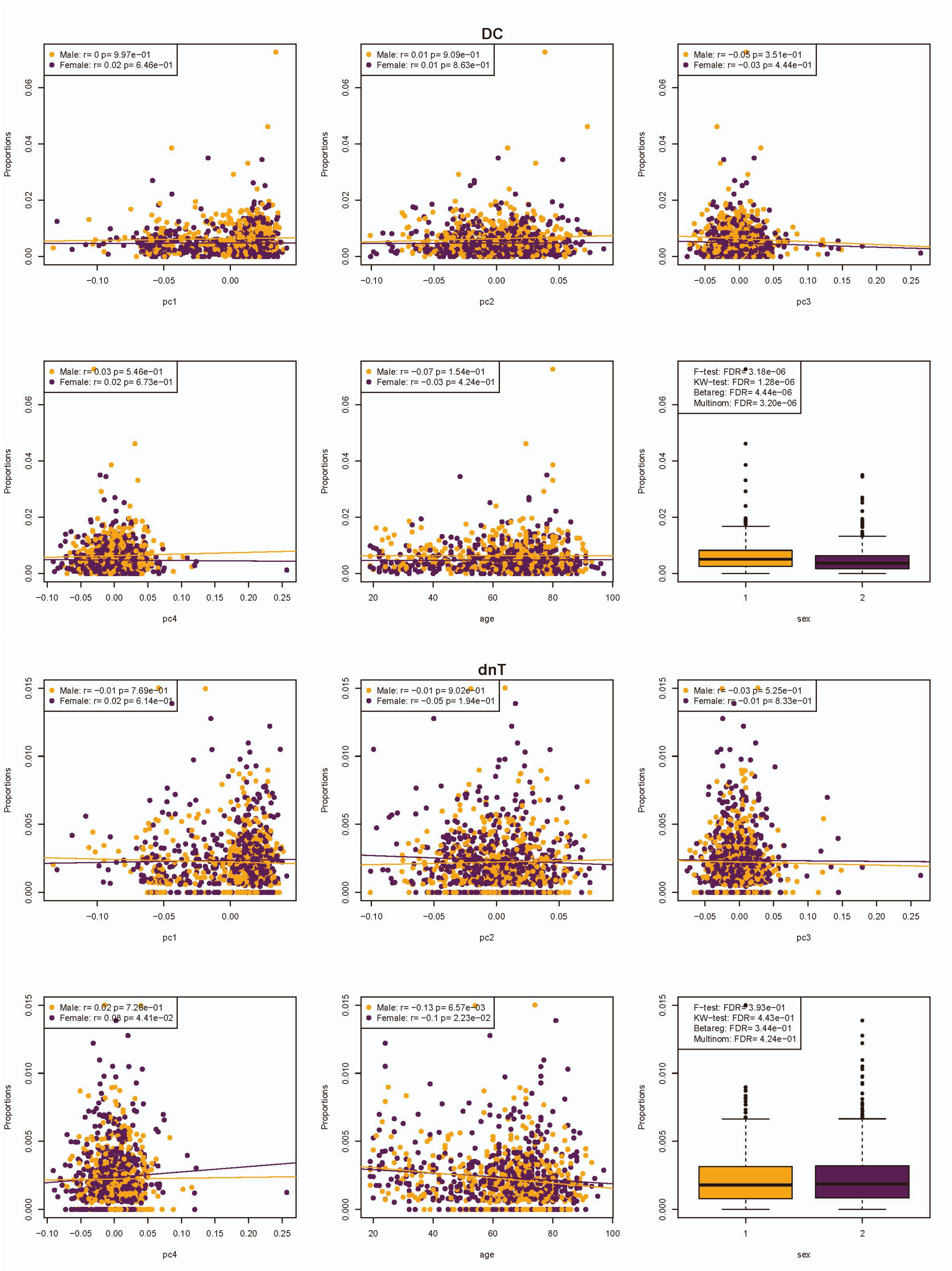

Figure S3 (continued) Proportions compared across covariates.

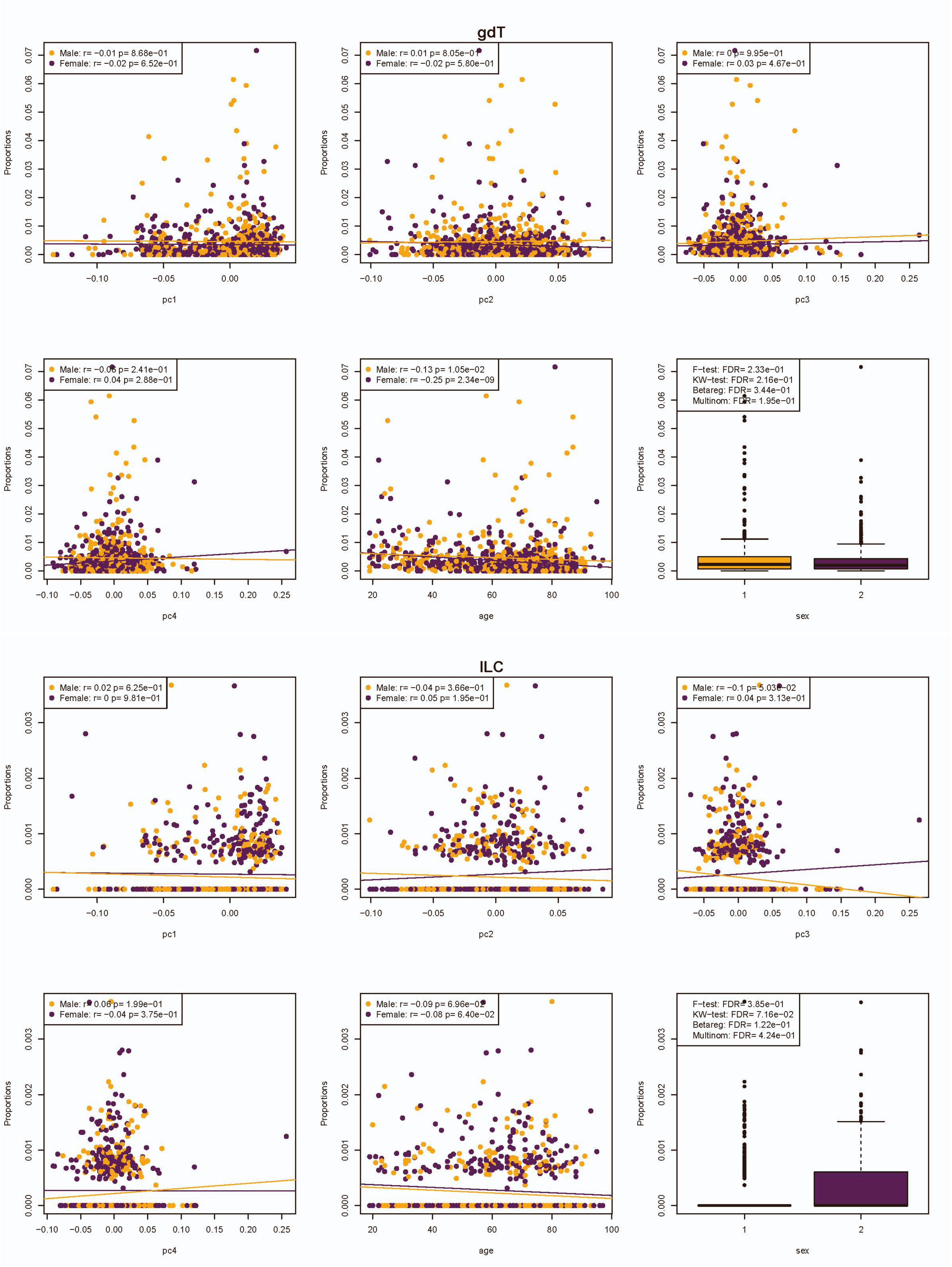

Figure S3 (continued) Proportions compared across covariates.

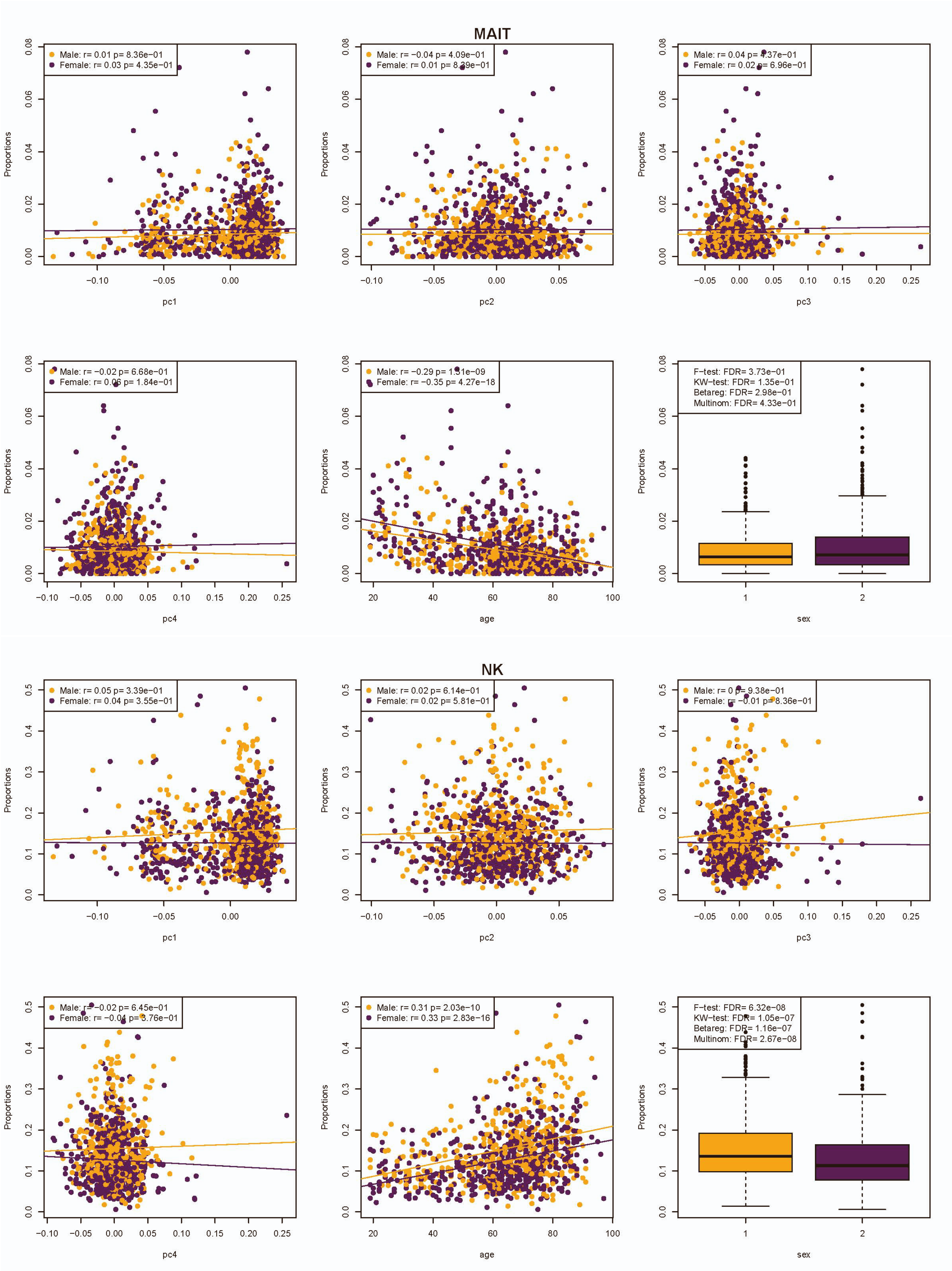

Figure S3 (continued) Proportions compared across covariates.

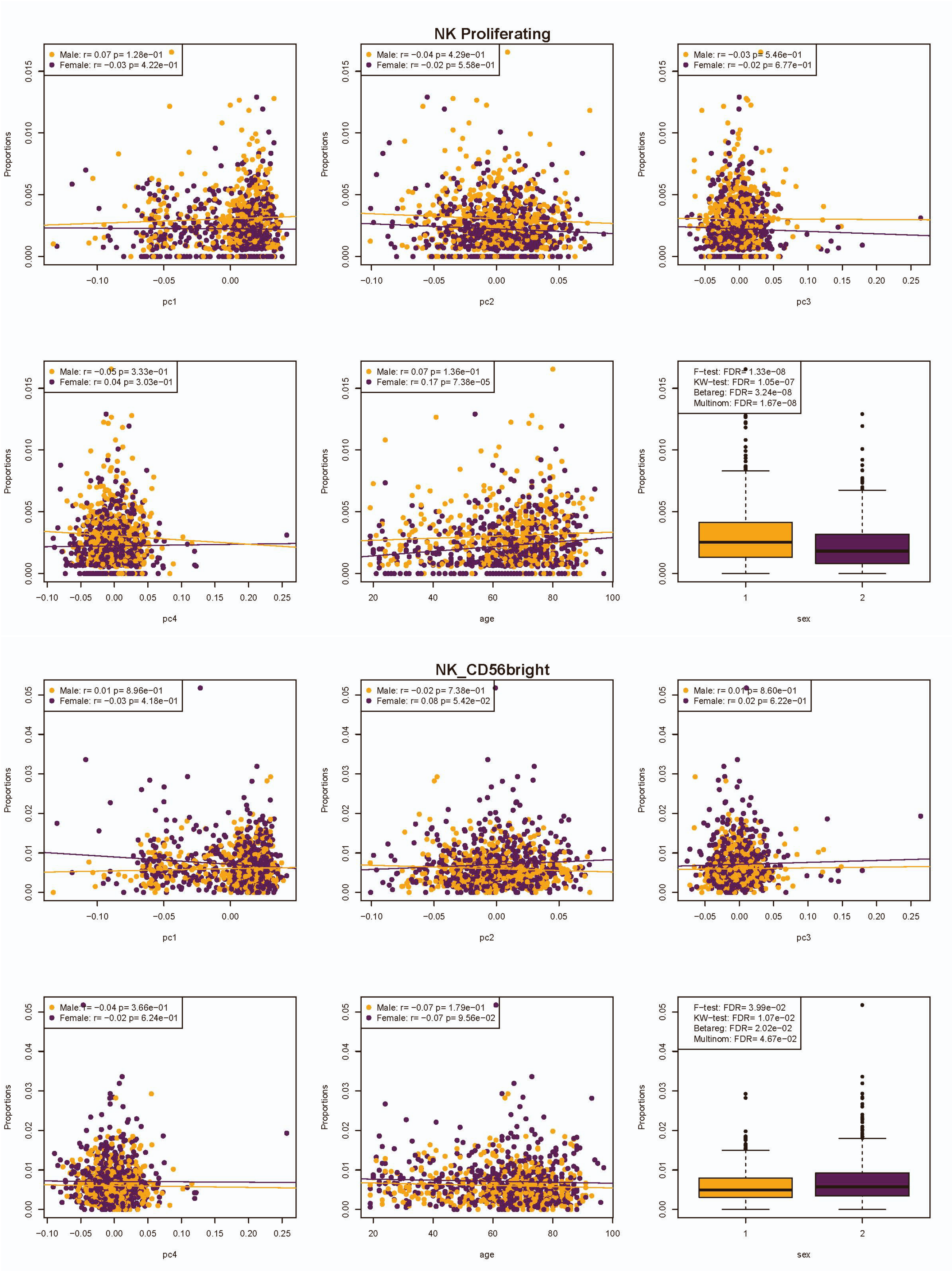

Figure S3 (continued) Proportions compared across covariates.

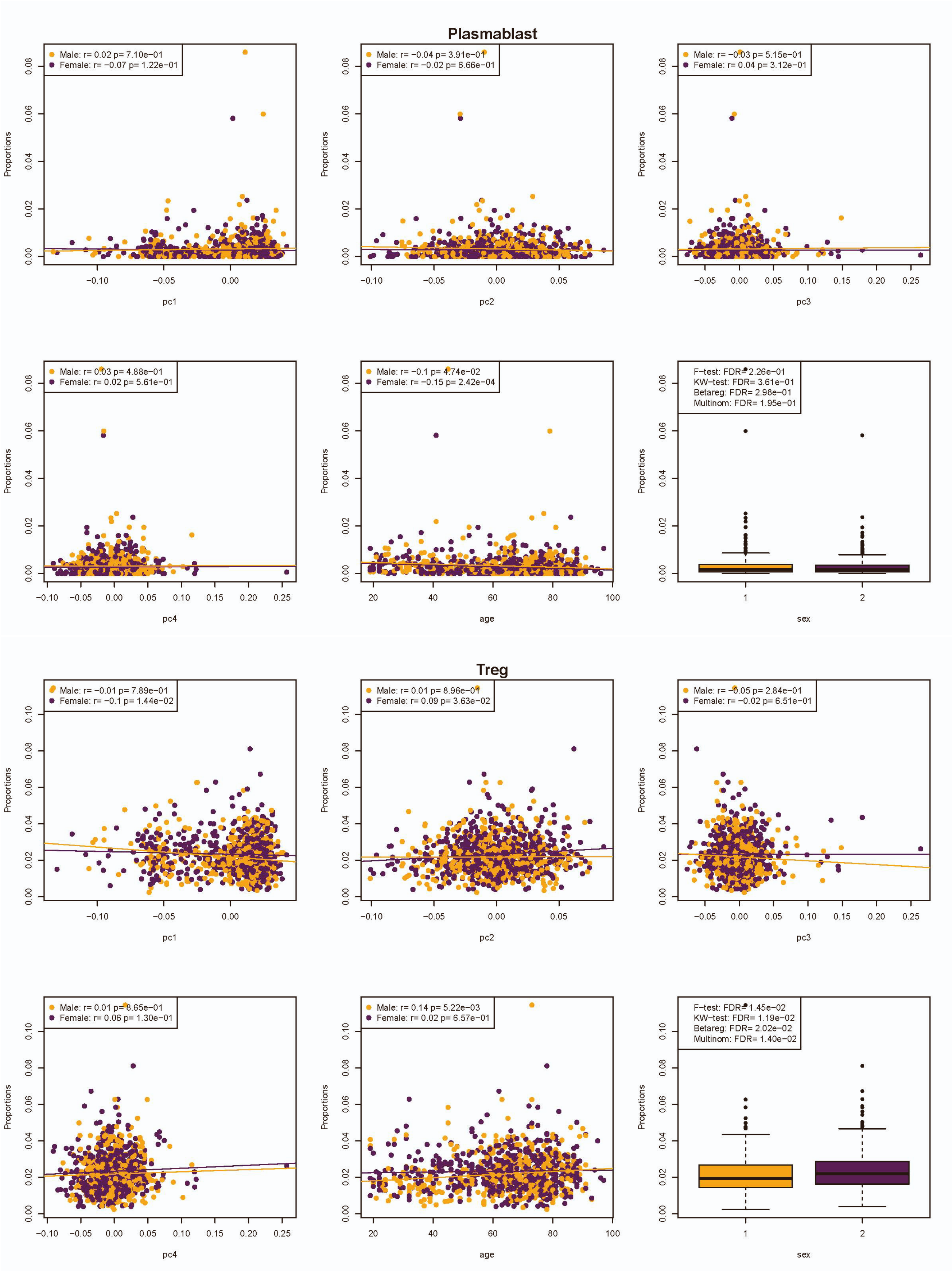

Figure S3 (continued) Proportions compared across covariates.

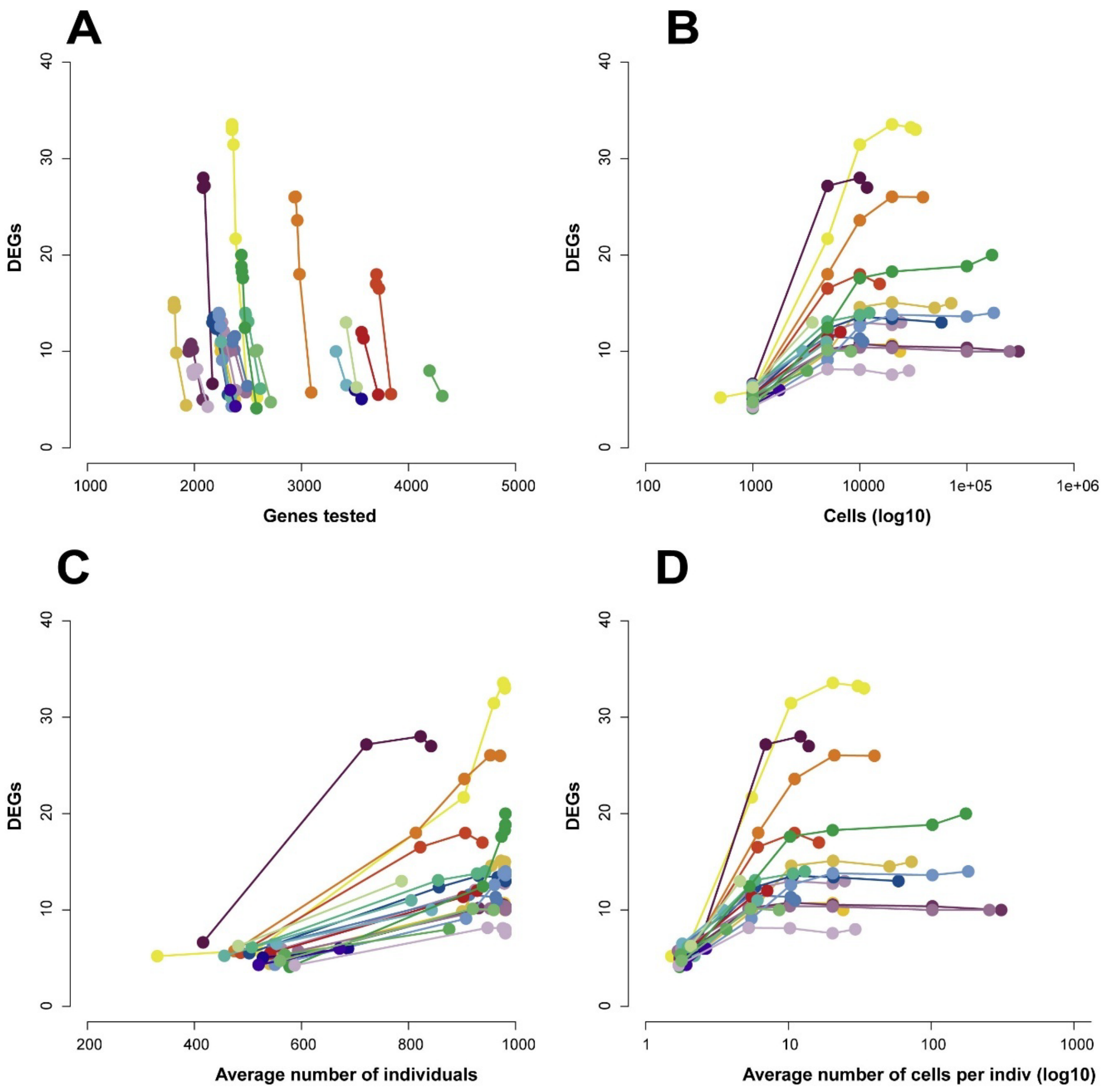

Figure S4 Downsampling differential expression analysis. (A) Plot of the number of genes tested and the resulting DEGs (total) per cell-type. (B) The number of cells downsampled versus the number of DEGs (total). (C) Number of individuals in the downsampled tests versus the DEGs. (D) The average number of cells per individual versus DEGs.

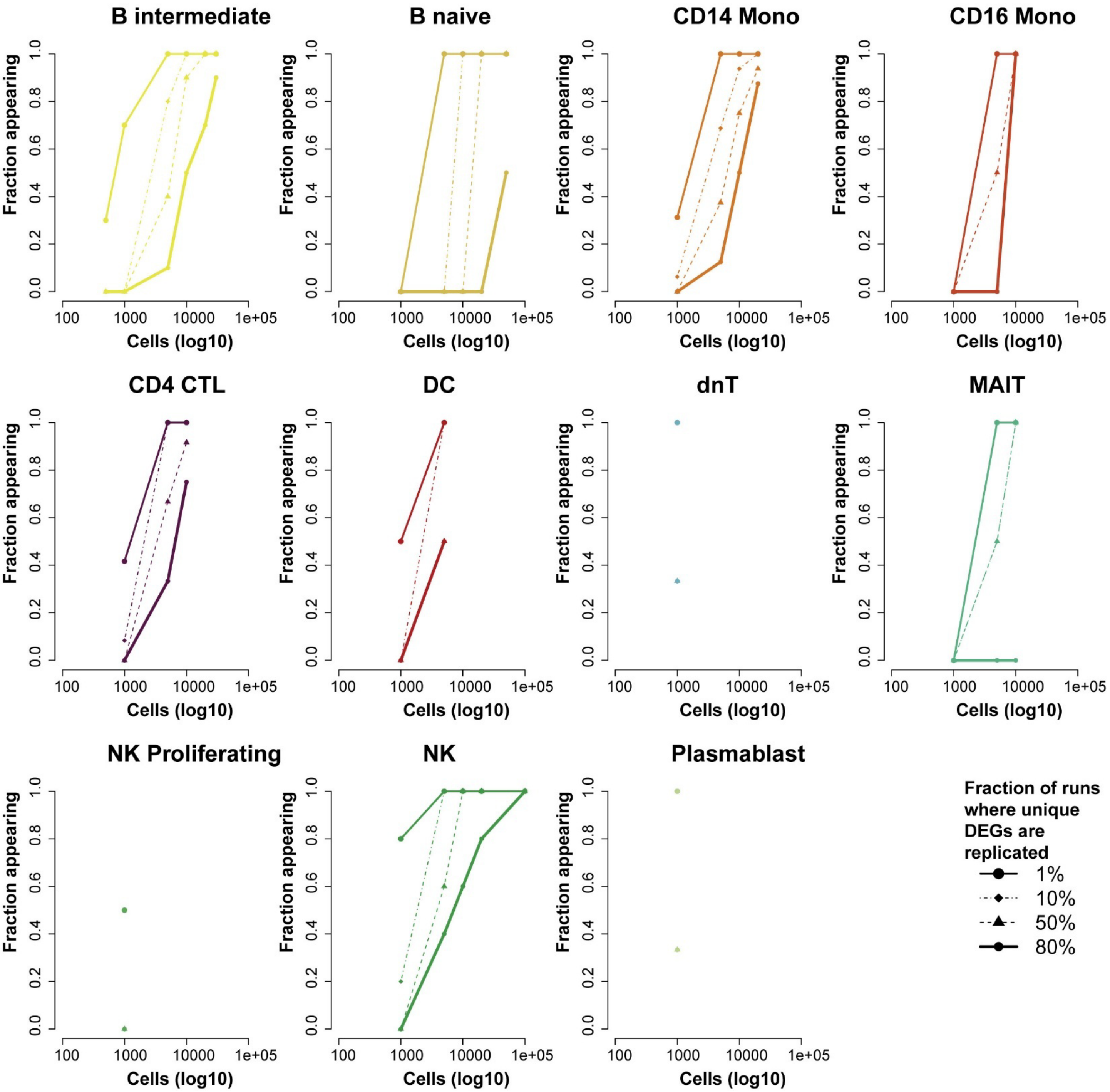

Figure S5 Replication of unique DEGs in downsampling analysis. Each plot indicates the frequency with which we consider a DEG replicated. In all these, we see obvious cell-type specific results that plateau suggesting that we are well powered for most of the analyses.

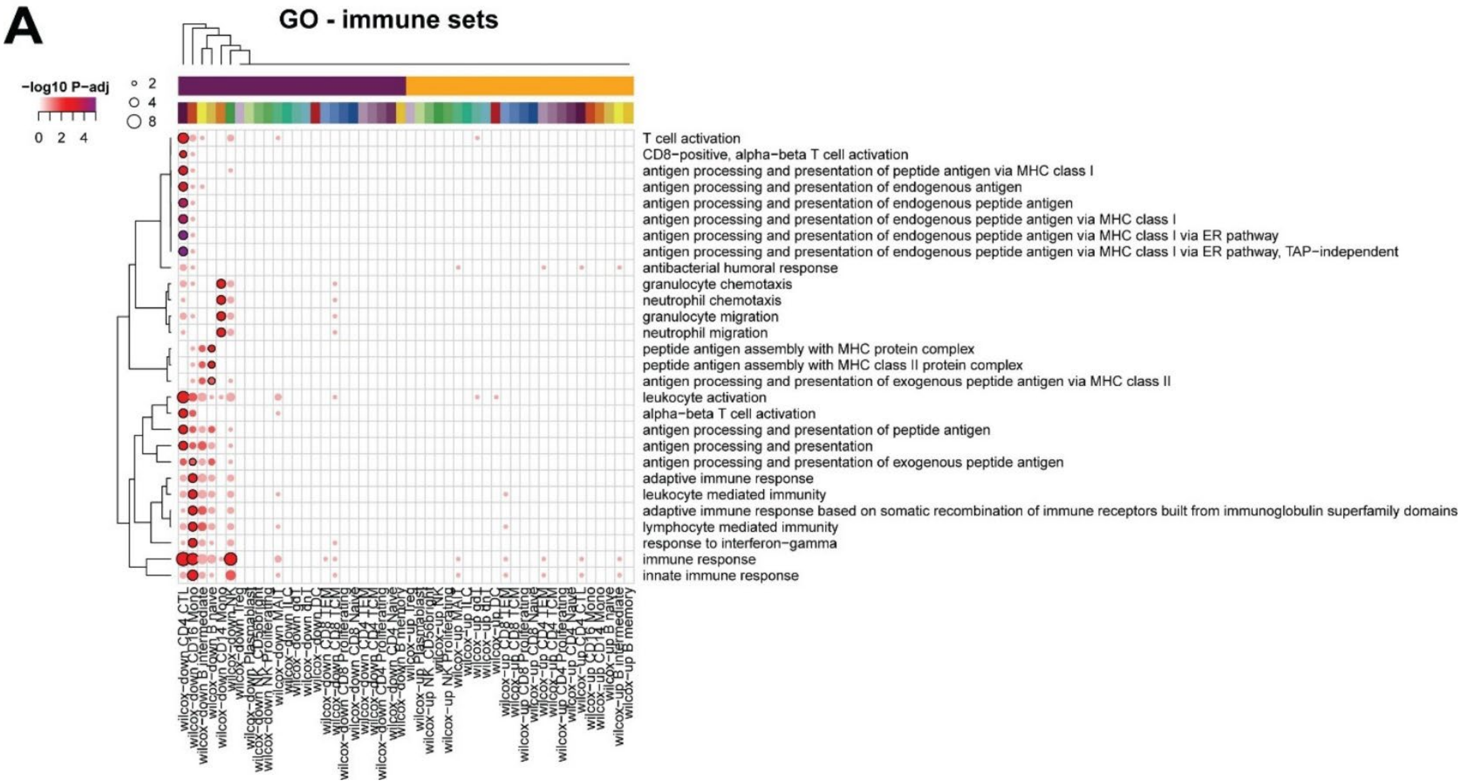

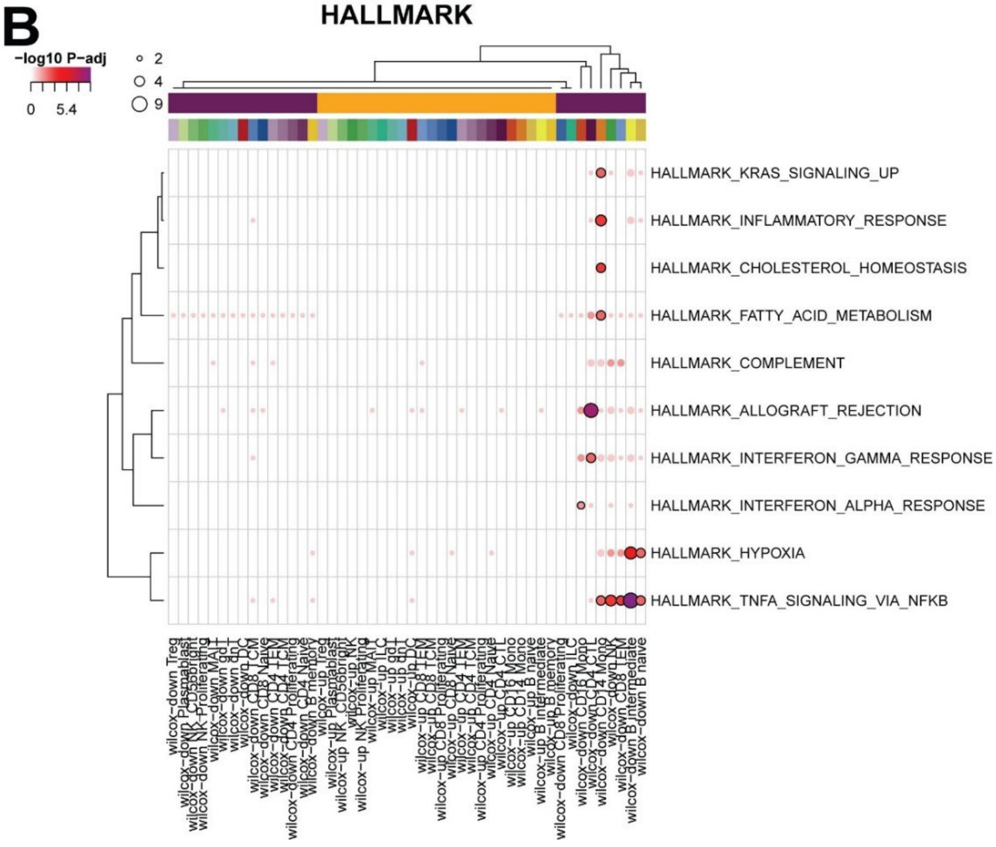

Figure S6 Sex-DEG enrichments. Gene set enrichment analysis for (A) GO immune sets, (B) HALLMARK, (C) KEGG/REACTOME/BIOCARTA, (D) HGNC, (E) Curated immune gene sets (MolSigDB C7) and (F) sex hormones and their targets.

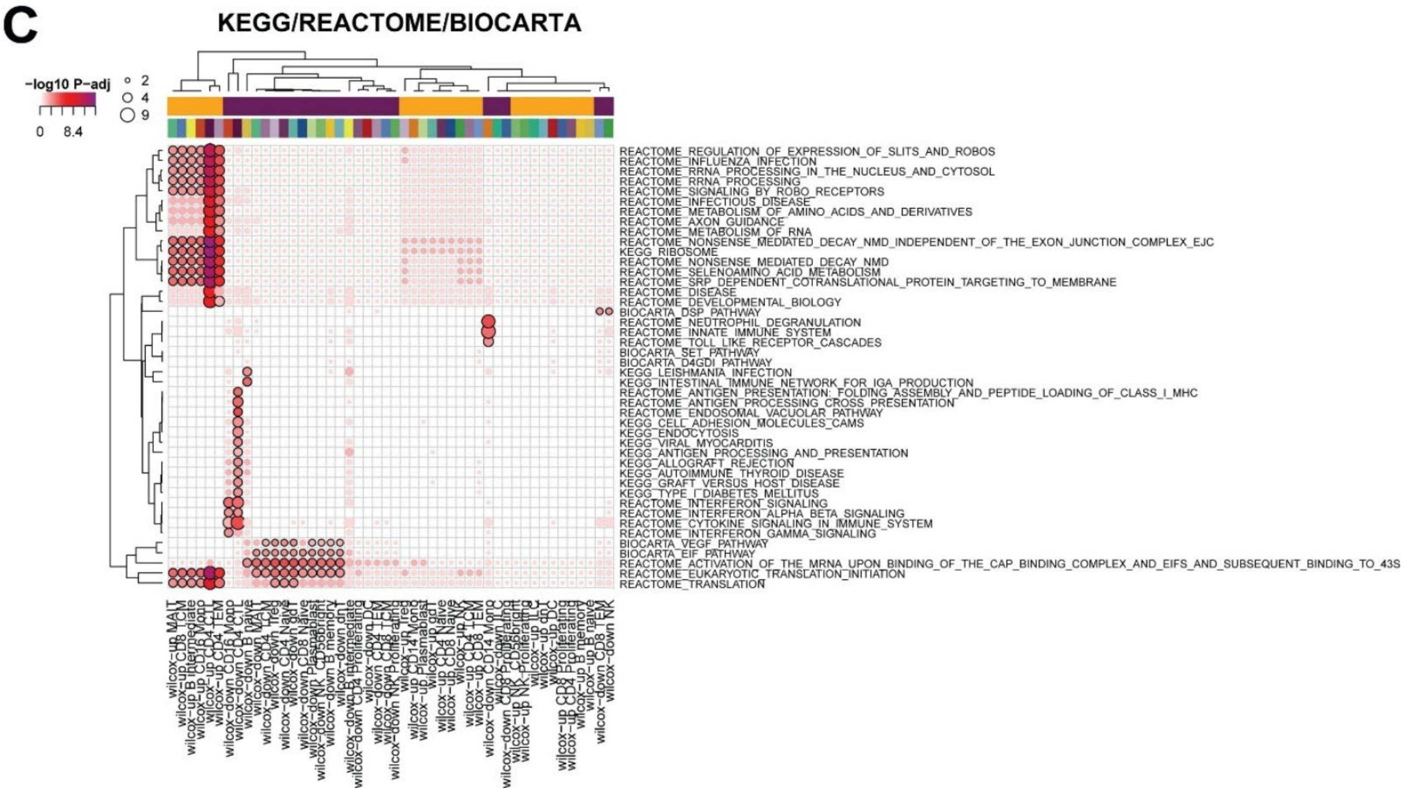

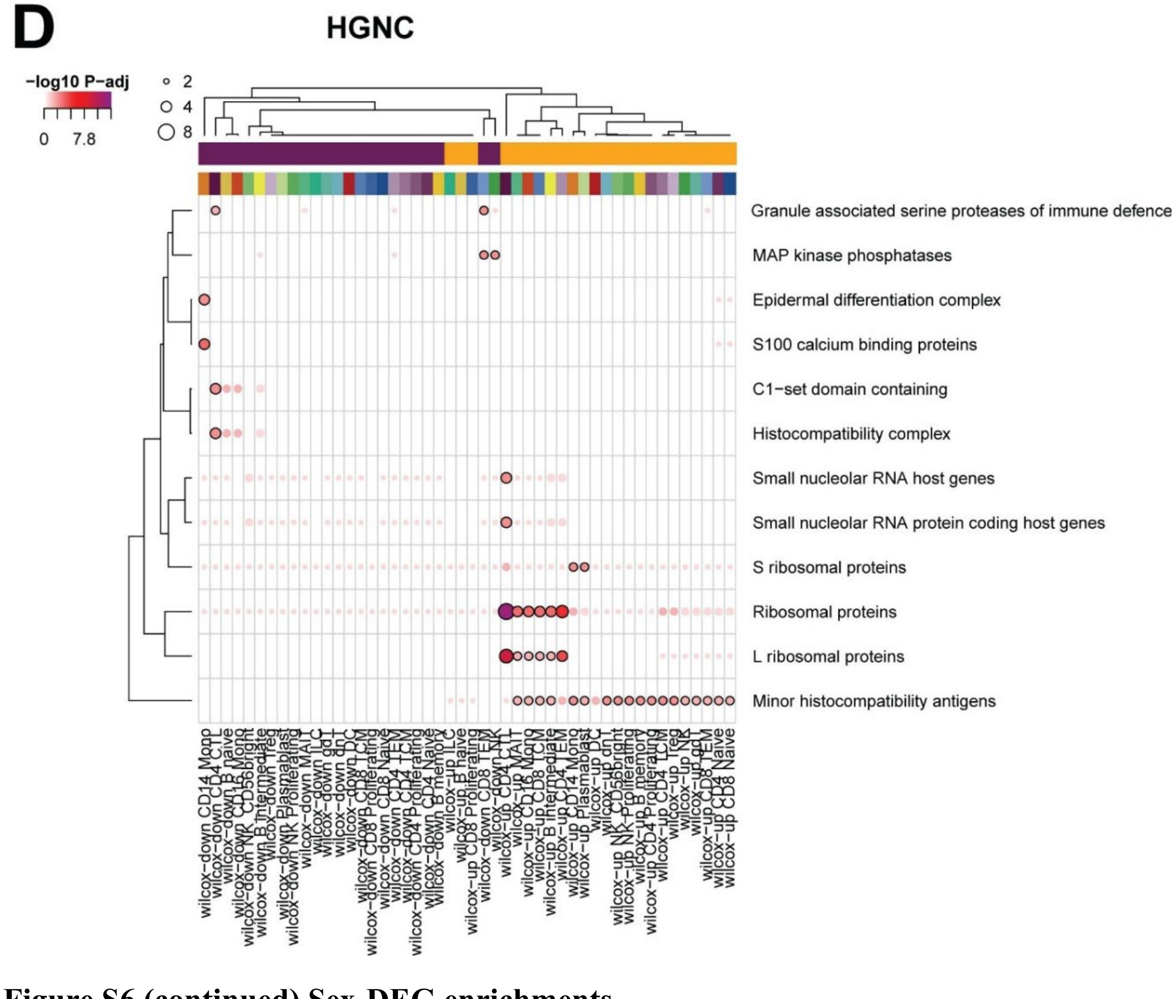

Figure S6 (continued) Sex-DEG enrichments.

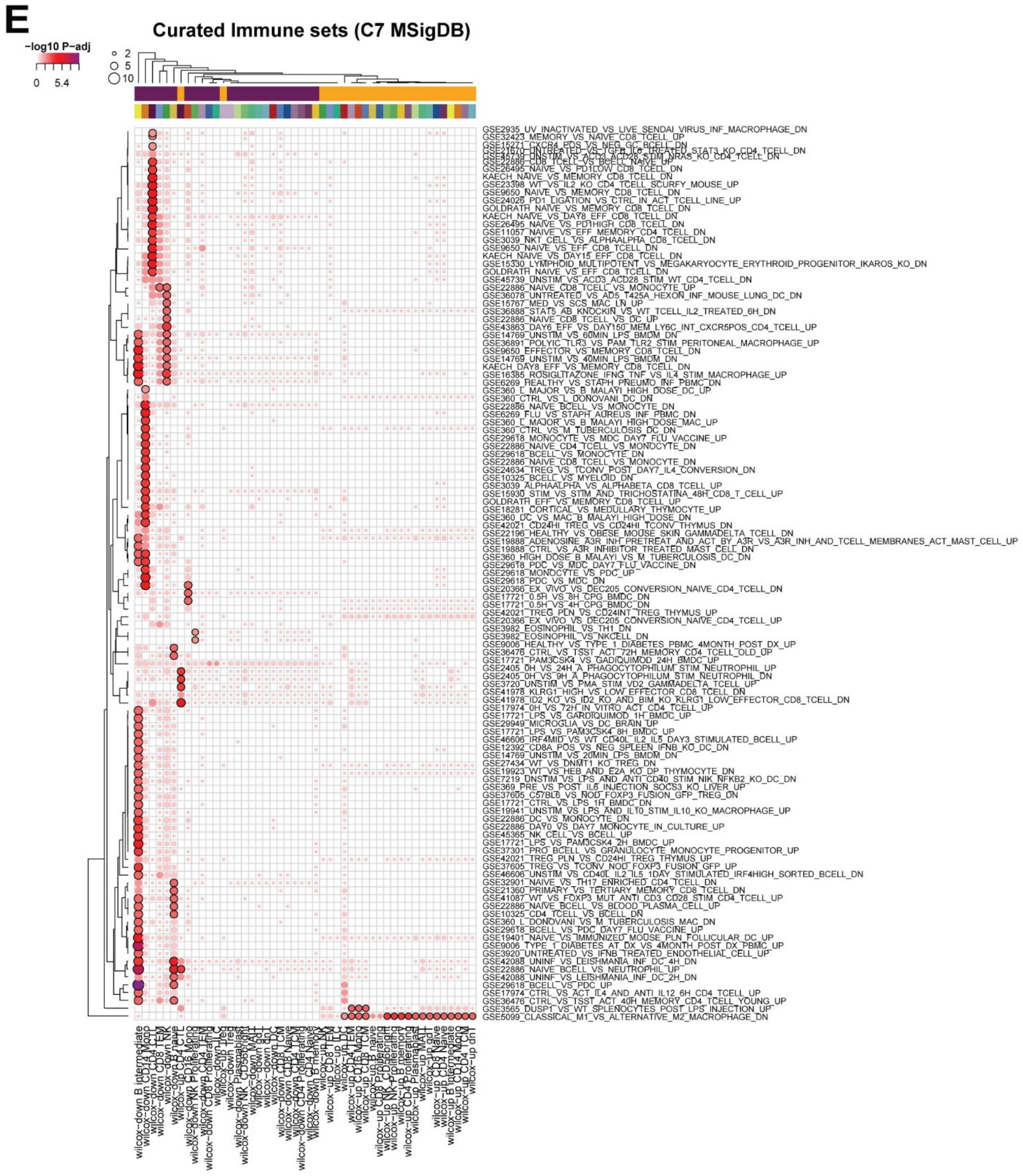

Figure S6 (continued) Sex-DEG enrichments.

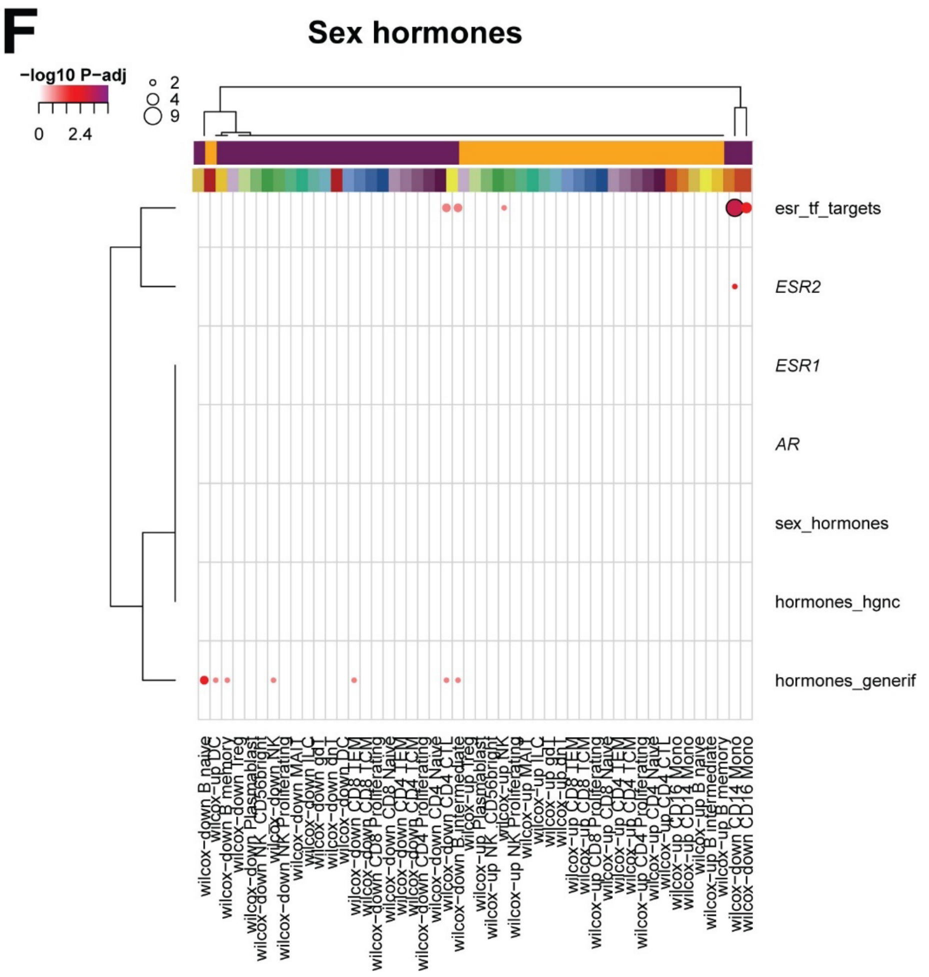

Figure S6 (continued) Sex-DEG enrichments.

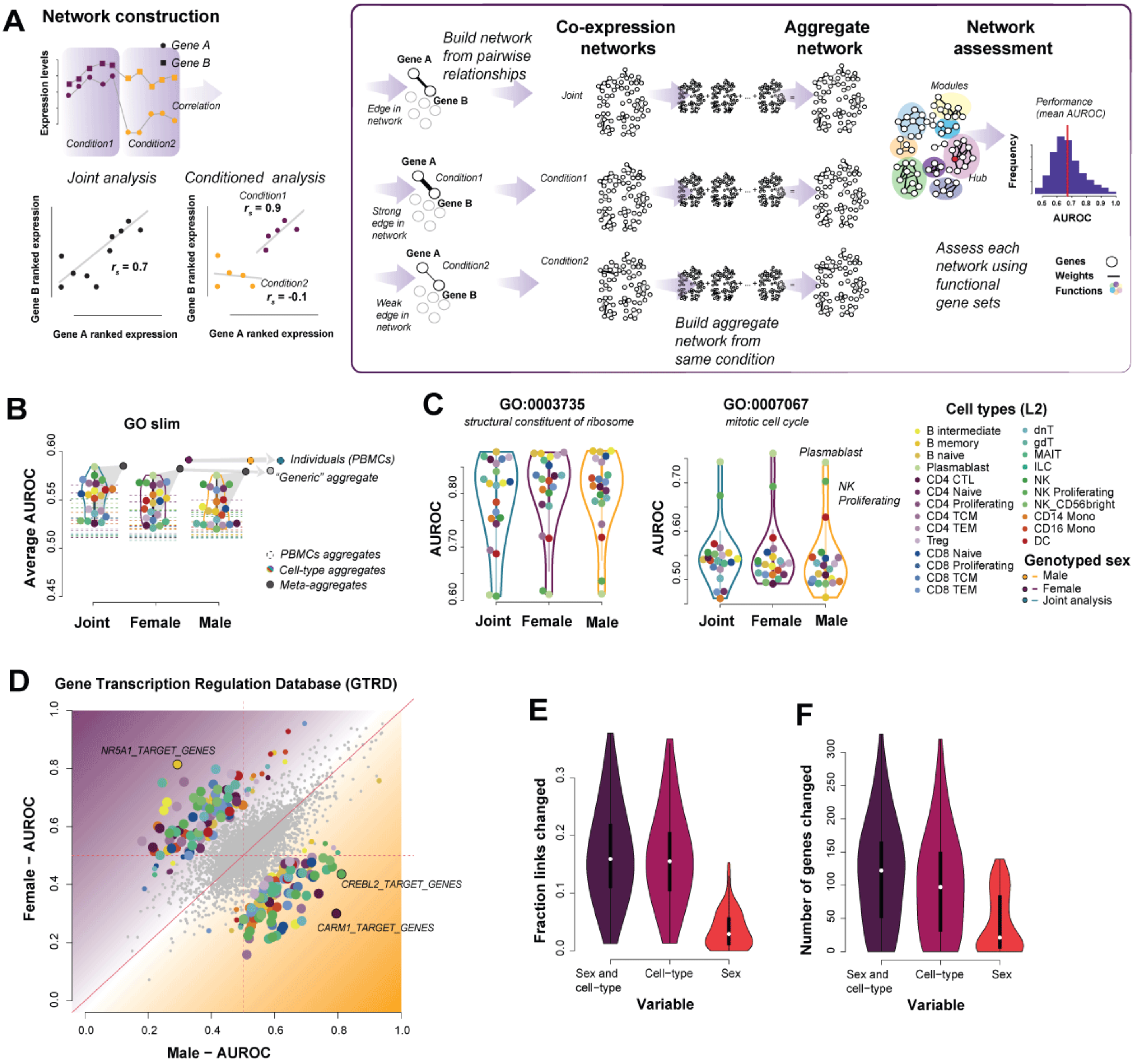

Figure S7 Co-expression by cell-type and sex. (A) Building co-expression networks and assessing performance. For each subset of cells, we calculate pairwise correlations between genes, which generates an edge in the network. This is repeated for different conditions (sex and cell-type), and these individual networks are aggregated into a final cell-type specific and sex-cell-type specific network. Enrichment of pathways and gene functions within modules formed in the network are tested through our neighbour-voting algorithm and recorded as an AUROC performance. (B) Network aggregation and performances of cell-type and sex-specific aggregates with GO slim. (C) Gene set specific performances showing differences for cell-types in ribosome and mitotic specific pathways (D) Comparing male AUROCs and female AUROCs for TF-target gene performances. Comparison of aggregate networks based on ranked expression weights (E) and node degree (F). On average, when comparing within the same cell-type but varying sex, we observe lower impacts on the edges, while comparing across cell-types within the same sex or across sex, the average number of differences are higher.

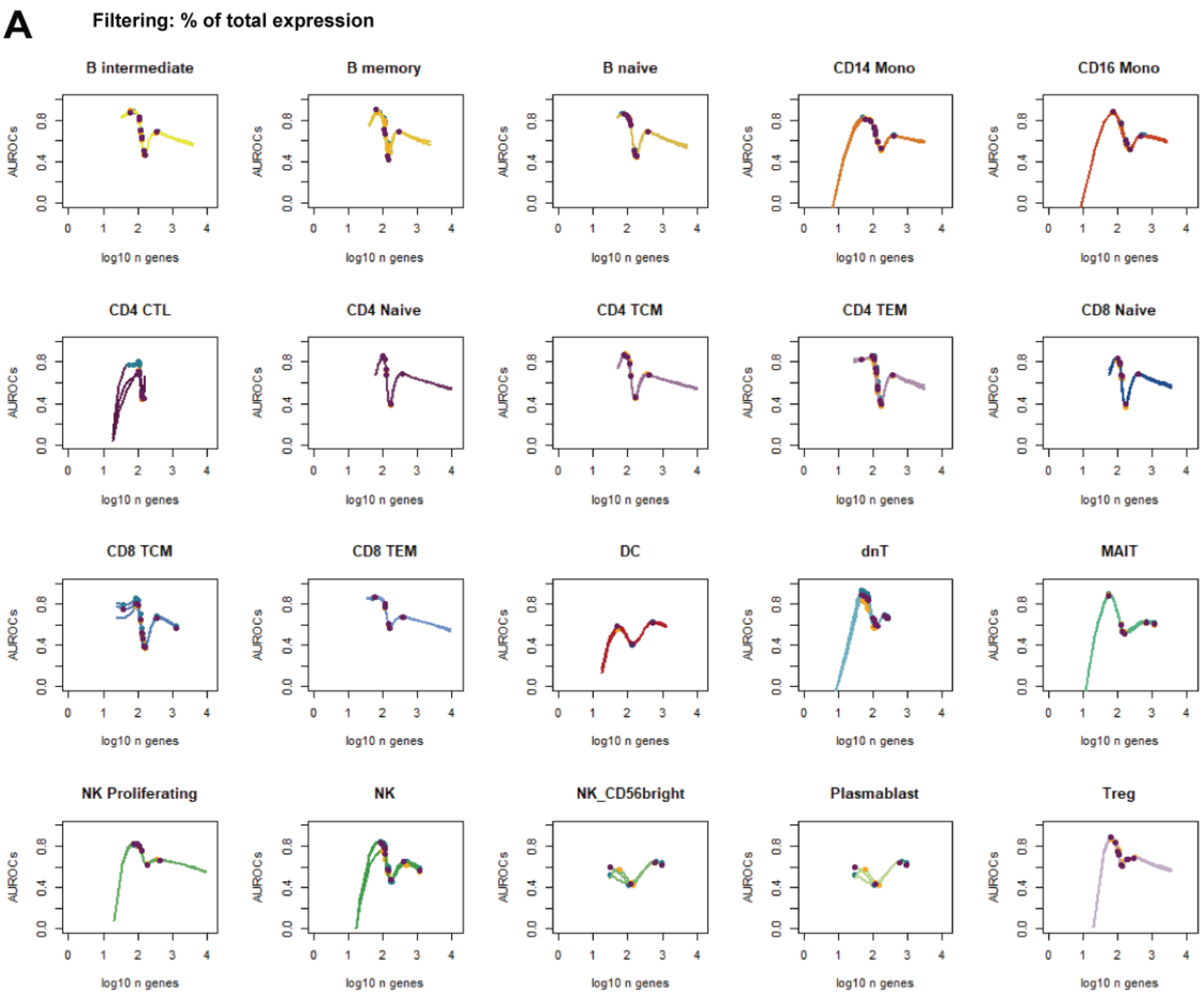

Figure S8 Aggregate co-expression downsampling comparisons. (A) For each cell-type aggregate, we filtered genes that were expressed in a fraction of the cells in that specific parameter set, (B) and those that overlapped across the sexes or joint analysis. The x-axis shows the size of the final set of genes (log10), and the y-axis the performance (AUROC) of that sub-network.

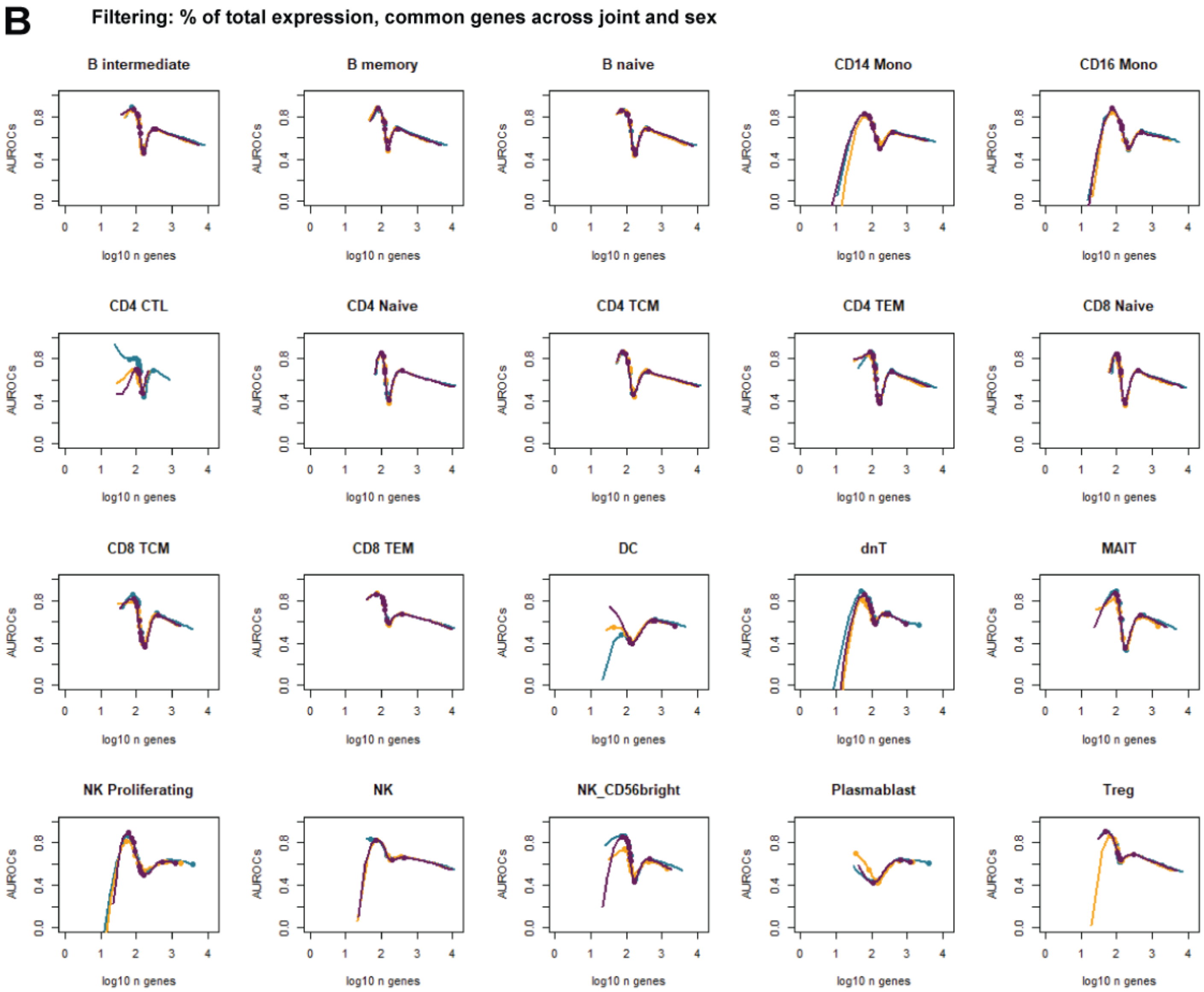

Figure S8 (continued) Aggregate co-expression downsampling comparisons.

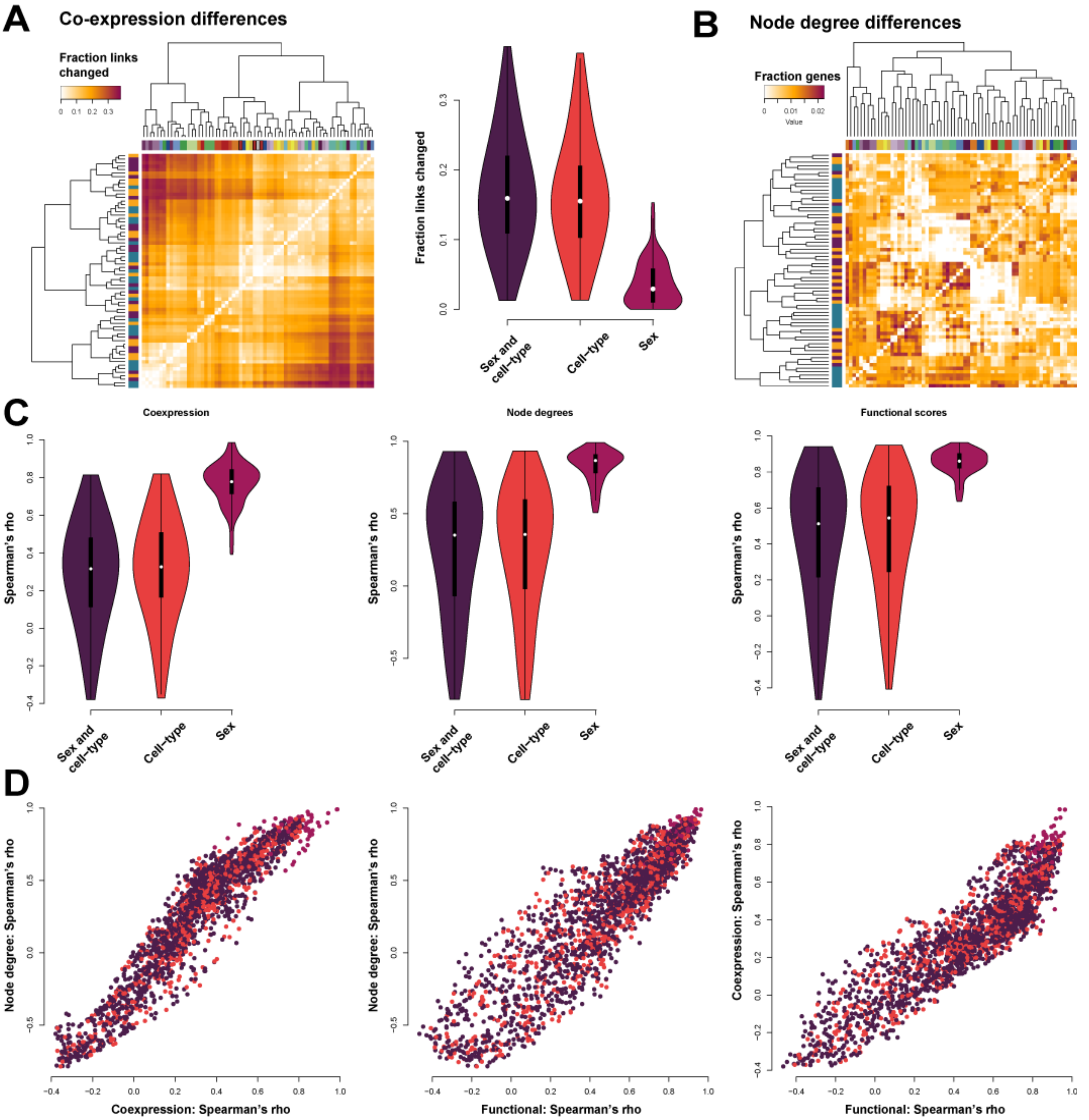

Figure S9 Differential co-expression comparisons. (A) Proportion of changed links between each aggregate network. (B) Proportion of genes with significantly different node degrees between each aggregate network. (C) Leftpanel: distribution of co-expression correlations between aggregates conditioned on sex and cell-type, cell-type only, or sex only. Middle panel: same as left panel but looking at node degrees and right-panel: network performance AUROCs for GO slim. (D) Comparing correlation values between co-expression, node degree and AUROCs.

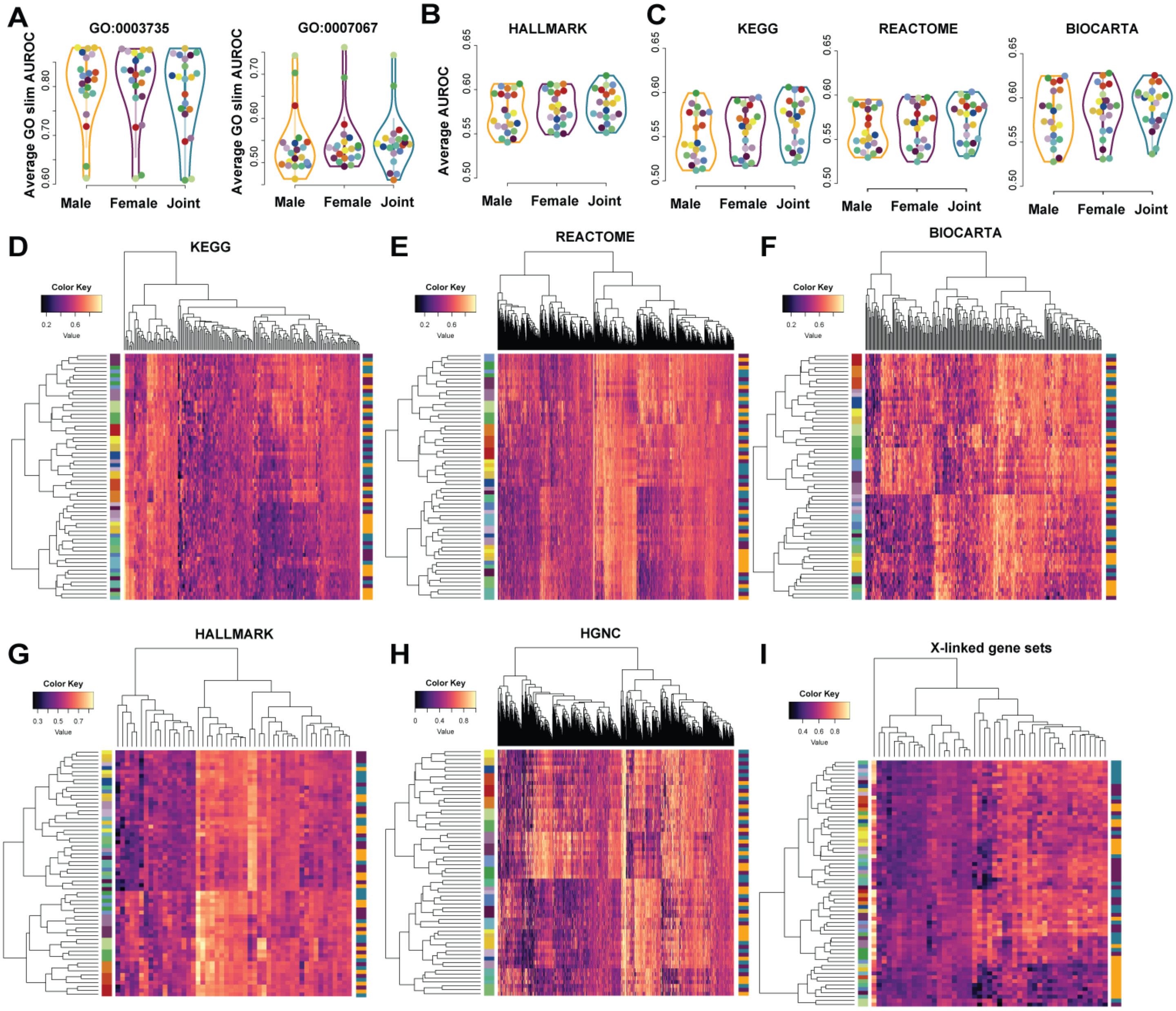

Figure S10 Functional enrichment results of aggregate networks using EGAD. (A) GO:0003735 structural constituent of ribosome example with high performances for most cell-types. (B) GO:0007067 mitotic cell cycle example with high performances in proliferating cell-types.(C) Average KEGG, REACTOME and BIOCARTA AUROCS. (D) Performance AUROCs for all KEGG, (E) REACTOME, (F) BIOCARTA, (G) HALLMARK, (H) HGNC and (I) curated X-linked gene sets and pathways.

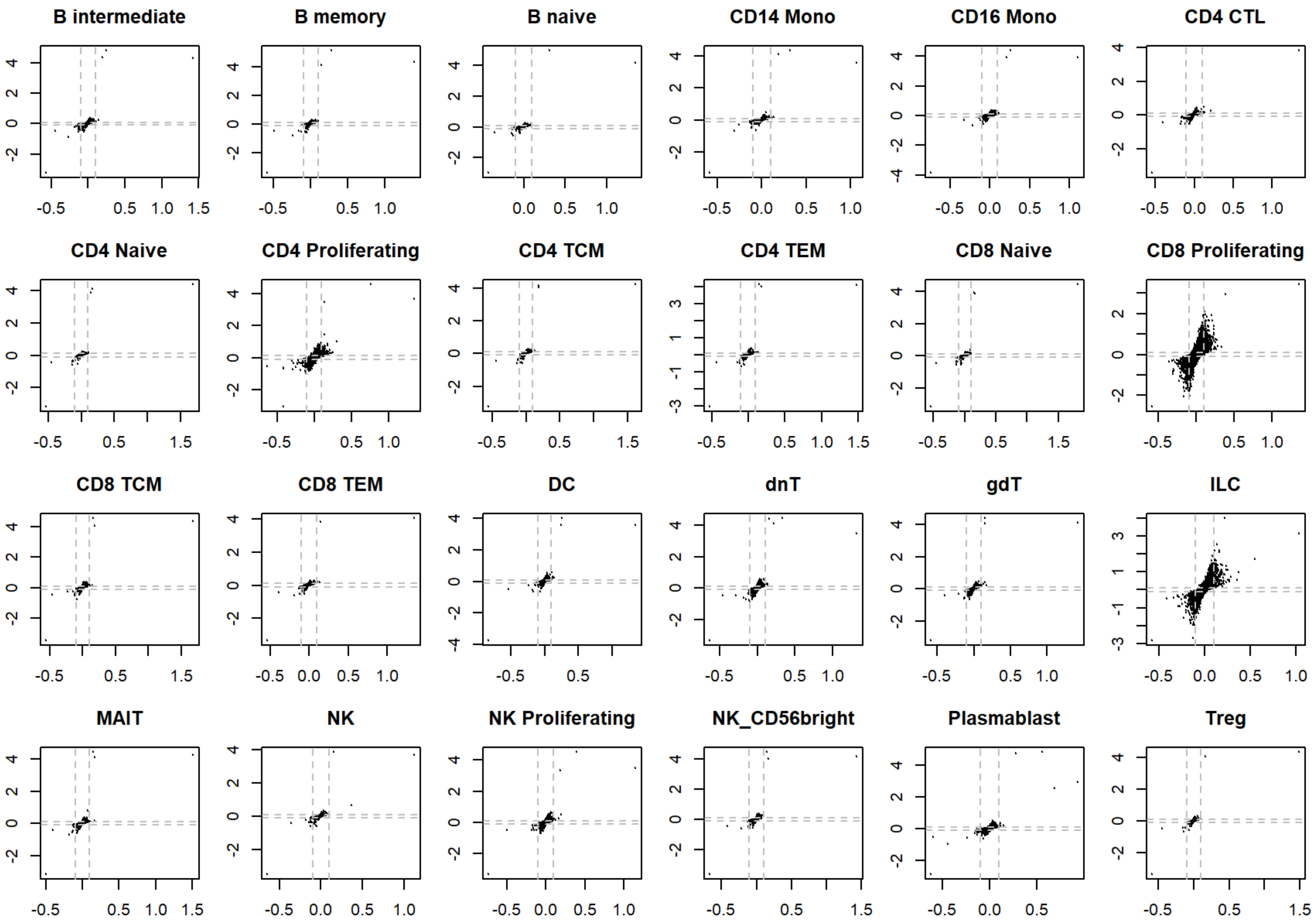

Figure S11 DEG comparison between Seurat v4 and v5: log2FC. For each cell type, the x-axis shows the log2FC from Seurat v4, and the y-axis shows the log2FC from Seurat v5. Note, only the minimal/intersect set between the two methods are shown.

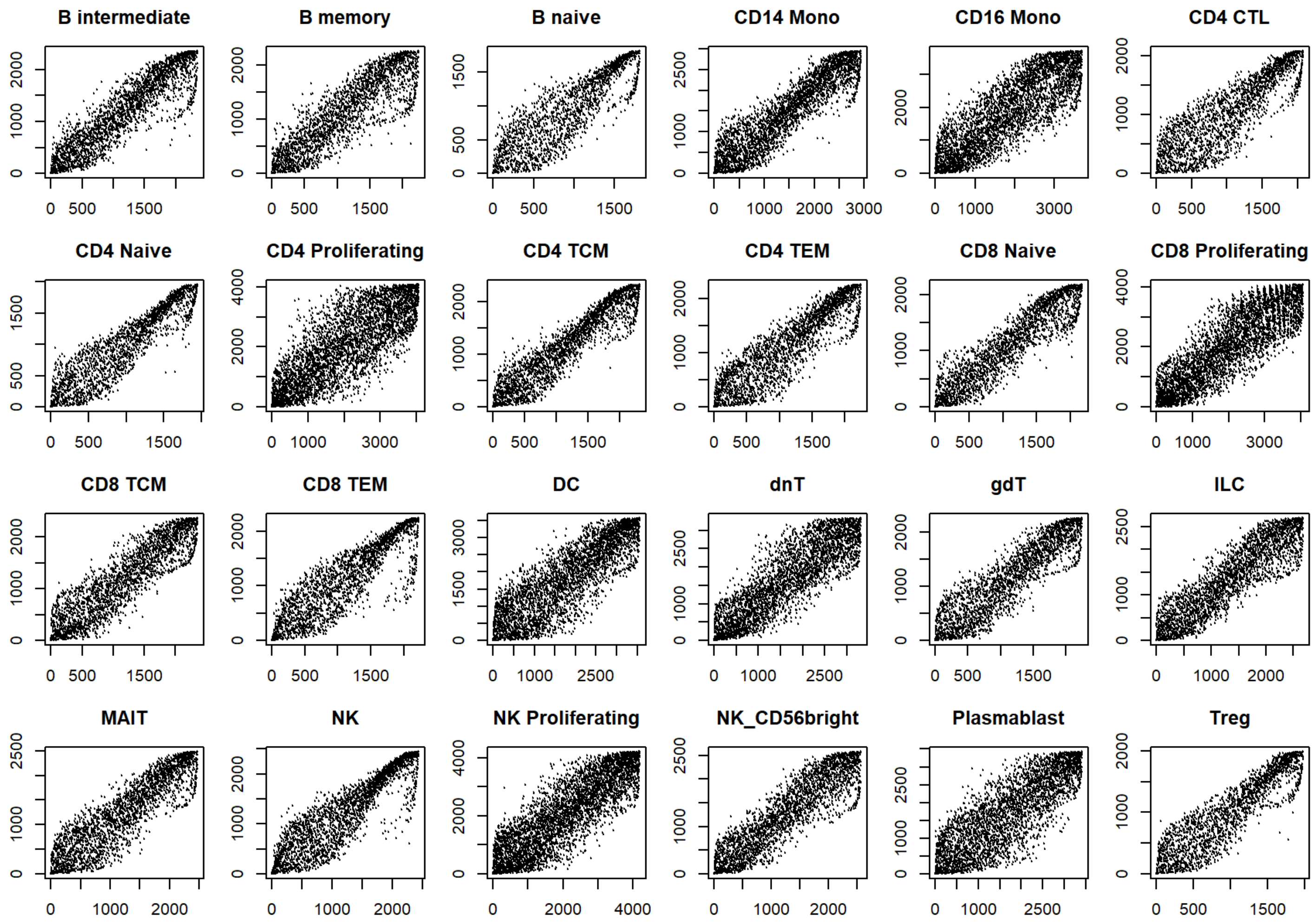

Figure S12 DEG comparison between Seurat v4 and v5: ranked log2FC. For each cell type, the x-axis shows the ranked log2FC from Seurat v4, and the y-axis shows the ranked log2FC from Seurat v5. Note, only the minimal/intersect set between the two methods are shown.

Supplemental Tables

Note:
Tables S1-4,9,14-15,17,20-21 are in the Supplemental Note
Tables S5-8,10-16,18-19, 22, 23 are in the Supplemental Spreadsheet

Table S1 Cell type counts and proportions
Table S2 Correlation of cell type proportions with age
Table S3 Correlation of cell type proportions with age – alternate models and tests
Table S4 Sex DEGs summary results per cell type and test
Table S5 List of sex DEGs per cell type
Table S6 Summary of DEG overlaps
Table S7 Downsampling DEGs statistics
Table S8 Downsampling unique counts
Table S9 Sex- specific eQTL summary results – autosomal
Table S10 Sex- specific eQTL results – autosomal – joint
Table S11 Sex- specific eQTL results – autosomal – female
Table S12 Sex- specific eQTL results – autosomal – male
Table S13 Sex- specific eQTL results – autosomal – interacting
Table S14 List of X-escape genes
Table S15 List of PAR genes
Table S16 List of MHC genes
Table S17 Sex- specific eQTL summary results – sex chromosomes
Table S18 Sex- specific eQTL results – PAR
Table S19 Sex- specific eQTL results – non-PAR
Table S20 Sex- specific eQTL and sex DEGs overlaps counts
Table S21 Sex- specific eQTL and sex DEGs overlap gene list
Table S22 Aggregate co-expression network clustering analysis
Table S23 Azimuth cell markers for L2 annotation

Table S1 Cell-type counts and proportions

	Cell-type	Cell counts			Mean proportions				
Stratification		*Total*	*Male*	*Female*	*Total*	*Male*	*Female*	FDR	Ratios
B-cells	B intermediate	33194	12440	20754	2.63%	2.40%	2.76%	1.45E-02	0.870
	B memory	23708	8582	15126	1.88%	1.63%	2.03%	9.78E-07	0.803
	B naive	71374	25965	45409	5.65%	4.98%	6.08%	2.99E-06	0.818
	Plasmablast	3602	1618	1984	0.29%	0.32%	0.28%	2.26E-01	1.169
CD4 T-cells	CD4 CTL	11680	5049	6631	0.92%	0.97%	0.92%	7.16E-01	1.056
	CD4 Naive	303555	114708	188847	24.02%	21.71%	25.35%	2.63E-06	0.856
	CD4 Proliferating	556	251	305	0.04%	0.05%	0.04%	1.62E-01	1.160
	CD4 TCM	251068	106156	144912	19.87%	20.04%	19.33%	2.26E-01	1.037
	CD4 TEM	24266	9665	14601	1.92%	1.80%	1.95%	5.14E-02	0.927
	Treg	28843	11502	17341	2.28%	2.18%	2.32%	1.45E-02	0.938
CD8 T-cells	CD8 Naive	57914	22248	35666	4.58%	4.17%	4.66%	1.32E-01	0.895
	CD8 Proliferating	256	124	132	0.02%	0.02%	0.02%	4.90E-03	1.330
	CD8 TCM	10724	4758	5966	0.85%	0.91%	0.79%	3.62E-02	1.143
	CD8 TEM	178401	79043	99358	14.12%	15.20%	13.76%	1.45E-02	1.105
Other T-cells	dnT	2939	1181	1758	0.23%	0.22%	0.24%	3.93E-01	0.946
	gdT	4883	2306	2577	0.39%	0.46%	0.35%	2.33E-01	1.320
	MAIT	12286	4511	7775	0.97%	0.85%	1.04%	3.73E-01	0.824
	ILC	315	115	200	0.02%	0.02%	0.03%	3.85E-01	0.804
Natural killer cells	NK	171757	79541	92216	13.59%	15.44%	12.65%	6.32E-08	1.221
	NK Proliferating	3215	1579	1636	0.25%	0.31%	0.22%	1.33E-08	1.365
	NK_CD56bright	8259	3112	5147	0.65%	0.60%	0.71%	3.99E-02	0.845
Monocytes	CD14 Mono	38908	18732	20176	3.08%	3.71%	2.84%	1.45E-02	1.306
	CD16 Mono	15341	7011	8330	1.21%	1.35%	1.15%	9.07E-02	1.175
Dendritic cells	DC	6596	3227	3369	0.52%	0.64%	0.48%	3.18E-06	1.324
	ASDC	*210*	*86*	*124*	*0.02%*	*0.02%*	*0.02%*	7.95E-01	*1.005*
	cDC1	*112*	*63*	*49*	*0.01%*	*0.01%*	*0.01%*	2.13E-02	*1.751*
	cDC2	*4330*	*2146*	*2184*	*0.35%*	*0.42%*	*0.31%*	4.69E-06	*1.359*
	pDC	*1944*	*932*	*1012*	*0.16%*	*0.19%*	*0.15%*	5.35E-03	*1.267*
Other	Eryth	580	232	348	*0.05%*	*0.04%*	*0.05%*	5.49E-01	*0.933*
	HSPC	1766	864	902	*0.14%*	*0.17%*	*0.12%*	8.12E-03	*1.365*
	Platelet	1773	741	1032	*0.14%*	*0.14%*	*0.14%*	5.54E-01	*1.023*
**Total**		**1265986**	**524520**	**741466**					

Table S2 Correlation of cell-type proportions with age

	Cell-type	Joint			Female			Male		
Stratification		**P-values**	**FDR**	**Rho**	**P-values**	**FDR**	**Rho**	**P-values**	**FDR**	**Rho**
B-cells	B intermediate	2.8E-08	3.9E-07	-0.18	3.8E-03	4.5E-02	-0.12	2.1E-07	4.2E-06	-0.25
	B memory	6.2E-13	1.2E-11	-0.23	4.7E-05	7.1E-04	-0.17	5.3E-10	1.2E-08	-0.30
	B naive	1.5E-03	1.5E-02	-0.10	2.7E-02	2.7E-01	-0.09	2.9E-02	3.2E-01	-0.11
	Plasmablast	4.4E-05	5.7E-04	-0.13	2.4E-04	3.1E-03	-0.15	4.7E-02	4.7E-01	-0.10
CD4 T-cells	CD4 CTL	4.4E-15	9.2E-14	0.25	5.3E-15	1.1E-13	0.32	3.0E-03	4.8E-02	0.15
	CD4 Naive	8.4E-11	1.5E-09	-0.21	4.2E-08	7.1E-07	-0.23	3.8E-04	6.8E-03	-0.17
	CD4 Proliferating	9.5E-01	1.0E+00	0.00	9.3E-01	1.0E+00	0.00	9.9E-01	1.0E+00	0.00
	CD4 TCM	7.4E-01	1.0E+00	-0.01	5.6E-01	1.0E+00	-0.02	9.4E-01	1.0E+00	0.00
	CD4 TEM	1.1E-02	8.6E-02	-0.08	3.6E-02	2.8E-01	-0.09	1.8E-01	1.0E+00	-0.07
	Treg	3.7E-02	1.9E-01	0.07	6.6E-01	1.0E+00	0.02	5.2E-03	7.8E-02	0.14
CD8 T-cells	CD8 Naive	2.9E-126	6.9E-125	-0.67	5.6E-75	1.3E-73	-0.67	4.1E-52	9.8E-51	-0.65
	CD8 Proliferating	1.1E-01	4.5E-01	0.05	2.2E-01	1.0E+00	0.05	3.4E-01	1.0E+00	0.05
	CD8 TCM	7.1E-13	1.4E-11	-0.23	1.4E-10	2.9E-09	-0.27	2.8E-04	5.4E-03	-0.18
	CD8 TEM	1.7E-09	2.7E-08	0.19	2.0E-08	3.7E-07	0.23	7.4E-03	9.6E-02	0.13
Other T-cells	dnT	4.2E-04	4.6E-03	-0.11	2.2E-02	2.5E-01	-0.10	6.6E-03	9.2E-02	-0.13
	gdT	1.0E-09	1.7E-08	-0.19	2.3E-09	4.4E-08	-0.25	1.1E-02	1.3E-01	-0.13
	MAIT	1.0E-26	2.4E-25	-0.33	4.3E-18	9.8E-17	-0.35	1.3E-09	2.7E-08	-0.29
	ILC	8.9E-03	8.0E-02	-0.08	6.4E-02	4.5E-01	-0.08	7.0E-02	6.3E-01	-0.09
Natural killer cells	NK	1.6E-24	3.6E-23	0.32	2.8E-16	6.2E-15	0.33	2.0E-10	4.7E-09	0.31
	NK Proliferating	6.3E-05	7.5E-04	0.13	7.4E-05	1.0E-03	0.17	1.4E-01	1.0E+00	0.07
	NK_CD56bright	2.3E-02	1.4E-01	-0.07	9.6E-02	5.7E-01	-0.07	1.8E-01	1.0E+00	-0.07
Monocytes	CD14 Mono	1.1E-02	8.6E-02	0.08	2.9E-02	2.7E-01	0.09	2.3E-01	1.0E+00	0.06
	CD16 Mono	1.0E-08	1.6E-07	0.18	2.0E-06	3.1E-05	0.20	1.2E-03	2.0E-02	0.16
Dendritic cells	DC	1.5E-01	4.5E-01	-0.05	4.2E-01	1.0E+00	-0.03	1.5E-01	1.0E+00	-0.07

Table S3 Correlation of cell-type proportions with age – alternate models and tests

	**F-test**			**Kruskal-Wallis**	**Beta regression**								
	Original	Covariate: age	Covariate: age + pcs		Original	Covariate: age		Covariate: age + pcs					
	sex	sex	sex	sex	sex	sex2	age	sex2	age	pc1	pc2	pc3	pc4
B intermediate	1.5E-02	1.7E-02	1.9E-02	2.5E-02	4.6E-02	4.2E-02	4.8E-04	1.2E-01	1.43E-03	0.92	1.00	0.88	0.88
B memory	9.8E-07	9.0E-07	1.2E-06	9.8E-07	2.2E-06	1.2E-06	1.0E-11	3.7E-06	3.00E-11	0.47	0.98	1.00	0.82
B naive	3.0E-06	2.9E-06	3.2E-06	6.4E-06	1.0E-05	7.3E-06	7.3E-03	2.2E-05	2.19E-02	0.20	0.99	0.96	0.69
CD14 Mono	1.5E-02	1.6E-02	1.9E-02	1.1E-02	3.0E-02	2.5E-02	6.2E-02	7.3E-02	1.60E-01	0.38	0.65	0.69	0.60
CD16 Mono	9.1E-02	1.1E-01	1.2E-01	4.8E-02	1.1E-01	1.0E-01	1.3E-08	2.4E-01	3.86E-08	0.30	0.92	0.16	1.00
CD4 CTL	7.2E-01	6.0E-01	5.9E-01	5.6E-01	5.8E-01	5.9E-01	3.8E-12	8.8E-01	1.13E-11	0.94	0.29	0.82	1.00
CD4 Naive	2.6E-06	2.7E-06	3.2E-06	2.8E-06	2.2E-06	1.2E-06	3.1E-10	3.7E-06	9.42E-10	1.00	0.10	0.64	0.82
CD4 Proliferating	1.6E-01	1.7E-01	1.9E-01	4.5E-01	3.4E-01	6.7E-01	6.7E-01	8.8E-01	8.86E-01	0.82	0.69	1.00	0.05
CD4 TCM	2.3E-01	2.2E-01	2.5E-01	2.2E-01	2.1E-01	2.0E-01	4.4E-01	4.3E-01	7.67E-01	0.16	0.88	0.87	0.68
CD4 TEM	5.1E-02	5.4E-02	5.4E-02	4.3E-02	6.3E-02	5.9E-02	2.7E-01	1.6E-01	5.64E-01	0.88	1.00	0.64	1.00
CD8 Naive	1.3E-01	1.1E-01	1.3E-01	5.0E-02	1.5E-01	1.4E-01	5.7E-131	3.2E-01	1.70E-130	0.17	1.00	0.92	1.00
CD8 Proliferating	4.9E-03	5.5E-03	4.9E-03	1.2E-02	2.0E-02	1.6E-02	2.2E-01	4.8E-02	4.72E-01	0.70	0.83	0.88	0.81
CD8 TCM	3.6E-02	2.2E-02	2.5E-02	4.3E-02	4.0E-02	3.6E-02	5.9E-10	1.0E-01	1.78E-09	0.70	0.88	0.92	0.88
CD8 TEM	1.5E-02	1.6E-02	1.9E-02	1.7E-02	2.0E-02	1.7E-02	1.3E-08	4.9E-02	3.76E-08	1.00	0.88	0.28	1.00
DC	3.2E-06	2.9E-06	3.2E-06	1.3E-06	4.4E-06	2.8E-06	3.7E-01	8.3E-06	6.87E-01	0.88	1.00	0.64	1.00
dnT	3.9E-01	4.3E-01	4.2E-01	4.4E-01	3.4E-01	3.6E-01	3.4E-05	6.8E-01	1.02E-04	0.98	0.79	0.98	0.56
gdT	2.3E-01	1.8E-01	1.9E-01	2.2E-01	3.4E-01	3.6E-01	2.2E-11	6.8E-01	6.57E-11	0.98	0.93	0.88	0.88
ILC	3.9E-01	4.2E-01	4.2E-01	7.2E-02	1.2E-01	1.2E-01	1.9E-02	2.8E-01	5.34E-02	0.92	0.82	1.00	1.00
MAIT	3.7E-01	4.3E-01	4.3E-01	1.4E-01	3.0E-01	2.9E-01	1.6E-27	6.0E-01	4.78E-27	0.38	1.00	0.88	0.88
NK	6.3E-08	3.2E-08	2.7E-08	1.0E-07	1.2E-07	3.9E-08	2.7E-20	1.2E-07	8.14E-20	0.82	0.91	0.88	0.77
NK Proliferating	1.3E-08	1.5E-08	1.7E-08	1.0E-07	3.2E-08	7.2E-09	6.2E-02	2.2E-08	1.60E-01	0.94	0.34	0.84	1.00
NK_CD56bright	4.0E-02	4.3E-02	4.7E-02	1.1E-02	2.0E-02	1.7E-02	1.3E-01	4.9E-02	3.00E-01	0.77	0.44	0.64	0.88
Plasmablast	2.3E-01	1.8E-01	1.9E-01	3.6E-01	3.0E-01	2.9E-01	1.5E-06	6.0E-01	4.50E-06	0.88	0.82	0.92	0.88
Treg	1.5E-02	1.6E-02	1.4E-02	1.2E-02	2.0E-02	1.7E-02	4.9E-02	4.9E-02	1.37E-01	0.15	0.23	1.00	0.38

Table S4 Sex DEGs summary results per cell-type and test

**Cell-type**	**Wilcoxon-test**	**MAST**	**MAST - age adjusted**

Stratification		Male	X	Y	Female	X	**Total**	Male	X	Y	Female	X	**Total**	Male	X	Y	Female	X	**Total**
B-cells	B intermediate	5	1	3	28	6	33	5	1	3	28	6	33	5	1	3	28	6	33
	B memory	3	0	3	7	4	10	3	0	3	7	4	10	3	0	3	7	4	10
	B naive	2	0	2	13	5	15	2	0	2	13	5	15	2	0	2	13	5	15
	Plasmablast	5	1	3	7	5	12	5	1	3	7	5	12	5	1	3	7	5	12
CD4 T-cells	CD4 CTL	8	2	1	19	5	27	8	2	1	19	5	27	8	2	1	19	5	27
	CD4 Naive	5	0	3	5	5	10	5	0	3	5	5	10	5	0	3	5	5	10
	CD4 Proliferating	3	0	3	4	4	7	3	0	3	4	4	7	3	0	3	4	4	7
	CD4 TCM	4	0	3	6	5	10	4	0	3	6	5	10	4	0	3	6	5	10
	CD4 TEM	6	1	3	7	4	13	6	1	3	7	4	13	6	1	3	7	4	13
	Treg	3	0	2	5	5	8	3	0	2	5	5	8	3	0	2	5	5	8
CD8 T-cells	CD8 Naive	5	0	3	8	5	13	5	0	3	8	5	13	5	0	3	8	5	13
	CD8 Proliferating	2	0	2	2	2	4	2	0	2	2	2	4	2	0	2	2	2	4
	CD8 TCM	5	0	3	6	4	11	5	0	3	6	4	11	5	0	3	6	4	11
	CD8 TEM	4	0	2	10	3	14	4	0	2	10	3	14	4	0	2	10	3	14
Other T-cells	dnT	4	0	4	7	7	11	4	0	4	7	7	11	4	0	4	7	7	11
	gdT	5	0	3	5	4	10	5	0	3	5	4	10	5	0	3	5	4	10
	MAIT	5	1	3	9	4	14	5	1	3	9	4	14	5	1	3	9	4	14
	ILC	1	0	1	2	2	3	1	0	1	2	2	3	1	0	1	2	2	3
Natural killer cells	NK	4	0	2	16	3	20	4	0	2	16	3	20	4	0	2	16	3	20
	NK Proliferating	3	0	3	6	5	9	3	0	3	6	5	9	3	0	3	6	5	9
	NK_CD56bright	3	0	3	7	4	10	3	0	3	7	4	10	3	0	3	7	4	10
Monocytes	CD14 Mono	5	0	3	21	3	26	5	0	3	21	3	26	5	0	3	21	3	26
	CD16 Mono	5	0	3	12	2	17	5	0	3	12	2	17	5	0	3	12	2	17
Dendritic cells	DC	7	1	3	5	4	12	7	1	3	5	4	12	7	1	3	5	4	12
Other	Eryth	1	0	1	2	2	3	1	0	1	2	2	3	1	0	1	2	2	3
	HSPC	3	0	3	2	2	5	3	0	3	2	2	5	3	0	3	2	2	5
	Platelet	3	0	2	3	2	6	3	0	2	3	2	6	3	0	2	3	2	6
**Average**		4.0	0.3	2.6	8.3	3.9	12.3	4.0	0.3	2.6	8.3	3.9	12.3	4.0	0.3	2.6	8.3	3.9	12.3

Table S9 Sex- specific eQTL summary results – autosomal

	Cell-type	Total* eQTLs			Novel**			Interacting
Stratification		Joint	Female	Male	Female-specific analysis only	Male-specific analysis only	Joint analysis only	
B-cells	B intermediate	433	148	120	21	18	433	0
	B memory	365	127	102	19	18	365	3
	B naive	856	365	249	56	39	856	5
	Plasmablast	49	18	12	3	2	49	1
CD4 T-cells	CD4 CTL	62	28	30	5	5	62	4
	CD4 Naive	2824	1554	1249	263	208	2824	9
	CD4 TCM	2845	1389	1335	227	227.	2845	1
	CD4 TEM	306	94	82	16	14	306	4
	Treg	395	150	129	23	22	395	0
CD8 T-cells	CD8 Naive	628	280	202	48	32	628	1
	CD8 TCM	125	48	41	7	7	125	1
	CD8 TEM	1782	775	778	120	128	1782	8
Other T-cells	dnT	24	10	8	2	1	24	0
	gdT	34	12	16	2	3	34	2
	MAIT	143	45	48	8	1	143	0
Natural killer cells	NK	2264	936	1056	144	171	2264	4
	NK Proliferating	47	14	14	2	2	47	1
	NK_CD56bright	135	51	42	8	6	135	0
Monocytes	CD14 Mono	546	186	217	29	35	509	3
	CD16 Mono	464	158	207	24	33	445	4
Dendritic cells	DC	161	36	65	3	10	161	0
Total		14488	6424	6002	1038	990	14432	51

*** After cell type specific FDR correction (local FDR <0.05)**

****After global multiple testing correction (global q-value<0.05)**

Table S17 Sex- specific eQTL summary results – sex chromosomes

Stratification	Celltype	PAR1 Joint	PAR1 Female	PAR1 Male	PAR2 Joint	PAR2 Female	PAR2 Male	PAR Joint	PAR Female	PAR Male	non-PAR Joint	non-PAR Female	non-PAR Male	Escapees Female	Total Joint	Total Female	Total Male
B-cells	B intermediate	0	0	0	0	0	0	0	0	0	4	0	0	0	4	0	0
	B memory	0	0	0	0	0	0	0	0	0	1	0	0	0	1	0	0
	B naive	2	1	0	1	0	0	3	1	0	16	2	4	1	19	3	4
	Plasmablast	0	0	0	1	0	0	1	0	0	3	2	1	0	4	2	1
CD4 T-cells	CD4 CTL	0	0	0	0	0	0	0	0	0	1	1	1	0	1	1	1
	CD4 Naive	2	1	1	0	0	0	2	1	1	38	26	25	1	40	27	26
	CD4 TCM	2	2	1	0	0	0	2	2	1	57	3	36	1	59	5	37
	CD4 TEM	2	1	1	0	0	0	2	1	1	18	22	2	5	20	23	3
	Treg	1	2	1	0	0	0	1	2	1	12	4	4	0	13	6	5
CD8 T-cells	CD8 Naive	2	0	0	0	0	0	2	0	0	17	4	4	0	19	4	4
	CD8 TCM	0	0	0	0	0	0	0	0	0	5	1	1	0	5	1	1
	CD8 TEM	1	0	0	1	0	0	2	0	0	34	13	14	1	36	13	14
Other T-cells	dnT	0	0	0	0	0	0	0	0	0	0	0	0	0	0	0	0
	gdT	0	0	0	1	0	0	1	0	0	3	1	1	0	4	1	1
	MAIT	0	0	0	0	0	0	0	0	0	3	2	1	0	3	2	1
Natural killer cells	NK	1	1	0	1	0	0	2	1	0	36	15	27	3	38	16	27
	NK Proliferating	0	0	0	0	0	0	0	0	0	1	0	2	0	1	0	2
	NK_CD56bright	0	0	0	0	0	0	0	0	0	1	0	0	0	1	0	0
Monocytes	CD14 Mono	1	0	0	0	0	0	1	0	0	12	1	1	1	13	1	1
	CD16 Mono	0	0	0	1	0	0	1	0	0	5	0	0	0	6	0	0
Dendritic cells	DC	0	0	0	0	0	0	0	0	0	0	0	0	3	0	0	0
Total		14	8	4	6	0	0	20	8	4	267	97	124	16	287	105	128

Table S20 Sex- specific eQTL and sex DEGs overlaps counts

	**Autosomal eQTLs**						**Sex chromosome eQTLs**				
	**Female-biased DEGs**			**Male-biased DEGs**			**Female-biased DEGs**			**Male-biased DEGs**	
	Female	*Joint*	*Interacting*	*Male*	*Joint*	*Interacting*	*Female X*	*Female X (including escape)*	*Joint X*	*Male XY*	*Joint X*
B intermediate	0	2	0	0	0	0	0	0	0	0	0
B memory	0	0	0	0	0	0	0	0	0	0	0
B naive	0	0	0	0	0	0	0	1	0	0	0
Plasmablast	0	0	0	0	0	0	0	0	0	0	0
CD4 CTL	0	0	0	0	0	0	0	0	0	0	0
CD4 Naive	0	0	0	0	0	0	1	2	0	0	0
CD4 TCM	0	0	0	0	0	0	1	2	1	0	0
CD4 TEM	0	1	0	0	0	0	0	0	0	0	0
Treg	0	0	0	0	0	0	0	1	0	0	0
CD8 Naive	0	0	0	0	0	0	0	0	0	0	0
CD8 TCM	0	0	0	0	0	0	0	0	0	0	0
CD8 TEM	0	2	0	0	0	0	0	0	0	0	0
dnT	0	0	0	0	0	0	0	0	0	0	0
gdT	0	0	0	0	0	0	0	0	0	0	0
MAIT	0	0	0	0	0	0	0	0	0	0	0
NK	1	6	0	0	1	0	0	0	0	0	0
NK Proliferating	0	1	0	0	0	0	0	0	0	0	0
NK_CD56bright	0	1	0	0	0	0	0	0	0	0	0
CD14 Mono	0	4	1	0	0	0	0	0	0	0	0
CD16 Mono	0	2	0	0	0	0	0	0	0	0	0
DC	0	0	0	0	0	0	0	1	0	0	0
**Totals**	1	19	1	0	1	0	2	7	1	0	0

Table S21 Sex- specific eQTL and sex DEGs overlap gene list

	Cell type	Gene	rsID		eQTL	DEG
**Autosomal**	B_intermediate	*CD83*	rs16874672	6:14087484_T	Joint	female biased expression
	B_intermediate	*CIB1*	rs9745131	15:90776154_T	Joint	female biased expression
	CD14_Mono	*CSTA*	rs67931976	3:122105715_G	Joint	female biased expression
	CD14_Mono	*ITGB2*	rs760462	21:46328099_C	Joint	female biased expression
	CD14_Mono	*LGALS2*	rs7291467	22:37972628_A	Joint	female biased expression
	CD14_Mono	*S100A8*	rs60752752	1:153339782_G	Joint	female biased expression
	CD16_Mono	*ANXA1*	rs10124476	9:75889773_C	Joint	female biased expression
	CD16_Mono	*IFITM2*	rs11246057	11:295808_G	Joint	female biased expression
	CD4_TEM	*GZMK*	rs7714681	5:54291899_T	Joint	female biased expression
	CD8_TEM	*GZMA*	rs2047745	5:54489541_C	Joint	female biased expression
	CD8_TEM	*HOPX*	rs35867715	4:57627999_C	Joint	female biased expression
	NK	*FCER1G*	rs11421	1:161188936_C	Joint	female biased expression
	NK	*FCGR3A*	rs6700241	1:161500975_G	Joint	female biased expression
	NK	*GZMA*	rs62361890	5:55052519_A	Joint	female biased expression
	NK	*HOPX*	rs4865137	4:57525669_C	Joint	female biased expression
	NK	*IFITM2*	rs1059091	11:309127_G	Joint	female biased expression
	NK	*PTGDS*	rs2271869	9:139848273_G	Joint	**male biased expression**
	NK	*XCL2*	rs141673522	1:168517508_T	Joint	female biased expression
	NK_CD56bright	*DONSON*	rs11088258	21:35034050_A	Joint	female biased expression
	NK_Proliferating	*LAIR2*	rs73070113	19:55014172_T	Joint	female biased expression
	NK	*FCGR3A*	rs2099684	1:161500130_G	Female	female biased expression
	CD14_Mono	*ITGB2*	rs760462	21:46328099_C	Interacting	female biased expression
**Sex chromosomes**						
	CD4_Naive	*SEPT6*		X:119505884_G	Female	female biased expression
	CD4_TCM	*SEPT6*		X:117782171_A	Female	female biased expression
**Escape genes**						
	B_naive	*RPS4X*		X:71468142_G	Female	female biased expression
	CD4_Naive	*EIF2S3*		X:24130140_C	Female	female biased expression
	CD4_TCM	*EIF2S3*		X:24419789_G	Female	female biased expression
	DC	*XIST*		X:73702119_C	Female	female biased expression
	Treg	*XIST*		X:72050435_C	Female	female biased expression

Supplemental Methods

Co-expression network generation

For each batch, we generated individual expression matrices using the normalized count data for each cell-type and sex. We used the most recurrent genes across all the data to ensure a common gene reference, totalling 14,716 genes. Then, we built a gene co-expression network from each of these expression matrices, using Spearman's correlation to measure gene-gene relationships. Due to low cell-numbers for some cell-types, we built networks for 21 of the 27 cell-types. Each network was then ranked to standardize the correlations. We then aggregated the same cell-type per sex to build meta-analytic co-expression networks. We also built individual cell-type specific networks that were not conditioned on sex, and similarly aggregated these networks (labelled "joint"). For additional analyses, we then built "PBMC" aggregates from the cell-type aggregates, for the joint and individual sexes. Finally, we built networks from each individual, removing Erythrocytes, HSPC and Platelets. We aggregated individuals by their genotyped sex. Overall, we generated 70 aggregate networks.

Differential co-expression analysis

To compare across networks, we assessed network differences based on overall topology, node degrees and functional performances. For each network, we took the correlation of the matrices as a measure of topological similarity. We calculated the node degrees of each network by summing the weights of each node and then took the correlation of these node degrees. And finally, using the outputs from the GO slim analysis, we calculated the similarity of their performances by taking the correlation of the AUROCs of the functional groups. To identify the changes by genes and gene modules, we calculated the frequency of genes whose co-expression ranking changed by at least 0.5 between the networks. With these tallies, we identified which genes had the most topological shifts. Secondly, we repeated this with the node degree of the genes, calculating standardised residuals and selecting outliers that were three standard deviations away.

Differential expression analysis - globalFDR

For an additional analysis, we calculated a global FDR. We calculated a gene-level P-value using the Simes procedure on the raw p-values across all cell types where the gene was expressed. For genes not expressed within that cell type, we used a value of 1. We then performed global multiple testing correction across all genes using the Benjamini-Hochberg (BH) method. We filtered for significance using the global FDR of 0.05 and the cell type specific $|\log2FC| > 0.1$.

Downsampling differential expression analysis

For each cell-type, we downsampled the number of cells (repeated) and ran the differential expression analysis using the FindMarkers function. With this we calculated the number of significant genes and the number of genes that overlap with the full set of cells for that cell-type.

Supplemental References

1. Klein, S.L., and Flanagan, K.L. (2016). Sex differences in immune responses. Nat Rev Immunol 16, 626-638.
2. Márquez, E.J., Chung, C.-H., Marches, R., Rossi, R.J., Nehar-Belaid, D., Eroglu, A., Mellert, D.J., Kuchel, G.A., Banchereau, J., and Ucar, D. (2020). Sexual-dimorphism in human immune system aging. Nat Commun 11, 751.
3. Taneja, V. (2018). Sex Hormones Determine Immune Response. Front Immunol 9, 1931.
4. Mitchell, E., Spencer Chapman, M., Williams, N., Dawson, K.J., Mende, N., Calderbank, E.F., Jung, H., Mitchell, T., Coorens, T.H.H., Spencer, D.H., et al. (2022). Clonal dynamics of haematopoiesis across the human lifespan. Nature 606, 343-350.
5. Hazeldine, J., and Lord, J.M. (2013). The impact of ageing on natural killer cell function and potential consequences for health in older adults. Ageing Res Rev 12, 1069-1078.
6. Chen, G., Lustig, A., and Weng, N.-P. (2013). T Cell Aging: A Review of the Transcriptional Changes Determined from Genome-Wide Analysis. Front Immunol 0.
7. Churov, A.V., Mamashov, K.Y., and Novitskaia, A.V. (2020). Homeostasis and the functional roles of CD4+ Treg cells in aging. Immunology Letters 226, 83-89.
8. Ballouz, S., Verleyen, W., and Gillis, J. (2015). Guidance for RNA-seq co-expression network construction and analysis: safety in numbers. Bioinformatics 31, 2123-2130.
9. Ballouz, S., Weber, M., Pavlidis, P., and Gillis, J. (2017). EGAD: ultra-fast functional analysis of gene networks. Bioinformatics 33, 612-614.
10. Ashburner, M., Ball, C.A., Blake, J.A., Botstein, D., Butler, H., Cherry, J.M., Davis, A.P., Dolinski, K., Dwight, S.S., Eppig, J.T., et al. (2000). Gene ontology: tool for the unification of biology. The Gene Ontology Consortium. Nat Genet 25, 25-29.
11. Gene Ontology, C. (2021). The Gene Ontology resource: enriching a GOld mine. Nucleic Acids Res 49, D325-D334.
12. Farahbod, M., and Pavlidis, P. (2020). Untangling the effects of cellular composition on coexpression analysis. Genome Res 30, 849-859.
13. Subramanian, A., Tamayo, P., Mootha, V.K., Mukherjee, S., Ebert, B.L., Gillette, M.A., Paulovich, A., Pomeroy, S.L., Golub, T.R., Lander, E.S., et al. (2005). Gene set enrichment analysis: a knowledge-based approach for interpreting genome-wide expression profiles. Proc Natl Acad Sci U S A 102, 15545-15550.
14. Liberzon, A., Birger, C., Thorvaldsdóttir, H., Ghandi, M., Mesirov, J.P., and Tamayo, P. (2015). The Molecular Signatures Database (MSigDB) hallmark gene set collection. Cell Syst 1, 417-425.
15. Kanehisa, M., and Goto, S. (2000). KEGG: Kyoto Encyclopedia of Genes and Genomes. Nucleic Acids Res 28, 27-30.
16. Gillespie, M., Jassal, B., Stephan, R., Milacic, M., Rothfels, K., Senff-Ribeiro, A., Griss, J., Sevilla, C., Matthews, L., Gong, C., et al. (2021). The reactome pathway knowledgebase 2022. Nucleic Acids Res 50, D687-D692.
17. Nishimura, D. (2001). BioCarta. Biotech softw Internet rep 2, 117-120.
18. So, J., Tai, A.K., Lichtenstein, A.H., Wu, D., and Lamon-Fava, S. (2021). Sexual dimorphism of monocyte transcriptome in individuals with chronic low-grade inflammation. Biology of Sex Differences 12, 43.
19. Varghese, M., Clemente, J., Lerner, A., Abrishami, S., Islam, M., Subbaiah, P., and Singer, K. (2022). Monocyte Trafficking and Polarization Contribute to Sex Differences in Meta-Inflammation. Frontiers in Endocrinology 13.
20. Kolmykov, S., Yevshin, I., Kulyashov, M., Sharipov, R., Kondrakhin, Y., Makeev, V.J., Kulakovskiy, I.V., Kel, A., and Kolpakov, F. (2021). GTRD: an integrated view of transcription regulation. Nucleic Acids Research 49, D104-D111.
21. Ma, X., Zhang, H., Yuan, L., Jing, H., Thacker, P., and Li, D. (2011). CREBL2, interacting with CREB, induces adipogenesis in 3T3-L1 adipocytes. Biochemical Journal 439, 27-38.
