# Supplementary material for: The impact of sex on the immune system explored at the single-cell level
Source: Am J Hum Genet. 2026 May 7;113(5):1006–23. doi: 10.1016/j.ajhg.2026.04.003 (PMC13277697; doi:10.1016/j.ajhg.2026.04.003)
Supplement: Document S2. Article plus supplemental information [file mmc3.pdf]

The impact of sex on the immune system explored at the single-cell level

Graphical abstract

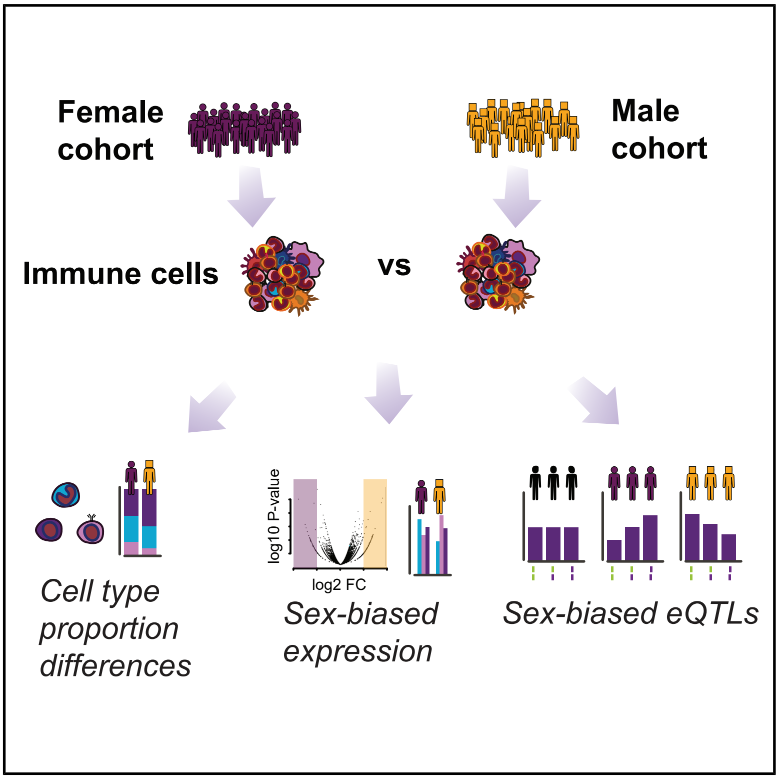

Authors

Seyhan Yazar,
Jose Alquicira-Hernandez,
Kristof Wing, ..., Alex W. Hewitt,
Joseph E. Powell, Sara Ballouz

Correspondence

s.yazar@garvan.org.au (S.Y.),
s.ballouz@unsw.edu.au (S.B.)

Yazar et al. characterized sex-based immune differences at single-cell resolution using sex-differential expression (differentially expressed genes [DEGs]) and sex-stratified expression quantitative loci (eQTLs). They identified sex-biased genes and pathways, including those previously linked to autoimmune diseases, suggesting that baseline sexual dimorphism in the immune system provides a molecular foundation for sex-specific disease susceptibility.

Yazar et al., 2026, The American Journal of Human Genetics *113*, 1006–1023
May 7, 2026 © 2026 The Authors. Published by Elsevier Inc. on behalf of American Society of Human Genetics.
https://doi.org/10.1016/j.ajhg.2026.04.003

ARTICLE

The impact of sex on the immune system explored at the single-cell level

Seyhan Yazar,[1,2,3,*] Jose Alquicira-Hernandez,[3] Kristof Wing,[4] Anne Senabouth,[3] Stacey Andersen,[5] Kirsten A. Fairfax,[4] Alex W. Hewitt,[4,6,7,10] Joseph E. Powell,[3,8,10] and Sara Ballouz[3,9,10,*]

Summary

Sex has a key role in disease susceptibility (in particular, autoimmunity). Sex differences in the immune system originate from genes and their interactions with both intrinsic and extrinsic factors. However, the cellular-level factors influencing sexual dimorphism are not fully understood. We thus examined immune sex differences at single-cell resolution to dissect the genetic impacts. Female-biased sex-differentially expressed genes (sex-DEGs) in multiple immune cells were involved in tumor necrosis factor alpha (TNF-α) signaling, whereas male DEGs were enriched for ribosomal-related functions. While *cis*-expression trait quantitative loci (eQTLs) were less common on sex chromosomes, we identified over 1,000 sex-specific eQTLs and 51 sex-interacting eQTLs on autosomes. When we examined the effect of genetic control on sex-DEGs, we found genetic variants affecting the female-biased expression of *FCGR3A* in natural killer (NK) cells (rs2099684) and *ITGB2* in monocytes (rs760462), both of which are associated with systemic lupus erythematosus. Our work reveals biases masked in bulk analyses and highlights sexually dimorphic genes and pathways at baseline.

Introduction

Sex differences in the immune system play a critical role in the susceptibility, progression and outcome of autoimmune diseases. These differences are evident in immune parameters such as antigen presentation strength and duration and intensity of immune responses, all of which vary between females and males. For instance, in females, vaccine responses are typically stronger and the rates of chronic viral infections and degree of viremia (e.g., in HIV [MIM: 609423]) are lower. However, the disadvantage of these robust immune responses is that they predispose females to higher rates of autoimmunity and inflammatory diseases. In contrast, males are more likely to develop non-reproductive cancers and become affected by bacterial and parasitic infections than females.[1,2]

At the cellular level, these clinical differences are quantifiable between males and females in both the innate and adaptive immune compartments. While males exhibit a greater number of circulating natural killer (NK) cells, females display a higher frequency of B cells.[3] Additionally, females show heightened T cell cytotoxic and inflammatory responses, particularly following multiple stimulations.[4] At the gene-expression level, most sexually dimorphic traits originate from and are influenced by the sex chromosomes. The X chromosome holds several critical immune-related genes, including interleukin receptors (*IL2RG* [MIM: 308380]), chemokines (*CXCR3* [MIM: 300574]), toll-like receptors (*TLR7* [MIM: 300365], *TLR8* [MIM: 300366]), and genes involved in T cell and B cell effector functions (*BTK* [MIM: 300300], *IKBKG* [MIM: 300248], *NKRF* [MIM: 300440]),[5] as well as regulatory molecules such as *FOXP3* [MIM: 300292] and *CD40LG* [MIM: 300386] (CD154). Their differential expression is a potential driver of sex-specific immune phenotypic variation and disease.

While differences are observable at the phenotypic and cellular levels as described, they are not apparent at the genetic level. Despite the identification of over 2,000 single-nucleotide variants (SNVs) associated with autoimmune diseases, our understanding of sex-differentiated genetic architecture remains limited. Specifically, only a small fraction of these SNVs demonstrate sex-specific effects. For example, polymorphisms of *TLR7* located on the X chromosome are a well-characterized risk factor for the autoimmune condition systemic lupus erythematosus (SLE [MIM: 301080]), which has a 9:1 prevalence in women compared to men.[6] We propose that the genetic regulation of sex-biased gene expression may provide additional evidence to clarify this unresolved research area.

Characterization of sexual dimorphism in the adaptive and innate immune systems has previously focused on investigating *a priori*-defined subsets of immune cells or on bulk analyses. While this hypothesis-driven research has demonstrated key phenotypic differences between male and female immune systems, including cell counts, population dynamics, and cytokine production, it can

[1]Precision Immunology, Garvan Institute of Medical Research, Sydney, NSW, Australia; [2]School of Clinical Medicine, UNSW Medicine and Health, Sydney, NSW, Australia; [3]Translational Genomics, Garvan Institute of Medical Research, Sydney, NSW, Australia; [4]Tasmanian School of Medicine, University of Tasmania, Hobart, TAS, Australia; [5]Institute for Molecular Bioscience, University of Queensland, Brisbane, QLD, Australia; [6]Department of Ophthalmology, Royal Hobart Hospital, Hobart, TAS, Australia; [7]Centre for Eye Research Australia, University of Melbourne, East Melbourne, VIC, Australia; [8]UNSW Cellular Genomics Futures Institute, University of New South Wales, Sydney, NSW, Australia; [9]School of Computer Science and Engineering, University of New South Wales, Sydney, NSW, Australia
[10]These authors contributed equally
*Correspondence: s.yazar@garvan.org.au (S.Y.), s.ballouz@unsw.edu.au (S.B.)
https://doi.org/10.1016/j.ajhg.2026.04.003.
© 2026 The Authors. Published by Elsevier Inc. on behalf of American Society of Human Genetics.
This is an open access article under the CC BY license (http://creativecommons.org/licenses/by/4.0/).

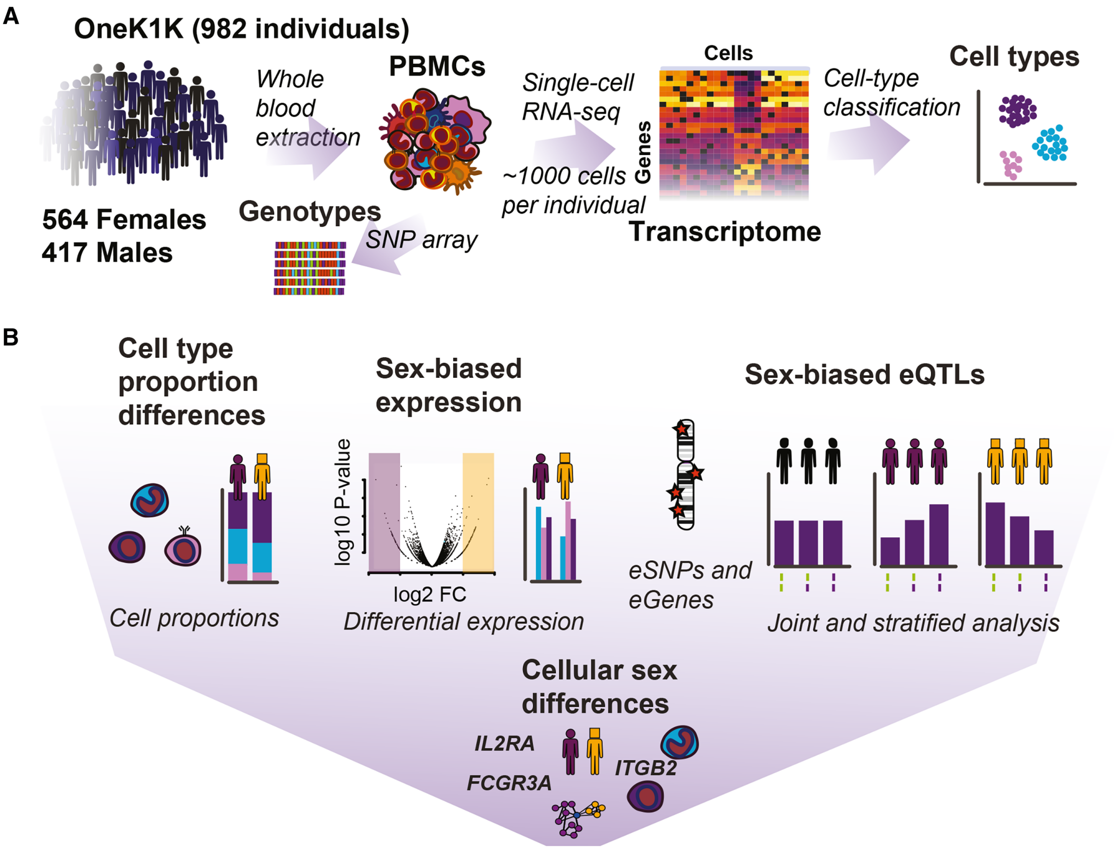

Figure 1. Overview of study
(A) Cohort: OneK1K of 982 individuals, with 564 females and 417 males. After single-cell sequencing of ~1,000 cells per individual, 1,267,758 PBMCs were classified into 30 cell types.
(B) Study design. We assessed differences in cell-type proportions, sex-biased differential expression, and sex-biased eQTLs using a statistical association framework.

lead to biased and insensitive analyses. As sex differences in the immune system arise from cellular diversity, cell-population composition, and cellular activity,[1] the application of single-cell analyses uniquely permits the unbiased characterization of sex differences in the peripheral immune system.

Here, we combine single-cell gene expression (single-cell RNA sequencing [sc-RNA-seq]) and genetic variation to assess sex differences at the cellular level in peripheral blood mononuclear cells (PBMCs) across a large cohort (Figure 1A). We evaluated cell-type proportions, sex-biased gene expression, and sex-specific and sex-interacting expression quantitative trait loci (eQTLs) along with co-expression networks (Figure 1B). By conditioning our analyses on sex and/or cell type, we disentangle their contributions to better understand the causes and consequences of sexual dimorphism in circulating immune cells.

Methods

OneK1K data and cell-type classification

Details of the OneK1K cohort and data generation have been described previously.[7] Briefly, 1,104 individuals from the Tasmanian Ophthalmic Biobank were recruited, and the study adhered to the tenets of the Declaration of Helsinki and was approved through the Human Research Ethics Committee University of Tasmania (approval number H0012902) and St Vincent's Hospital Sydney (2020/ETH01307). Written informed consent was sought from all the participants. All participants genotyped using Illumina Infinium Global Screening Array and had PBMCs sequenced using the 10× Genomics Chromium Single Cell 3′ v2 platform. Imputation was performed using the Michigan Imputation Server,[8] with Minimac4[9] and the Haplotype Reference Consortium (HRC) panel.[10]

While most participants identified themselves with a northern European ancestry in the study survey, the ancestral relationships were further investigated using genotype information. Individuals of non-European ancestry were excluded to maintain cohort homogeneity.[7] Following quality control, 982 individuals remained (564 females and 418 males); sex was confirmed through a SNP-based analysis. Approximately 1,200 cells on average were sequenced per individual, totaling 1,267,758 cells across 75 batches (i.e., pools). Samples were multiplexed (10–14 samples per pool), with a target capture of 20,000 cells per pool.

Sequencing was done with the Illumina NovaSeq 6000. Reads were processed using the Cell Ranger Single Cell Software Suite (v 2.2.0; 10× Genomics)[11] and demultiplexed into their respective pools. Mapping and alignment were performed against

GRCh37/hg19 (release 84) reference using STAR[12] within the Cell Ranger Suite. Batch correction and SCTransform normalization were performed through Seurat (v4.4.0).[13] Cell types were classified via the Azimuth pipeline with the human PBMC reference for L1 and L2 annotations[14] (Table S22). Dendritic cells subsets (ASDC, cDC1, cDC2, and pDC) were merged due to low cell counts, and other rare cell types were retained but excluded from specific analyses where higher cell numbers were required. Furthermore, genotype-based principal components (PCs) were extracted from previous work,[7] with PCs 1–4 used to account for ancestry.

Cell-type proportions

For each individual, cell-type proportions were calculated as a ratio of specific cell-type counts to the total cell count. To normalize these proportions, we applied both the *logit* and *arcsin* square-root transformation from the Speckle R package.[15] While both methods were evaluated, we report the results from *arcsin* transformation. The *propeller* function was used to determine whether the average proportions were significantly different between the sexes using an F test on the transformed data.

Normality was assessed post transformation using the Shapiro-Wilk test. Further to this, we ran the Kruskal-Wallis test (nonparametric test) on the data (without transformation) as some of the cell types failed the normality tests. To account for potential confounders, we repeated the F test using a custom design matrix ($\sim 0 + \text{sex} + \text{age} + \text{PCs1–4}$) to adjust for age and ancestry. Furthermore, as cell proportions bounded between 0 and 1, we implemented a beta-regression model using the DCATS package.[16] Specifically, we used the *dcats_GLM()* function with the adjusted design matrix to validate our findings.

Age correlation analysis

We tested for significant correlation between cell proportions and age using Spearman's rho for the correlation, and then measured significance with the adjusted *p* value (*cor.test* and *p.adjust* in R[17]). This was done per cell type, first jointly across the sexes, and then stratified by sex.

Sex-differential expression analysis

We initially performed single-cell differential expression analysis for each cell type with the *FindMarkers* function in Seurat (v4.4.0) using the Wilcoxon test with default parameters and $\log_2$ fold change ($\log_2$FC) threshold set to 0. Additionally, to adjust for confounders, we used MAST[18] within the *FindMarkers* function, incorporating age, donor ID, and PCs 1–4 as latent variables. To address correlated data structures and sample overlap across cell types, we performed a cell-type meta-analysis using multivariate adaptive shrinkage (MASH) via the R package *mashr*.[19] For all tested genes, we provided *Z* scores derived from the $\log_2$FC; for genes not tested within a specific cell type, we set the $\log_2$FC to 0. We used canonical covariances to fit the model and accounted for measurement correlations using the expectation maximization (EM) method as detailed in the *mashr* vignette. Significant sex-differentially expressed genes (sex-DEGs) were filtered using the calculated local false sign rate (LFSR) <0.05 and the cell-type-specific $|\log_2\text{FC}| > 0.1$. This $\log_2$FC threshold was determined based on an original analysis that calculated absolute fold changes of approximately 3 SD.

Sex-chromosome gene-expression analysis

To validate the sex-specific molecular profiles of captured cells, we performed dimensionality reduction using expression data from X and Y chromosome genes. Uniform manifold approximation and projection (UMAP) was generated via the Seurat R package[13] (v4.4.0) using sex-linked genes as the variable feature set to partition cells by chromosomal sex. We visualized these clustering results via their UMAP dimensions with the *DimPlot* function in Seurat and highlighted the expression of a subset of genes of interest using the *plot_density* function in Nebulosa.[20]

Cell-type gene-marker identification

For each cell type, we determined cell-type marker genes using the *FindAllMarkers* function in Seurat with a $|\log_2\text{FC}|$ greater than 0.25 and percent expressed 0.25. We then filtered on significance using a false discovery rate (FDR) of 0.05. We performed this per batch and took the genes that recurred in at least 80% of the 75 batches as markers. Finally, we repeated this procedure in a sex-stratified manner to identify cell-type markers that were conditioned on sex, allowing for the characterization of sex-specific patterns within each lineage.

Classifier and class prediction

To test for the ability of the gene sets to label or classify cells, we used a classification score based on the ranked gene-expression levels and calculated an enrichment statistic (*analytic_auroc* in EGAD[21]). An AUROC score approaching 1 indicates that the gene set is highly specific and consistently expressed within the target cell population, serving as a robust marker for classification.

Gene sets and functional-enrichment analysis

We curated gene sets from multiple biological domains. We downloaded the Gene Ontology (GO)[22,23] and the generic GO slim subset. Additionally, we used MSigDB,[24] with a focus on the HALLMARK,[25] Kyoto Encyclopedia of Genes and Genomes (KEGG),[26] REACTOME,[27] and BIOCARTA[28] gene sets and pathways. We downloaded regulatory TFs from MotifMap[29] and ENCODE TF-target[30] gene sets curated through Harmonizome.[29] Furthermore, we generated curated X-linked datasets from sex-differential and X-inactivation analysis papers. We labeled these datasets as Jansen2014,[31] Mele2015,[32] Tukiainen2017,[33] Schmiedel2018,[34] Bongen2019,[35] and Oliva2020.[36] Escape genes were selected from Tukiainen et al.[33] (Table S14); PAR genes (Table S15) and genes within the major histocompatibility complex (MHC) locus (Table S16) were extracted from GENCODE (v47[37]).

For gene set enrichment analysis, we used the hypergeometric test in R (*phyper*) and adjusted for multiple tests using *p.adjust*. For network assessment and analysis, we ran the neighbor-voting algorithm in EGAD in R,[21] which uses the guilt-by-association (GBA) principle to assess network connectivity.

Sex-specific eQTL and sex-interacting eQTL analysis

Here, we define multiple types of sex eQTLs based on their calculations. Sex-specific eQTLs are *cis*-eQTLs that show female or male effects, but not both, when sex stratified. Sex-interacting eQTLs are *cis*-eQTLs that show opposing effects in males and females or weaker effects in one sex versus the other when sex stratified. In some cases where we observe the eQTLs in the joint analysis but only in one sex, we have labeled these as ambiguous. Autosomal eQTLs are sex-specific and sex-interacting *cis*-eQTLs that

are tested on the autosomal chromosomes. Sex-chromosome eQTLs are sex-specific *cis*-eQTLs that are tested on the sex chromosomes. The analyses are split into PAR (diploid) and non-PAR (haploid) tests. Finally, we define sex-biased eQTLS as *cis*-eQTLs that show sex-specific effects or are sex interacting from both the autosomes and sex chromosomes. We go through the calculations of each in the next sections.

Joint eQTLs

We performed *cis*-eQTL analysis per cell type across all the autosomes jointly (code available from https://github.com/powellgenomicslab/onek1k_phase1). Details of this analysis were described previously.[7] In brief, average expression of each gene per person across all genes available for each cell type was calculated using the corrected counts with SCTransform.[38] We then calculated the number of individuals with non-zero expression for each gene and filtered genes expressed in less than 10% of the cohort. All values are then log transformed ($\log x + 1$). Within each cell type, *cis*-eQTLs were identified by Spearman's rank correlation testing using residual expression levels adjusted for sex, age, first four genotype-based PCs, and two PEER factors from original analysis. We restricted our search to variants within 1Mb of the TSS of either end of a gene. The resulting SNP-gene pairs were filtered at the FDR threshold of 5% at the chromosomal level for each cell type, and the most significantly associated SNPs were labeled as *cis*-eQTLs.

Equation 1: joint *cis*-eQTLs

$$\widehat{G_X} = \beta_0 + \beta_S.sex + \beta_A\,.age + \beta_{PC1}\,.PC1 \ldots + \beta_{PC4}\,.PC4 + \beta_{PF1}\,.PF1 + \beta_{PF2}\,.PF2$$

$$e_{X_0} = G_X - \widehat{G_X}$$

$\widehat{G_X}$ is a matrix consisting of the average expression of gene X per individual. e_{X_0} is the matrix including residual expression of gene X after adjusting for sex, age, six genotyping PCs, and two PEER factors.

$$for\ each\ SNP\ and\ e_{X_0}\ pair, \varrho = 1 - \frac{6\sum d^2}{n(n^2 - 1)}$$

$$\begin{bmatrix} SNP_v & \rho_1 & q_1 \\ SNP_w & \rho_2 & q_2 \\ SNP_x & \rho_3 & q_3 \\ SNP_y & \rho_4 & q_4 \\ SNP_z & \rho_5 & q_5 \\ & . & \\ & . & \\ & . & \end{bmatrix} \begin{matrix} q - value \\ \rightarrow \\ ranking \end{matrix} \begin{bmatrix} SNP_x & \rho_3 & q_3 \\ SNP_y & \rho_4 & q_4 \\ SNP_w & \rho_2 & q_2 \\ SNP_z & \rho_5 & q_5 \\ SNP_v & \rho_1 & q_1 \\ & . & \\ & . & \\ & . & \end{bmatrix} \begin{matrix} top\ SNP \\ \rightarrow \\ determined \end{matrix}$$

$$[SNP_x = eSNP_1]$$

ρ is the correlation between e_{X_0} and a matrix of three genotypes coded as 0,1 and 2 where 2 represents the assessed allele for each of 5,433,038 SNPs and q is the associated q value. d is the difference between two rankings of residuals (e_A), and n is the number of measurements.

Sex-specific eQTL discovery (stratified approach)

To identify sex-specific eQTLs, we performed a sex-stratified analysis as a discovery step then formally validated these using an interaction model.

Step 1: Discovery. In the OneK1K cohort, we have slightly more female participants than males. To ensure equal power to detect eQTLs in each sex, we downsampled the number of female participants in each cell type to match numbers of male participants.

Equation 2: sex-specific cis-eQTLs

$$\widehat{G_X} = \beta_0 + \beta_A\,.age + \beta_{PC1}\,.PC1 \ldots + \beta_{PC4}\,.PC4 + \beta_{PF1}\,.PF1 + \beta_{PF2}\,.PF2$$

$$e_{X_0} = G_X - \widehat{G_X}$$

$\widehat{G_X}$ is a matrix consisting of the average expression of gene X per individual. e_{X_0} is the matrix including residual expression of gene X after adjusting for age, six genotyping PCs, and two PEER factors.

$$for\ each\ SNP\ and\ e_{X_0}\ pair, \varrho = 1 - \frac{6\sum d^2}{n(n^2 - 1)}$$

$$\begin{bmatrix} SNP_v & \rho_1 & q_1 \\ SNP_w & \rho_2 & q_2 \\ SNP_x & \rho_3 & q_3 \\ SNP_y & \rho_4 & q_4 \\ SNP_z & \rho_5 & q_5 \\ & . & \\ & . & \\ & . & \end{bmatrix} \begin{matrix} q - value \\ \rightarrow \\ ranking \end{matrix} \begin{bmatrix} SNP_x & \rho_3 & q_3 \\ SNP_y & \rho_4 & q_4 \\ SNP_w & \rho_2 & q_2 \\ SNP_z & \rho_5 & q_5 \\ SNP_v & \rho_1 & q_1 \\ & . & \\ & . & \\ & . & \end{bmatrix} \begin{matrix} top\ SNP \\ \rightarrow \\ determined \end{matrix}$$

$$[SNP_x = eSNP_1]$$

ρ is the correlation between e_{X_0} and a matrix of three genotypes coded as 0,1 and 2, where 2 represents the assessed allele for each of 5,433,038 SNPs and q is the associated q value. d is the difference between two rankings of residuals (e_A), and n is the number of measurements.

Step 2: Filtering. After applying FDR threshold of 5% at the chromosomal level for each cell type and identifying the most significant *cis*-eQTL per gene per cell type in each sex, we implemented two additional statistical assessments to filter away false positives.

1. Distributional consistency (π_0): we estimated the proportion of null hypotheses (π_0) for the female-only eQTLs in the male dataset (and vice versa).[39] We removed associations where the opposite sex showed evidence of an underlying signal, ensuring we only retained associations that were truly different (threshold π_0 > X).[39]
2. Effect size comparison (Z test): we used the two-sample Z test to compare the beta estimates of two populations (β_{female} vs. β_{male}). For this analysis, we ran the stratified analysis using *MatrixEQTL*[40] and generated beta estimates and standard errors for each SNP-gene pair in each cell type for females and males. Next, we calculated the z-statistics for *cis*-eQTLs identified in each sex analysis and retained only eQTLs with significant difference in magnitude ($p_{Z\text{-}test} < 0.05$). In our final step, we defined a sex-specific eQTL only if the *cis*-eQTL passed both levels of testing.

Step 3: Validation. We applied a SNP × sex interaction model to all candidate eQTLs that passed the filtering steps. We assessed the significance of interaction term, considering the candidates are validated if they reach an FDR threshold of <0.05.

Note that, for each of the three analyses (joint, female, and male specific), we controlled for multiple testing using the FDR and considered associations significant if their q values (FDR-adjusted p values) were ≤ 0.05.

Sex-interacting eQTLs

Next, to identify sex-differential effects among robust, "established" associations, using the joint *cis*-eQTLs results, we ran a sex-interaction analysis to identify eQTLs with varying effect sizes by sex. In this analysis, for each gene-SNP pair within each cell type, we fitted a linear regression model and tested for genotype-by-sex interaction while adjusting for previously mentioned additional factors:

Equation 3: sex-interacting cis-eQTLs

$$y = \beta_0 + \beta_S.sex + \beta_A\ .age + \beta_{PC1}\ .PC1 \ldots + \beta_{PC4}\ .PC4 + \beta_{PF1}\ .PF1 + \beta_{PF2}\ .PF2 + \beta_G.genotype + \beta_{GxS}.genotype\ .\ sex \quad \text{(Equation 3)}$$

where y is the gene expression, β_0 is the intercept, and β is the corresponding effect size. β_{GxS} is the effect size of genotype-by-sex interaction on gene expression. Since we have already applied a multiple-testing correction and accounted for the number of independent eQTLs tested per chromosome in our initial analysis, we applied Storey q value across genes to identify genes with at least one significant (FDR $\leq$ 0.25) sex-interacting eQTL.

We also repeated our original analysis in a sex-stratified manner, using age, first four genotype-based PCs, and two PEER factors for each sex.

$$\widehat{G_X} = \beta_0 + \beta_A\ .age + \beta_{PC1}\ .PC1 \ldots + \beta_{PC4}\ .PC4 + \beta_{PF1}\ .PF1 + \beta_{PF2}\ .PF2\ \ e_{X_0} = G_X - \widehat{G_X}$$

$$for\ each\ SNP\ and\ e_{X_0}\ pair, \varrho = 1 - \frac{6\sum d^2}{n(n^2-1)}$$

$$remove\ this\ (merged\ with\ above)$$

$$\begin{array}{ccc} SNP_v & \rho_1 & q_1 \end{array} \qquad\qquad \begin{array}{ccc} SNP_x & \rho_3 & q_3 \end{array}$$

$$\begin{bmatrix} SNP_w & \rho_2 & q_2 \\ SNP_x & \rho_3 & q_3 \\ SNP_y & \rho_4 & q_4 \\ SNP_z & \rho_5 & q_5 \\ & . & \\ & . & \end{bmatrix} \begin{array}{c} q - value \rightarrow \\ ranking \end{array} \begin{bmatrix} SNP_y & \rho_4 & q_4 \\ SNP_w & \rho_2 & q_2 \\ SNP_z & \rho_5 & q_5 \\ SNP_v & \rho_1 & q_1 \\ & . & \\ & . & \end{bmatrix}$$

$$\qquad .\qquad\qquad\qquad\qquad .$$

$$\begin{array}{c} top\ SNP \rightarrow \\ determined \end{array} [SNP_x = eSNP_1]\rho$$

It is important to note that we applied Storey q value[39] across genes to identify genes with at least one significant (FDR $\leq$ 0.25) sex-interacting eQTL. We made this choice given the reduced power of interacting testing and the exploratory nature of this analysis, which aims to identify broad patterns and prioritize candidate genes for future validation. Applying a stricter threshold ($\leq$0.10 and $\leq$0.05) yielded a smaller number of associations, justifying a more relaxed threshold to have more meaningful biological signals.

Imputation of the sex chromosomes

The sex chromosomes in humans share pseudoautosomal regions (PARs) and are assessed as diploid regions. PAR1 (X 60,001 to 2,699,520 and Y 10,001 to 2,649,520) and PAR2 (X 154,931,044 to 155,260,560 and Y 59,034,050 to 59,363,566) are located at the tips of the chromosomes and recombine. The remainder of each chromosome is labeled the non-PAR. In females, the non-PAR X is genotypically diploid, while in males the non-PAR Y is haploid. Thus, to analyze the sex chromosomes, we needed to impute them split by these regions. For the X chromosome, we extracted genotyped SNPs from the genotype PLINK file from the non-PAR (chrX or 23) and PAR (1 and 2) (chrXY or 25). Further filtering was performed on the PAR variants to match the HRC panel. Imputation was performed using the Michigan Imputation Server[8] with Minimac4[9] and the HRC panel[10] separately for the non-PAR and PAR segments. For the Y chromosome, we extracted genotyped SNPs from the genotype PLINK file (chrY or 24). As the non-PAR Y does not undergo recombination like the non-PAR X does in females, we cannot impute genotypes on the Y. Instead, we can haplotype the Y based on their genotypes. To this end, we ran *yhalpo*[41] to identify the broad haplogroup of the male individuals.

Sex-chromosome eQTL analysis

We performed first sex stratified then joint eQTL analysis for PAR1, PAR2, and non-PAR regions separately. When PAR1 regions were tested, both genotypes on chromosome X and Y were modeled as 0 (homozygous for allele 1), 1 (heterozygous for allele 1), and 2 (homozygous for allele 2) and the analysis was performed as described for sex-specific eQTLs (Equation 3). Due to differences in gene locations on X and Y chromosomes, we tested an average of 4,419 SNP-gene pairs per cell type in females and 4,318 SNP-gene pairs per cell type in males. Joint sex-chromosome eQTLs were identified as depicted in Equation 1, where sex was included as a covariate when calculating the residuals before testing for Spearman's rank correlation. To overcome the differences in available SNP-gene pairs between females and males, only pairs that were present in both sexes were included in this analysis. We conducted the eQTL analysis for PAR2 region in the same manner as PAR1. A similar approach was followed when analyses were performed for the non-PAR region with the difference being that, when testing for males, genotypes on chromosome X were modeled as 0 (homozygous for allele 1) and 1 (homozygous for allele 2) and analysis was completed using non-parametric Mann-Whitney U-test.

Results

Single-cell data reveal cell-type proportion differences

We classified ~1.25 million cells from 982 individuals from the OneK1K study[7] (565 females, 418 males) into 30 transcriptionally distinct cell types using the Azimuth classification tool[13] (Figures 2A and 2B). Calculating proportions of each cell type per individual, we observed clear

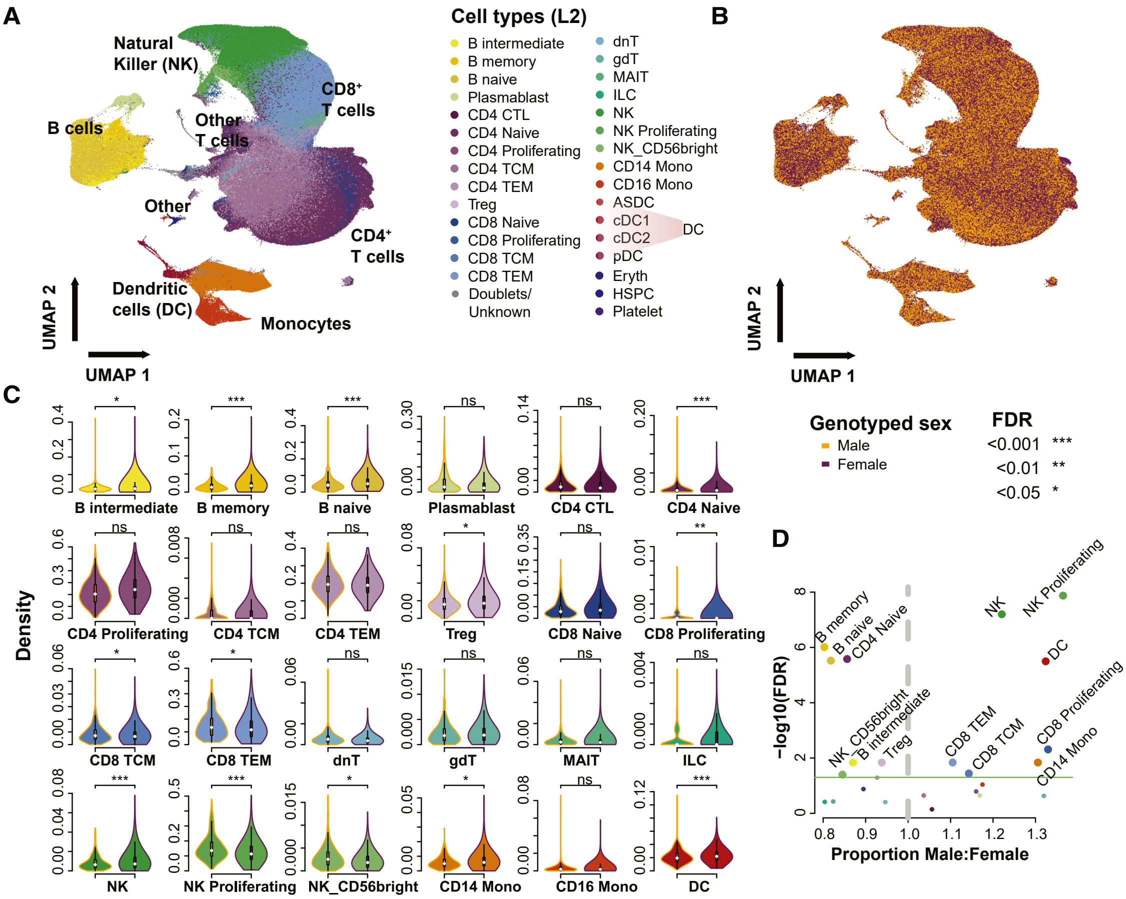

Figure 2. Distributions of cell-type proportions across sex

(A) UMAP of all 1,267,758 cells, colored by cell type. Dendritic cell labels (ASDC, cDC1, cDC2, and pDC) were combined for all downstream work.

(B) UMAP of cells colored by sex: males in gold, females in purple.

(C) Density plots showing the distribution of proportions of each cell type. Females are shown as a density plot on the left, and males on the right. Significance of differences based on an FDR from an F test (proportions) are indicated by asterisks (*FDR < 0.05, **FDR < 0.01, ***FDR < 0.001).

(D) Male to female ratios versus the FDR.

compositional differences between the sexes (Figures 2C and 2D; Table S1). In males, we found higher proportions of CD14$^+$ monocytes, dendritic cells (DCs), NK cells, NK proliferating cells, CD8$^+$ proliferating cells, T-effector memory (TEM) cells, and T-central memory (TCM) cells. In females, we found significantly higher proportions of B cells, CD4$^+$ naive T cells, NK CD56$^+$, and regulatory T cells (Tregs). Most of these differences have been reported in the literature,[3,42–45] yet some were not previously reported, including Tregs and DCs.

In the innate immune system, CD14$^+$ monocytes were present at higher proportions in males (3.71% in males versus 2.84% in females; FDR = 0.014). Higher proportions of monocytes have been reported in male infants[46] and some ethnicities,[47] but it is not a well-established phenomenon between the sexes.[48,49] No proportional difference was present in CD16$^+$ monocytes, consistent with others' observations.[48] Due to low overall counts, we combined the DC subtypes plasmacytoid (pDC), conventional cell 1 (cDC1), conventional cell 2 (cDC2), and *AXL*+ DC (aDC) into a broad dendritic cell category. We observed a strong relationship between sex and DC proportion, with higher percentages in males (0.64% in males vs. 0.48% in females; FDR < 0.001). This has not been previously observed in flow-cytometric studies of immune variation.

In the adaptive immune compartment, we found higher proportions of Tregs in our female samples (2.18% in males vs. 2.32% in females; FDR = 0.0145), whereas the opposite or no difference was typically reported.[44,50] Tregs vary during the female menstrual cycle in response to estrogen[51] and other sex steroids, so these results are potentially confounded with sex hormone levels. As we did not have associated hormone-related information, we used age as a proxy to test our hypothesis. As an in-depth analysis with age is beyond the scope of this work,[52] we performed a correlation analysis (Figure S1; Table S2) and identified no significant correlation between Tregs and age.[53] To adjust for confounders and consider statistical assumptions, we tested potential alternative models and

ran additional diagnostic checks. These included the incorporation of age and ethnicity as covariates as well as the application of alternative modeling frameworks, specifically a non-parametric Kruskal-Wallis test and a beta-regression model (see methods). We observed no changes to the results (Table S3).

Sex-differential expression by cell type reveals sex biases obscured in bulk analyses

To measure differences in response and activity across the transcriptome, we performed a differential expression analysis between the sexes to identify sex-biased gene expression for each cell type. We detected between three and 33 sex-DEGs per cell type prior to multiple test correction. Adjusting for confounding factors (age and ethnicity) retained the same DEGs (MAST[18]), with the exception that *NKG7* (MIM: 606008) was no longer significant in gdT. To model the effects across cell types, given sample dependence and potentially correlations, we applied MASH.[19] Following this analysis, we detected 78 DEGs across 24 cell types: 16 genes with male-biased expression and 65 genes with female-biased expression (Figures 3A and 3B; Tables S4, S5, S6, and S7), and three showing both female and male-biased expression in different cell types (*CD79A* [MIM: 112205]), *TSC22D3* [MIM: 300506], and *JUN* [MIM: 165160]). These genes showed female-biased expression in B cells but were more highly expressed in male dendritic cells. Of the three, TSC22 Domain Family Member 3 (*TSC22D3*), also known as glucocorticoid (GC)-induced leucine zipper (*GILZ*), is a known sex-biased and X-linked gene, while the others were not previously reported. Important to note that, of the 78 genes, only 16 of these genes were on the sex chromosomes (11 on the X and five on the Y), implying additional sex-specific regulation of autosomal genes. For further sensitivity analysis, see the supplemental notes.

We tested for overlap between our results and known sex-DEGs from bulk RNA-seq studies and X-linked gene sets of interest[31–36] (Figure 3C; see methods). Of the 78 genes, 42 have been identified as sex-DEGs in bulk studies of whole blood or other tissues. We also noted consistent sex-biased expression profiles. That is, if it is upregulated in females (female biased), this expression pattern is maintained at the cellular level (Table S5). The exceptions include *FLNA* (filamin A [MIM: 300017]) and *SAT1* (Spermidine [MIM: 313020]). These are male-biased sex-DEGs in bulk, but both appear female biased in our data. This inversion of biased expression could also be due to bulk analyses masking expression or could be linked to variation in X escape of these genes in different tissues and cell types. Of the remaining 36 genes, around 17 were detected in one cell type, suggesting that there are signals at single-cell resolution results obscured in bulk.

Immune pathways are enriched in sex-biased genes

To quantify the overlap between sex-biased genes and their functions, we tested for gene set enrichment of pathways and gene groups (Figure S2). The female-biased sex-DEGs in B intermediate, B naive, $CD14^+$ monocytes, $CD8^+$ TEM, and NK cells were enriched for the tumor necrosis factor alpha (TNF-α) signaling pathway (adjusted p: B intermediate $\sim 2.04 \times 10^{-9}$, B memory $\sim 6.74 \times 10^{-3}$, $CD14^+$ Mono $\sim 2.81 \times 10^{-2}$, $CD8^+$ TEM $\sim 2.94 \times 10^{-3}$, and NK $\sim 3.41 \times 10^{-4}$), which included genes regulated by nuclear factor kappa-light-chain-enhancer of activated B cells (NF-κB) in response to TNF-α expression. TNF-α is a cytokine used by the immune system for cell signaling, and dysregulation of NF-κB has been linked to inflammatory and autoimmune diseases.[54] The genes driving this enrichment were similar for most cell types and included *JUN*, *DUSP1* (MIM: 600714), *DUSP2* (MIM: 603068), *IER2* (MIM: 620036), *ZFP36* (MIM: 190700), *CD69* (MIM: 107273), *CD83* (MIM: 604534), *SAT1*, *KLF6* (MIM: 602053), and *PPP1R15A* (MIM: 611048). Many of these genes encode proteins involved in the hypoxia pathway that was significantly enriched in B cells (p-adjusted: B intermediate $\sim 9.72 \times 10^{-5}$, B naive $\sim 5.73 \times 10^{-3}$). Furthermore, these genes also encoded proteins involved in cellular proliferation, specifically of T cells.[55] Interestingly, the $CD14^+$ monocytes had a distinct set of genes driving the TNF-α enrichment, including *G0S2* (MIM: 614447), *NFKBIA* (MIM: 164008), and *PLAUR* (MIM: 173391). The proteins encoded by these genes were also involved in the inflammatory response ($CD14^+$ monocytes p-adjusted $\sim 8.88 \times 10^{-4}$), along with *CD14* (MIM: 158120) and *EMP3* (MIM: 602335), both linked to monocyte differentiation/proliferation. In $CD4^+$ CTLs, the interferon-gamma response (p-adjusted$\sim 3.77 \times 10^{-9}$) and allograft rejection (p-adjusted$\sim 2.20 \times 10^{-2}$) were enriched in female-biased sex-DEGs. Some genes were shared between these two pathways, including *CD2* (MIM: 186990), *GZMA* (MIM: 140050), *HLA-A* (MIM: 142800), *HLA-E* (MIM: 143010), *IL2RG* (MIM: 308380), *FLNA*, and *CCL5* (MIM: 187011). The sex specificity of all these genes is unclear as their proteins have broad functions; however, this could be due to the higher activity of these pathways in females. Monocytes are reported to have increased functional activity in females, summarized as primed interferon (IFN)/immune pathways and overexpression of immune genes at basal levels.[56] Male-biased sex-DEGs were enriched for ribosomal-related functions and pathways. These include rRNA processing, translation, and metabolism of RNA pathways (Figure S6B).

One mechanism for sex differences in gene expression is believed to occur through gene regulation by sex hormones and their receptors in immune cells.[57] To evaluate this hypothesis, we tested for enrichment of sex hormone receptor target gene sets (estrogen receptors *ESR1* [MIM: 133430], *ESR2* [MIM: 601663], and androgen receptor *AR* [MIM: 313700]) from MotifMap.[58] We found no enrichment of the receptor target genes in the DEGs (Figure S2F). Instead, genes with female-biased expression in $CD14^+$ monocytes were enriched for the *ESR1* TF targets gene set from ENCODE (*G0S2*, *ITGB2*

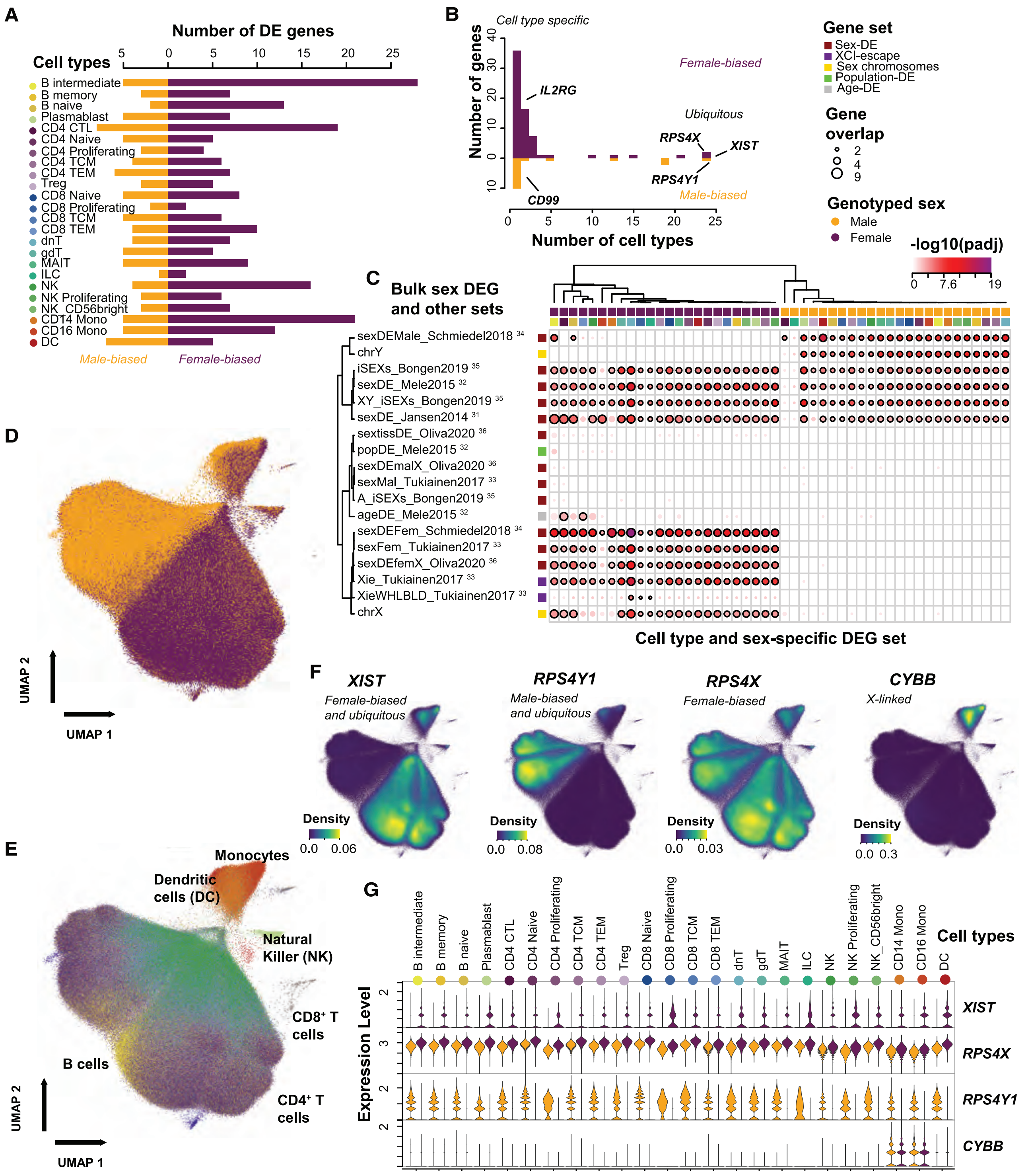

Figure 3. Sex-differential expression

(A) Number of sex-DEGs using the Wilcoxon test per cell type (two sided).

(B) Recurrence of these DEGs shows mostly unique genes, but sex-specific markers were recurrent across all cell types (ubiquitous).

(C) Gene set enrichment analysis of DEGs showing enrichment of sex-specific gene sets. Additionally, age-related enrichment in DEGs of CD8 TEMs and CD4 CTLs and population-related genes in B intermediate cells.

(D) Exploiting variation of the genes on the XY chromosomes to visualize sex through a UMAP.

(E) Then colored by cell types, showing that some cell-type markers are on the sex chromosomes.

(F and G) (F) UMAPs and (G) violin plots of genes of interest. Cell-type-specific markers (*XIST* and *RPS4Y1*) show clear sex differences. Expression of X-linked genes *CYBB*, with no sex bias, and *RPS4X*, which is differentially expressed.

[MIM: 600065], *MALAT1* [MIM: 607924], *MT2A* [MIM: 156360], *MYL6* [MIM: 609931], *NFKBIA*, *S100A10* [MIM: 114085], and *S100A11* [MIM: 603114], *p*-adjusted $\sim 5.42 \times 10^{-4}$). Additionally, *LGALS2* (MIM: 150571) (sex-DE in $CD14^+$ monocytes) was a target of *ESR2* (ERβ). Monocyte cell counts decrease as estrogen levels increase through the Fas/FasL system and the ERβ receptor (*ESR2*).[59] Thus, our analysis highlights the role of estrogen in differing monocyte cell counts and their functional activity.

Genes responsible for establishing immune cell-type identity are mostly distinct from those determining sex identity

Cell-type transcriptional identity is defined by the expression levels of marker genes, some of which are located on the sex chromosomes. These genes are not merely markers but also potentially functional elements. We examined the intersection between the sex-chromosome genes, sex-DEGs (including autosomal), and known cell-type marker genes to assess their impact on cellular and sex-linked identity. We first evaluated the variation of sex-chromosome genes within a dimension reduction framework (UMAP), which allowed us to visualize the influence of these genes on cell identity, agnostic of their sex-biased expression profiles. Using the variation of genes expressed on the X and Y chromosomes, we observed a clustering by sex (Figure 3D) and a strong clustering by the myeloid and lymphoid lineages, with monocytes and dendritic cells split from the remaining cell types (Figure 3E). This separation indicates an important role for a subset of sex-chromosome genes in monocyte and dendritic cell identity with between 10 and 16 marker genes located on the sex chromosomes (examples in Figures 3F and 3G). However, this overlap was not significantly enriched but was strong enough to drive the clustering. We do note, although the clustering is quite tight, that this may be due to artificial structures generated from the dimensionality-reduction approach.

To further explore this finding and focus on a potential set of sexually dimorphic and immune-related genes, we tested for overlap of the sex-biased genes (sex-DEGs) and cell-type marker genes. For this, we utilized the L2 cell-type classification marker genes from the Azimuth reference panel. Overall, 20 of the 215 cell-type marker genes (Table S23) were sex biased. When we looked at the cell-type-specific distribution of these 20 genes, we found 14 of them were both cell-type markers and sex-DEGs within the same cell type (Table S5). These included *CD79A* (MIM: 112205) in B intermediate and naive cells; *CXCR4* (MIM: 162643) in B naive cells; *CD14*, *G0S2*, and *S100A8* (MIM: 123885) in $CD14^+$ monocytes; *FCGR3A* (MIM: 146740) in $CD16^+$ monocytes; *FGFBP2* (MIM: 607713), *GZMA*, and *GZMH* (MIM: 116831) in CD4 CTLs; *GZMK* (MIM: 600784) in CD4 TEM, CD8 TEM, and MAIT cells; *KLRC1* (MIM: 161555) in gDT; *NKG7* in MAIT; and finally *FCER1G* (MIM: 147139) in NK cells. Of these, none were on the sex chromosomes.

The 49 autosomal sex-biased marker genes could play crucial roles in cell function and in sexual dimorphism of immune traits. For example, the autosomal gene *CCL5*, which is a marker for $CD8^+$ T cells, shows cell-type-specific expression and is differentially expressed by sex, with higher expression in females in $CD8^+$ TCM cells (log2FC $= -0.22$, FDR $\approx 2.02 \times 10^{-22}$). Increased expression of *CCL5* is potentially linked to improved antiviral immunity through lymph node and splenic homing of viral-specific CD8 T cells.

Sex-specific *cis*-eQTLs at the single-cell level are mostly cell-type specific

Sexually dimorphic phenotypes may partly derive from genetic effects and their interactions with the environment. Several eQTLs have been identified to show sex-biased or sex-interacting effects using bulk RNA-seq[36,60,61] and more recently using single-cell sequencing of lymphoblastoid cell lines[62] and PBMCs from the Asian Immune Diversity Atlas.[63] To understand the impact of sex on genetic control of gene expression at the single-cell level at the population scale, we tested for *cis*-eQTLs on the autosomes (Figure 4) and sex chromosomes (Figure 5). For autosomes, we first performed a joint analysis of both sexes to identify robust shared eQTLs. We found 14,432 autosomal eQTLs across 21 cell types in our joint analysis (Figure 4B; Table S10). Next, we conducted a sex-stratified discovery analysis in downsampled groups to identify sex-specific eQTLs. The candidate eQTLs from this analysis were then filtered using a two-sample Z test and π_0 estimation and subsequently validated through a genotype-by-sex model (Figure 4A; Table S9). In our stratified analyses, we found fewer eQTLs overall, likely due to reduced power from stratification and stringent filtering, with 1,038 eQTLs in females and 990 in males (Figure 4B; Tables S11 and S12). Between cell types, many significant eQTLs were also unique (i.e., cell-type specific; Figure 4C) (global q <0.05); however, these discoveries are based on the number of available cells for that cell type.[7] A total of 951 eGenes were observed in males, with 39 (4%) occurring in more than two cell types. In females, of the total of 989 eGenes, 41 were in two cell types, and four genes were in three (4.6% > 1). Additionally, 14 female-specific eGenes were in the MHC region, compared to 16 male-specific eGenes. Although the Spearman's ρ estimates for sex-specific eQTLs were modest (|Rho| $\approx$ 0.3), these associations were significant, reflecting consistent but weaker genotype-expression correlations relative to joint eQTLs (Figure 4D). This reflects low but significant effects of SNPs on sex-specific eGenes. While the SNPs differed, 122 eGenes were common to both male and female associations. The majority of these eGenes (107) showed significance in distinct cell types, indicating that a small subset of genes may have a sex-specific and cell-type-specific regulatory mechanism. The 15 eGenes in the same cell types had variants that were not in linkage disequilibrium

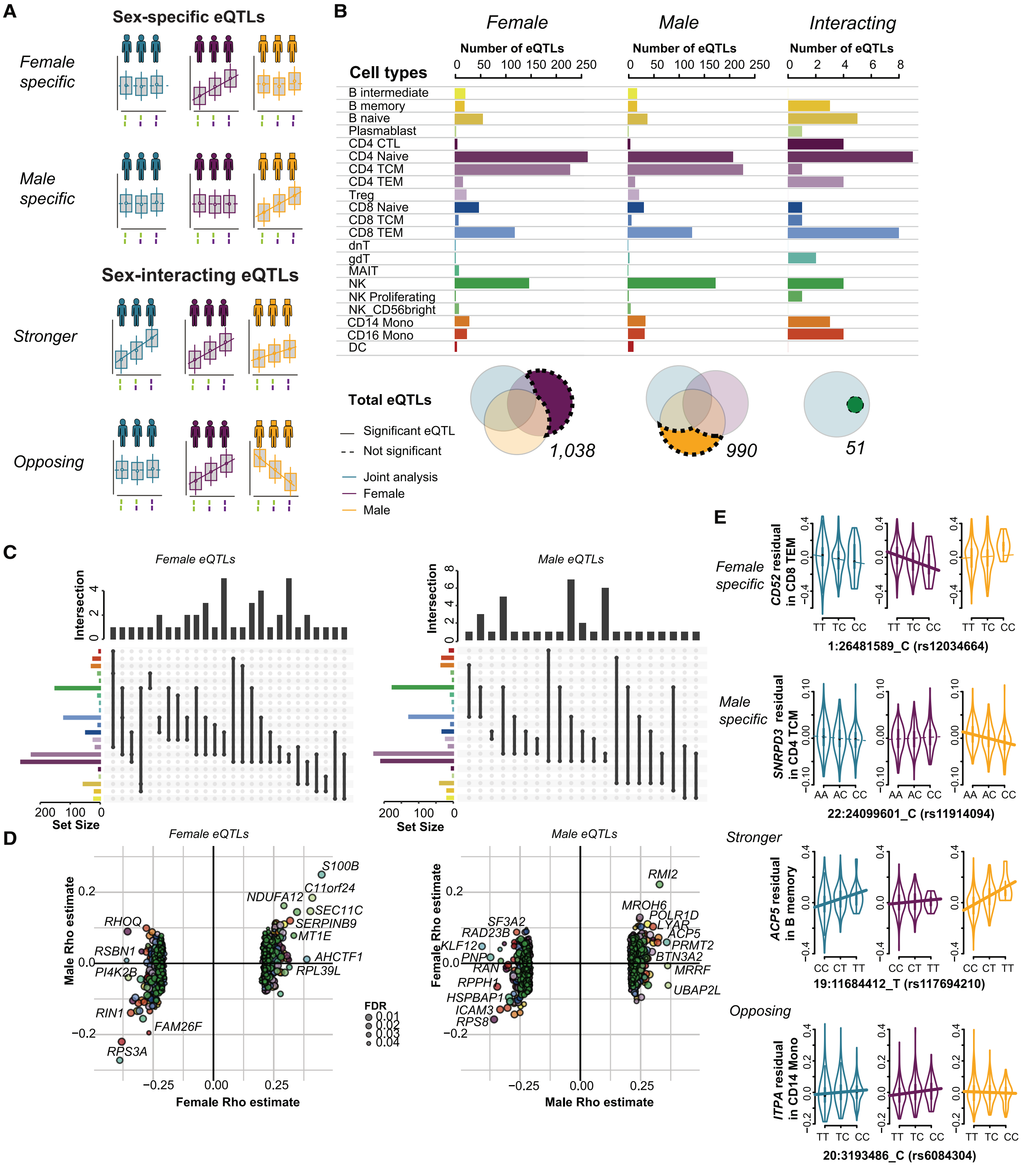

Figure 4. Sex-linked *cis*-eQTLs and sex-interacting eQTLs

(A) Sex-biased eQTLs can either be sex specific or sex interacting. Sex-specific eQTLs can be female or male, without significant association in the joint analysis or in the opposite sex. Sex-interacting eQTLs are significant in all analyses but have different effect sizes. Joint analysis in the model is colored turquoise, with males in gold and females in purple.

(B) Total number of *cis*-eQTLs per cell type across the joint, stratified, and interacting eQTLs.

(C) Overlapping *cis*-eQTLs across cell types for female-specific (left) and male-specific (right) eQTLs.

(D) Scatterplot of effect sizes (rho estimates) for female (left) and male (right) specific eQTLs plotted against estimate in the opposite sex. Colored by cell type, and size of point reflects sex-specific FDR.

(E) Example eQTLs for all four sex-biased eQTLs from (A).

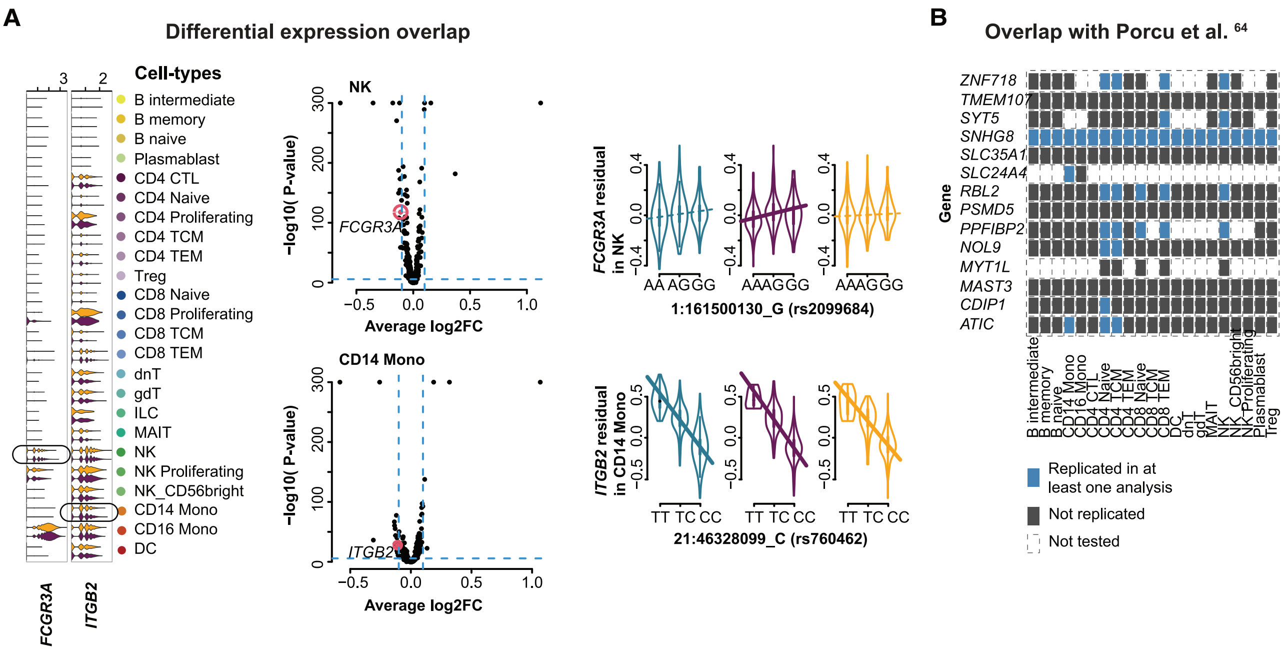

Figure 5. Overlap and replication of *cis*-eQTLs

(A) Overlap of sex-biased eQTLs with a sex-biased expression showing eQTL plot, differential expression, and expression of genes *FCGR3A* (female specific) and *ITGB2* (interacting).

(B) Replication of sex-interacting SNPs from Porcu et al.[64] in our data per cell type. Blue indicates replication, while gray boxes describe no replication. Dashed were not tested.

(LD), suggesting these eGenes were also under different genetic regulatory control.

We next tested for functional enrichment of the eGenes with sex-specific associations. We identified enrichment of transcription factor targets and immune-related functions using MSigDB.[25] Genes with associations in either females or males are likely to have sex-specific functions, which are not captured by the enrichment. For example, the female-specific eQTL of *CD52* (MIM: 114280) rs12034664 in CD8 TEMs (Figure 4E) may be linked to downstream activity: the CD52 glycoprotein functions as an effector molecule in suppressing Tregs in type 1 diabetes (MIM: 222100).[65] Interestingly, a second variant in DC (rs11577318) is a male-specific eQTL. These SNPs are not in LD (rs11577318 and rs12034664, $R^2 = 0.0087$) and thus exhibit independent control of the same gene in different cell types. Another eGene of interest is *SNRPD3* (MIM: 601062) with a male-specific eQTL rs11914094 in CD4 TCMs (Figure 4E). *SNRPD3* encodes a subunit of the spliceosome in humans, but, in mice, this gene and associated complex have increased expression in male mice, believed to be linked to sex determination through alternative splicing.[66]

Sex-specific allele effects drive gene-expression differences

The previous stratified analysis focused on *cis*-eQTLs that were absent in the other sex, yet there may be different effect sizes of the same variant, which we call sex interacting. To identify sex-interacting eSNPs, we took eQTLs from the joint set and tested for a genotype-by-sex interaction (see methods). This analysis highlights expression differences that occur when the allelic effect of genotype differs between males and females.[61] We identified 51 sex-interacting eQTLs (Table S13). The majority (46 eSNPs) were in the same allelic direction in both sexes but with a noticeable difference in magnitude; specifically, 27 showed a stronger effect in males and 19 in females. While we used a discovery threshold of FDR ≤ 0.25, we found a core set of 12 and two associations remain significant at the more stringent FDR <0.10 and <0.05 levels, respectively (Table S13).

Our approach also detected effects present in one sex or in the opposite direction of the allelic effect (45 eGenes). These include *NLRP2* (MIM: 609364) ($p = 3.18 \times 10^{-3}$, FDR = 0.17), *ITPA* (MIM: 147520) ($p = 5.98 \times 10^{-3}$, FDR = 0.19), *IL2RA* (MIM: 147730) ($p = 2.11 \times 10^{-9}$, FDR = 0.079), and *NAGK* (MIM: 606828) ($p = 4.52 \times 10^{-3}$, FDR = 0.19). *NLRP2* (NACHT, LRR, and PYD domains-containing protein 2, rs12969457 B naive) is a sensing component of NLRP2 inflammasomes and contributes to the regulation of immune responses regulating activities of NK-κB and acting as a pro-inflammatory molecule through caspase-1 activation.[67] Furthermore, NLRP2 also has reproductive functions, believed to be responsible for maintaining fertility in females[68] and establishing maternal-fetal tolerance during pregnancy.[69] *ITPA* (inosine triphosphate pyrophosphatase ITPase, rs6084304 CD14^{+} monocytes) variants have been linked to chronic hepatitis C response treatment efficacy.[70] For

IL2RA (interleukin-2 receptor subunit alpha, rs7261003, CD8 TEM), the interleukin-2 receptor is involved in regulating immune tolerance by controlling the activity of Tregs.[71]

Sex-biased gene expression in SLE-associated genes is influenced by genetic regulation

To link gene expression that is biased between sexes to genetic regulation, we examined the overlap between sex-biased eQTLs and the sex-biased DEGs. We identified most of the overlap signals occurring in eQTLs with joint effects rather than those with sex-specific effects (19 genes). However, we found one gene with a female-specific eQTL and sex-biased expression: *FCGR3A* in NK cells. This gene forms part of the immunoglobulin gamma (IgG) receptor and mediates IgG effector function in NK cells. It has also been associated with sex-linked traits: immunodeficiency of NK cells,[72] including susceptibility to recurrent viral infections[73]; severity of COVID-19[74]; and the autoimmune disease SLE.[75] More specifically, the rs2099684 variant is associated with Takayasu arteritis (MIM: 207600) in the Han Chinese population, which has a 90% female bias.[76] Other variants (rs396991) on *FCGR3A* are shown to affect the efficacy of antibody-dependent NK cell-mediated cytotoxicity in patients receiving rituximab treatment.[44] Additionally, we find a *ITGB2* sex-interacting eQTL in $CD14^+$ monocytes, which also shows female-biased expression in our data (Figure 5A). *ITGB2* (Integrin beta chain-2), along with the alpha subunit, encode integrin heterodimers involved in cell adhesion and cell-surface mediated signaling. It is linked to the inflammatory response in monocytes,[77] along with roles in the autoimmune disease systemic sclerosis (scleroderma (MIM: 181750)).[78] More recently the *ITGB2* signaling pathway was shown to be enriched both in SLE and primary Sjogren's syndrome (MIM: 270150).[79] However, the specific variant (rs760462) has no known clinical correlation. Nevertheless, these two genes are also located in two distinct *cis*-regulatory elements and are interesting examples of sex-specific genetic regulation.

To verify and replicate our sex-specific eQTLs on autosomes, we looked for overlap with other bulk studies available.[36,61,64,80] In these studies, little replication was evident across them. Of the 26 genes identified overall, we successfully replicated 10 eGenes/eQTLs across various cell types using different analyses (Figure 5B). Once again, this analysis suggests that single-cell resolution can detect associations missed in bulk. Lastly, we conducted a gene co-expression network analysis (see supplemental notes and methods), considering cell type and sex specificity, to identify additional genes sharing common regulatory control or pathway involvement (Figures S7–S10; Table S22). However, the generated networks did not overlap with modules for either *FCGR3A* or *ITGB2*.

cis-eQTLs are depleted on the sex chromosomes

The effects of genetic variants on the sex chromosomes have not been thoroughly assessed in many eQTL studies, and this may explain some of the observed sex differences. To assess the sex chromosomes for eQTLs, we separated our analysis of the X into PAR and non-PAR and then further into X-escape and non-escape genes (Figure 6A; Table S17). The non-PAR regions of the X chromosome are haploid, as females inactivate one of their X chromosomes, and males only have one copy of the X. This region spans most of the X chromosome, approximately 152 Mbp. Around 953 genes are located in this region (GENCODE hg19/GRCh37), with 41 immune-related genes (~4%) and approximately 66 genes escaping X-inactivation.[33] The pseudoautosomal regions (PAR1 and PAR2) are short regions of homology between the X and Y chromosomes at the tips of both chromosomes. The PARs are thus diploid, as genes on the female inactive X escape inactivation. In total, 26 protein-coding and lncRNAs sit in these PARs.

For the PAR analysis, we genotyped approximately 1,300 SNPs on the PAR XY, with around 300 passing QC. As with the autosomes, we ran our analyses both jointly and stratified (Figure 6B; Table S18). In the joint analysis, we identified 14 eQTLs in PAR1 and six in PAR2, totaling of 20 eQTLs (nine eGenes). In the stratified analysis, we found eight eQTLs in females and five in males, all in PAR1. We identified no significant associations in PAR2, which may be due to a loss of power. All eGenes detected in the stratified analysis were detected in the joint analysis (Figure 6C) except *AKAP17A* (MIM: 312095), a protein kinase A anchoring protein, found in female Tregs ($FDR \approx 0.0107$),[81] and *IL3RA* (MIM: 308385) in females in NK cells ($FDR \approx 0.01$). The latter encodes CD123 (interleukin 3 receptor alpha) and has been shown to influence COVID-19 responses between the sexes.[82]

In the non-PAR joint analysis, we found fewer eQTLs on the X chromosome relative to autosomes (Figure 6D; Table S19). We tested genes that do not escape X-inactivation by removing known X-escaping genes from our list (66 genes[33]; Table S14). This was done to compare similar gene dosages across males and females. We identified 97 significant eQTLs in the female-stratified analysis. In males, we detected 124 eQTLs on the non-PAR X chromosome. The additional results in the male analysis were likely due to variation in XCI for heterozygous females. We jointly repeated the analysis and identified 267 eQTLs. Of the 97 eQTLs identified in females, 69 were unique (i.e., not in the joint and male analysis), and, of the 124 in males, 93 were unique. Finally, we examined genes that are known to escape X-inactivation in females. In this analysis, we found 16 eQTLs, with *XIST* and *RPS4X* (MIM: 312760) being the most recurrent across cell types (Figure 6E). Many sex-biased eQTLs that were in the same eGenes had differing lead SNPs. For example, in NK cells (Figure 7), 13 eGenes had all male-specific associations (e.g., *AMOT* [MIM: 300410]). At the

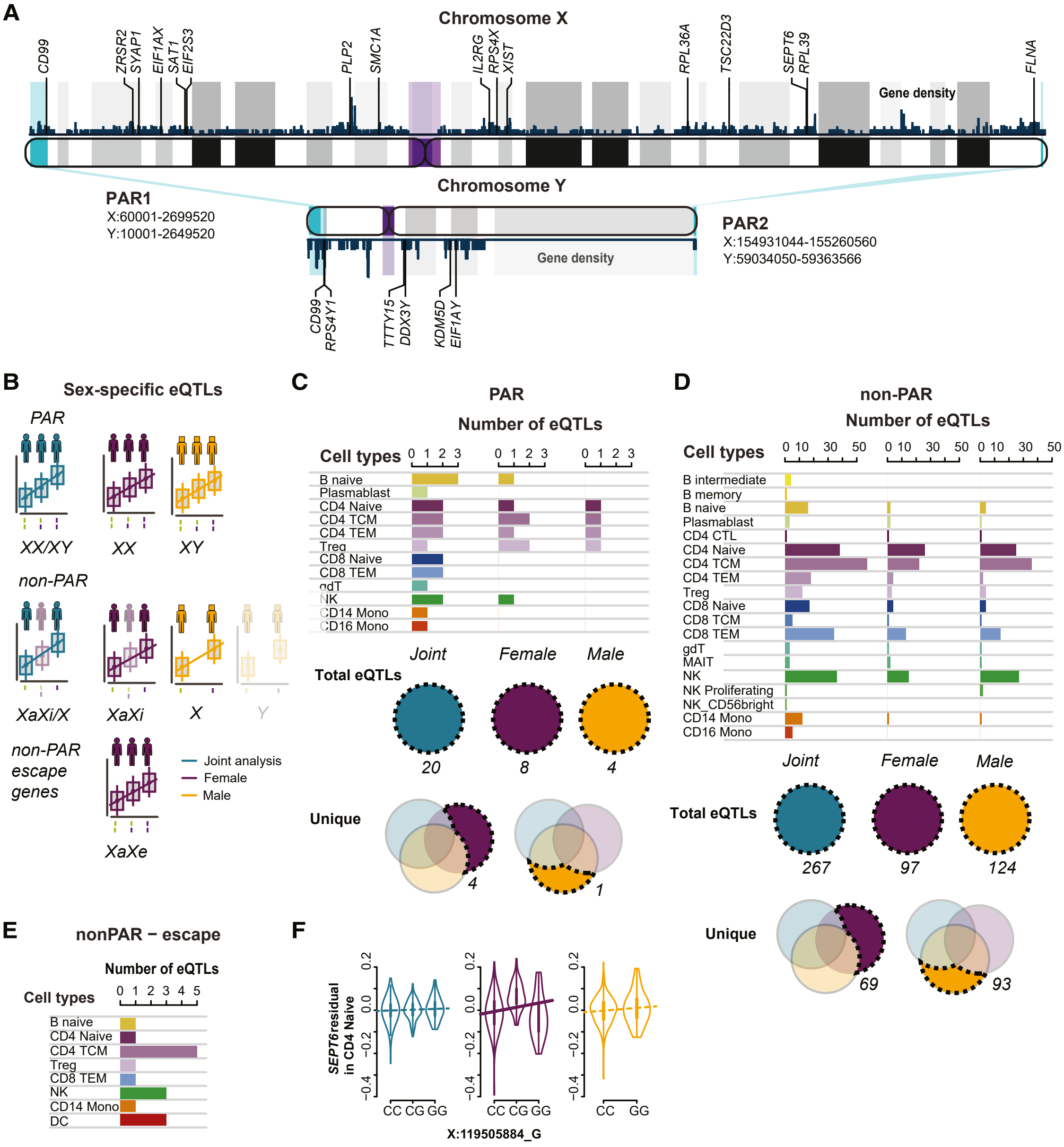

Figure 6. Sex-chromosome eQTL analysis

(A) Ideograms of the X and Y chromosomes showing PAR and non-PAR regions (cyan) and centromere (purple). Gene-density (navy) histogram across the chromosomes, along with highlighted genes of interest on the X and Y.

(B) Sex-specific eQTL models of the sex chromosomes split into the PAR, non-PAR, and X escapee genes. Each model highlights the joint analysis (turquoise) and the sex stratified (females in purple and males in gold). PAR analysis is similar to the autosomes, where genes escape X-inactivation. In contrast, heterozygous alleles in females in the non-PAR analysis require knowledge of the inactive/active allele (light purple) as males are only ever homozygous.

(C–E) Total number of *cis*-eQTLs by cell type in the (C) PAR regions, (D) non-PAR regions, and (E) non-PAR escape genes.

(F) Example gene *SEPT6* with a female-specific eQTL and female-biased DEG.

same time, three were female-specific (e.g., *TMEM255A*) and three X-escape genes (*PNPLA4* [MIM: 300102], *OFD1* [MIM: 300170], and *EIF2S3* [MIM: 300161]) were not tested in males.

In addition to the X non-PAR, the Y chromosome has a non-PAR spanning 56 Mbp and containing 102 genes. In our data, we genotyped ~7,000 SNPs, of which 3,243 remained after QC. As no recombination occurs on the Y, imputation here was difficult and unlikely to be informative. We attempted to use the haplogroups of the Y chromosomes[41]; however, all the males were classified as belonging to major haplogroups BT-M8947 and BT-M8949, which differ by one variant each and thus there is not enough variation to test for *cis*-eQTLs.

In cases where genes are differentially expressed by sex on the sex chromosomes, this may be due to genetic control. To test this, we again looked for an overlap between

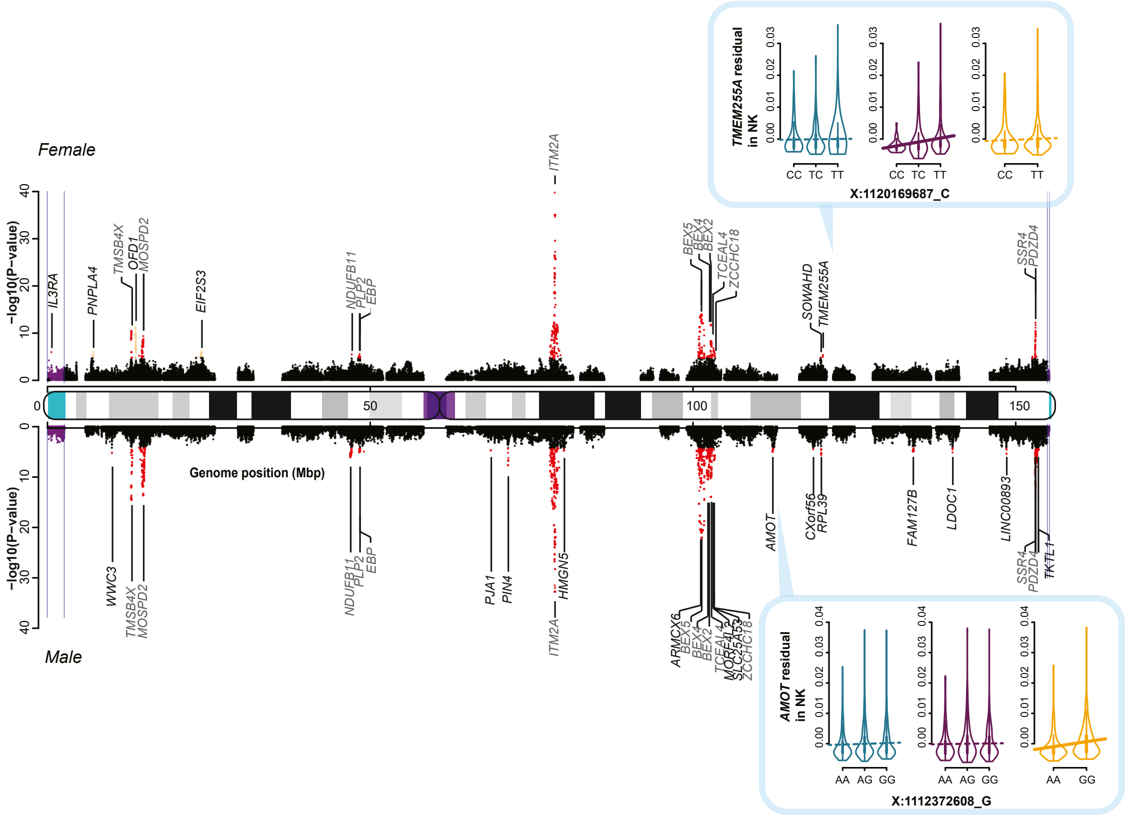

Figure 7. Example Manhattan plots of sex-chromosome eQTLs for NK cells

Top female, bottom males, with female specific (*TMEM255A*) and male specific (*AMOT*) as examples. The x axis is the chromosome position, while the y axis shows the $-\log10(p$ value) at that position.

the eQTLs and sex-DEGs (Tables S20 and S21). Among the PAR X chromosome genes, we observed no overlap between eGenes and sex-DEGs. In the non-PAR X, *SEPT6* (MIM: 300683) was an eGene with both female-biased expression and an eQTL in CD4$^+$ naive and CD4$^+$ TCMs (Figure 6F). *SEPT6*, a septin GTPase, plays a role in T cell migration[83] and may potentially escape XCI.[84] Of the escape genes, *RPS4X*, *XIST*, and *EIF2S3* had both eQTLs and female-biased expression in a few of the cell types (B naive, CD4$^+$ naive, CD4$^+$ TCM, DC, and Treg). As in the autosomal analysis, no male-specific eQTLs overlapped male-biased expression. Together, these results indicate higher immune gene expression in females at baseline, reflected primarily in more female-biased expression but also alternate regulatory control, likely linked to X-inactivation and escape.

Discussion

Our analysis of sex differences in PBMCs highlights the importance of studying the immune system in a sex-specific manner. Despite known differences in the immune systems of males and females being recorded, many researchers still study and assess their functions, pathways, gene expression, and genetic regulation agnostic of sex. This work examined these differences in a dataset of close to 1,000 individuals and found that small differences in cell-type proportions do exist between the sexes. These differences are likely to underpin functional effects, such as heightened responses to foreign stimuli (e.g., monocytes) and autoimmune reactivity (e.g., T and B cells).

In addition to differences in the cellular landscape between male and female individuals, we also identified sex-specific differences in gene expression. Expectedly, many of these genes were on the sex chromosomes. However, the differentially expressed functions of these genes remain open to investigation. Ribosomal genes that exhibit male-biased expression may be linked to differences in proliferation, cell activation, and cellular exhaustion, common mechanisms in cancer. In contrast, the number of genes that showed sex-biased expression in females was linked to immune pathways, highlighting downstream activity likely to be influenced in activated or stimulated immune systems.

Through a comprehensive evaluation of the effect of sex and the X chromosome on control of gene expression in a cell-type-specific manner, we identified numerous sex-specific eQTLs that were not previously observed in bulk whole-blood studies. The impact of genetic variation on gene expression on the X chromosome differed from that on autosomes, with fewer eQTLs overall and lower effect sizes. Moreover, the X-chromosome eQTLs were less likely to be shared between cell types. These findings align

with previous studies and support the hypothesis that a more efficient purifying selection on the X is present compared to autosomes.[61] When considering the functional enrichment of sex-specific DEGs and eQTLs, we found that male-specific DEGs and eGenes were related to non-reproductive-system cancers (e.g., lung). In contrast, female-specific ones were generally involved in immunological pathways.

However, our findings have their limitations. In addition to differences in sex, several other factors can influence gene expression. For example, immune responses change over a lifetime; as we age, B cells increase in females, while Tregs increase in males. Furthermore, there is considerable evidence that parity[85,86] and hormonal changes with menopause impact gene-expression levels in mammary glands, which can change the risk of breast cancer. These changes may also have affected circulating immune cells and should therefore be considered in future studies. A further caveat to this analysis is that none of these cells were stimulated or activated by any infectious trigger, and the effect sizes we observe reflect baseline differences. In other conditions, these small differences may be exacerbated. Additionally, because we have collected our data at a single time point, any fluctuations in hormone levels or dynamic/periodic changes in gene expression, such as circadian rhythms, will be missed or averaged out. Future work to assess the influence of infections, stress, or other environmental factors may show additional or larger effect sizes.

There is a well observed disparity in disease prevalence between sexes with autoimmune diseases being more common in females.[1] Overall, our results suggest that genes with sex differences are involved in immunologically important functions, showing higher overall activity in females. These results highlight that genes that are sexually dimorphic at baseline could potentially vary in their response in immune disease between the sexes in a cell-type-specific way.

Data and code availability

The datasets and code generated during this study are available on Zenodo: https://doi.org/10.5281/zenodo.19210998 (https://zenodo.org/records/19210998) or on GitHub: https://github.com/ballouzlab/sex_diffs.

Acknowledgments

This research was supported by a National Health and Medical Research Council Research Fellowship and MS Australia Postdoctoral Fellowship (S.Y.), Leader Fellowship (A.W.H., 2009079), Career Development Fellowship (J.E.P., 1107599), and Investigator Fellowship (J.E.P., 1175781). K.A.F. is supported by the Alex Gadomski Fellowship, funded by Maddie Riewoldt's Vision. Additional grant support was provided by the National Health and Medical Research Council (1150144, 1143163, and 2020517), the Australian Research Council (180101405), and the Royal Hobart Hospital Research Foundation. The content is solely the responsibility of the authors and does not necessarily represent the official views of the funding agents. The funders had no role in study design, data collection and analysis, decision to publish, or preparation of the manuscript.

Declaration of interests

The authors declare no competing interests.

Web resources

Gene Expression Omnibus (GSE196830), https://www.ncbi.nlm.nih.gov/geo/query/acc.cgi?acc=GSE196830

GitHub, https://github.com/powellgenomicslab/onek1k_phase1

Human Cell Atlas (HCA), https://cellxgene.cziscience.com/collections/dde06e0f-ab3b-46be-96a2-a8082383c4a1

Supplemental information

Supplemental information can be found online at https://doi.org/10.1016/j.ajhg.2026.04.003.

Received: January 27, 2025
Accepted: April 3, 2026
Published: May 7, 2026

References

1. Klein, S.L., and Flanagan, K.L. (2016). Sex differences in immune responses. Nat. Rev. Immunol. *16*, 626–638. https://doi.org/10.1038/nri.2016.90.
2. Wilkinson, N.M., Chen, H.-C., Lechner, M.G., and Su, M.A. (2022). Sex Differences in Immunity. Annu. Rev. Immunol. *40*, 75–94. https://doi.org/10.1146/annurev-immunol-101320-125133.
3. Abdullah, M., Chai, P.-S., Chong, M.-Y., Tohit, E.R.M., Ramasamy, R., Pei, C.P., and Vidyadaran, S. (2012). Gender effect on in vitro lymphocyte subset levels of healthy individuals. Cell. Immunol. *272*, 214–219. https://doi.org/10.1016/j.cellimm.2011.10.009.
4. Hewagama, A., Patel, D., Yarlagadda, S., Strickland, F.M., and Richardson, B.C. (2009). Stronger inflammatory/cytotoxic T-cell response in women identified by microarray analysis. Genes Immun. *10*, 509–516. https://doi.org/10.1038/gene.2009.12.
5. Fish, E.N. (2008). The X-files in immunity: sex-based differences predispose immune responses. Nat. Rev. Immunol. *8*, 737–744. https://doi.org/10.1038/nri2394.
6. Yacoub Wasef, S.Z. (2004). Gender differences in systemic lupus erythematosus. Gend. Med. *1*, 12–17. https://doi.org/10.1016/S1550-8579(04)80006-8.
7. Yazar, S., Alquicira-Hernandez, J., Wing, K., Senabouth, A., Gordon, M.G., Andersen, S., Lu, Q., Rowson, A., Taylor, T.R.P., Clarke, L., et al. (2022). Single-cell eQTL mapping identifies cell type–specific genetic control of autoimmune disease. Science *376*, eabf3041. https://doi.org/10.1126/science.abf3041.
8. Das, S., Forer, L., Schönherr, S., Sidore, C., Locke, A.E., Kwong, A., Vrieze, S.I., Chew, E.Y., Levy, S., McGue, M., et al. (2016). Next-generation genotype imputation service and methods. Nat. Genet. *48*, 1284–1287. https://doi.org/10.1038/ng.3656.

9. Fuchsberger, C., Abecasis, G.R., and Hinds, D.A. (2015). minimac2: faster genotype imputation. Bioinformatics *31*, 782–784. https://doi.org/10.1093/bioinformatics/btu704.
10. McCarthy, S., Das, S., Kretzschmar, W., Delaneau, O., Wood, A.R., Teumer, A., Kang, H.M., Fuchsberger, C., Danecek, P., Sharp, K., et al. (2016). A reference panel of 64,976 haplotypes for genotype imputation. Nat. Genet. *48*, 1279–1283. https://doi.org/10.1038/ng.3643.
11. Zheng, G.X.Y., Terry, J.M., Belgrader, P., Ryvkin, P., Bent, Z.W., Wilson, R., Ziraldo, S.B., Wheeler, T.D., McDermott, G.P., Zhu, J., et al. (2017). Massively parallel digital transcriptional profiling of single cells. Nat. Commun. *8*, 14049. https://doi.org/10.1038/ncomms14049.
12. Dobin, A., Davis, C.A., Schlesinger, F., Drenkow, J., Zaleski, C., Jha, S., Batut, P., Chaisson, M., and Gingeras, T.R. (2013). STAR: ultrafast universal RNA-seq aligner. Bioinformatics *29*, 15–21. https://doi.org/10.1093/bioinformatics/bts635.
13. Hao, Y., Hao, S., Andersen-Nissen, E., Mauck, W.M., 3rd, Zheng, S., Butler, A., Lee, M.J., Wilk, A.J., Darby, C., Zager, M., et al. (2021). Integrated analysis of multimodal single-cell data. Cell *184*, 3573–3587.e29. https://doi.org/10.1016/j.cell.2021.04.048.
14. Bakken, T.E., Jorstad, N.L., Hu, Q., Lake, B.B., Tian, W., Kalmbach, B.E., Crow, M., Hodge, R.D., Krienen, F.M., Sorensen, S.A., et al. (2021). Comparative cellular analysis of motor cortex in human, marmoset and mouse. Nature *598*, 111–119. https://doi.org/10.1038/s41586-021-03465-8.
15. Phipson, B., Sim, C.B., Porrello, E.R., Hewitt, A.W., Powell, J., and Oshlack, A. (2022). Propeller: testing for differences in cell type proportions in single cell data. Bioinformatics *38*, 4720–4726. https://doi.org/10.1093/bioinformatics/btac582.
16. Lin, X., Chau, C., Ma, K., Huang, Y., and Ho, J.W.K. (2023). DCATS: differential composition analysis for flexible single-cell experimental designs. Genome Biol. *24*, 151. https://doi.org/10.1186/s13059-023-02980-3.
17. R Core Team (2021). R: A Language and Environment for Statistical Computing (R Foundation for Statistical Computing).
18. Finak, G., McDavid, A., Yajima, M., Deng, J., Gersuk, V., Shalek, A.K., Slichter, C.K., Miller, H.W., McElrath, M.J., Prlic, M., et al. (2015). MAST: a flexible statistical framework for assessing transcriptional changes and characterizing heterogeneity in single-cell RNA sequencing data. Genome Biol. *16*, 278. https://doi.org/10.1186/s13059-015-0844-5.
19. Urbut, S.M., Wang, G., Carbonetto, P., and Stephens, M. (2019). Flexible statistical methods for estimating and testing effects in genomic studies with multiple conditions. Nat. Genet. *51*, 187–195. https://doi.org/10.1038/s41588-018-0268-8.
20. Alquicira-Hernandez, J., and Powell, J.E. (2021). Nebulosa recovers single-cell gene expression signals by kernel density estimation. Bioinformatics *37*, 2485–2487. https://doi.org/10.1093/bioinformatics/btab003.
21. Ballouz, S., Weber, M., Pavlidis, P., and Gillis, J. (2017). EGAD: ultra-fast functional analysis of gene networks. Bioinformatics *33*, 612–614. https://doi.org/10.1093/bioinformatics/btw695.
22. Ashburner, M., Ball, C.A., Blake, J.A., Botstein, D., Butler, H., Cherry, J.M., Davis, A.P., Dolinski, K., Dwight, S.S., Eppig, J.T., et al. (2000). Gene ontology: tool for the unification of biology. The Gene Ontology Consortium. Nat. Genet. *25*, 25–29. https://doi.org/10.1038/75556.
23. Gene Ontology Consortium (2021). The Gene Ontology resource: enriching a GOld mine. Nucleic Acids Res. *49*, D325–D334. https://doi.org/10.1093/nar/gkaa1113.
24. Subramanian, A., Tamayo, P., Mootha, V.K., Mukherjee, S., Ebert, B.L., Gillette, M.A., Paulovich, A., Pomeroy, S.L., Golub, T.R., Lander, E.S., and Mesirov, J.P. (2005). Gene set enrichment analysis: a knowledge-based approach for interpreting genome-wide expression profiles. Proc. Natl. Acad. Sci. USA *102*, 15545–15550. https://doi.org/10.1073/pnas.0506580102.
25. Liberzon, A., Birger, C., Thorvaldsdóttir, H., Ghandi, M., Mesirov, J.P., and Tamayo, P. (2015). The Molecular Signatures Database (MSigDB) hallmark gene set collection. Cell Syst. *1*, 417–425. https://doi.org/10.1016/j.cels.2015.12.004.
26. Kanehisa, M., and Goto, S. (2000). KEGG: Kyoto Encyclopedia of Genes and Genomes. Nucleic Acids Res. *28*, 27–30. https://doi.org/10.1093/nar/28.1.27.
27. Gillespie, M., Jassal, B., Stephan, R., Milacic, M., Rothfels, K., Senff-Ribeiro, A., Griss, J., Sevilla, C., Matthews, L., Gong, C., et al. (2022). The reactome pathway knowledgebase 2022. Nucleic Acids Res. *50*, D687–D692. https://doi.org/10.1093/nar/gkab1028.
28. Nishimura, D. (2001). BioCarta. Biotech softw. Internet rep. *2*, 117–120. https://doi.org/10.1089/152791601750294344.
29. Rouillard, A.D., Gundersen, G.W., Fernandez, N.F., Wang, Z., Monteiro, C.D., McDermott, M.G., and Ma'ayan, A. (2016). The harmonizome: a collection of processed datasets gathered to serve and mine knowledge about genes and proteins. Database *2016*, baw100. https://doi.org/10.1093/database/baw100.
30. ENCODE Project Consortium (2011). A user's guide to the encyclopedia of DNA elements (ENCODE). PLoS Biol. *9*, e1001046. https://doi.org/10.1371/journal.pbio.1001046.
31. Jansen, R., Batista, S., Brooks, A.I., Tischfield, J.A., Willemsen, G., van Grootheest, G., Hottenga, J.-J., Milaneschi, Y., Mbarek, H., Madar, V., et al. (2014). Sex differences in the human peripheral blood transcriptome. BMC Genom. *15*, 33. https://doi.org/10.1186/1471-2164-15-33.
32. Melé, M., Ferreira, P.G., Reverter, F., DeLuca, D.S., Monlong, J., Sammeth, M., Young, T.R., Goldmann, J.M., Pervouchine, D.D., Sullivan, T.J., et al. (2015). The human transcriptome across tissues and individuals. Science *348*, 660–665. https://doi.org/10.1126/science.aaa0355.
33. Tukiainen, T., Villani, A.-C., Yen, A., Rivas, M.A., Marshall, J.L., Satija, R., Aguirre, M., Gauthier, L., Fleharty, M., Kirby, A., et al. (2017). Landscape of X chromosome inactivation across human tissues. Nature *550*, 244–248. https://doi.org/10.1038/nature24265.
34. Schmiedel, B.J., Singh, D., Madrigal, A., Valdovino-Gonzalez, A.G., White, B.M., Zapardiel-Gonzalo, J., Ha, B., Altay, G., Greenbaum, J.A., McVicker, G., et al. (2018). Impact of Genetic Polymorphisms on Human Immune Cell Gene Expression. Cell *175*, 1701–1715.e16. https://doi.org/10.1016/j.cell.2018.10.022.
35. Bongen, E., Lucian, H., Khatri, A., Fragiadakis, G.K., Bjornson, Z.B., Nolan, G.P., Utz, P.J., and Khatri, P. (2019). Sex Differences in the Blood Transcriptome Identify Robust Changes in Immune Cell Proportions with Aging and Influenza Infection. Cell Rep. *29*, 1961–1973.e4. https://doi.org/10.1016/j.celrep.2019.10.019.
36. Oliva, M., Muñoz-Aguirre, M., Kim-Hellmuth, S., Wucher, V., Gewirtz, A.D.H., Cotter, D.J., Parsana, P., Kasela, S., Balliu, B.,

Viñuela, A., et al. (2020). The impact of sex on gene expression across human tissues. Science *369*, eaba3066. https://doi.org/10.1126/science.aba3066.
37. Harrow, J., Frankish, A., Gonzalez, J.M., Tapanari, E., Diekhans, M., Kokocinski, F., Aken, B.L., Barrell, D., Zadissa, A., Searle, S., et al. (2012). GENCODE: The reference human genome annotation for The ENCODE Project. Genome Res. *22*, 1760–1774.
38. Choudhary, S., and Satija, R. (2022). Comparison and evaluation of statistical error models for scRNA-seq. Genome Biol. *23*, 27. https://doi.org/10.1186/s13059-021-02584-9.
39. Storey, J.D., and Tibshirani, R. (2003). Statistical significance for genomewide studies. Proc. Natl. Acad. Sci. USA *100*, 9440–9445. https://doi.org/10.1073/pnas.1530509100.
40. Shabalin, A.A. (2012). Matrix eQTL: ultra fast eQTL analysis via large matrix operations. Bioinformatics *28*, 1353–1358. https://doi.org/10.1093/bioinformatics/bts163.
41. Poznik, G.D. (2016). Identifying Y-chromosome haplogroups in arbitrarily large samples of sequenced or genotyped men. Preprint at bioRxiv. https://doi.org/10.1101/088716.
42. Al-Attar, A., Presnell, S.R., Peterson, C.A., Thomas, D.T., and Lutz, C.T. (2016). The effect of sex on immune cells in healthy aging: Elderly women have more robust natural killer lymphocytes than do elderly men. Mech. Ageing Dev. *156*, 25–33. https://doi.org/10.1016/j.mad.2016.04.001.
43. Li, M., Yao, D., Zeng, X., Kasakovski, D., Zhang, Y., Chen, S., Zha, X., Li, Y., and Xu, L. (2019). Age related human T cell subset evolution and senescence. Immun. Ageing *16*, 24. https://doi.org/10.1186/s12979-019-0165-8.
44. Robinson, G.A., Peng, J., Peckham, H., Butler, G., Pineda-Torra, I., Ciurtin, C., and Jury, E.C. (2022). Investigating sex differences in T regulatory cells from cisgender and transgender healthy individuals and patients with autoimmune inflammatory disease: a cross-sectional study. Lancet Rheumatol. *4*, e710–e724. https://doi.org/10.1016/s2665-9913(22)00198-9.
45. Huang, Z., Chen, B., Liu, X., Li, H., Xie, L., Gao, Y., Duan, R., Li, Z., Zhang, J., Zheng, Y., and Su, W. (2021). Effects of sex and aging on the immune cell landscape as assessed by single-cell transcriptomic analysis. Proc. Natl. Acad. Sci. USA *118*, e2023216118. https://doi.org/10.1073/pnas.2023216118.
46. Bellamy, G.J., Hinchliffe, R.F., Crawshaw, K.C., Finn, A., and Bell, F. (2000). Total and differential leucocyte counts in infants at 2, 5 and 13 months of age. Clin. Lab. Haematol. *22*, 81–87. https://doi.org/10.1046/j.1365-2257.2000.00288.x.
47. Chen, Y., Zhang, Y., Zhao, G., Chen, C., Yang, P., Ye, S., and Tan, X. (2016). Difference in Leukocyte Composition between Women before and after Menopausal Age, and Distinct Sexual Dimorphism. PLoS One *11*, e0162953. https://doi.org/10.1371/journal.pone.0162953.
48. Puissant-Lubrano, B., Apoil, P.A., Guedj, K., Congy-Jolivet, N., Roubinet, F., Guyonnet, S., Sourdet, S., Nourhashemi, F., and Blancher, A. (2018). Distinct effect of age, sex, and CMV seropositivity on dendritic cells and monocytes in human blood. Immunol. Cell Biol. *96*, 114–120. https://doi.org/10.1111/imcb.1004.
49. Jiang, W., Zhang, L., Lang, R., Li, Z., and Gilkeson, G. (2014). Sex Differences in Monocyte Activation in Systemic Lupus Erythematosus (SLE). PLoS One *9*, e114589. https://doi.org/10.1371/journal.pone.0114589.
50. Kverneland, A.H., Streitz, M., Geissler, E., Hutchinson, J., Vogt, K., Boës, D., Niemann, N., Pedersen, A.E., Schlickeiser, S., and Sawitzki, B. (2016). Age and gender leucocytes variances and references values generated using the standardized ONE-Study protocol. Cytometry. A. *89*, 543–564. https://doi.org/10.1002/cyto.a.22855.
51. Arruvito, L., Sanz, M., Banham, A.H., and Fainboim, L. (2007). Expansion of CD4+CD25+and FOXP3+ regulatory T cells during the follicular phase of the menstrual cycle: implications for human reproduction. J. Immunol. *178*, 2572–2578. https://doi.org/10.4049/jimmunol.178.4.2572.
52. Sopena-Rios, M., Ripoll-Cladellas, A., Omidi, F., Ballouz, S., Alquicira-Hernandez, J., Oelen, R., Hewitt, A.W., Franke, L., van der Wijst, M.G.P., Powell, J.E., and Melé, M. (2026). Single-cell analysis of the human immune system reveals sex-specific dynamics of immunosenescence. Nature Aging. https://doi.org/10.1038/s43587-026-01099-x.
53. Churov, A.V., Mamashov, K.Y., and Novitskaia, A.V. (2020). Homeostasis and the functional roles of CD4+ Treg cells in aging. Immunol. Lett. *226*, 83–89. https://doi.org/10.1016/j.imlet.2020.07.004.
54. Barnabei, L., Laplantine, E., Mbongo, W., Rieux-Laucat, F., and Weil, R. (2021). NF-κB: At the Borders of Autoimmunity and Inflammation. Front. Immunol. *12*, 716469. https://doi.org/10.3389/fimmu.2021.716469.
55. Grosche, L., Knippertz, I., König, C., Royzman, D., Wild, A.B., Zinser, E., Sticht, H., Muller, Y.A., Steinkasserer, A., and Lechmann, M. (2020). The CD83 Molecule – An Important Immune Checkpoint. Front. Immunol. *11*, 721. https://doi.org/10.3389/fimmu.2020.00721.
56. So, J., Tai, A.K., Lichtenstein, A.H., Wu, D., and Lamon-Fava, S. (2021). Sexual dimorphism of monocyte transcriptome in individuals with chronic low-grade inflammation. Biol. Sex Differ. *12*, 43. https://doi.org/10.1186/s13293-021-00387-y.
57. Bhatia, A., Sekhon, H.K., and Kaur, G. (2014). Sex hormones and immune dimorphism. Sci. World J. *2014*, 159150. https://doi.org/10.1155/2014/159150.
58. Daily, K., Patel, V.R., Rigor, P., Xie, X., and Baldi, P. (2011). MotifMap: integrative genome-wide maps of regulatory motif sites for model species. BMC Bioinf. *12*, 495. https://doi.org/10.1186/1471-2105-12-495.
59. Mor, G., Sapi, E., Abrahams, V.M., Rutherford, T., Song, J., Hao, X.-Y., Muzaffar, S., and Kohen, F. (2003). Interaction of the estrogen receptors with the Fas ligand promoter in human monocytes. J. Immunol. *170*, 114–122. https://doi.org/10.4049/jimmunol.170.1.114.
60. Dimas, A.S., Nica, A.C., Montgomery, S.B., Stranger, B.E., Raj, T., Buil, A., Giger, T., Lappalainen, T., Gutierrez-Arcelus, M., et al.; MuTHER Consortium (2012). Sex-biased genetic effects on gene regulation in humans. Genome Res. *22*, 2368–2375. https://doi.org/10.1101/gr.134981.111.
61. Kukurba, K.R., Parsana, P., Balliu, B., Smith, K.S., Zappala, Z., Knowles, D.A., Favé, M.-J., Davis, J.R., Li, X., Zhu, X., et al. (2016). Impact of the X Chromosome and sex on regulatory variation. Genome Res. *26*, 768–777. https://doi.org/10.1101/gr.197897.115.
62. Jones, A.G., Connelly, G.G., Dalapati, T., Wang, L., Schott, B.H., San Roman, A.K., and Ko, D.C. (2024). Biological sex affects functional variation across the human genome. Preprint at medRxiv. https://doi.org/10.1101/2024.09.03.24313025.
63. Tomofuji, Y., Edahiro, R., Sonehara, K., Shirai, Y., Kock, K.H., Wang, Q.S., Namba, S., Moody, J., Ando, Y., Suzuki, A., et al. (2024). Quantification of escape from X chromosome inactivation with single-cell omics data reveals heterogeneity

across cell types and tissues. Cell Genom. *4*, 100625. https://doi.org/10.1016/j.xgen.2024.100625.
64. Porcu, E., Claringbould, A., Weihs, A., Lepik, K., , et al.; BIOS Consortium, Richardson, T.G., Völker, U., Santoni, F.A., Teumer, A., Franke, L. (2022). Limited evidence for blood eQTLs in human sexual dimorphism. Genome Med. *14*, 89. https://doi.org/10.1186/s13073-022-01088-w.
65. Bandala-Sanchez, E., Zhang, Y., Reinwald, S., Dromey, J.A., Lee, B.-H., Qian, J., Böhmer, R.M., and Harrison, L.C. (2013). T cell regulation mediated by interaction of soluble CD52 with the inhibitory receptor Siglec-10. Nat. Immunol. *14*, 741–748. https://doi.org/10.1038/ni.2610.
66. Planells, B., Gómez-Redondo, I., Pericuesta, E., Lonergan, P., and Gutiérrez-Adán, A. (2019). Differential isoform expression and alternative splicing in sex determination in mice. BMC Genom. *20*, 202. https://doi.org/10.1186/s12864-019-5572-x.
67. Tschopp, J., Martinon, F., and Burns, K. (2003). NALPs: a novel protein family involved in inflammation. Nat. Rev. Mol. Cell Biol. *4*, 95–104. https://doi.org/10.1038/nrm1019.
68. Kuchmiy, A.A., D'Hont, J., Hochepied, T., and Lamkanfi, M. (2016). NLRP2 controls age-associated maternal fertility. J. Exp. Med. *213*, 2851–2860. https://doi.org/10.1084/jem.20160900.
69. Tilburgs, T., Meissner, T.B., Ferreira, L.M.R., Mulder, A., Musunuru, K., Ye, J., and Strominger, J.L. (2017). NLRP2 is a suppressor of NF-κB signaling and HLA-C expression in human trophoblasts. Biol. Reprod. *96*, 831–842. https://doi.org/10.1093/biolre/iox009.
70. Rembeck, K., Waldenström, J., Hellstrand, K., Nilsson, S., Nyström, K., Martner, A., Lindh, M., Norkrans, G., Westin, J., Pedersen, C., et al. (2014). Variants of the inosine triphosphate pyrophosphatase gene are associated with reduced relapse risk following treatment for HCV genotype 2/3. Hepatology *59*, 2131–2139. https://doi.org/10.1002/hep.27009.
71. Chinen, T., Kannan, A.K., Levine, A.G., Fan, X., Klein, U., Zheng, Y., Gasteiger, G., Feng, Y., Fontenot, J.D., and Rudensky, A.Y. (2016). An essential role for the IL-2 receptor in T cell function. Nat. Immunol. *17*, 1322–1333. https://doi.org/10.1038/ni.3540.
72. Grier, J.T., Forbes, L.R., Monaco-Shawver, L., Oshinsky, J., Atkinson, T.P., Moody, C., Pandey, R., Campbell, K.S., and Orange, J.S. (2012). Human immunodeficiency-causing mutation defines CD16 in spontaneous NK cell cytotoxicity. J. Clin. Investig. *122*, 3769–3780. https://doi.org/10.1172/JCI64837.
73. Wang, T.T., Sewatanon, J., Memoli, M.J., Wrammert, J., Bournazos, S., Bhaumik, S.K., Pinsky, B.A., Chokephaibulkit, K., Onlamoon, N., Pattanapanyasat, K., et al. (2017). IgG antibodies to dengue enhanced for FcγRIIIA binding determine disease severity. Science *355*, 395–398. https://doi.org/10.1126/science.aai8128.
74. Vietzen, H., Danklmaier, V., Zoufaly, A., and Puchhammer-Stöckl, E. (2022). High-affinity FcγRIIIa genetic variants and potent NK cell-mediated antibody-dependent cellular cytotoxicity (ADCC) responses contributing to severe COVID-19. Genet. Med. *24*, 1449–1458. https://doi.org/10.1016/j.gim.2022.04.005.
75. Zhu, X.-W., Wang, Y., Wei, Y.-H., Zhao, P.-P., Wang, X.-B., Rong, J.-J., Zhong, W.-Y., Zhang, X.-W., Wang, L., and Zheng, H.-F. (2016). Comprehensive Assessment of the Association between FCGRs polymorphisms and the risk of systemic lupus erythematosus: Evidence from a Meta-Analysis. Sci. Rep. *6*, 31617. https://doi.org/10.1038/srep31617.
76. Chen, S., Wen, X., Li, J., Li, Y., Li, L., Tian, X., Yuan, H., Zhang, F., and Li, Y. (2017). Association of FCGR2A/FCGR3A variant rs2099684 with Takayasu arteritis in the Han Chinese population. Oncotarget *8*, 17239–17245. https://doi.org/10.18632/oncotarget.12738.
77. Immune cell - ITGB2 - The Human Protein Atlas. https://www.proteinatlas.org/ENSG00000160255-ITGB2/immune+cell.
78. Xu, D., Li, T., Wang, R., and Mu, R. (2021). Expression and Pathogenic Analysis of Integrin Family Genes in Systemic Sclerosis. Front. Med. *8*, 674523. https://doi.org/10.3389/fmed.2021.674523.
79. Cui, Y., Zhang, H., Wang, Z., Gong, B., Al-Ward, H., Deng, Y., Fan, O., Wang, J., Zhu, W., and Sun, Y.E. (2023). Exploring the shared molecular mechanisms between systemic lupus erythematosus and primary Sjögren's syndrome based on integrated bioinformatics and single-cell RNA-seq analysis. Front. Immunol. *14*, 1212330. https://doi.org/10.3389/fimmu.2023.1212330.
80. Yao, C., Joehanes, R., Johnson, A.D., Huan, T., Esko, T., Ying, S., Freedman, J.E., Murabito, J., Lunetta, K.L., Metspalu, A., et al. (2014). Sex- and age-interacting eQTLs in human complex diseases. Hum. Mol. Genet. *23*, 1947–1956. https://doi.org/10.1093/hmg/ddt582.
81. Meester, I., Manilla-Muñoz, E., León-Cachón, R.B.R., Paniagua-Frausto, G.A., Carrión-Alvarez, D., Ruiz-Rodríguez, C.O., Rodríguez-Rangel, X., and García-Martínez, J.M. (2020). SeXY chromosomes and the immune system: reflections after a comparative study. Biol. Sex Differ. *11*, 3. https://doi.org/10.1186/s13293-019-0278-y.
82. Butler-Laporte, G., Gonzalez-Kozlova, E., Su, C.-Y., Zhou, S., Nakanishi, T., Brunet-Ratnasingham, E., Morrison, D., Laurent, L., Afilalo, J., Afilalo, M., et al. (2022). The dynamic changes and sex differences of 147 immune-related proteins during acute COVID-19 in 580 individuals. Clin. Proteomics *19*, 34. https://doi.org/10.1186/s12014-022-09371-z.
83. Dolat, L., Hu, Q., and Spiliotis, E.T. (2014). Septin functions in organ system physiology and pathology. Biol. Chem. *395*, 123–141. https://doi.org/10.1515/hsz-2013-0233.
84. Shvetsova, E., Sofronova, A., Monajemi, R., Gagalova, K., Draisma, H.H.M., White, S.J., Santen, G.W.E., Chuva de Sousa Lopes, S.M., Heijmans, B.T., van Meurs, J., et al. (2019). Skewed X-inactivation is common in the general female population. Eur. J. Hum. Genet. *27*, 455–465. https://doi.org/10.1038/s41431-018-0291-3.
85. Verlinden, I., Güngör, N., Wouters, K., Janssens, J., Raus, J., and Michiels, L. (2005). Parity-induced changes in global gene expression in the human mammary gland. Eur. J. Cancer Prev. *14*, 129–137. https://doi.org/10.1097/00008469-200504000-00008.
86. Santucci-Pereira, J., Zeleniuch-Jacquotte, A., Afanasyeva, Y., Zhong, H., Slifker, M., Peri, S., Ross, E.A., López de Cicco, R., Zhai, Y., Nguyen, T., et al. (2019). Genomic signature of parity in the breast of premenopausal women. Breast Cancer Res. *21*, 46. https://doi.org/10.1186/s13058-019-1128-x.

The American Journal of Human Genetics, Volume *113*

Supplemental information

The impact of sex on the immune system explored at the single-cell level

Seyhan Yazar, Jose Alquicira-Hernandez, Kristof Wing, Anne Senabouth, Stacey Andersen, Kirsten A. Fairfax, Alex W. Hewitt, Joseph E. Powell, and Sara Ballouz

Supplemental Notes

Impact of age on sex differences in immune cell types

Cell type abundances change with age, and this occurs in a sex-specific manner [1; 2]. The sex-specific changes in cell proportions with age is linked to hormonal changes during puberty, menstruation and menopause[3] and loss of clonal diversity in stem cell populations[4]. When comparing proportions to recorded biological age **(Figure S1, Table S2**), we see clear correlation patterns in NK cells (Rho=0.32, FDR~0.002)[5] and CD8+ Naïve T-cells (Rho=-0.67, FDR< 0.001)[6], both of which change with age irrespective of sex. However, we see no significant correlation with Tregs with age which had been reported[7], instead NK proliferating cells, Plasmablasts, CD8+ TEM and gamma-delta T cells (gdT) are correlated with age in females (Rho NK proliferating=0.17 FDR~ 0.001, Rho CD8+ TEM=0.23, FDR~$3.7x10^{-7}$ and Rho gdT=-0.25, FDR~$4.4x10^{-8}$) but not in males.

Cell-type and sex-specific co-expression replicates known biological functions

In addition to genetic control, we also wished to check whether observed sex-biased expression was linked to co-variation (*i.e.,* co-expression or co-regulation). Genes that co-vary are believed to be co-functional, either under common regulatory control or are in common pathways. Like gene set enrichment analyses, this approach includes additional network information in the enrichment, defined as a gene-gene interaction. Thus, analysing co-expression gene-gene networks that are conditioned on properties of the underlying cell types, tissues or sex highlights modules or interactions that are specific to these conditions. To assess sex-specific networks derived from PBMCs, we built cell-type and sex-specific networks from the data. We performed differential co-expression to determine the sex and cell-type specific modules and gene sets that differ by sex and/or cell type (**Figure S7-10, Table S22**). To perform this robustly and remove confounding variables such as batch effects, we built separate networks per pool and aggregated these networks[8]. By conditioning on sex and cell-type, we aimed to remove co-variation confounded with these conditions and capture the intrinsic variation within each "state". We use the performance of the neighbour-voting algorithm in cross-validation (EGAD[9], **Methods**) to measure enrichment for known biological pathways and gene sets, with model performance measured by the averaged AUROC (area under the ROC curve) for each group across the *n*-folds (**Figure S7A**).

Using the Gene Ontology (GO [10; 11]) slim gene sets to measure broad biological functions first, we find similar average AUROC scores between the sexes of the same cell-type, with average scores varying based on cell-type (**Figure S7B,** AUROC~0.53-0.58). The joint aggregate networks show slightly higher performances than the sex-stratified networks, even with down-sampling, suggesting that some of the connections within these joint networks are lost when conditioning on sex – *i.e.*, sex has some influence on co-expression and contributes to the broad GO pathways and functions (AUROCs +0.02). We see that the individuals' network aggregates outperform the cell-type specific networks (AUROC~0.6), highlighting that cell-type composition (variation) drives a

fraction (+0.04) of the additional co-expression in these networks. Notably, these performances are still just above average, reflecting the sparsity in single-cell RNA-seq data, as many genes are not expressed or detected and could not be assessed. The co-expression analysis reaffirms, as in bulk data, that a large fraction of the co-expression we observe is driven by cellular composition [12]. Conditioning on cell type removes these connections in the network, impacting the AUROC performance. Sex, on the other hand, has much less of an effect, as removing sex-specific connections in the network does not impact the AUROC as much.

We then looked at the specific gene set module differences between the networks (**Figure S7C**). The highest performing gene set module in most of the cell-types was GO:0003735 (structural constituent of the ribosome, AUROC~0.79),yet had lower performances in NK proliferating and plasmablast cells (AUROC~0.61). On the other hand, NK proliferating and plasmablast cells co-express mitotic-related genes (GO GO:0000278, mitotic cell cycle AUROCs~0.75), while other cell types do not (AUROCs~0.5). To identify functional specificity further, we ran our analysis on additional gene sets from the Molecular Signature Database (MSigDB[13]), with a focus on the HALLMARK[14], KEGG[15], REACTOME[16] and BIOCARTA[17] gene sets and pathways. As before, we see cell-type specific networks scoring highly in particular pathways and gene sets related to their cell-type specific functions. Of note were monocyte-specific networks, where higher AUROCs in pathways linked to hypoxia, apoptosis and TNFα signaling were similar to the pathways found to be sex-differentially expressed in those cell-types (**Figure S10**). This suggests that these pathways and their genes show differential activity in monocytes [18; 19] dependent on sex.

In addition to pathways, co-expression may reflect co-regulation. Thus, we tested for TF-target enrichment within the networks, and whether we could observe differences in co-regulation between the sexes. We used the Gene Transcription Regulation Database (GTRD)[20]. TF-target genes dataset curated in MSigDB and observed highly correlated performances between the sexes once more (**Figure S7D**). The biggest differences were in the co-expression values of the target genes of *NR5A1* (steroidogenic factor 1 (SF-1)), a transcriptional activator involved in sex determination, and *CREBL2*[21], linked to adipose tissue differentiation. Whether these genes play roles in the immune system requires additional validation, but their roles in sex differences and sex-specific phenotypes (such as adipogenesis) are well established.

To further explore the performance differences, we looked at the topological network differences (i.e., gene-gene connections, **Figure S7E**) by comparing the pairwise co-expression ranked values of the cell-type specific aggregate networks. On average, ~16% of the edges change between the networks, with the most differences between the CD4+ T cell aggregates and the other cell types (~38%). Overall, we see that sex differences on average are low, as most cell-type specific networks have greater similarities to their same cell-type or similar cell-type within the hematopoietic hierarchy. To further measure the impact of sex and cell-type conditioning on the network topology, we then compared the change in overall connectivity per gene by assessing the node degree changes. As in the previous analysis, node degrees were similar between cell-type specific aggregates conditioned

on sex, with most differences appearing between the different cell types (**Figure S3F**). On average, less than 1% of genes (~99) have changes in their node degree standardized residuals of 3SDs across cell types (holding sex constant). In contrast, between the sexes of the same cell type, there are less than ~0.5% of genes that have significant node degree differences (~43 genes). We find these genes to be enriched for those on the sex chromosome, a few of which are paralogs (e.g., *UTY* and its X paralog *KDM6A/UTX*, *ZFY* and *ZFX, KDM5D* and *KDM5C*). X chromosome paralogs are likely more variable as they are known to escape X-inactivation. Overall, we observe little sex network differences from the autosomal genes, reaffirming our observation of few detectable changes in these networks as reflected by their similar AUROCs performance. This analysis highlights that sex differences have small effects on the broad biological pathways or functions in contrast to cell type, as highlighted by the more similar AUROCs in the former and specific gene set performances differences in the latter.

We identified core co-expressed gene-gene modules in the immune cell types along with gene expression differences. Our work highlighted differential co-expression changes between the sexes primarily a result of sex-chromosome genes, along with a few autosomal genes. The regulatory relationships that were impacted were not significant, but the differences do reflect changes to signalling pathways that are likely buffered in a sex-specific way. The Y chromosome is known to influence inflammatory pathways. As such, females have paralogs to several Y-specific genes that escape X-inactivation to compensate. Their co-expression partners and regulatory factors are likely shared, which we observe in our data. RNA-seq analysis may need to distinguish between the XY paralogs and hence expression estimates, and subsequently, co-expression relationships may be obscured or miscalculated.

Differential expression sensitivity analysis

To ensure our findings are not artifacts of approach or software implementation, we performed a sensitivity analysis. First, we applied Simes for global multiple test correction, which returned similar significant genes, except for cell types with low cell or individual counts which may have influenced the analysis's power (**Table S4**). To ensure that these were not only due to differences in power, we performed a downsampling analysis. We observed that most of these genes were robust to this process (**Table S7-8**). We further benchmarked our sex-DE results across Seurat v4.4.0 and Seurat v5.3.0. While Seurat v5 produced systematically larger absolute |log2FC| values due to updated pseudocount handling, the gene rank-order remained highly stable (**Figure S11**). Spearman correlation of |log2FC| values across all cell types was high (average rho = 0.88), confirming that the relative magnitude of sex-biased effects is preserved (**Figure S12**). Applying our significance thresholds (FDR < 0.05, |log2FC| > 0.1) to both versions yielded a 98% consensus overlap. Genes unique to v5 were typically marginal cases pushed over the fold-change threshold by version-specific scaling.

Supplemental Figures

Figure S1 Cell type proportions and age changes
Figure S2 Tests of normality of proportions for each cell type
Figure S3 Proportions compared across covariates
Figure S4 Downsampling differential expression analysis
Figure S5 Replication of unique DEGs in downsampling analysis
Figure S6 Sex-DEG enrichments
Figure S7 Co-expression by cell type and sex
Figure S8 Co-expression downsampling analysis
Figure S9 Differential co-expression comparisons
Figure S10 Functional enrichment results of aggregate networks using EGAD
Figure S11 DEG comparison between Seurat v4 and v5: log2FC
Figure S12 DEG comparison between Seurat v4 and v5: ranked log2FC

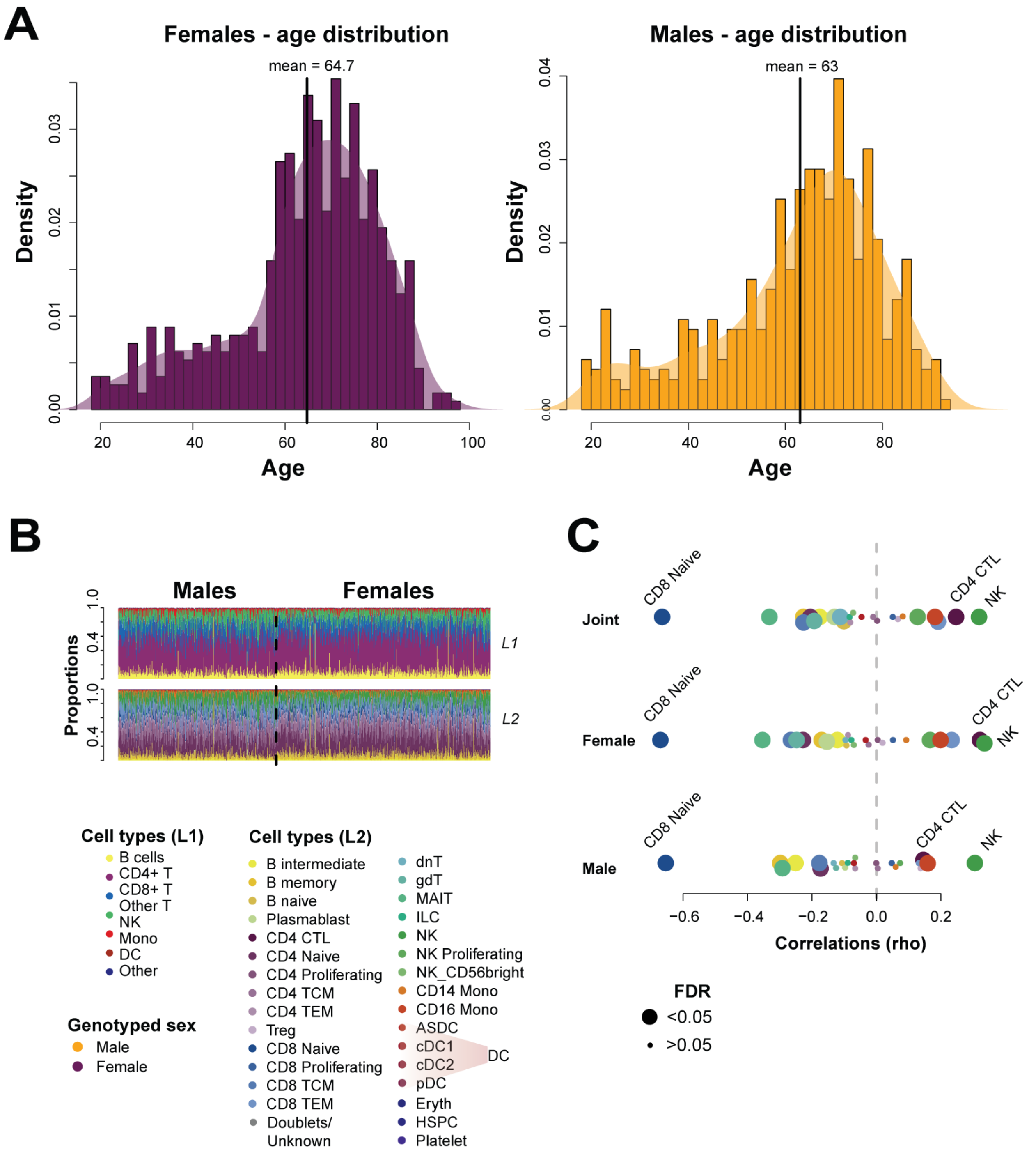

Figure S1 Cell-type proportions and age changes. (A) Distribution of ages for females and males in OneK1K. (B) Distributions of cell-type proportions for L1 (broad) classification and L2 classifications across all individuals. (C) Correlations of cell-type proportions to age for both sexes (joint), females only and males only.

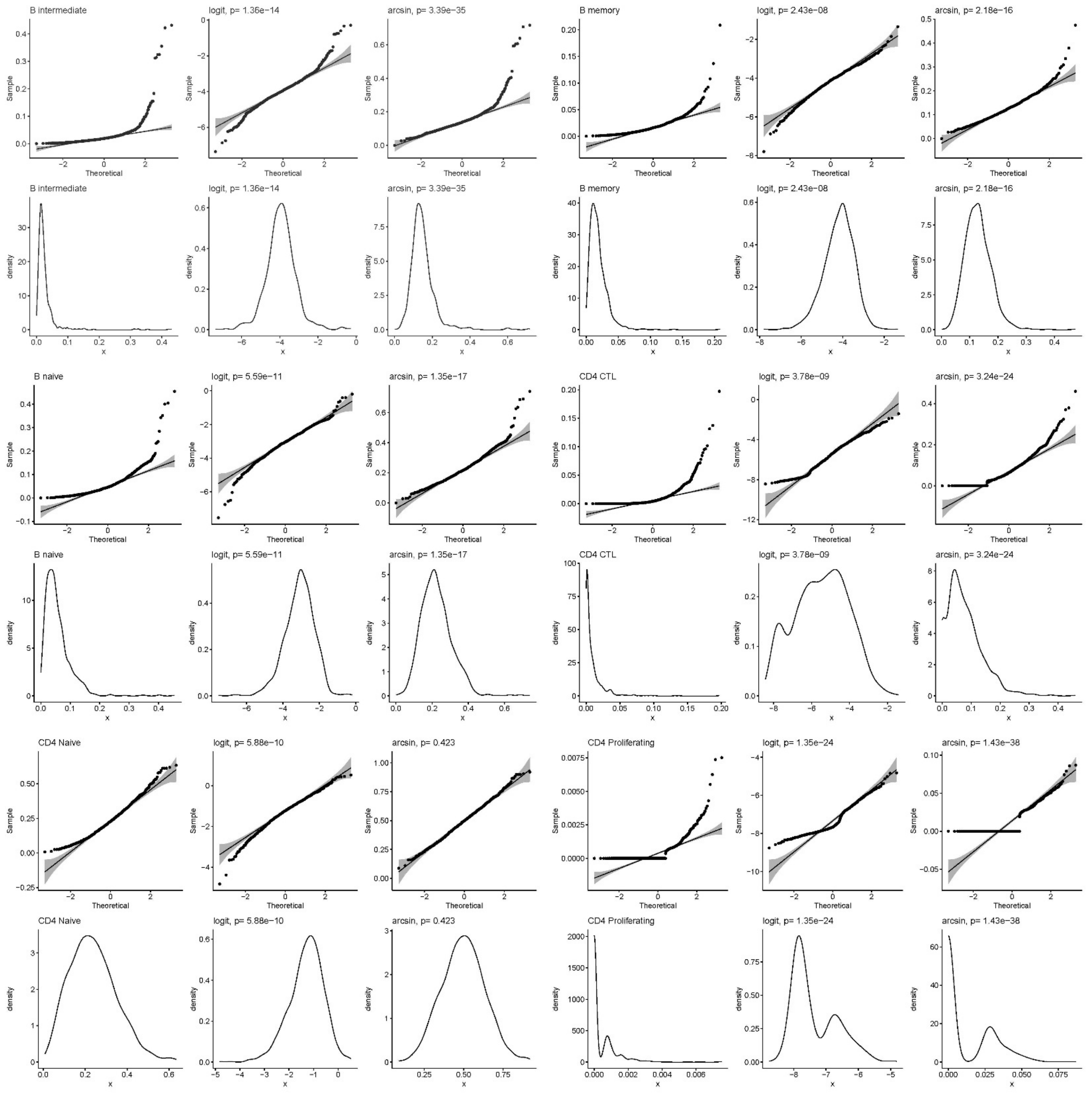

Figure S2 Tests of normality of proportions for each cell-type. Each QQ-plot shows the original proportions, the logit transformed and then the arcsin transformed data. Beneath each QQ-plot is the density distribution plot. Note, these are not split by sex. The remaining cell types are continued on the following pages.

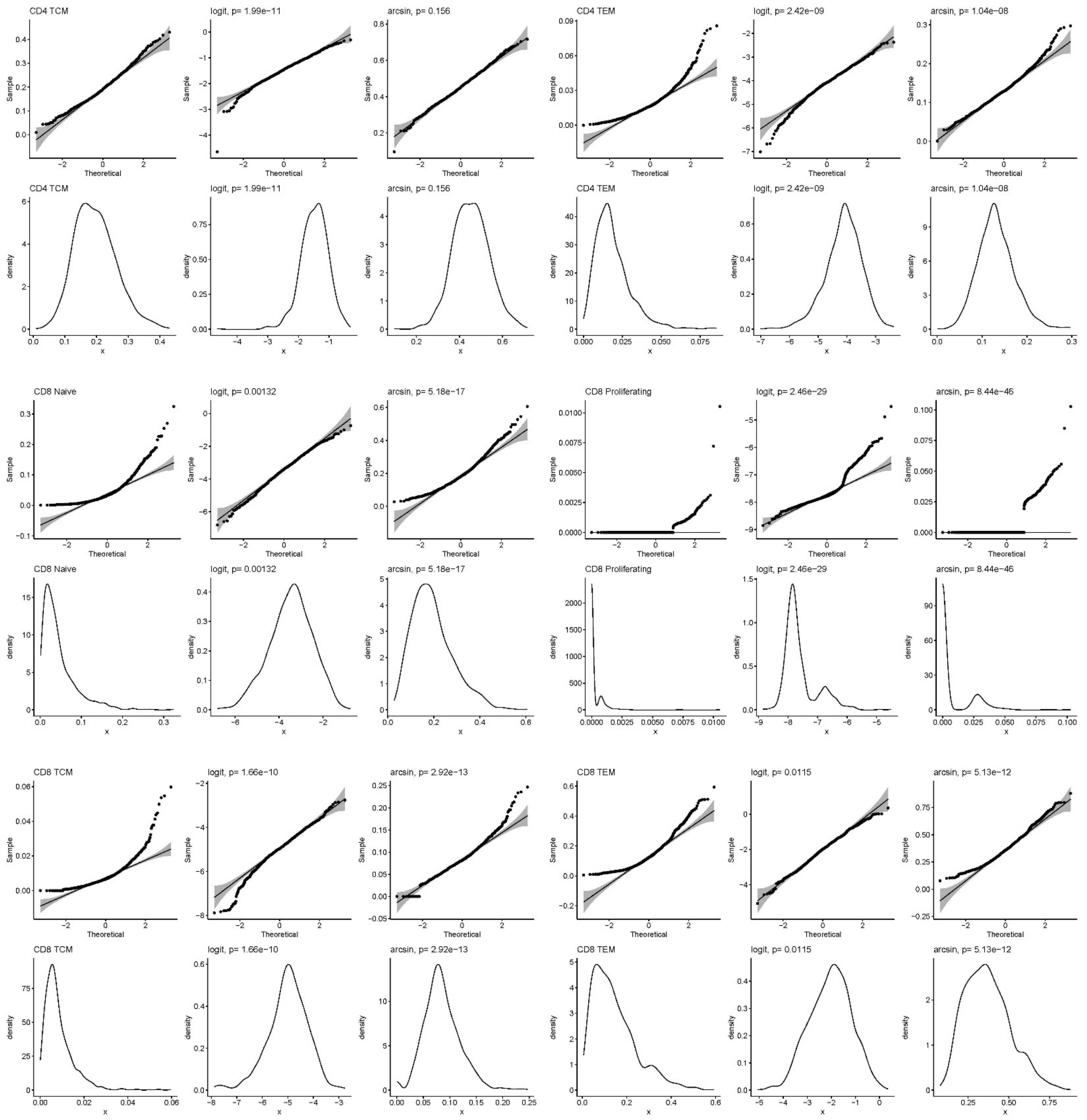

Figure S2 (continued) Tests of normality of proportions for each cell-type.

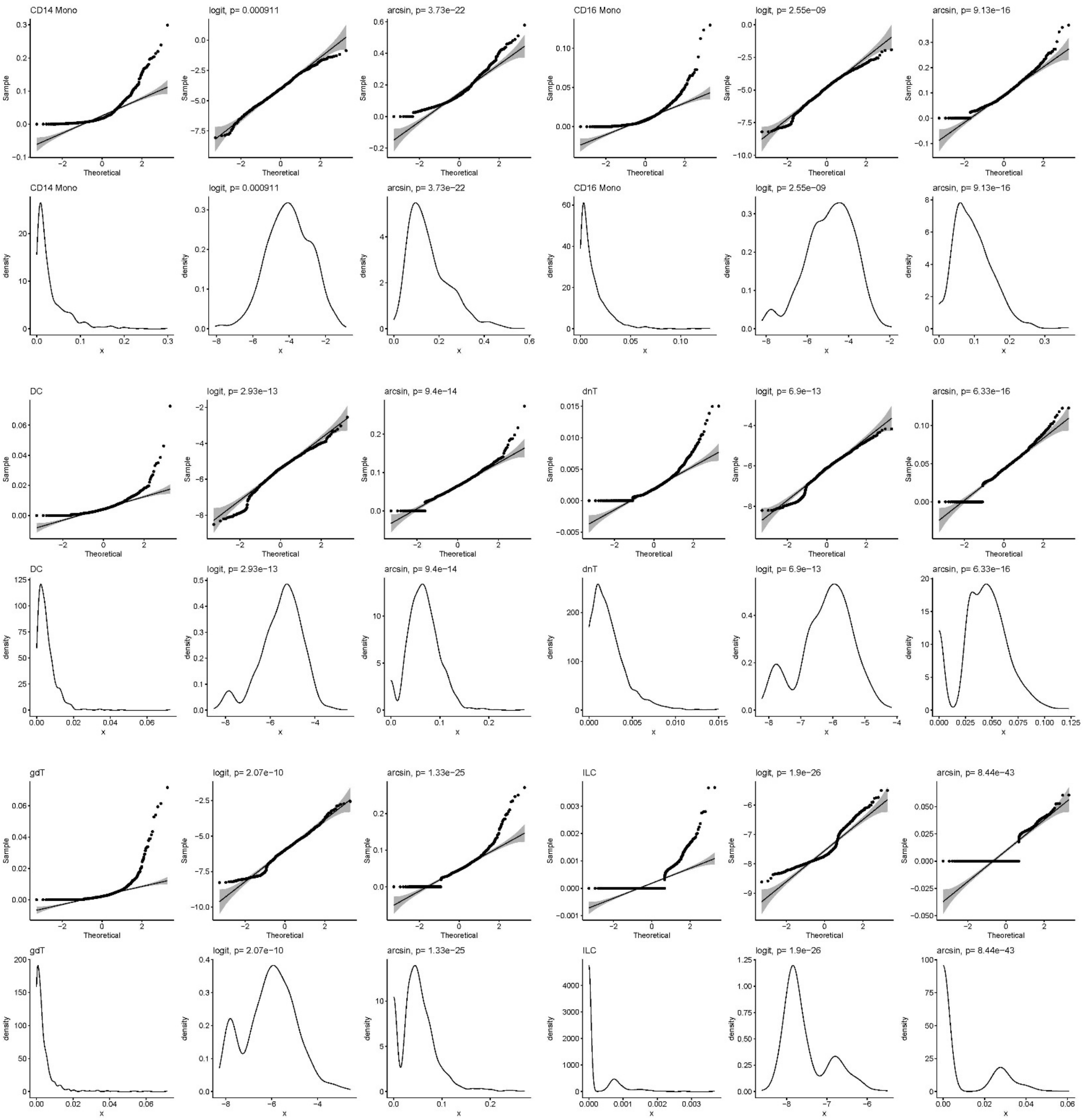

Figure S2 (continued) Tests of normality of proportions for each cell-type.

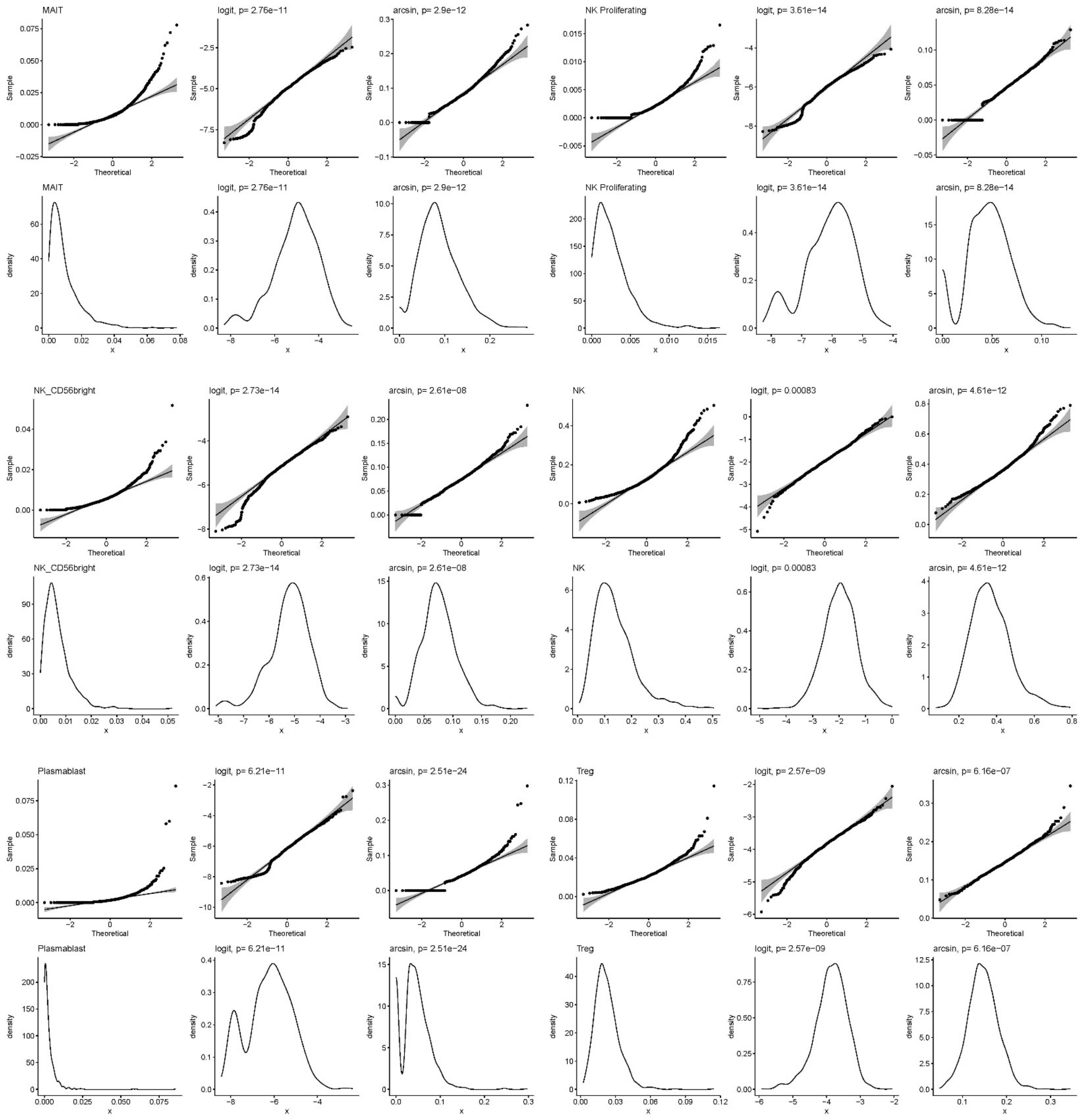

Figure S2 (continued) Tests of normality of proportions for each cell-type.

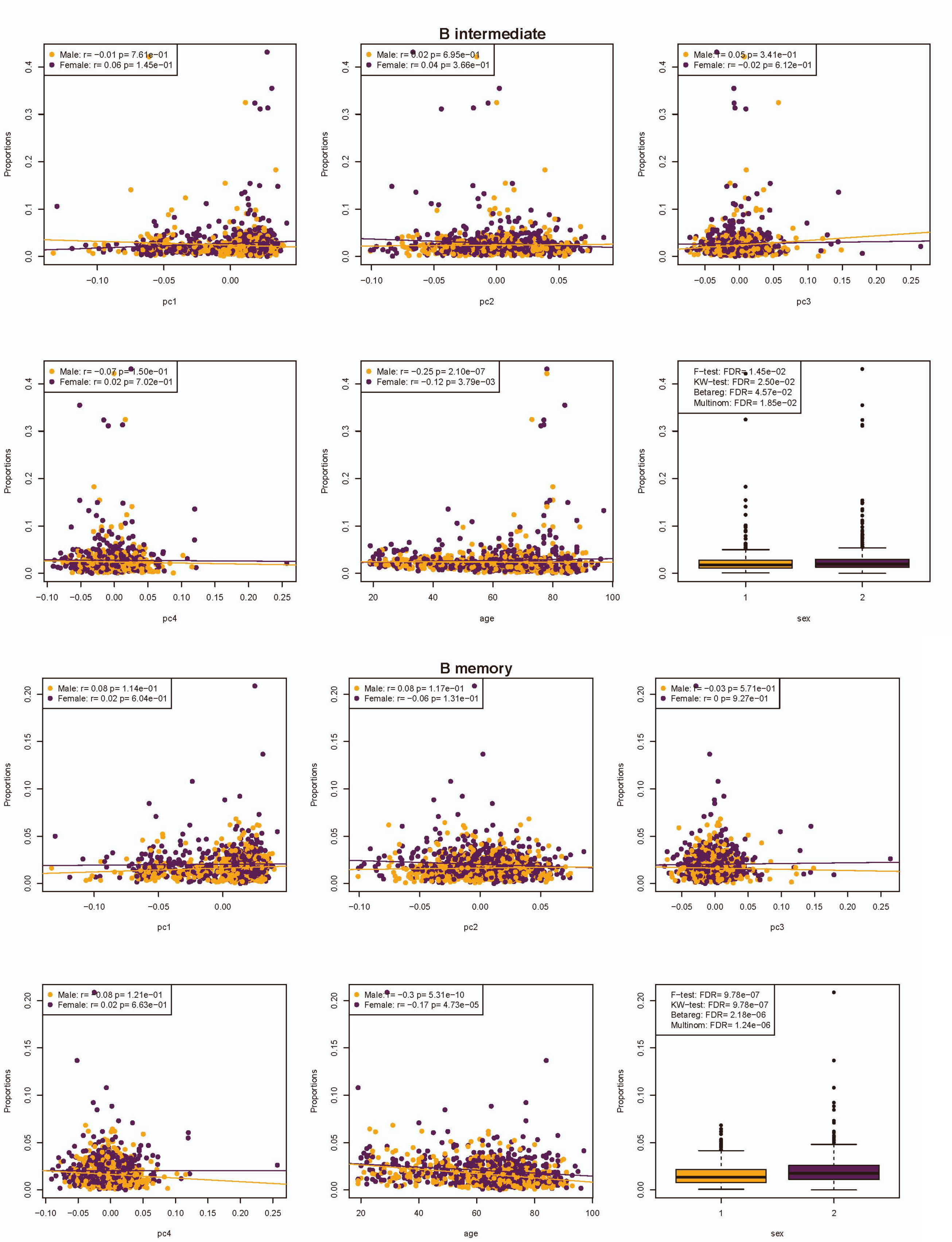

Figure S3 Proportions compared across covariates. Each scatter plot shows a variable (e.g., age, PC) versus the proportion, colored by sex. Purple are females, gold are males. A box plot shows the differences by sex. Each of the tests are labeled and marked in the plots.

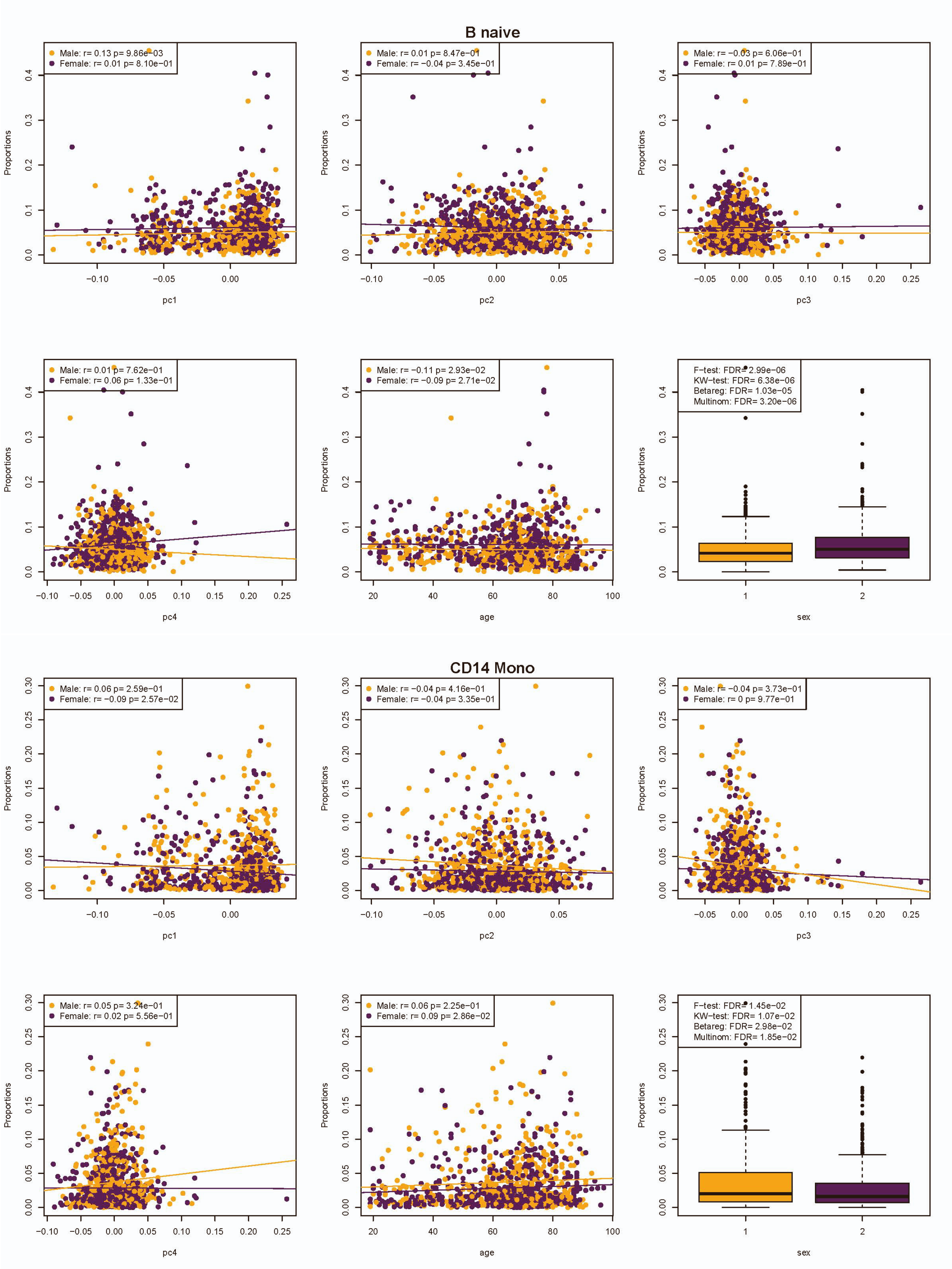

Figure S3 (continued) Proportions compared across covariates.

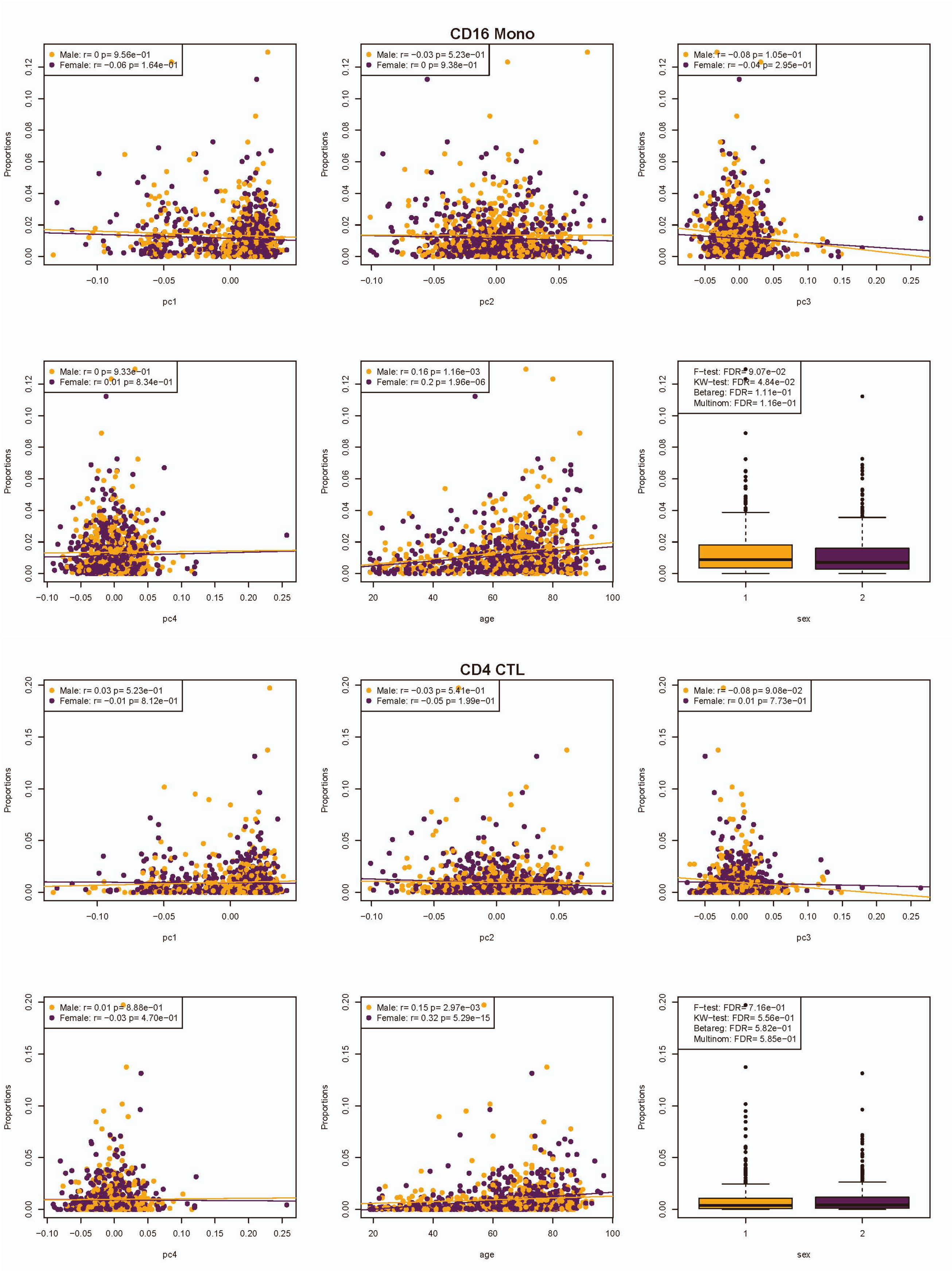

Figure S3 (continued) Proportions compared across covariates.

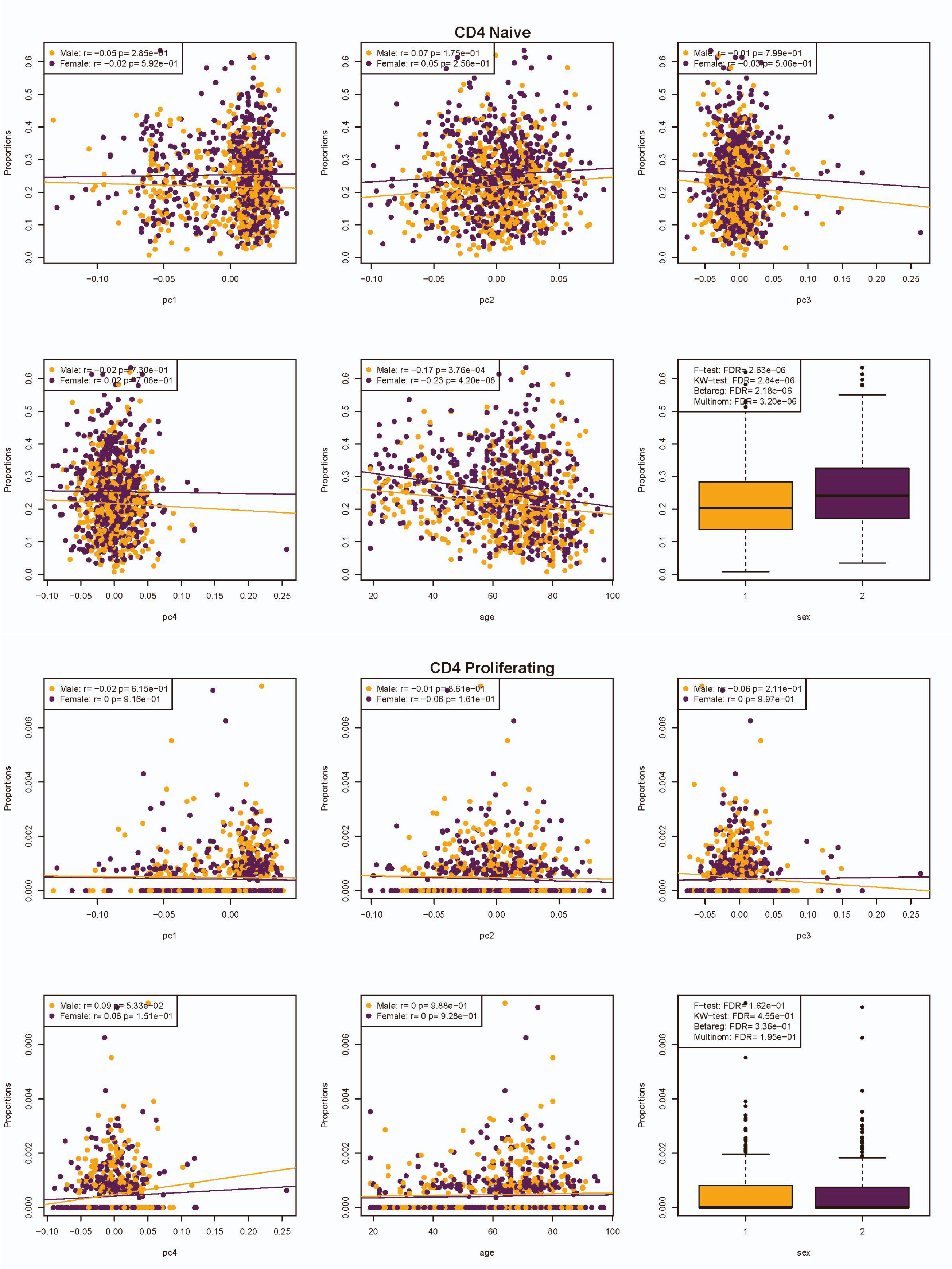

Figure S3 (continued) Proportions compared across covariates.

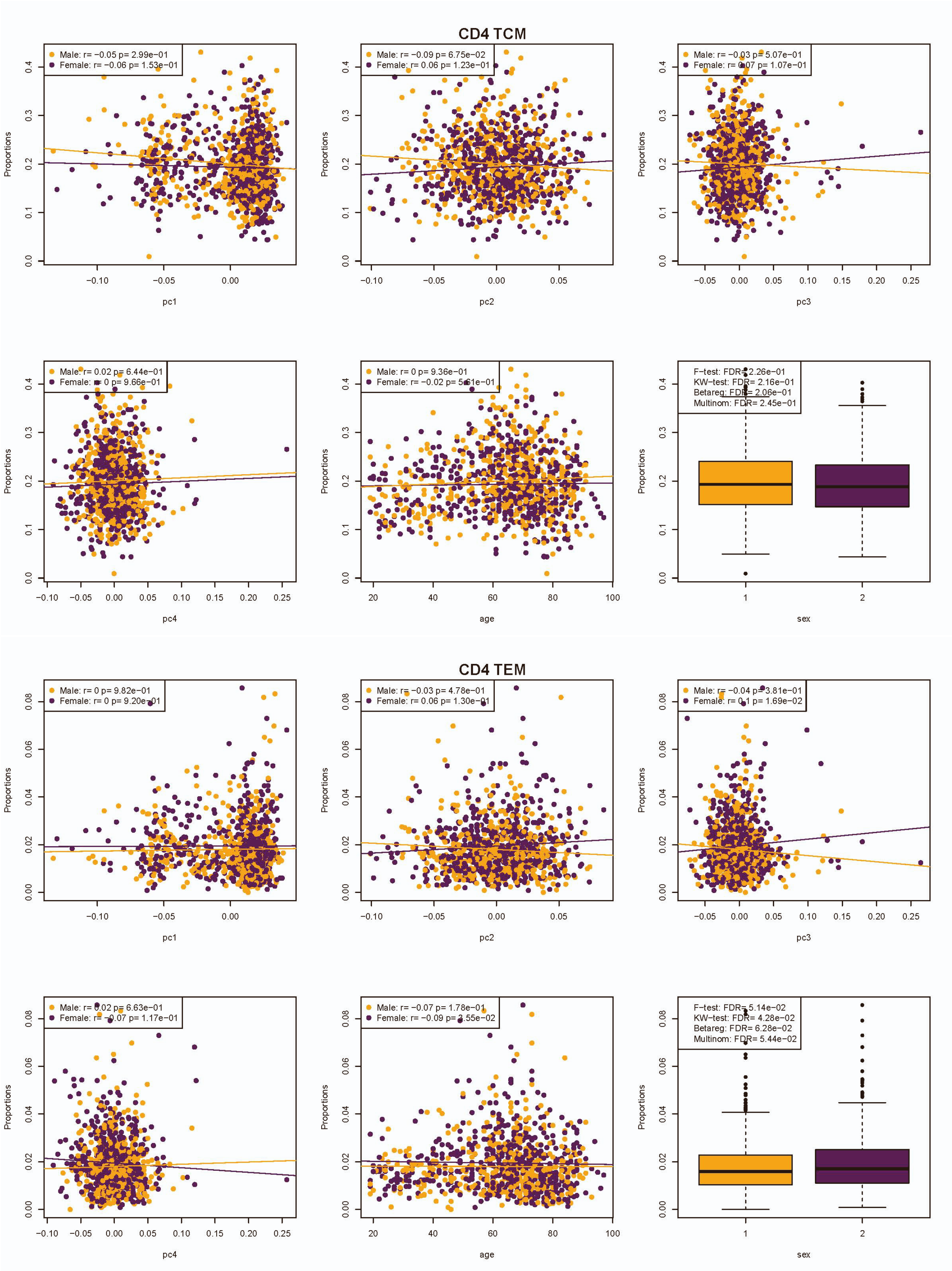

Figure S3 (continued) Proportions compared across covariates.

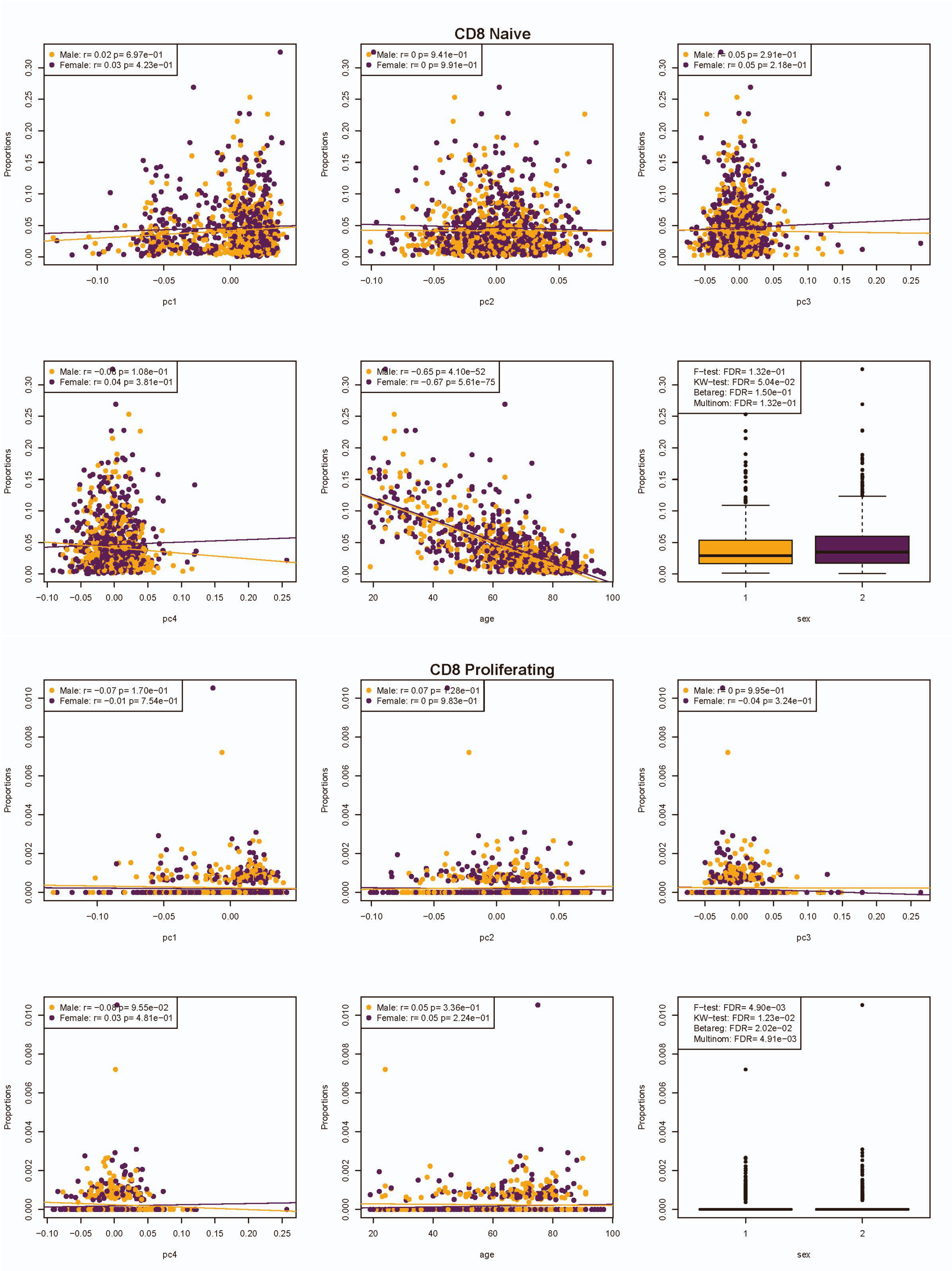

Figure S3 (continued) Proportions compared across covariates.

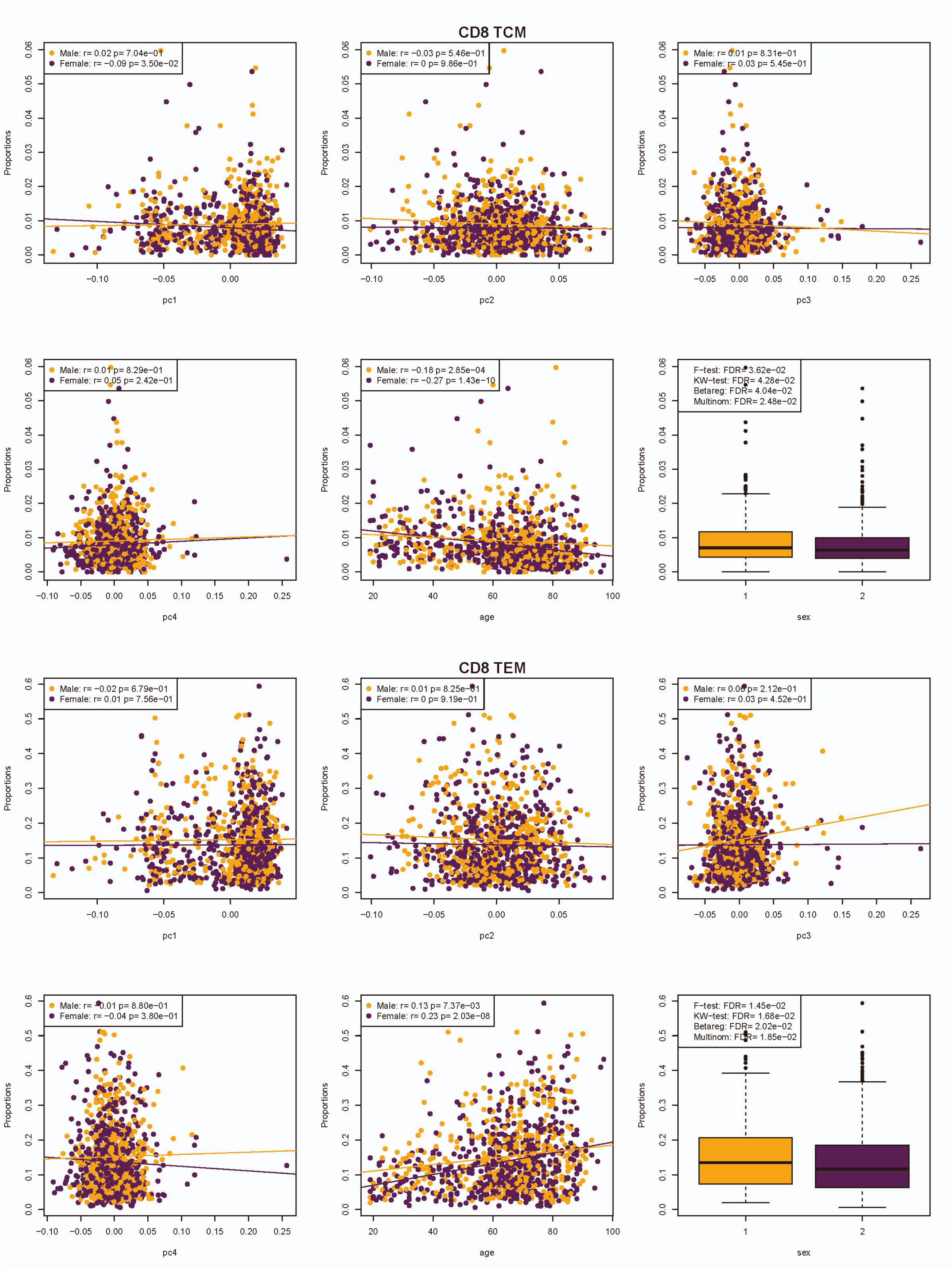

Figure S3 (continued) Proportions compared across covariates.

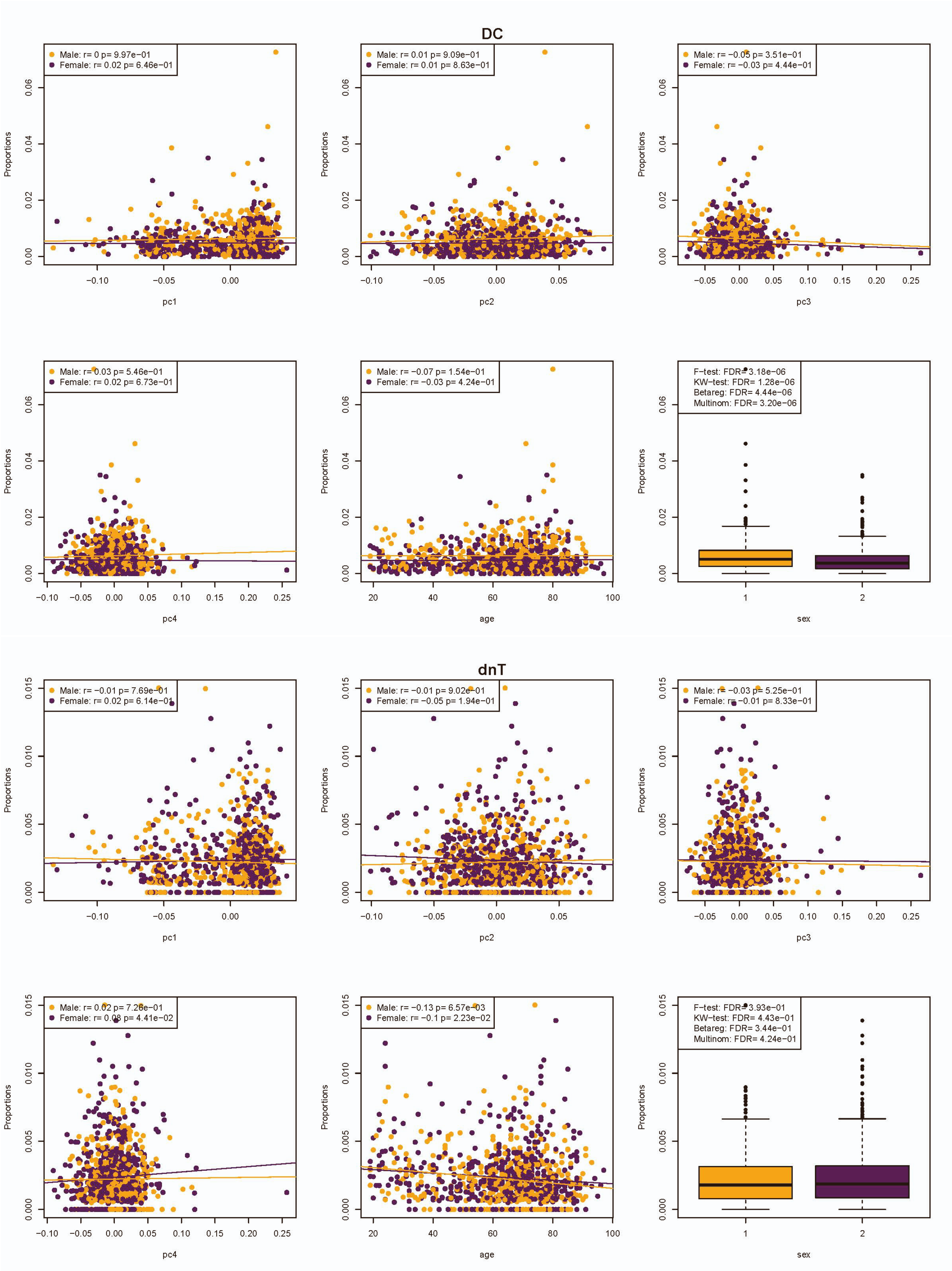

Figure S3 (continued) Proportions compared across covariates.

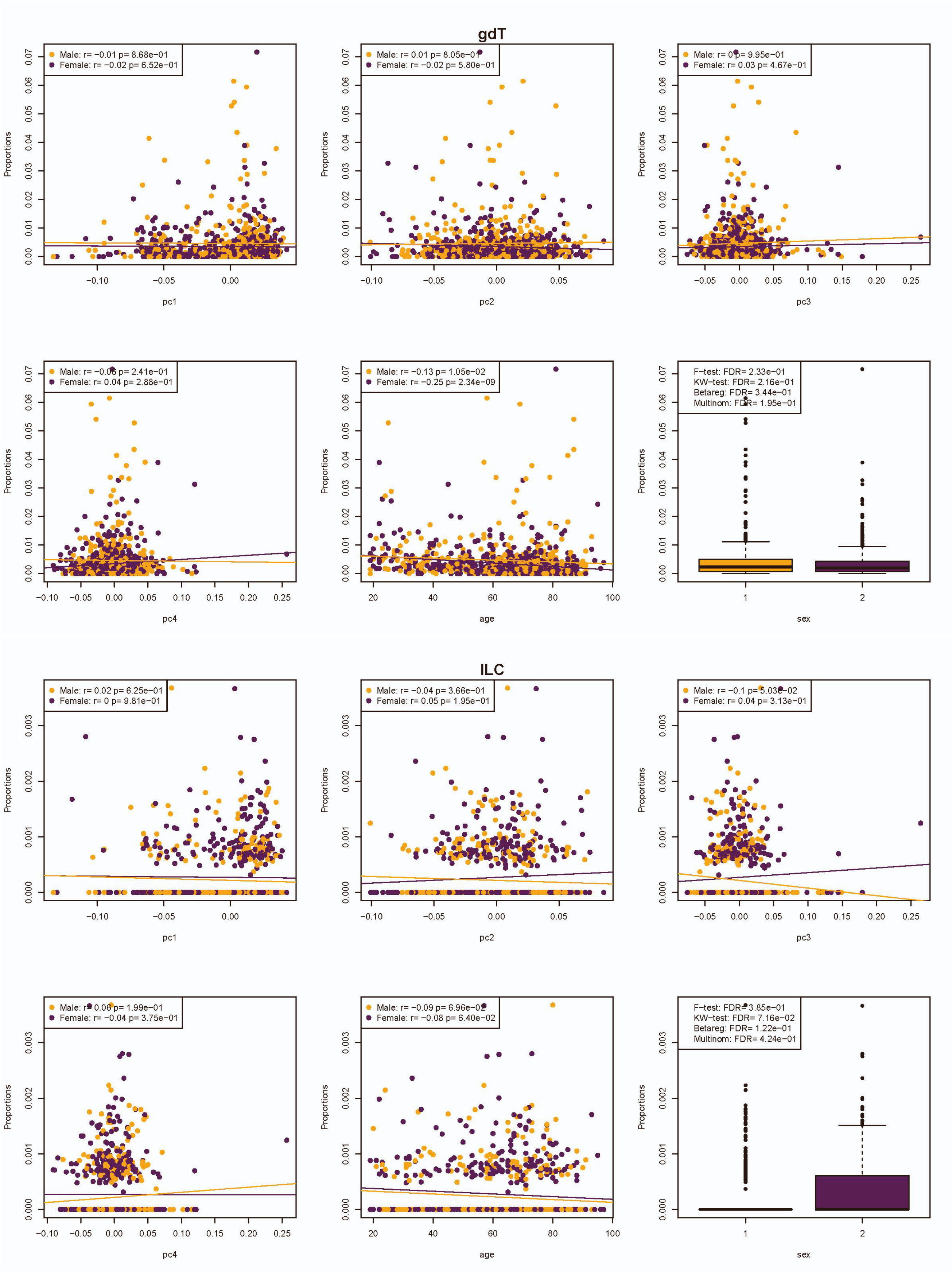

Figure S3 (continued) Proportions compared across covariates.

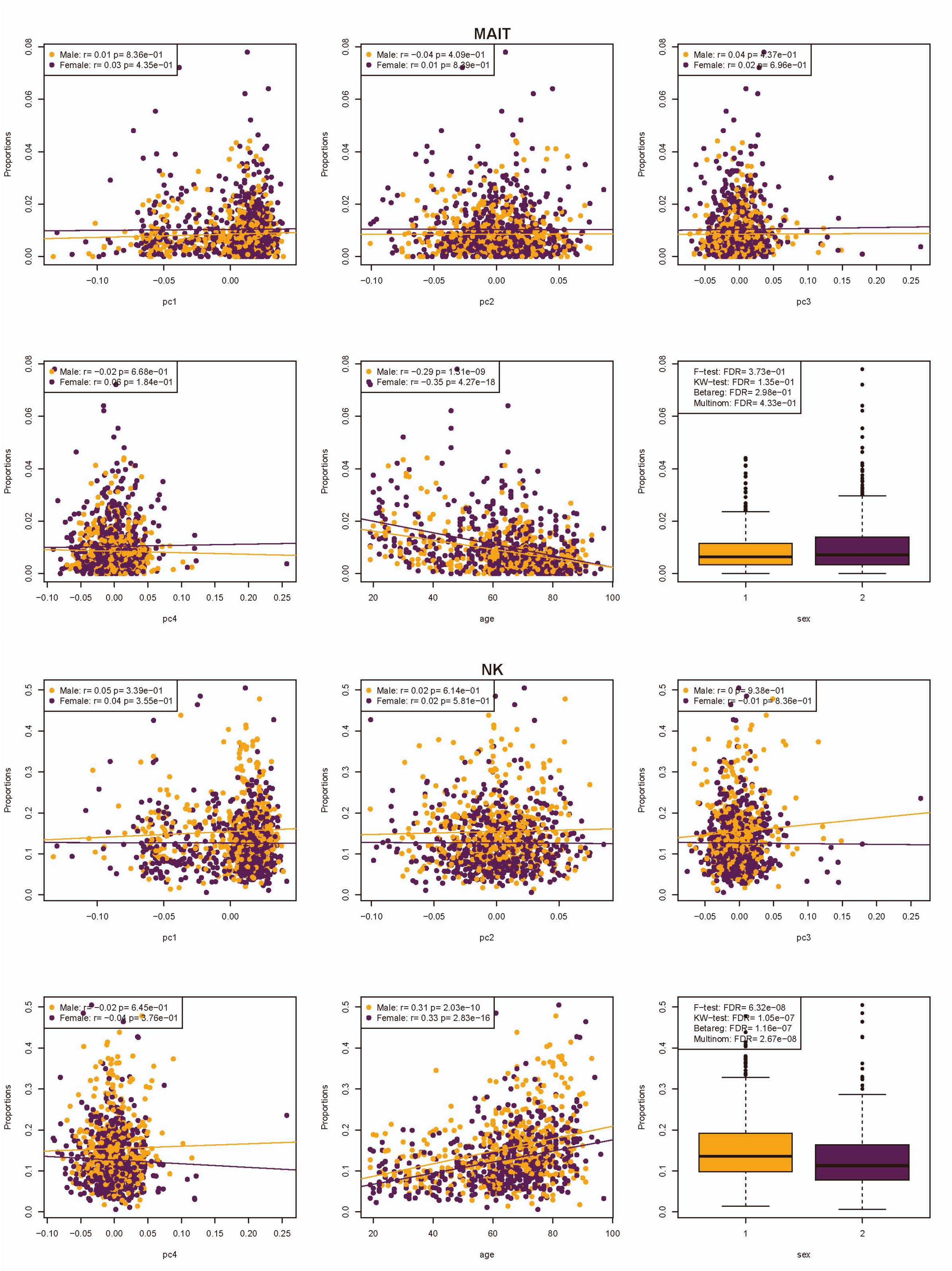

Figure S3 (continued) Proportions compared across covariates.

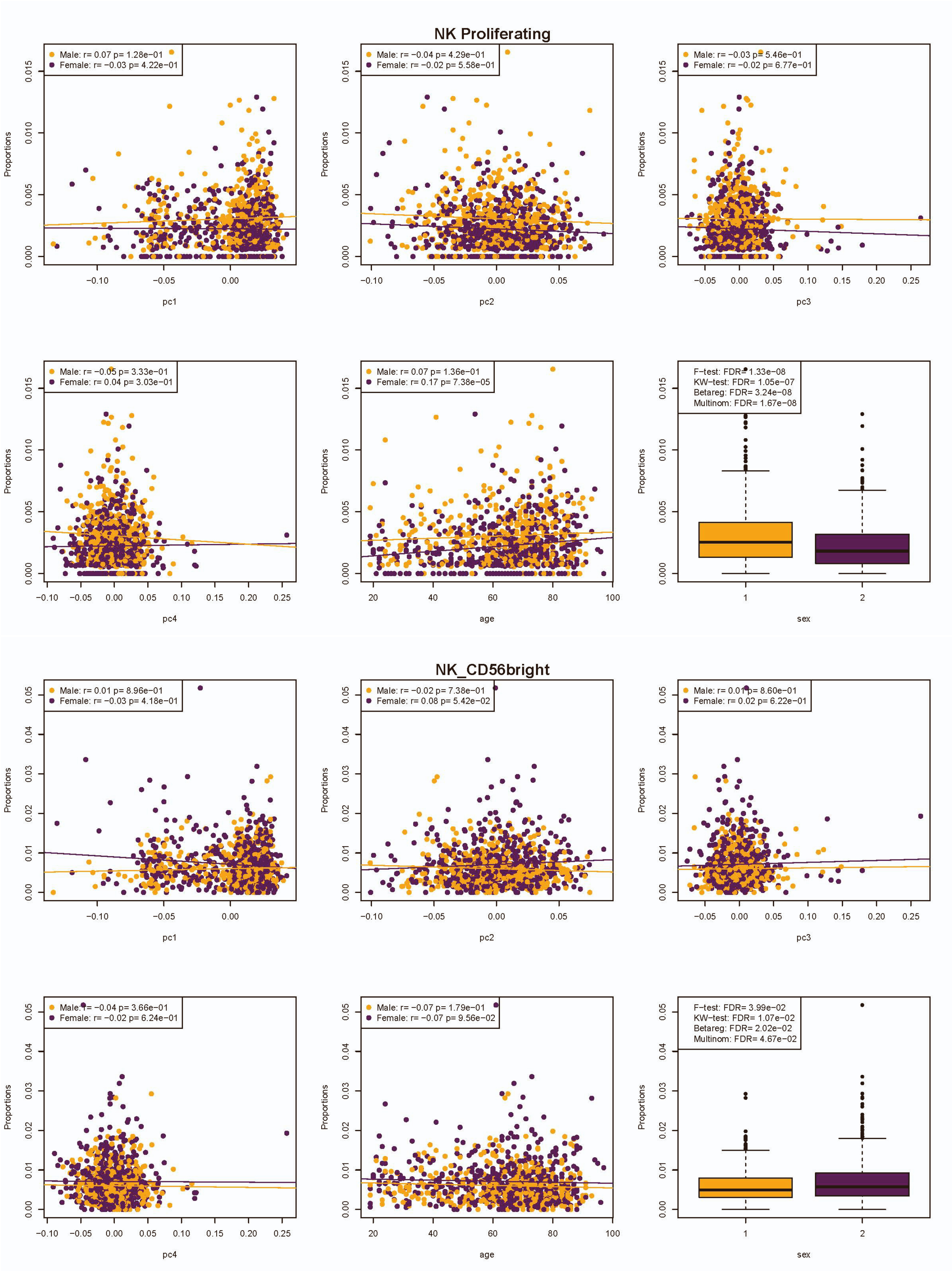

Figure S3 (continued) Proportions compared across covariates.

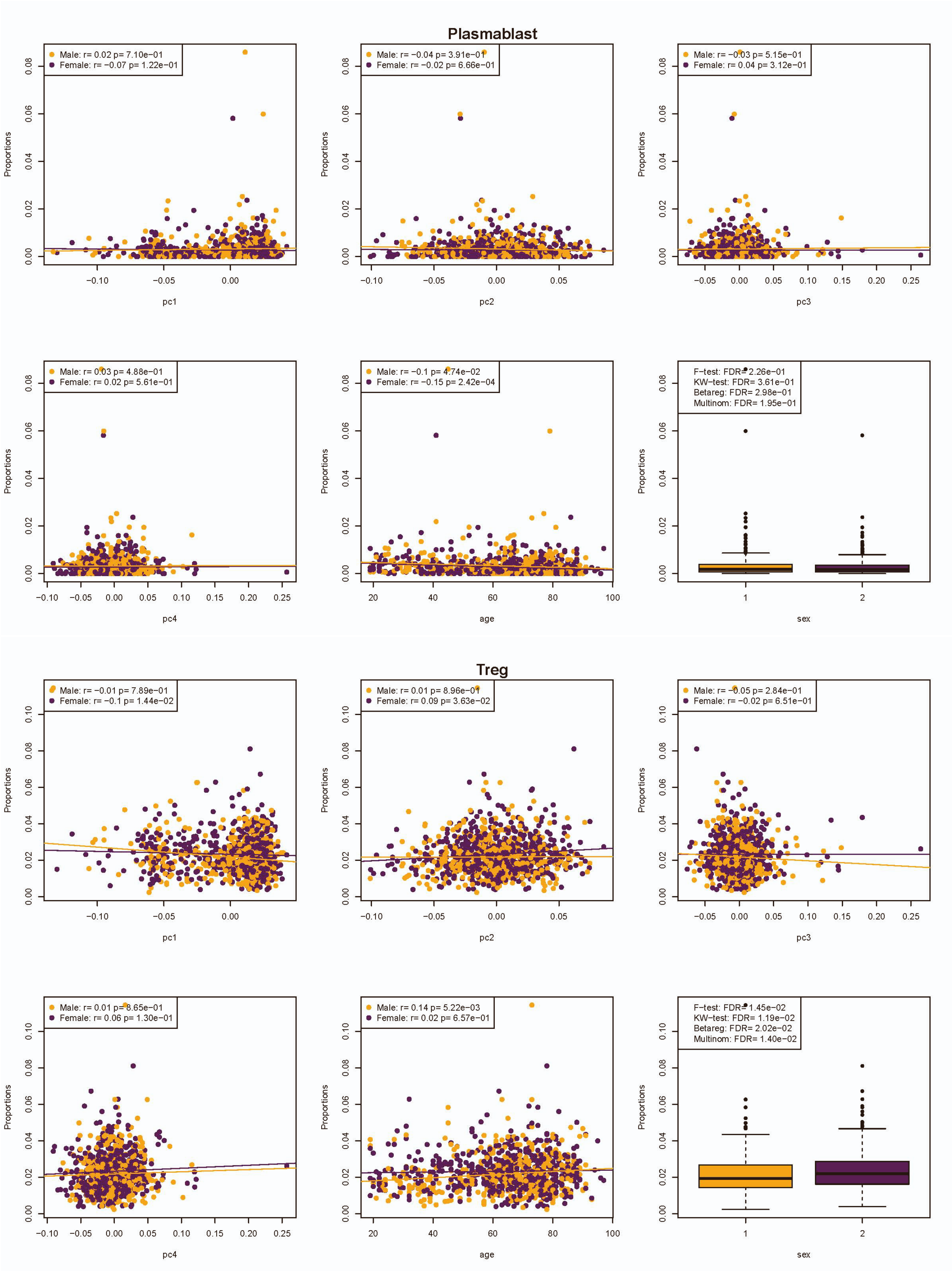

Figure S3 (continued) Proportions compared across covariates.

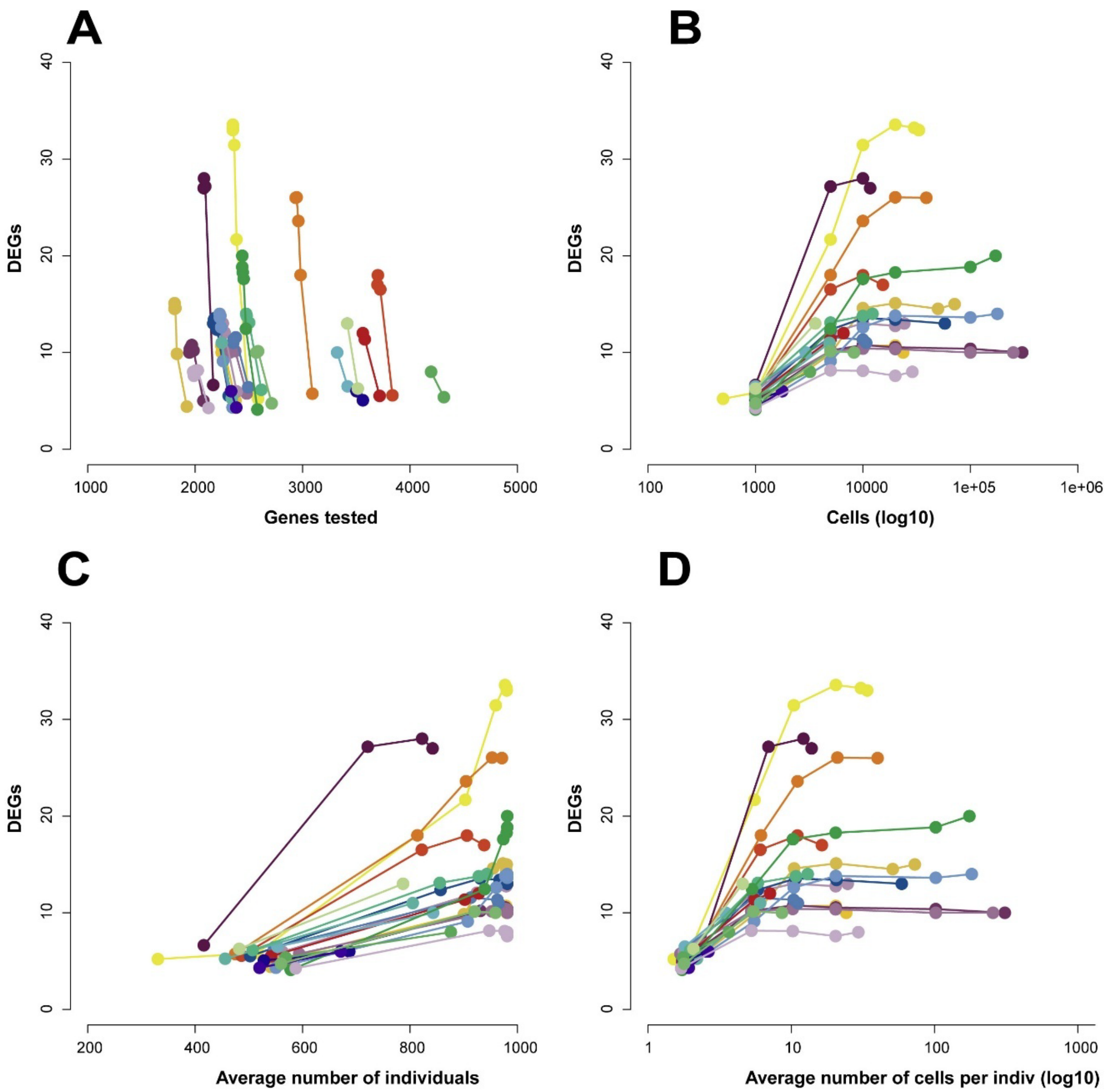

Figure S4 Downsampling differential expression analysis. (A) Plot of the number of genes tested and the resulting DEGs (total) per cell-type. (B) The number of cells downsampled versus the number of DEGs (total). (C) Number of individuals in the downsampled tests versus the DEGs. (D) The average number of cells per individual versus DEGs.

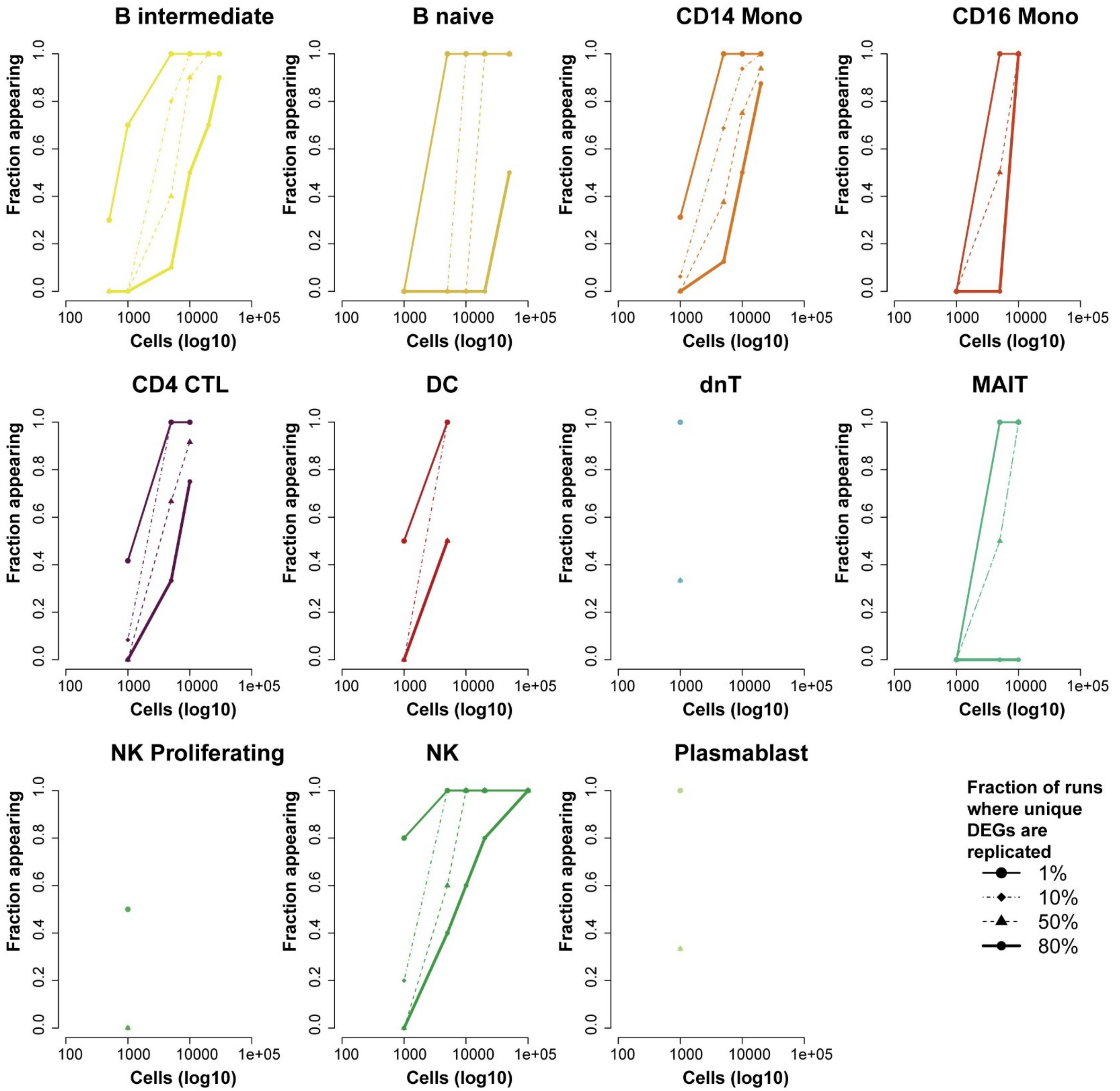

Figure S5 Replication of unique DEGs in downsampling analysis. Each plot indicates the frequency with which we consider a DEG replicated. In all these, we see obvious cell-type specific results that plateau suggesting that we are well powered for most of the analyses.

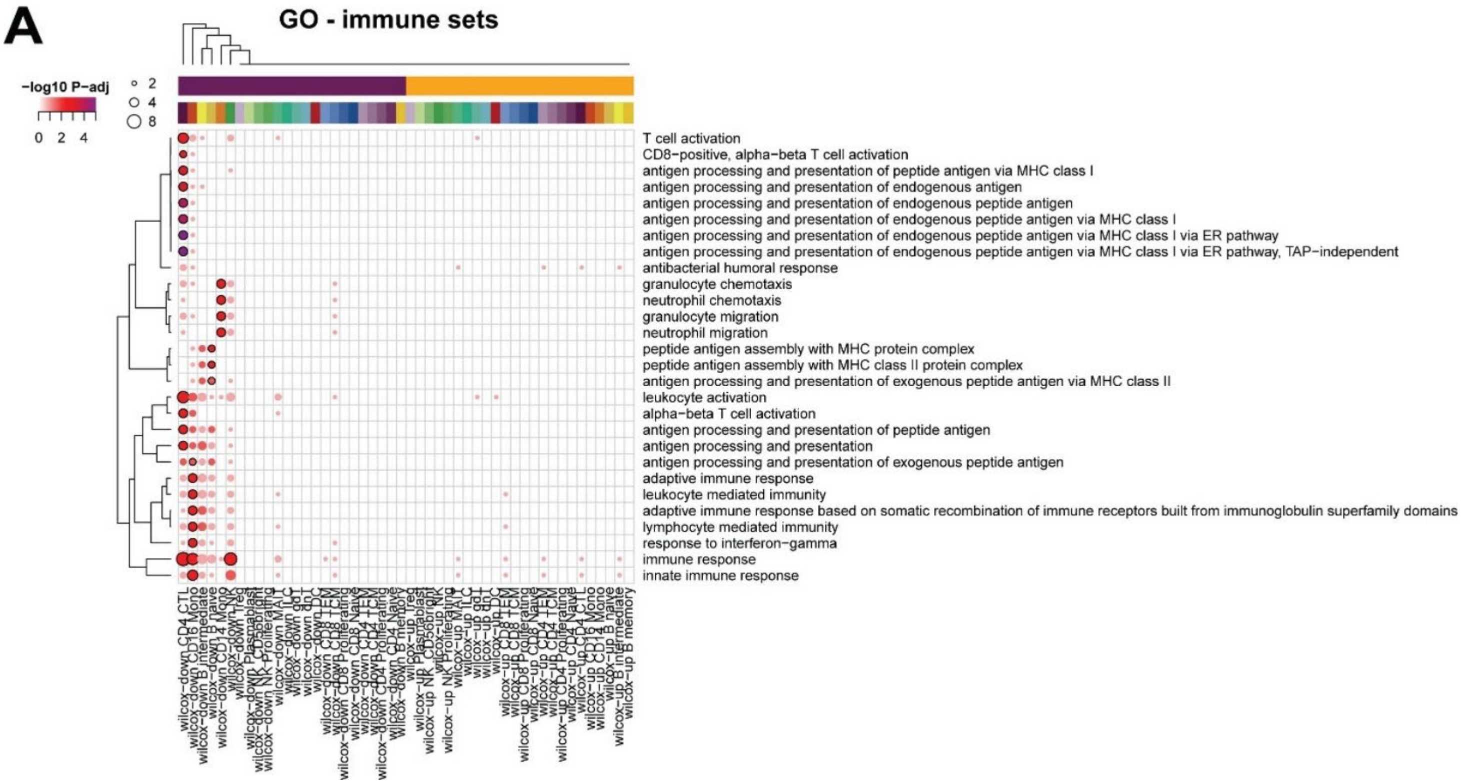

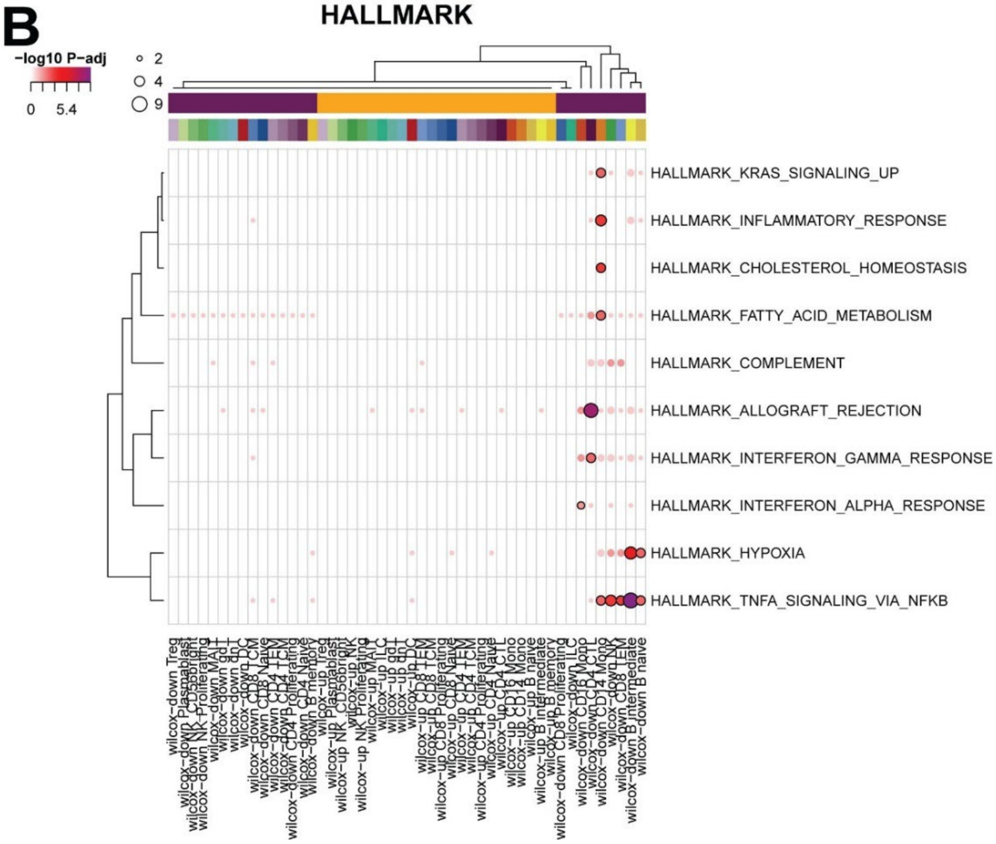

Figure S6 Sex-DEG enrichments. Gene set enrichment analysis for (A) GO immune sets, (B) HALLMARK, (C) KEGG/REACTOME/BIOCARTA, (D) HGNC, (E) Curated immune gene sets (MolSigDB C7) and (F) sex hormones and their targets.

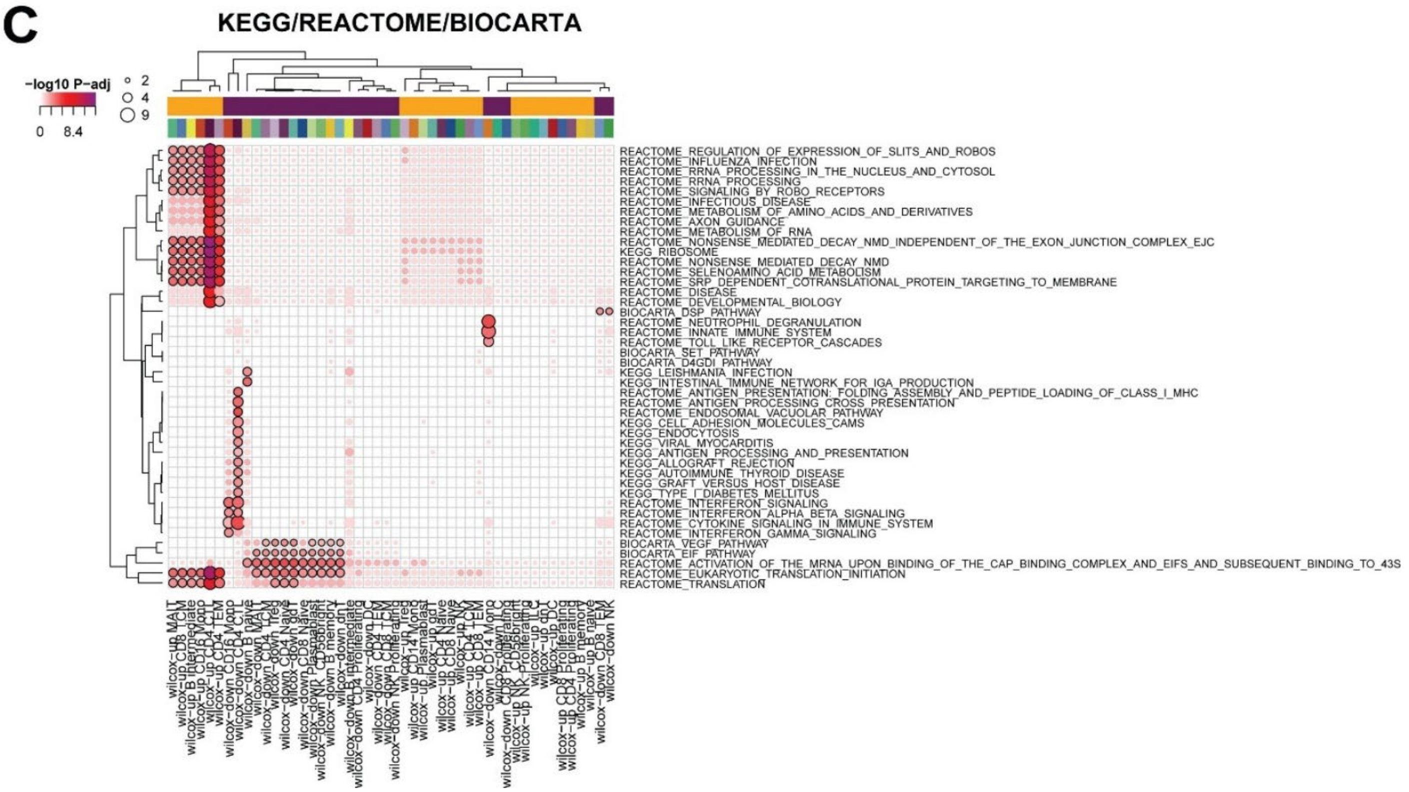

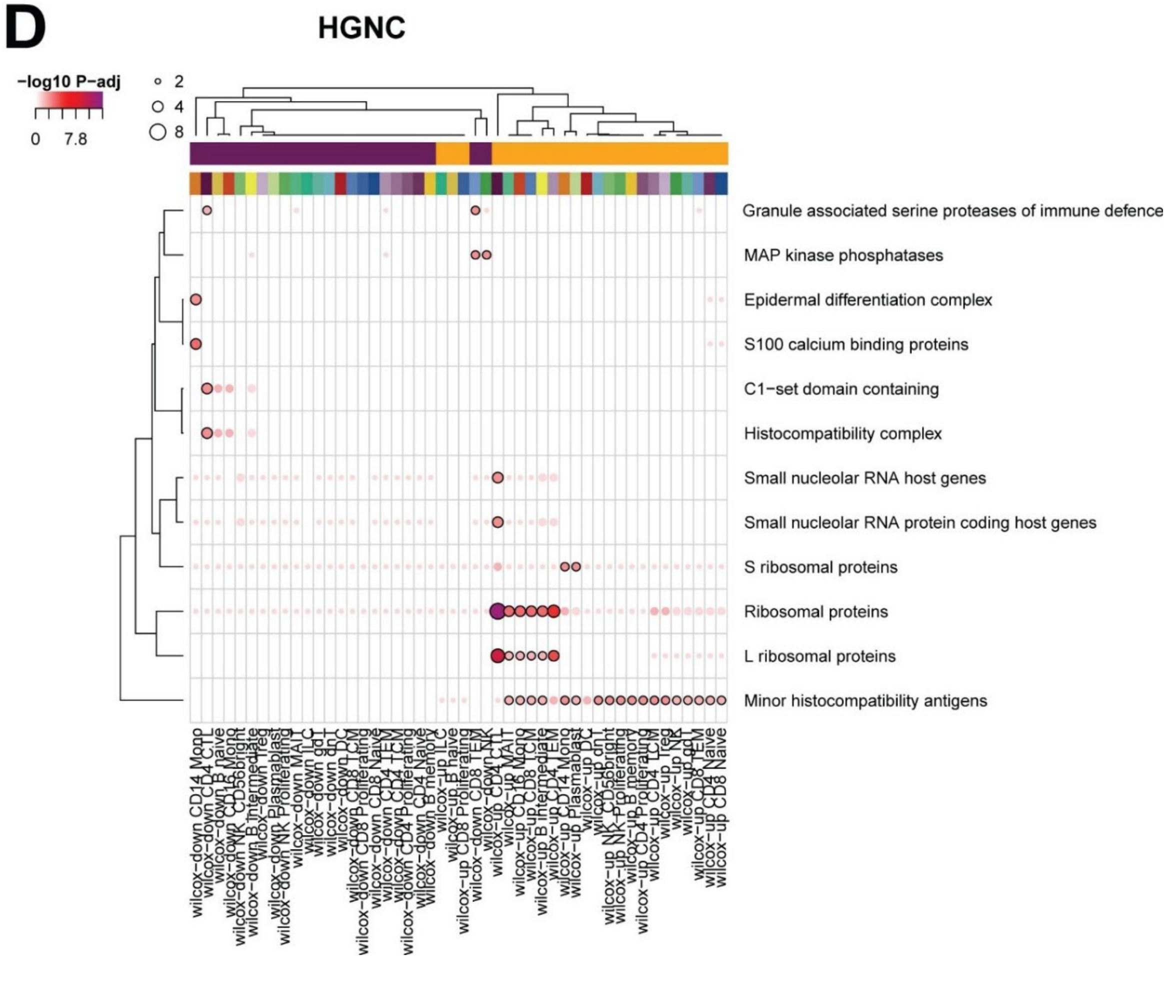

Figure S6 (continued) Sex-DEG enrichments.

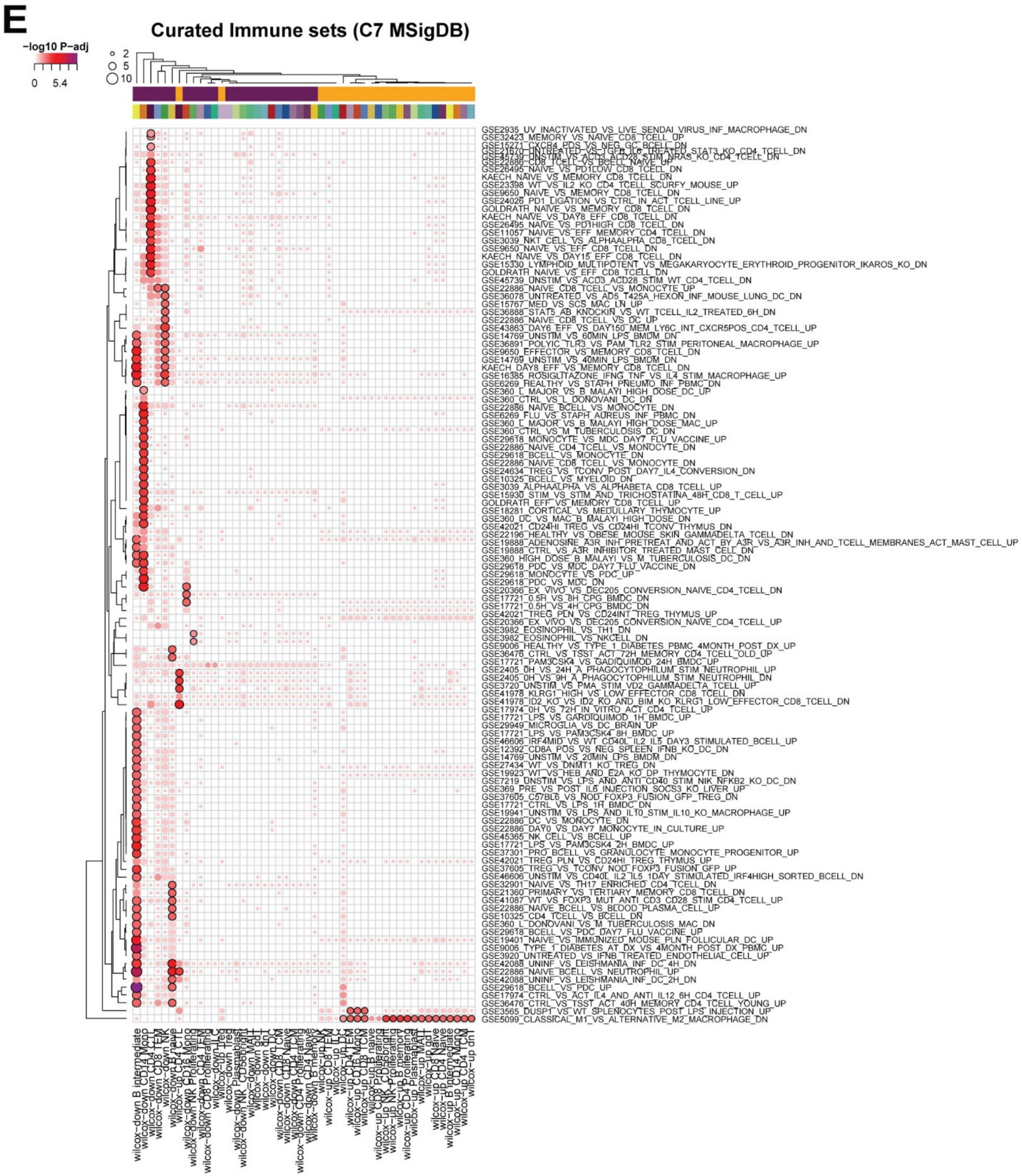

Figure S6 (continued) Sex-DEG enrichments.

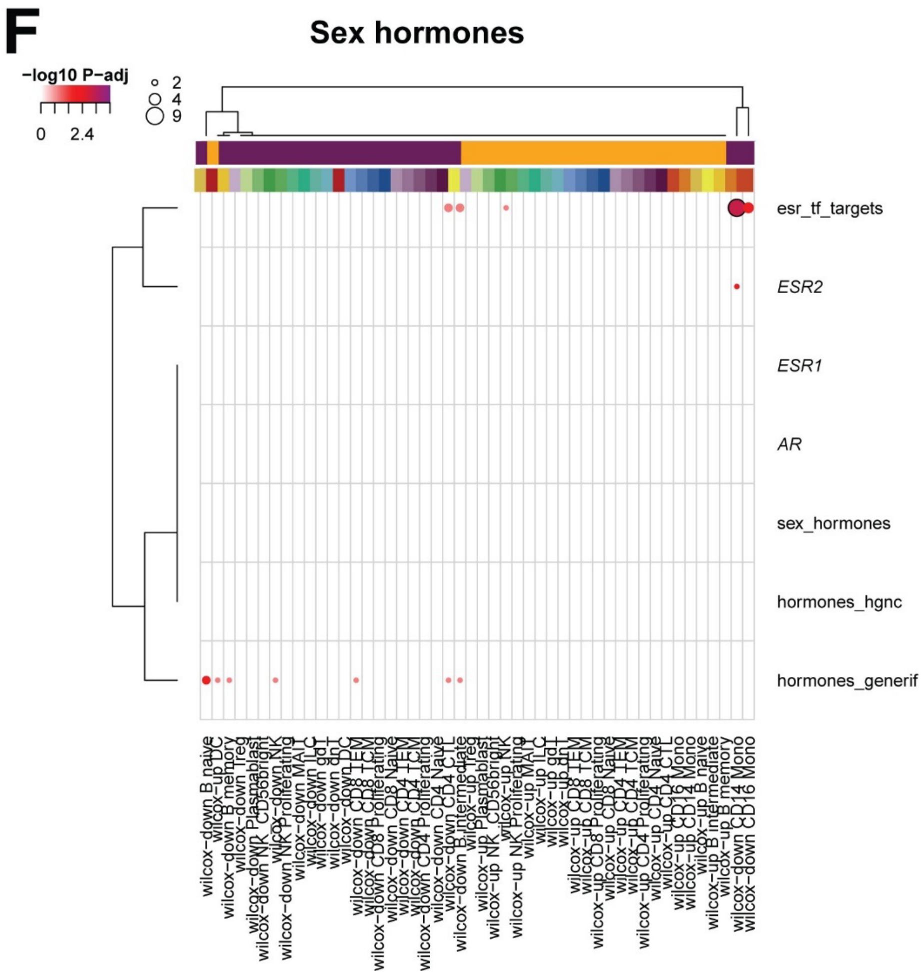

Figure S6 (continued) Sex-DEG enrichments.

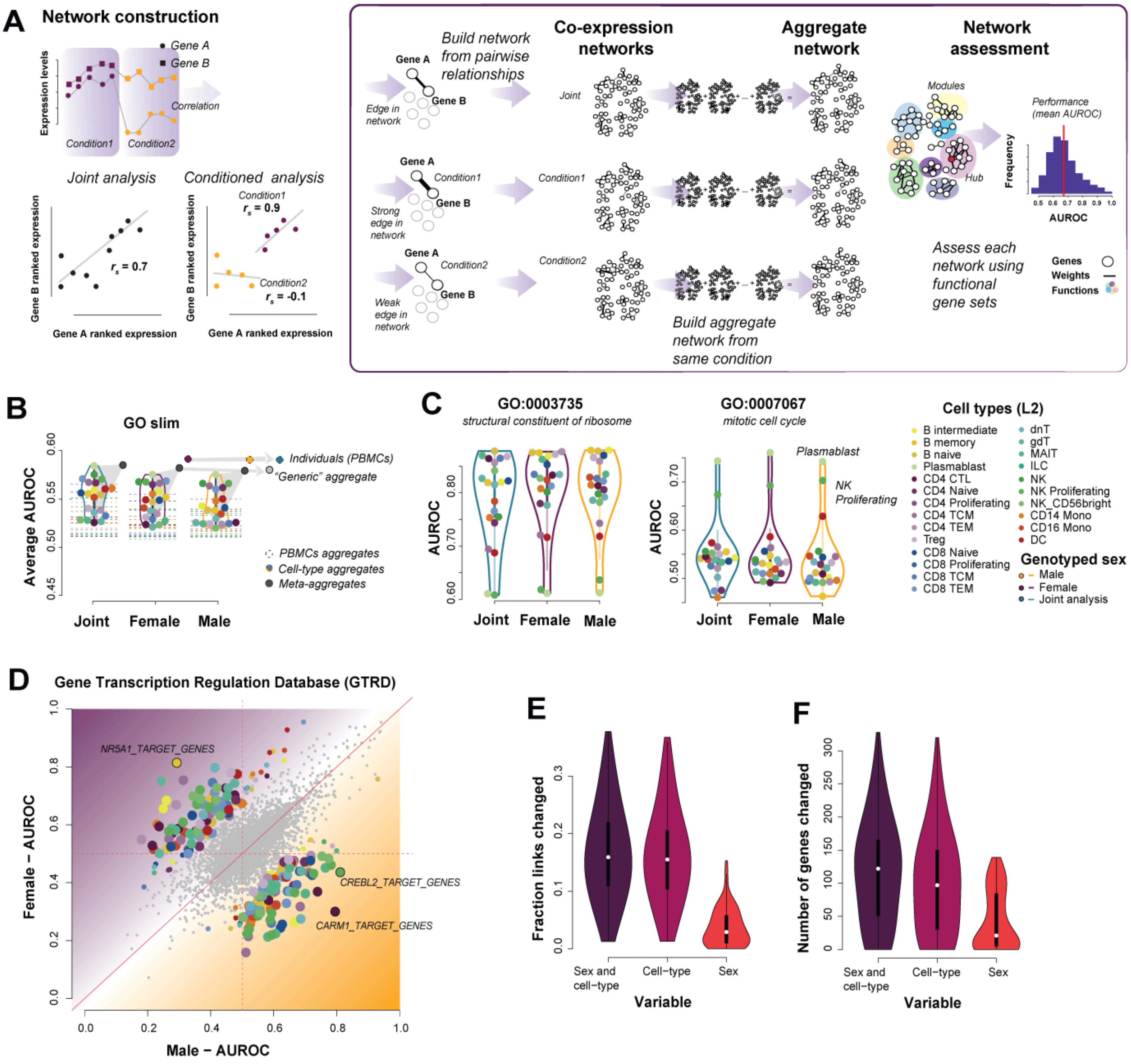

Figure S7 Co-expression by cell-type and sex. (A) Building co-expression networks and assessing performance. For each subset of cells, we calculate pairwise correlations between genes, which generates an edge in the network. This is repeated for different conditions (sex and cell-type), and these individual networks are aggregated into a final cell-type specific and sex-cell-type specific network. Enrichment of pathways and gene functions within modules formed in the network are tested through our neighbour-voting algorithm and recorded as an AUROC performance. (B) Network aggregation and performances of cell-type and sex-specific aggregates with GO slim. (C) Gene set specific performances showing differences for cell-types in ribosome and mitotic specific pathways (D) Comparing male AUROCs and female AUROCs for TF-target gene performances. Comparison of aggregate networks based on ranked expression weights (E) and node degree (F). On average, when comparing within the same cell-type but varying sex, we observe lower impacts on the edges, while comparing across cell-types within the same sex or across sex, the average number of differences are higher.

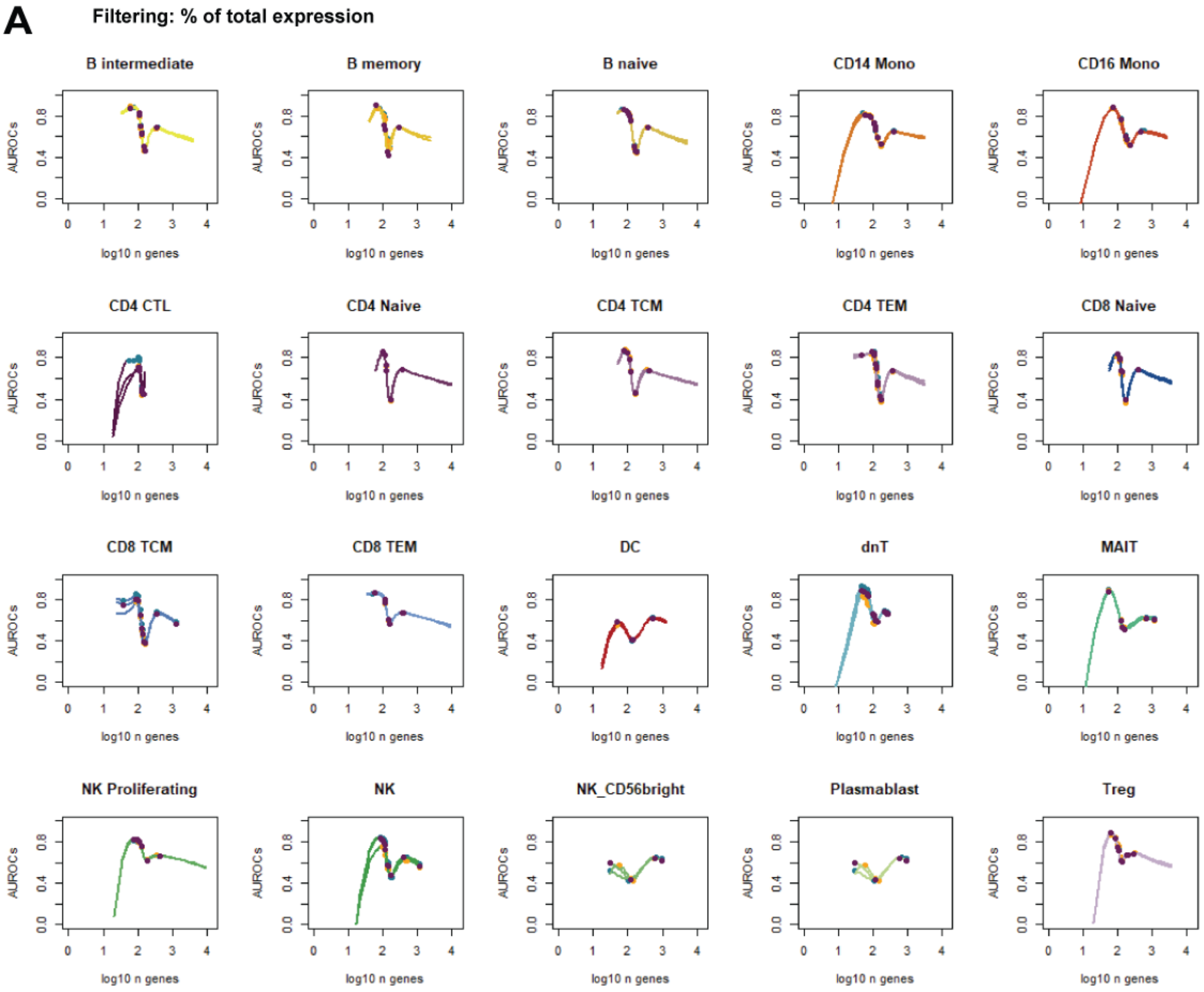

Figure S8 Aggregate co-expression downsampling comparisons. (A) For each cell-type aggregate, we filtered genes that were expressed in a fraction of the cells in that specific parameter set, (B) and those that overlapped across the sexes or joint analysis. The x-axis shows the size of the final set of genes (log10), and the y-axis the performance (AUROC) of that sub-network.

B Filtering: % of total expression, common genes across joint and sex

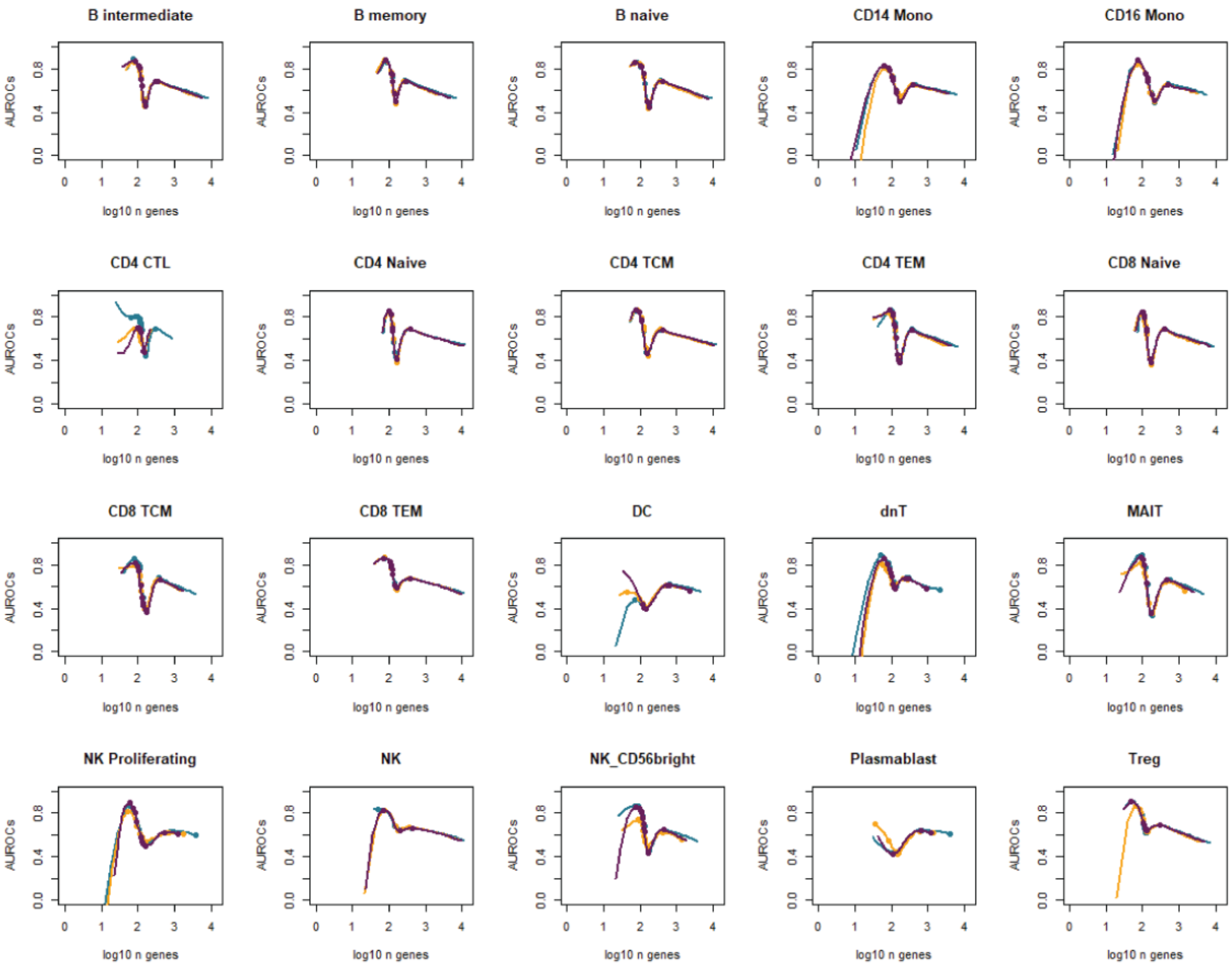

Figure S8 (continued) Aggregate co-expression downsampling comparisons.

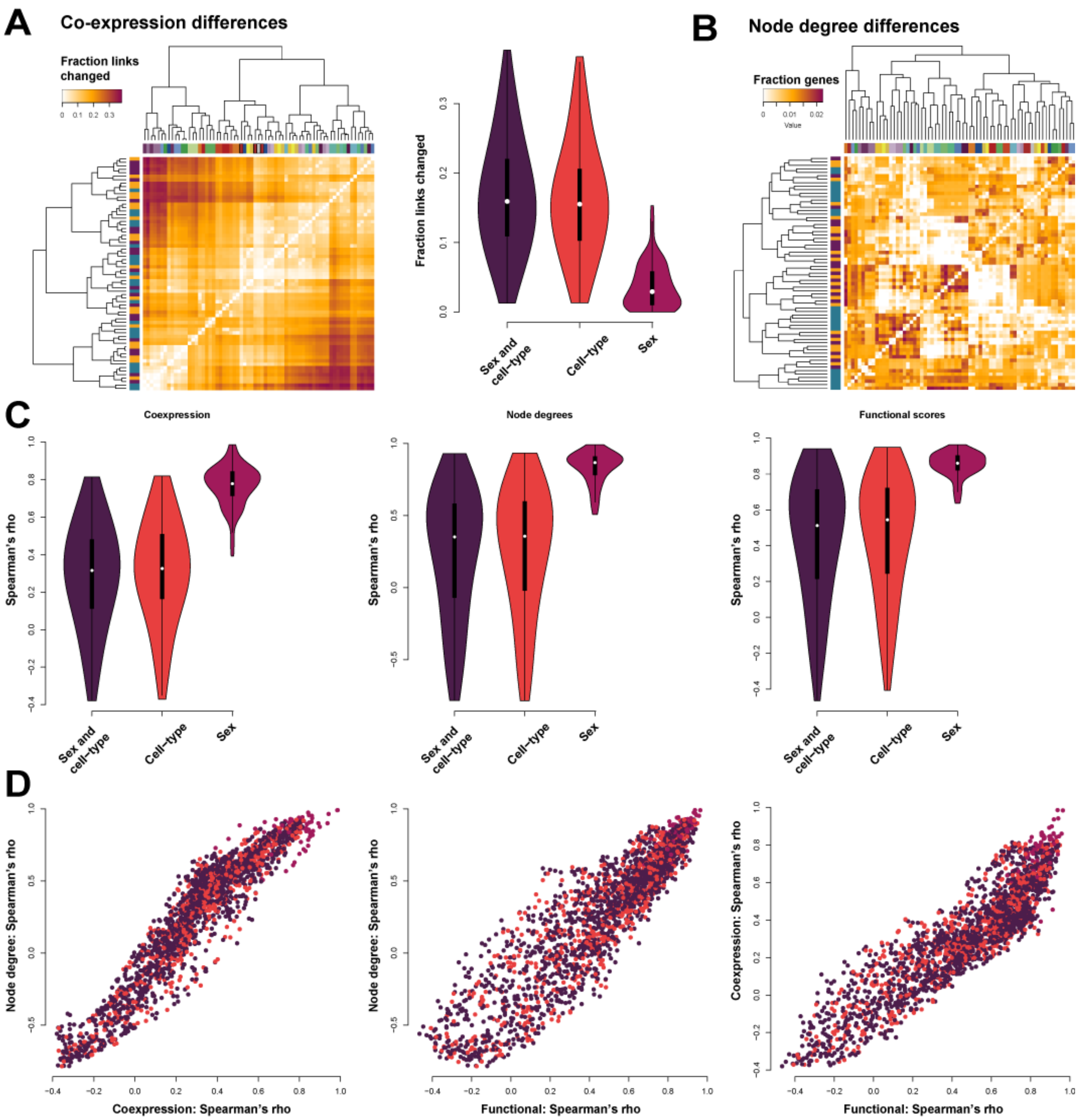

Figure S9 Differential co-expression comparisons. (A) Proportion of changed links between each aggregate network. (B) Proportion of genes with significantly different node degrees between each aggregate network. (C) Leftpanel: distribution of co-expression correlations between aggregates conditioned on sex and cell-type, cell-type only, or sex only. Middle panel: same as left panel but looking at node degrees and right-panel: network performance AUROCs for GO slim. (D) Comparing correlation values between co-expression, node degree and AUROCs.

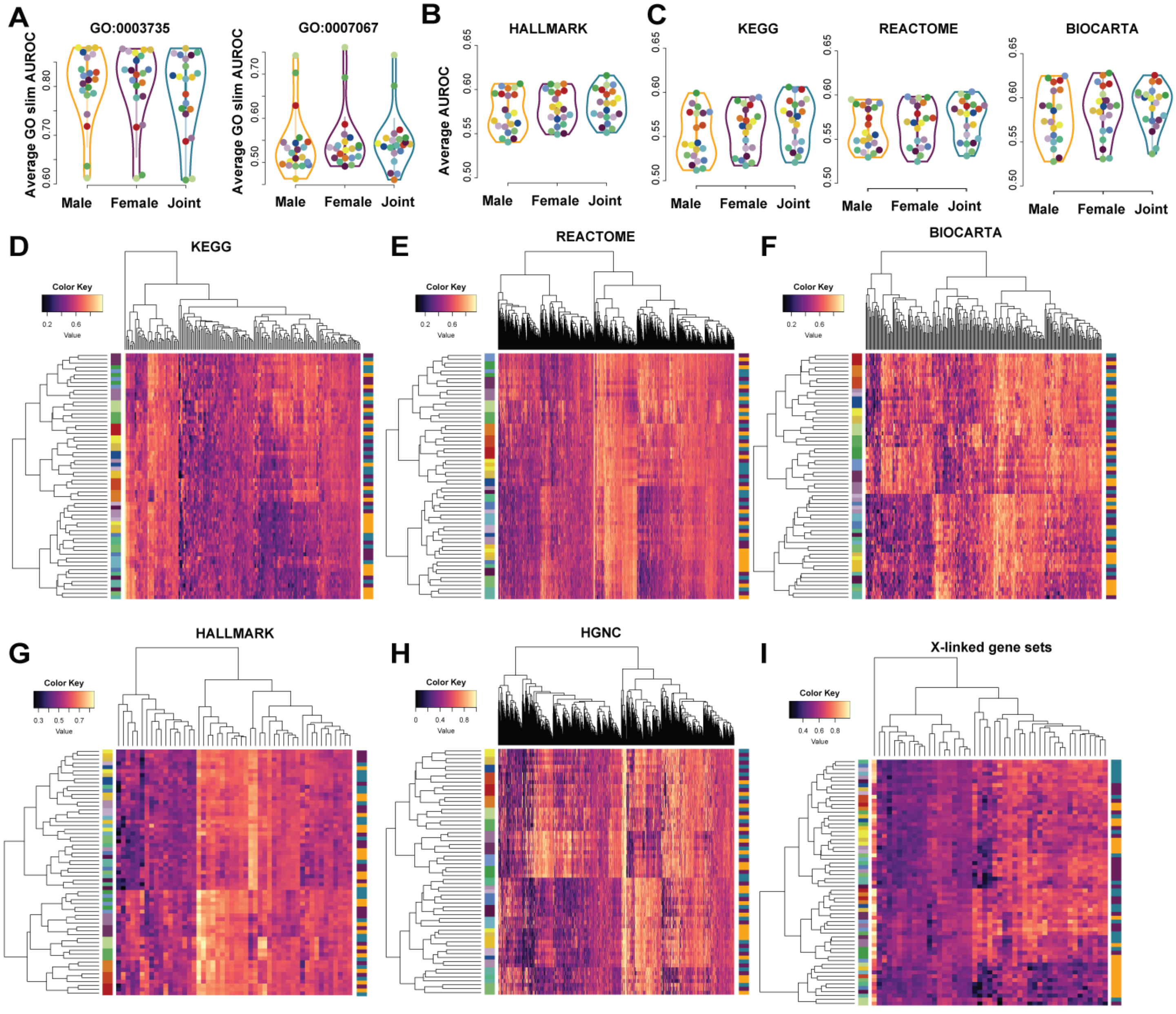

Figure S10 Functional enrichment results of aggregate networks using EGAD. (A) GO:0003735 structural constituent of ribosome example with high performances for most cell-types. (B) GO:0007067 mitotic cell cycle example with high performances in proliferating cell-types.(C) Average KEGG, REACTOME and BIOCARTA AUROCS. (D) Performance AUROCs for all KEGG, (E) REACTOME, (F) BIOCARTA, (G) HALLMARK, (H) HGNC and (I) curated X-linked gene sets and pathways.

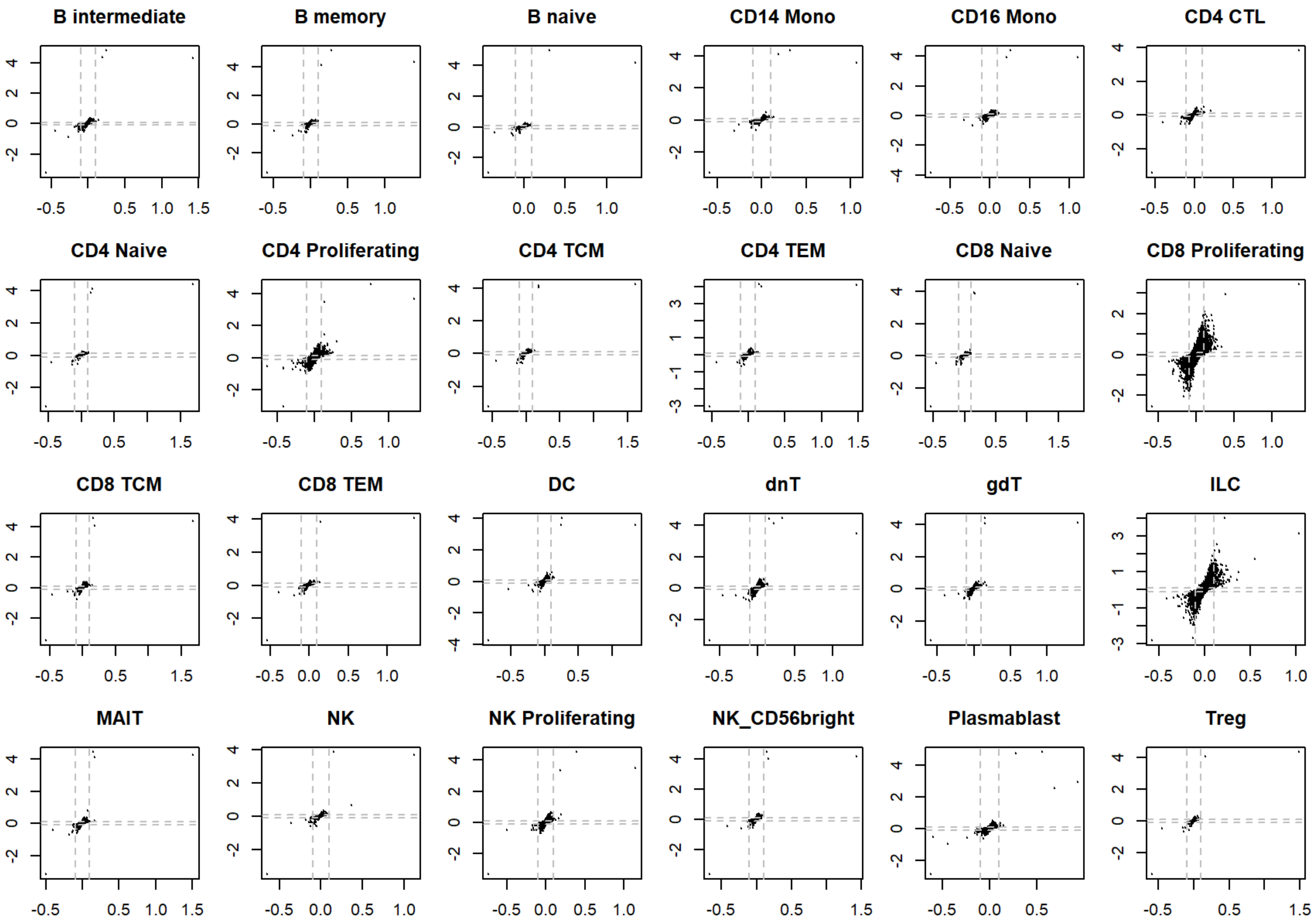

Figure S11 DEG comparison between Seurat v4 and v5: log2FC. For each cell type, the x-axis shows the log2FC from Seurat v4, and the y-axis shows the log2FC from Seurat v5. Note, only the minimal/intersect set between the two methods are shown.

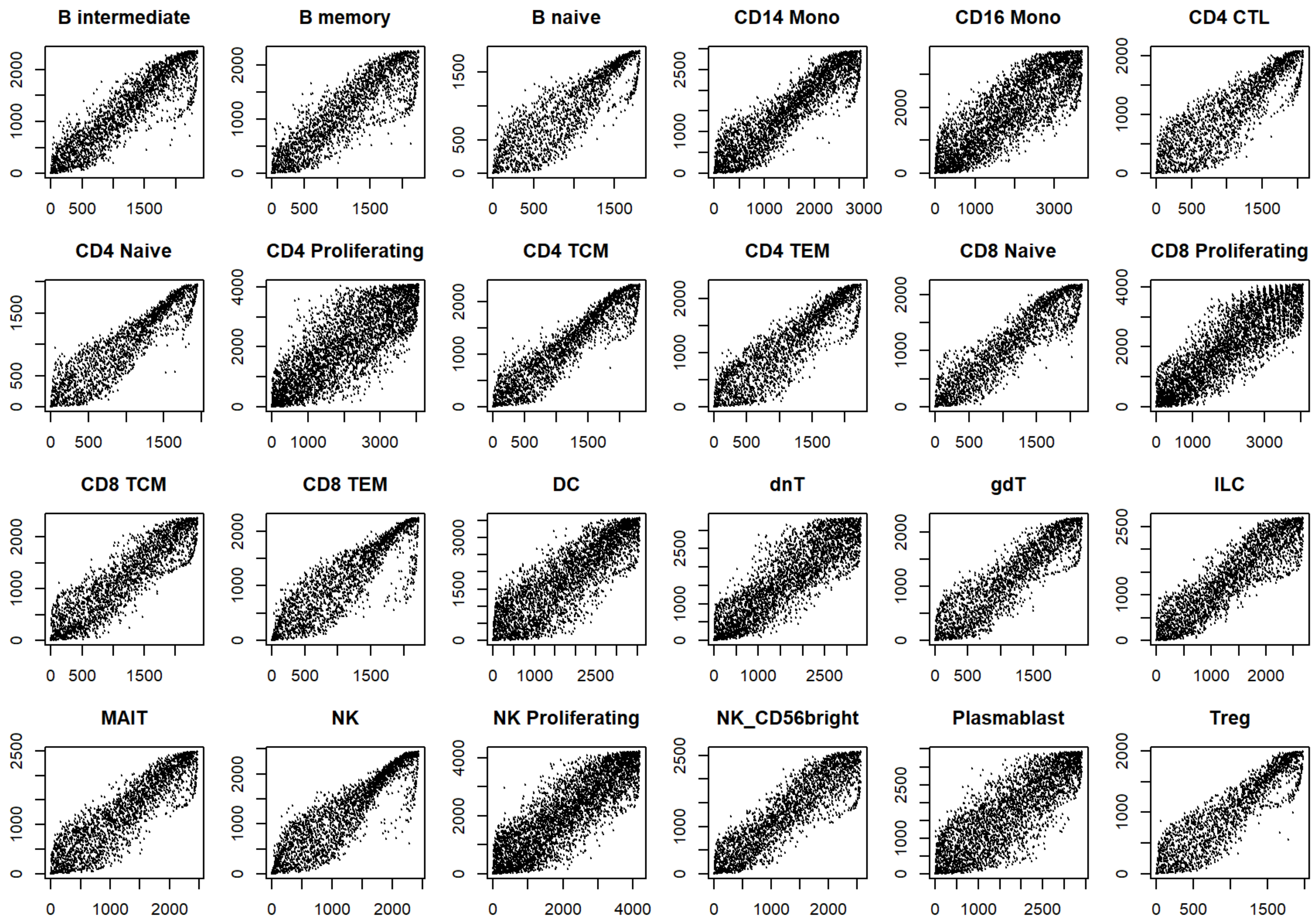

Figure S12 DEG comparison between Seurat v4 and v5: ranked log2FC. For each cell type, the x-axis shows the ranked log2FC from Seurat v4, and the y-axis shows the ranked log2FC from Seurat v5. Note, only the minimal/intersect set between the two methods are shown.

Supplemental Tables

Note:
Tables S1-4,9,14-15,17,20-21 are in the Supplemental Note
Tables S5-8,10-16,18-19, 22, 23 are in the Supplemental Spreadsheet

Table S1 Cell type counts and proportions
Table S2 Correlation of cell type proportions with age
Table S3 Correlation of cell type proportions with age – alternate models and tests
Table S4 Sex DEGs summary results per cell type and test
Table S5 List of sex DEGs per cell type
Table S6 Summary of DEG overlaps
Table S7 Downsampling DEGs statistics
Table S8 Downsampling unique counts
Table S9 Sex- specific eQTL summary results – autosomal
Table S10 Sex- specific eQTL results – autosomal – joint
Table S11 Sex- specific eQTL results – autosomal – female
Table S12 Sex- specific eQTL results – autosomal – male
Table S13 Sex- specific eQTL results – autosomal – interacting
Table S14 List of X-escape genes
Table S15 List of PAR genes
Table S16 List of MHC genes
Table S17 Sex- specific eQTL summary results – sex chromosomes
Table S18 Sex- specific eQTL results – PAR
Table S19 Sex- specific eQTL results – non-PAR
Table S20 Sex- specific eQTL and sex DEGs overlaps counts
Table S21 Sex- specific eQTL and sex DEGs overlap gene list
Table S22 Aggregate co-expression network clustering analysis
Table S23 Azimuth cell markers for L2 annotation

Table S1 Cell-type counts and proportions

	Cell-type	Cell counts			Mean proportions				
Stratification		*Total*	*Male*	*Female*	*Total*	*Male*	*Female*	FDR	Ratios
B-cells	B intermediate	33194	12440	20754	2.63%	2.40%	2.76%	1.45E-02	0.870
	B memory	23708	8582	15126	1.88%	1.63%	2.03%	9.78E-07	0.803
	B naive	71374	25965	45409	5.65%	4.98%	6.08%	2.99E-06	0.818
	Plasmablast	3602	1618	1984	0.29%	0.32%	0.28%	2.26E-01	1.169
CD4 T-cells	CD4 CTL	11680	5049	6631	0.92%	0.97%	0.92%	7.16E-01	1.056
	CD4 Naive	303555	114708	188847	24.02%	21.71%	25.35%	2.63E-06	0.856
	CD4 Proliferating	556	251	305	0.04%	0.05%	0.04%	1.62E-01	1.160
	CD4 TCM	251068	106156	144912	19.87%	20.04%	19.33%	2.26E-01	1.037
	CD4 TEM	24266	9665	14601	1.92%	1.80%	1.95%	5.14E-02	0.927
	Treg	28843	11502	17341	2.28%	2.18%	2.32%	1.45E-02	0.938
CD8 T-cells	CD8 Naive	57914	22248	35666	4.58%	4.17%	4.66%	1.32E-01	0.895
	CD8 Proliferating	256	124	132	0.02%	0.02%	0.02%	4.90E-03	1.330
	CD8 TCM	10724	4758	5966	0.85%	0.91%	0.79%	3.62E-02	1.143
	CD8 TEM	178401	79043	99358	14.12%	15.20%	13.76%	1.45E-02	1.105
Other T-cells	dnT	2939	1181	1758	0.23%	0.22%	0.24%	3.93E-01	0.946
	gdT	4883	2306	2577	0.39%	0.46%	0.35%	2.33E-01	1.320
	MAIT	12286	4511	7775	0.97%	0.85%	1.04%	3.73E-01	0.824
	ILC	315	115	200	0.02%	0.02%	0.03%	3.85E-01	0.804
Natural killer cells	NK	171757	79541	92216	13.59%	15.44%	12.65%	6.32E-08	1.221
	NK Proliferating	3215	1579	1636	0.25%	0.31%	0.22%	1.33E-08	1.365
	NK_CD56bright	8259	3112	5147	0.65%	0.60%	0.71%	3.99E-02	0.845
Monocytes	CD14 Mono	38908	18732	20176	3.08%	3.71%	2.84%	1.45E-02	1.306
	CD16 Mono	15341	7011	8330	1.21%	1.35%	1.15%	9.07E-02	1.175
Dendritic cells	DC	6596	3227	3369	0.52%	0.64%	0.48%	3.18E-06	1.324
	ASDC	*210*	*86*	*124*	*0.02%*	*0.02%*	*0.02%*	7.95E-01	*1.005*
	cDC1	*112*	*63*	*49*	*0.01%*	*0.01%*	*0.01%*	2.13E-02	*1.751*
	cDC2	*4330*	*2146*	*2184*	*0.35%*	*0.42%*	*0.31%*	4.69E-06	*1.359*
	pDC	*1944*	*932*	*1012*	*0.16%*	*0.19%*	*0.15%*	5.35E-03	*1.267*
Other	Eryth	580	232	348	*0.05%*	*0.04%*	*0.05%*	5.49E-01	*0.933*
	HSPC	1766	864	902	*0.14%*	*0.17%*	*0.12%*	8.12E-03	*1.365*
	Platelet	1773	741	1032	*0.14%*	*0.14%*	*0.14%*	5.54E-01	*1.023*
**Total**		**1265986**	**524520**	**741466**					

Table S2 Correlation of cell-type proportions with age

	Cell-type	Joint			Female			Male		
Stratification		**P-values**	**FDR**	**Rho**	**P-values**	**FDR**	**Rho**	**P-values**	**FDR**	**Rho**
B-cells	B intermediate	2.8E-08	3.9E-07	-0.18	3.8E-03	4.5E-02	-0.12	2.1E-07	4.2E-06	-0.25
	B memory	6.2E-13	1.2E-11	-0.23	4.7E-05	7.1E-04	-0.17	5.3E-10	1.2E-08	-0.30
	B naive	1.5E-03	1.5E-02	-0.10	2.7E-02	2.7E-01	-0.09	2.9E-02	3.2E-01	-0.11
	Plasmablast	4.4E-05	5.7E-04	-0.13	2.4E-04	3.1E-03	-0.15	4.7E-02	4.7E-01	-0.10
CD4 T-cells	CD4 CTL	4.4E-15	9.2E-14	0.25	5.3E-15	1.1E-13	0.32	3.0E-03	4.8E-02	0.15
	CD4 Naive	8.4E-11	1.5E-09	-0.21	4.2E-08	7.1E-07	-0.23	3.8E-04	6.8E-03	-0.17
	CD4 Proliferating	9.5E-01	1.0E+00	0.00	9.3E-01	1.0E+00	0.00	9.9E-01	1.0E+00	0.00
	CD4 TCM	7.4E-01	1.0E+00	-0.01	5.6E-01	1.0E+00	-0.02	9.4E-01	1.0E+00	0.00
	CD4 TEM	1.1E-02	8.6E-02	-0.08	3.6E-02	2.8E-01	-0.09	1.8E-01	1.0E+00	-0.07
	Treg	3.7E-02	1.9E-01	0.07	6.6E-01	1.0E+00	0.02	5.2E-03	7.8E-02	0.14
CD8 T-cells	CD8 Naive	2.9E-126	6.9E-125	-0.67	5.6E-75	1.3E-73	-0.67	4.1E-52	9.8E-51	-0.65
	CD8 Proliferating	1.1E-01	4.5E-01	0.05	2.2E-01	1.0E+00	0.05	3.4E-01	1.0E+00	0.05
	CD8 TCM	7.1E-13	1.4E-11	-0.23	1.4E-10	2.9E-09	-0.27	2.8E-04	5.4E-03	-0.18
	CD8 TEM	1.7E-09	2.7E-08	0.19	2.0E-08	3.7E-07	0.23	7.4E-03	9.6E-02	0.13
Other T-cells	dnT	4.2E-04	4.6E-03	-0.11	2.2E-02	2.5E-01	-0.10	6.6E-03	9.2E-02	-0.13
	gdT	1.0E-09	1.7E-08	-0.19	2.3E-09	4.4E-08	-0.25	1.1E-02	1.3E-01	-0.13
	MAIT	1.0E-26	2.4E-25	-0.33	4.3E-18	9.8E-17	-0.35	1.3E-09	2.7E-08	-0.29
	ILC	8.9E-03	8.0E-02	-0.08	6.4E-02	4.5E-01	-0.08	7.0E-02	6.3E-01	-0.09
Natural killer cells	NK	1.6E-24	3.6E-23	0.32	2.8E-16	6.2E-15	0.33	2.0E-10	4.7E-09	0.31
	NK Proliferating	6.3E-05	7.5E-04	0.13	7.4E-05	1.0E-03	0.17	1.4E-01	1.0E+00	0.07
	NK_CD56bright	2.3E-02	1.4E-01	-0.07	9.6E-02	5.7E-01	-0.07	1.8E-01	1.0E+00	-0.07
Monocytes	CD14 Mono	1.1E-02	8.6E-02	0.08	2.9E-02	2.7E-01	0.09	2.3E-01	1.0E+00	0.06
	CD16 Mono	1.0E-08	1.6E-07	0.18	2.0E-06	3.1E-05	0.20	1.2E-03	2.0E-02	0.16
Dendritic cells	DC	1.5E-01	4.5E-01	-0.05	4.2E-01	1.0E+00	-0.03	1.5E-01	1.0E+00	-0.07

Table S3 Correlation of cell-type proportions with age – alternate models and tests

	**F-test**			**Kruskal-Wallis**	**Beta regression**								
	Original	Covariate: age	Covariate: age + pcs		Original	Covariate: age		Covariate: age + pcs					
	sex	sex	sex	sex	sex	sex2	age	sex2	age	pc1	pc2	pc3	pc4
B intermediate	1.5E-02	1.7E-02	1.9E-02	2.5E-02	4.6E-02	4.2E-02	4.8E-04	1.2E-01	1.43E-03	0.92	1.00	0.88	0.88
B memory	9.8E-07	9.0E-07	1.2E-06	9.8E-07	2.2E-06	1.2E-06	1.0E-11	3.7E-06	3.00E-11	0.47	0.98	1.00	0.82
B naive	3.0E-06	2.9E-06	3.2E-06	6.4E-06	1.0E-05	7.3E-06	7.3E-03	2.2E-05	2.19E-02	0.20	0.99	0.96	0.69
CD14 Mono	1.5E-02	1.6E-02	1.9E-02	1.1E-02	3.0E-02	2.5E-02	6.2E-02	7.3E-02	1.60E-01	0.38	0.65	0.69	0.60
CD16 Mono	9.1E-02	1.1E-01	1.2E-01	4.8E-02	1.1E-01	1.0E-01	1.3E-08	2.4E-01	3.86E-08	0.30	0.92	0.16	1.00
CD4 CTL	7.2E-01	6.0E-01	5.9E-01	5.6E-01	5.8E-01	5.9E-01	3.8E-12	8.8E-01	1.13E-11	0.94	0.29	0.82	1.00
CD4 Naive	2.6E-06	2.7E-06	3.2E-06	2.8E-06	2.2E-06	1.2E-06	3.1E-10	3.7E-06	9.42E-10	1.00	0.10	0.64	0.82
CD4 Proliferating	1.6E-01	1.7E-01	1.9E-01	4.5E-01	3.4E-01	6.7E-01	6.7E-01	8.8E-01	8.86E-01	0.82	0.69	1.00	0.05
CD4 TCM	2.3E-01	2.2E-01	2.5E-01	2.2E-01	2.1E-01	2.0E-01	4.4E-01	4.3E-01	7.67E-01	0.16	0.88	0.87	0.68
CD4 TEM	5.1E-02	5.4E-02	5.4E-02	4.3E-02	6.3E-02	5.9E-02	2.7E-01	1.6E-01	5.64E-01	0.88	1.00	0.64	1.00
CD8 Naive	1.3E-01	1.1E-01	1.3E-01	5.0E-02	1.5E-01	1.4E-01	5.7E-131	3.2E-01	1.70E-130	0.17	1.00	0.92	1.00
CD8 Proliferating	4.9E-03	5.5E-03	4.9E-03	1.2E-02	2.0E-02	1.6E-02	2.2E-01	4.8E-02	4.72E-01	0.70	0.83	0.88	0.81
CD8 TCM	3.6E-02	2.2E-02	2.5E-02	4.3E-02	4.0E-02	3.6E-02	5.9E-10	1.0E-01	1.78E-09	0.70	0.88	0.92	0.88
CD8 TEM	1.5E-02	1.6E-02	1.9E-02	1.7E-02	2.0E-02	1.7E-02	1.3E-08	4.9E-02	3.76E-08	1.00	0.88	0.28	1.00
DC	3.2E-06	2.9E-06	3.2E-06	1.3E-06	4.4E-06	2.8E-06	3.7E-01	8.3E-06	6.87E-01	0.88	1.00	0.64	1.00
dnT	3.9E-01	4.3E-01	4.2E-01	4.4E-01	3.4E-01	3.6E-01	3.4E-05	6.8E-01	1.02E-04	0.98	0.79	0.98	0.56
gdT	2.3E-01	1.8E-01	1.9E-01	2.2E-01	3.4E-01	3.6E-01	2.2E-11	6.8E-01	6.57E-11	0.98	0.93	0.88	0.88
ILC	3.9E-01	4.2E-01	4.2E-01	7.2E-02	1.2E-01	1.2E-01	1.9E-02	2.8E-01	5.34E-02	0.92	0.82	1.00	1.00
MAIT	3.7E-01	4.3E-01	4.3E-01	1.4E-01	3.0E-01	2.9E-01	1.6E-27	6.0E-01	4.78E-27	0.38	1.00	0.88	0.88
NK	6.3E-08	3.2E-08	2.7E-08	1.0E-07	1.2E-07	3.9E-08	2.7E-20	1.2E-07	8.14E-20	0.82	0.91	0.88	0.77
NK Proliferating	1.3E-08	1.5E-08	1.7E-08	1.0E-07	3.2E-08	7.2E-09	6.2E-02	2.2E-08	1.60E-01	0.94	0.34	0.84	1.00
NK_CD56bright	4.0E-02	4.3E-02	4.7E-02	1.1E-02	2.0E-02	1.7E-02	1.3E-01	4.9E-02	3.00E-01	0.77	0.44	0.64	0.88
Plasmablast	2.3E-01	1.8E-01	1.9E-01	3.6E-01	3.0E-01	2.9E-01	1.5E-06	6.0E-01	4.50E-06	0.88	0.82	0.92	0.88
Treg	1.5E-02	1.6E-02	1.4E-02	1.2E-02	2.0E-02	1.7E-02	4.9E-02	4.9E-02	1.37E-01	0.15	0.23	1.00	0.38

Table S4 Sex DEGs summary results per cell-type and test

**Cell-type**	**Wilcoxon-test**	**MAST**	**MAST - age adjusted**

Stratification		Male	X	Y	Female	X	**Total**	Male	X	Y	Female	X	**Total**	Male	X	Y	Female	X	**Total**
B-cells	B intermediate	5	1	3	28	6	33	5	1	3	28	6	33	5	1	3	28	6	33
	B memory	3	0	3	7	4	10	3	0	3	7	4	10	3	0	3	7	4	10
	B naive	2	0	2	13	5	15	2	0	2	13	5	15	2	0	2	13	5	15
	Plasmablast	5	1	3	7	5	12	5	1	3	7	5	12	5	1	3	7	5	12
CD4 T-cells	CD4 CTL	8	2	1	19	5	27	8	2	1	19	5	27	8	2	1	19	5	27
	CD4 Naive	5	0	3	5	5	10	5	0	3	5	5	10	5	0	3	5	5	10
	CD4 Proliferating	3	0	3	4	4	7	3	0	3	4	4	7	3	0	3	4	4	7
	CD4 TCM	4	0	3	6	5	10	4	0	3	6	5	10	4	0	3	6	5	10
	CD4 TEM	6	1	3	7	4	13	6	1	3	7	4	13	6	1	3	7	4	13
	Treg	3	0	2	5	5	8	3	0	2	5	5	8	3	0	2	5	5	8
CD8 T-cells	CD8 Naive	5	0	3	8	5	13	5	0	3	8	5	13	5	0	3	8	5	13
	CD8 Proliferating	2	0	2	2	2	4	2	0	2	2	2	4	2	0	2	2	2	4
	CD8 TCM	5	0	3	6	4	11	5	0	3	6	4	11	5	0	3	6	4	11
	CD8 TEM	4	0	2	10	3	14	4	0	2	10	3	14	4	0	2	10	3	14
Other T-cells	dnT	4	0	4	7	7	11	4	0	4	7	7	11	4	0	4	7	7	11
	gdT	5	0	3	5	4	10	5	0	3	5	4	10	5	0	3	5	4	10
	MAIT	5	1	3	9	4	14	5	1	3	9	4	14	5	1	3	9	4	14
	ILC	1	0	1	2	2	3	1	0	1	2	2	3	1	0	1	2	2	3
Natural killer cells	NK	4	0	2	16	3	20	4	0	2	16	3	20	4	0	2	16	3	20
	NK Proliferating	3	0	3	6	5	9	3	0	3	6	5	9	3	0	3	6	5	9
	NK_CD56bright	3	0	3	7	4	10	3	0	3	7	4	10	3	0	3	7	4	10
Monocytes	CD14 Mono	5	0	3	21	3	26	5	0	3	21	3	26	5	0	3	21	3	26
	CD16 Mono	5	0	3	12	2	17	5	0	3	12	2	17	5	0	3	12	2	17
Dendritic cells	DC	7	1	3	5	4	12	7	1	3	5	4	12	7	1	3	5	4	12
Other	Eryth	1	0	1	2	2	3	1	0	1	2	2	3	1	0	1	2	2	3
	HSPC	3	0	3	2	2	5	3	0	3	2	2	5	3	0	3	2	2	5
	Platelet	3	0	2	3	2	6	3	0	2	3	2	6	3	0	2	3	2	6
**Average**		4.0	0.3	2.6	8.3	3.9	12.3	4.0	0.3	2.6	8.3	3.9	12.3	4.0	0.3	2.6	8.3	3.9	12.3

Table S9 Sex- specific eQTL summary results – autosomal

	**Cell-type**	**Total* eQTLs**			**Novel****			**Interacting**
Stratification		Joint	Female	Male	Female-specific analysis only	Male-specific analysis only	Joint analysis only	
B-cells	B intermediate	433	148	120	21	18	433	0
	B memory	365	127	102	19	18	365	3
	B naive	856	365	249	56	39	856	5
	Plasmablast	49	18	12	3	2	49	1
CD4 T-cells	CD4 CTL	62	28	30	5	5	62	4
	CD4 Naive	2824	1554	1249	263	208	2824	9
	CD4 TCM	2845	1389	1335	227	227.	2845	1
	CD4 TEM	306	94	82	16	14	306	4
	Treg	395	150	129	23	22	395	0
CD8 T-cells	CD8 Naive	628	280	202	48	32	628	1
	CD8 TCM	125	48	41	7	7	125	1
	CD8 TEM	1782	775	778	120	128	1782	8
Other T-cells	dnT	24	10	8	2	1	24	0
	gdT	34	12	16	2	3	34	2
	MAIT	143	45	48	8	1	143	0
Natural killer cells	NK	2264	936	1056	144	171	2264	4
	NK Proliferating	47	14	14	2	2	47	1
	NK_CD56bright	135	51	42	8	6	135	0
Monocytes	CD14 Mono	546	186	217	29	35	509	3
	CD16 Mono	464	158	207	24	33	445	4
Dendritic cells	DC	161	36	65	3	10	161	0
Total		14488	6424	6002	1038	990	14432	51

*** After cell type specific FDR correction (local FDR <0.05)**

****After global multiple testing correction (global q-value<0.05)**

Table S17 Sex- specific eQTL summary results – sex chromosomes

	Celltype	PAR1			PAR2			PAR			non-PAR			Escapees	Total		
Stratification		Joint	Female	Male	Joint	Female	Male	Joint	Female	Male	Joint	Female	Male	Female	Joint	Female	Male
B-cells	B intermediate	0	0	0	0	0	0	0	0	0	4	0	0	0	4	0	0
	B memory	0	0	0	0	0	0	0	0	0	1	0	0	0	1	0	0
	B naive	2	1	0	1	0	0	3	1	0	16	2	4	1	19	3	4
	Plasmablast	0	0	0	1	0	0	1	0	0	3	2	1	0	4	2	1
CD4 T-cells	CD4 CTL	0	0	0	0	0	0	0	0	0	1	1	1	0	1	1	1
	CD4 Naive	2	1	1	0	0	0	2	1	1	38	26	25	1	40	27	26
	CD4 TCM	2	2	1	0	0	0	2	2	1	57	3	36	1	59	5	37
	CD4 TEM	2	1	1	0	0	0	2	1	1	18	22	2	5	20	23	3
	Treg	1	2	1	0	0	0	1	2	1	12	4	4	0	13	6	5
CD8 T-cells	CD8 Naive	2	0	0	0	0	0	2	0	0	17	4	4	0	19	4	4
	CD8 TCM	0	0	0	0	0	0	0	0	0	5	1	1	0	5	1	1
	CD8 TEM	1	0	0	1	0	0	2	0	0	34	13	14	1	36	13	14
Other T-cells	dnT	0	0	0	0	0	0	0	0	0	0	0	0	0	0	0	0
	gdT	0	0	0	1	0	0	1	0	0	3	1	1	0	4	1	1
	MAIT	0	0	0	0	0	0	0	0	0	3	2	1	0	3	2	1
Natural killer cells	NK	1	1	0	1	0	0	2	1	0	36	15	27	3	38	16	27
	NK Proliferating	0	0	0	0	0	0	0	0	0	1	0	2	0	1	0	2
	NK_CD56bright	0	0	0	0	0	0	0	0	0	1	0	0	0	1	0	0
Monocytes	CD14 Mono	1	0	0	0	0	0	1	0	0	12	1	1	1	13	1	1
	CD16 Mono	0	0	0	1	0	0	1	0	0	5	0	0	0	6	0	0
Dendritic cells	DC	0	0	0	0	0	0	0	0	0	0	0	0	3	0	0	0
Total		14	8	4	6	0	0	20	8	4	267	97	124	16	287	105	128

Table S20 Sex- specific eQTL and sex DEGs overlaps counts

	Autosomal eQTLs						Sex chromosome eQTLs				
	**Female-biased DEGs**			**Male-biased DEGs**			**Female-biased DEGs**			**Male-biased DEGs**	
	Female	*Joint*	*Interacting*	*Male*	*Joint*	*Interacting*	*Female X*	*Female X (including escape)*	*Joint X*	*Male XY*	*Joint X*
B intermediate	0	2	0	0	0	0	0	0	0	0	0
B memory	0	0	0	0	0	0	0	0	0	0	0
B naive	0	0	0	0	0	0	0	1	0	0	0
Plasmablast	0	0	0	0	0	0	0	0	0	0	0
CD4 CTL	0	0	0	0	0	0	0	0	0	0	0
CD4 Naive	0	0	0	0	0	0	1	2	0	0	0
CD4 TCM	0	0	0	0	0	0	1	2	1	0	0
CD4 TEM	0	1	0	0	0	0	0	0	0	0	0
Treg	0	0	0	0	0	0	0	1	0	0	0
CD8 Naive	0	0	0	0	0	0	0	0	0	0	0
CD8 TCM	0	0	0	0	0	0	0	0	0	0	0
CD8 TEM	0	2	0	0	0	0	0	0	0	0	0
dnT	0	0	0	0	0	0	0	0	0	0	0
gdT	0	0	0	0	0	0	0	0	0	0	0
MAIT	0	0	0	0	0	0	0	0	0	0	0
NK	1	6	0	0	1	0	0	0	0	0	0
NK Proliferating	0	1	0	0	0	0	0	0	0	0	0
NK_CD56bright	0	1	0	0	0	0	0	0	0	0	0
CD14 Mono	0	4	1	0	0	0	0	0	0	0	0
CD16 Mono	0	2	0	0	0	0	0	0	0	0	0
DC	0	0	0	0	0	0	0	1	0	0	0
**Totals**	1	19	1	0	1	0	2	7	1	0	0

Table S21 Sex- specific eQTL and sex DEGs overlap gene list

	Cell type	Gene	rsID		eQTL	DEG
**Autosomal**	B_intermediate	*CD83*	rs16874672	6:14087484_T	Joint	female biased expression
	B_intermediate	*CIB1*	rs9745131	15:90776154_T	Joint	female biased expression
	CD14_Mono	*CSTA*	rs67931976	3:122105715_G	Joint	female biased expression
	CD14_Mono	*ITGB2*	rs760462	21:46328099_C	Joint	female biased expression
	CD14_Mono	*LGALS2*	rs7291467	22:37972628_A	Joint	female biased expression
	CD14_Mono	*S100A8*	rs60752752	1:153339782_G	Joint	female biased expression
	CD16_Mono	*ANXA1*	rs10124476	9:75889773_C	Joint	female biased expression
	CD16_Mono	*IFITM2*	rs11246057	11:295808_G	Joint	female biased expression
	CD4_TEM	*GZMK*	rs7714681	5:54291899_T	Joint	female biased expression
	CD8_TEM	*GZMA*	rs2047745	5:54489541_C	Joint	female biased expression
	CD8_TEM	*HOPX*	rs35867715	4:57627999_C	Joint	female biased expression
	NK	*FCER1G*	rs11421	1:161188936_C	Joint	female biased expression
	NK	*FCGR3A*	rs6700241	1:161500975_G	Joint	female biased expression
	NK	*GZMA*	rs62361890	5:55052519_A	Joint	female biased expression
	NK	*HOPX*	rs4865137	4:57525669_C	Joint	female biased expression
	NK	*IFITM2*	rs1059091	11:309127_G	Joint	female biased expression
	NK	*PTGDS*	rs2271869	9:139848273_G	Joint	**male biased expression**
	NK	*XCL2*	rs141673522	1:168517508_T	Joint	female biased expression
	NK_CD56bright	*DONSON*	rs11088258	21:35034050_A	Joint	female biased expression
	NK_Proliferating	*LAIR2*	rs73070113	19:55014172_T	Joint	female biased expression
	NK	*FCGR3A*	rs2099684	1:161500130_G	Female	female biased expression
	CD14_Mono	*ITGB2*	rs760462	21:46328099_C	Interacting	female biased expression
**Sex chromosomes**						
	CD4_Naive	*SEPT6*		X:119505884_G	Female	female biased expression
	CD4_TCM	*SEPT6*		X:117782171_A	Female	female biased expression
**Escape genes**						
	B_naive	*RPS4X*		X:71468142_G	Female	female biased expression
	CD4_Naive	*EIF2S3*		X:24130140_C	Female	female biased expression
	CD4_TCM	*EIF2S3*		X:24419789_G	Female	female biased expression
	DC	*XIST*		X:73702119_C	Female	female biased expression
	Treg	*XIST*		X:72050435_C	Female	female biased expression

Supplemental Methods

Co-expression network generation

For each batch, we generated individual expression matrices using the normalized count data for each cell-type and sex. We used the most recurrent genes across all the data to ensure a common gene reference, totalling 14,716 genes. Then, we built a gene co-expression network from each of these expression matrices, using Spearman's correlation to measure gene-gene relationships. Due to low cell-numbers for some cell-types, we built networks for 21 of the 27 cell-types. Each network was then ranked to standardize the correlations. We then aggregated the same cell-type per sex to build meta-analytic co-expression networks. We also built individual cell-type specific networks that were not conditioned on sex, and similarly aggregated these networks (labelled "joint"). For additional analyses, we then built "PBMC" aggregates from the cell-type aggregates, for the joint and individual sexes. Finally, we built networks from each individual, removing Erythrocytes, HSPC and Platelets. We aggregated individuals by their genotyped sex. Overall, we generated 70 aggregate networks.

Differential co-expression analysis

To compare across networks, we assessed network differences based on overall topology, node degrees and functional performances. For each network, we took the correlation of the matrices as a measure of topological similarity. We calculated the node degrees of each network by summing the weights of each node and then took the correlation of these node degrees. And finally, using the outputs from the GO slim analysis, we calculated the similarity of their performances by taking the correlation of the AUROCs of the functional groups. To identify the changes by genes and gene modules, we calculated the frequency of genes whose co-expression ranking changed by at least 0.5 between the networks. With these tallies, we identified which genes had the most topological shifts. Secondly, we repeated this with the node degree of the genes, calculating standardised residuals and selecting outliers that were three standard deviations away.

Differential expression analysis - globalFDR

For an additional analysis, we calculated a global FDR. We calculated a gene-level P-value using the Simes procedure on the raw p-values across all cell types where the gene was expressed. For genes not expressed within that cell type, we used a value of 1. We then performed global multiple testing correction across all genes using the Benjamini-Hochberg (BH) method. We filtered for significance using the global FDR of 0.05 and the cell type specific |log2FC| > 0.1.

Downsampling differential expression analysis

For each cell-type, we downsampled the number of cells (repeated) and ran the differential expression analysis using the FindMarkers function. With this we calculated the number of significant genes and the number of genes that overlap with the full set of cells for that cell-type.

Supplemental References

1. Klein, S.L., and Flanagan, K.L. (2016). Sex differences in immune responses. Nat Rev Immunol 16, 626-638.
2. Márquez, E.J., Chung, C.-H., Marches, R., Rossi, R.J., Nehar-Belaid, D., Eroglu, A., Mellert, D.J., Kuchel, G.A., Banchereau, J., and Ucar, D. (2020). Sexual-dimorphism in human immune system aging. Nat Commun 11, 751.
3. Taneja, V. (2018). Sex Hormones Determine Immune Response. Front Immunol 9, 1931.
4. Mitchell, E., Spencer Chapman, M., Williams, N., Dawson, K.J., Mende, N., Calderbank, E.F., Jung, H., Mitchell, T., Coorens, T.H.H., Spencer, D.H., et al. (2022). Clonal dynamics of haematopoiesis across the human lifespan. Nature 606, 343-350.
5. Hazeldine, J., and Lord, J.M. (2013). The impact of ageing on natural killer cell function and potential consequences for health in older adults. Ageing Res Rev 12, 1069-1078.
6. Chen, G., Lustig, A., and Weng, N.-P. (2013). T Cell Aging: A Review of the Transcriptional Changes Determined from Genome-Wide Analysis. Front Immunol 0.
7. Churov, A.V., Mamashov, K.Y., and Novitskaia, A.V. (2020). Homeostasis and the functional roles of CD4+ Treg cells in aging. Immunology Letters 226, 83-89.
8. Ballouz, S., Verleyen, W., and Gillis, J. (2015). Guidance for RNA-seq co-expression network construction and analysis: safety in numbers. Bioinformatics 31, 2123-2130.
9. Ballouz, S., Weber, M., Pavlidis, P., and Gillis, J. (2017). EGAD: ultra-fast functional analysis of gene networks. Bioinformatics 33, 612-614.
10. Ashburner, M., Ball, C.A., Blake, J.A., Botstein, D., Butler, H., Cherry, J.M., Davis, A.P., Dolinski, K., Dwight, S.S., Eppig, J.T., et al. (2000). Gene ontology: tool for the unification of biology. The Gene Ontology Consortium. Nat Genet 25, 25-29.
11. Gene Ontology, C. (2021). The Gene Ontology resource: enriching a GOld mine. Nucleic Acids Res 49, D325-D334.
12. Farahbod, M., and Pavlidis, P. (2020). Untangling the effects of cellular composition on coexpression analysis. Genome Res 30, 849-859.
13. Subramanian, A., Tamayo, P., Mootha, V.K., Mukherjee, S., Ebert, B.L., Gillette, M.A., Paulovich, A., Pomeroy, S.L., Golub, T.R., Lander, E.S., et al. (2005). Gene set enrichment analysis: a knowledge-based approach for interpreting genome-wide expression profiles. Proc Natl Acad Sci U S A 102, 15545-15550.
14. Liberzon, A., Birger, C., Thorvaldsdóttir, H., Ghandi, M., Mesirov, J.P., and Tamayo, P. (2015). The Molecular Signatures Database (MSigDB) hallmark gene set collection. Cell Syst 1, 417-425.
15. Kanehisa, M., and Goto, S. (2000). KEGG: Kyoto Encyclopedia of Genes and Genomes. Nucleic Acids Res 28, 27-30.
16. Gillespie, M., Jassal, B., Stephan, R., Milacic, M., Rothfels, K., Senff-Ribeiro, A., Griss, J., Sevilla, C., Matthews, L., Gong, C., et al. (2021). The reactome pathway knowledgebase 2022. Nucleic Acids Res 50, D687-D692.
17. Nishimura, D. (2001). BioCarta. Biotech softw Internet rep 2, 117-120.
18. So, J., Tai, A.K., Lichtenstein, A.H., Wu, D., and Lamon-Fava, S. (2021). Sexual dimorphism of monocyte transcriptome in individuals with chronic low-grade inflammation. Biology of Sex Differences 12, 43.
19. Varghese, M., Clemente, J., Lerner, A., Abrishami, S., Islam, M., Subbaiah, P., and Singer, K. (2022). Monocyte Trafficking and Polarization Contribute to Sex Differences in Meta-Inflammation. Frontiers in Endocrinology 13.
20. Kolmykov, S., Yevshin, I., Kulyashov, M., Sharipov, R., Kondrakhin, Y., Makeev, V.J., Kulakovskiy, I.V., Kel, A., and Kolpakov, F. (2021). GTRD: an integrated view of transcription regulation. Nucleic Acids Research 49, D104-D111.
21. Ma, X., Zhang, H., Yuan, L., Jing, H., Thacker, P., and Li, D. (2011). CREBL2, interacting with CREB, induces adipogenesis in 3T3-L1 adipocytes. Biochemical Journal 439, 27-38.
